# PFKM‐Driven Lactate Overproduction Promotes Atrial Fibrillation via Triggering Cardiac Fibroblasts Histone Lactylation

**DOI:** 10.1002/advs.202500963

**Published:** 2025-06-26

**Authors:** Ning Fang, Ning Zhang, Xiaohui Jiang, Sen Yan, Zhiqi Wang, Qianhui Gao, Mingcheng Xu, Lin Mu, Xiaoming Li, Jiuling Chen, Song Zhang, Yu Duan, Fengxiang Yun, Luyifei Li, Yun Zhang, Yongtai Gong

**Affiliations:** ^1^ Department of Cardiology The First Affiliated Hospital Harbin Medical University Harbin 150001 China; ^2^ Key Laboratory of Cardiac Diseases and Heart Failure Harbin Medical University Harbin 150001 China; ^3^ Institute of Metabolic Disease Heilongjiang Academy of Medical Science Harbin 150001 China; ^4^ Heilongjiang Key Laboratory for Metabolic Disorder & Cancer Related Cardiovascular Diseases Harbin 150081 China; ^5^ Department of Clinical Laboratory Shanghai Tenth People's Hospital School of Medicine Tongji University Shanghai 200072 China

**Keywords:** atrial fibrillation, glycolysis, histone lactylation, H3K18la, PFKM

## Abstract

Increasing evidence has clarified that atrial fibrillation (AF) is associated with enhanced glycolysis, leading to lactate accumulation. However, whether glycolysis promotes AF remains unknown, as does whether histone lactylation plays a role in its pathogenesis. In the study, spontaneous AF mice are established to monitor AF susceptibility and atrial substrates at different ages (3, 5, 7 months), indicating that enhanced glycolysis acts as a promoter during AF development by inducing atrial fibrosis. The promoting effect of glycolysis on AF and the pivotal enzyme in driving glycolysis are confirmed by treatment with glycolysis inhibitor 2‐deoxyglucose (2‐DG) and adeno‐associated virus‐mediated atrial PFKM expression. Furthermore, lactate stimulates primary mouse cardiac fibroblast (CF) activation. Mechanistically, the observations indicated that atrial lactate accumulation promotes global lactylation and H3K18 lactylation in atrial fibroblasts. P300‐mediated H3K18 lactylation up‐regulates TGF‐β1 transcription, leading to activation of CF, and thereby contributing to atrial fibrosis. The results reveal a novel role of the metabolic‐epigenetic axis in AF pathogenesis, which raises the possibility of potential therapeutic strategies targeting AF.

## Graphical abstract

The upregulation of PFKM activates glycolysis, thereby enhancing lactate production in atrial tissue. Atrial lactate accumulation promotes global lactylation and H3K18 lactylation in atrial fibroblasts. P300‐mediated H3K18 lactylation up‐regulates TGF‐β1 transcription, leading to fibroblast activation and thereby contributing to atrial fibrosis. The findings highlight a new role of the metabolic‐epigenetic axis in AF pathogenesis, suggesting potential therapeutic strategies for targeting AF.

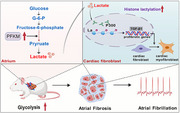

## 1 Introduction

Atrial fibrillation (AF) is the most common arrhythmia in clinical practice, associated with high morbidity and mortality, affecting 2%‐4% of the general population.^[^ ^1^, ^2^ ^]^ While the mechanisms of AF, such as structural,^[^ ^3^ ^]^ electrical,^[^ ^4^ ^]^ and autonomic remodeling are well established, emerging evidence indicates that metabolic remodeling may also increase the risk of AF.^[^ ^5^, ^6^, ^7^ ^]^ During AF, atrial energy mainly derives from glycolysis, as evidenced by increased expression of key glycolytic enzymes and lactate accumulation in atrial tissue.^[^ ^8^, ^9^ ^]^ This suggests that enhanced glycolysis serves as a major metabolic remodeling to meet the increased energy demands of fibrillating atria in AF development.^[^ ^10^ ^]^ However, whether glycolysis contributes to AF development and the underlying mechanisms remains unclear.

Lactate, the end product of glycolysis, was previously considered as a by‐product of metabolism.^[^ ^11^, ^12^ ^]^ However, growing evidence has demonstrated that lactate plays an important role as a signaling molecule. Zhang et al. found that lactate directly modifies histones by attaching a lactyl group to lysine (K) residues.^[^ ^13^ ^]^ This modification process is referred to as “lactylation,” which ultimately influences gene transcription. Histone lactylation has been implicated in the activation of repair genes post‐myocardial infarction^[^ ^14^ ^]^, enhanced vascular calcification,^[^ ^15^ ^]^ and regulation of heart failure development.^[^ ^16^ ^]^ However, it is currently unclear whether histone lactylation also contributes to the occurrence and development of AF.

Atrial fibrosis represents the fundamental pathological feature of the persistence of AF and has been recognized as the typical hallmark of AF. Atrial fibrosis is induced by activated cardiac fibroblasts (CF), targeting CF activation has emerged as a potential therapeutic target for AF.^[^ ^17^, ^18^ ^]^ Recent studies have shown that histone lactylation drives fibrotic progression in pulmonary diseases and cancer by activating lung fibroblast and tumor‐associated fibroblast proliferation.^[^ ^19^, ^20^ ^]^ However, the role of histone lactylation in cardiac fibroblast activation in AF has not been investigated.

In this study, we identified and verified the promoting effect of glycolysis on AF and elucidated the mechanism by which lactate activates CF by promoting histone lactylation‐mediated TGF‐β1 transcription. Our findings provide a new perspective on the mechanism of the metabolome‐epigenome‐fibrosis cascade during AF development.

## 2 Results

### 2.1 Enhanced Atrial Glycolysis Increases AF Susceptibility in CREM Mice

**Figure 1:**
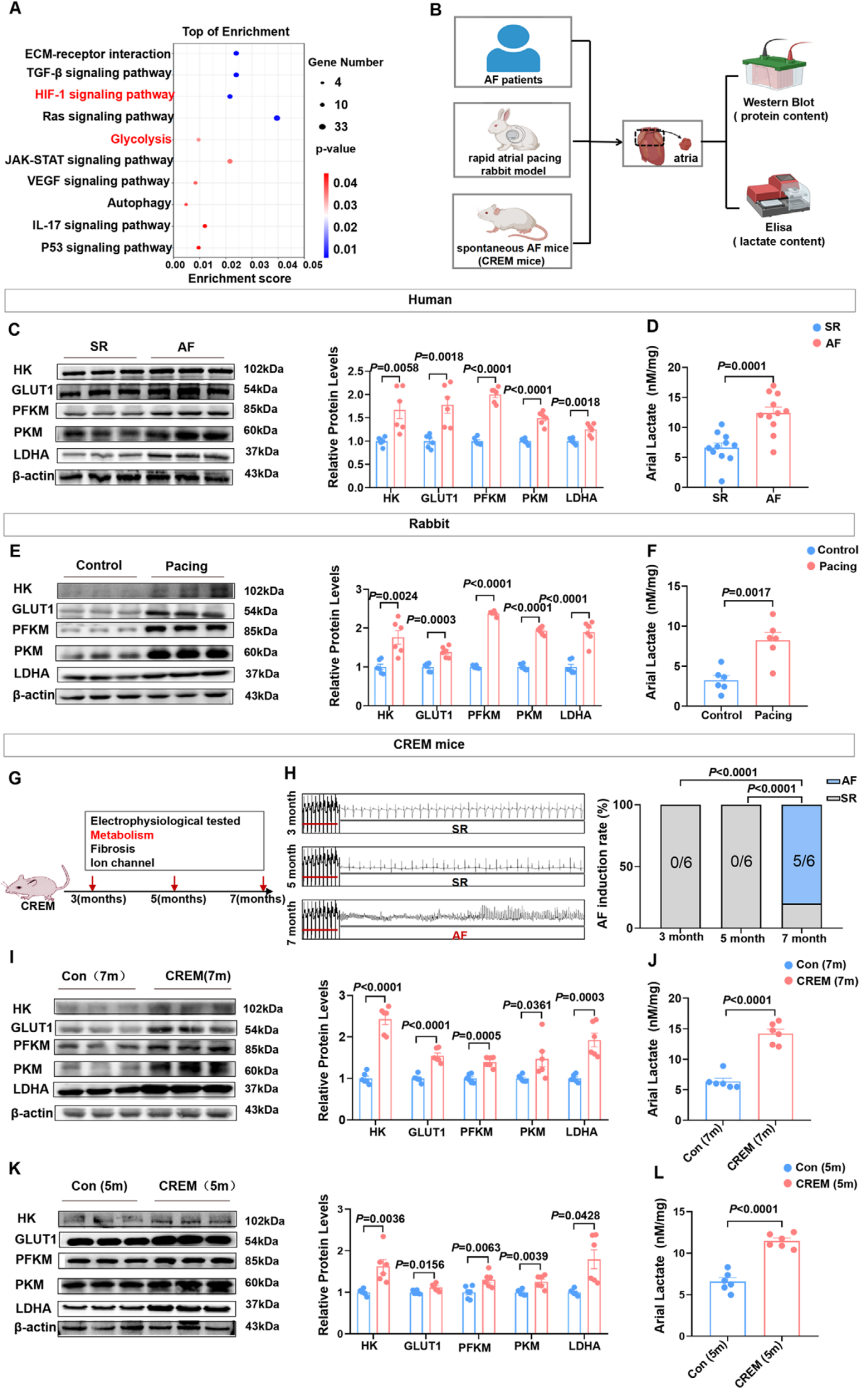
Glycolysis is associated with enhanced AF in atrial pacing rabbits, CREM mice and human. A) The results of KEGG pathway analysis from GEO database (GSE128188) with atrium in people with AF and sinus rhythm (n = 5). B) Schematic diagram of atrial tissue obtained from AF patients, rapid atrial pacing rabbits and CREM mice for the detection of glycolytic pathway and lactate content. C) Representative bands and quantification of the protein levels of HK, GLUT1, PFKM, PKM and LDHA in atrial tissue with AF and sinus rhythm (n = 6). D) The atrial concentration of lactate (n = 11). E) Representative bands and quantification of the protein levels of GLUT1, HK, PFKM, PKM and LDHA in atrial tissue from rabbits in control group and rapid atrial pacing group (n = 6). F) The atrial concentration of lactate (n = 6). G) Schematic diagram showing dynamic monitoring of the pathological mechanism of AF in CREM mice at different months of age. H) Representative examples of AF induction attempt in CREM mice and AF inducibility (n = 6). I) Representative bands and quantification of the protein levels of GLUT1, HK, PFKM, PKM, and LDHA in atria of 7‐month CREM mice (n = 6). J) The atrial concentration of lactate (n = 6). K) Representative bands and quantification of the protein levels of HK, GLUT1, PFKM, PKM, and LDHA in atria of CREM mice in 5 months (n = 6). L) The atrial concentration of lactate (n = 6). SR = sinus rhythm; AF = atrial fibrillation; Con = control; Pacing = atrial rapid pacing. The data are given as mean ± SEM and compared by Student's *t* test (C, D, E, F, I, J, K, and L) and one‐way ANOVA (H). The diagram was created in https://BioRender.com.

To verify the link between AF and glycolysis, we analyzed the GEO database (GSE128188) (**Figure** **1**A). As shown in Figure 1A, analysis using the KEGG pathway revealed that atrial tissue in the AF population was primarily enriched in the glycolysis pathway. We further confirmed the changes of glycolysis pathway in atrial tissues across multiple experimental models, including AF patients (Table S1, Figure S1, Supporting Information), a rapid atrial pacing rabbit model, and a spontaneous AF mouse model (CREM mice) (Figure 1B, Figure S2, Supporting Information). Analysis revealed significant upregulation of key glycolytic enzymes including hexokinase 2 (HK2), glucose transporter 1 (GLUT1), phosphofructokinase muscle type (PFKM), pyruvate kinase M2 (PKM2), and lactate dehydrogenase A (LDHA), along with elevated lactate accumulation in atrial tissues of AF patients compared to sinus rhythm controls (Figure 1C,D). Furthermore, the expression of key glycolytic enzyme proteins and lactate content was up‐regulated in the atria of rapid atrial pacing rabbits (Figure 1E,F).

We established CREM mice to mimic the natural disease evolution typically seen in AF patients^[^ ^21^, ^22^, ^23^ ^]^ (Figure S3A,B) and performed atrial electrophysiological testing and atrial substrate detection, including glycolysis, atrial fibrosis, and ion channels at sequential time points (3, 5, and 7 months of age) (Figure 1G). We found that atrial burst pacing did not induce AF at 3 and 5 months of age in CREM mice. However, 7‐month‐old CREM mice displayed a significantly elevated AF inducibility (Figure 1H, S4A, Supporting Information). Relative to age‐matched controls, 7‐month‐old CREM mice exhibited significant elevations in both glycolytic enzyme proteins and atrial lactate accumulation, which is consistent with the population studies (Figure 1I,J). Meanwhile, higher atrial collagen deposition and collagen volume fraction were observed in 7‐month‐old CREM mice than in 7‐month‐old control mice (Figure S5A, Supporting Information). Correspondingly, compared to the control group, the CREM group demonstrated an upregulation of fibrosis‐related proteins, including collagen III, transforming growth factor‐beta 1 (TGF‐β1), and alpha‐smooth muscle actin (α‐SMA) (Figure S5B, Supporting Information). In addition, we analyzed ATP levels, reactive oxygen species (ROS), apoptosis, and ion channel proteins in atrial tissues of both groups. Our results revealed that CREM mice were accompanied by impaired ATP production and increased ROS levels (Figure S5 C,D, Supporting Information). However, there were no significant alterations in ion channel proteins and apoptosis‐related proteins at 7 months of age (Figure S5 E,F, Supporting Information).

Subsequently, we evaluated mice at the age of 5 months for a similar analysis. Interestingly, no significant difference in atrial fibrosis and ion channel proteins was detected between control and CREM groups, except for the up‐regulation of the glycolysis pathway (Figure 1K,L, Figure S6A–D, Supporting Information). These results suggest that glycolysis may act as a mechanism to promote the occurrence of AF. To determine whether reduction of glycolysis would ameliorate AF susceptibility, the glycolysis inhibitor 2‐DG was administered via intraperitoneal injection for 8 weeks (Figure S7A, Supporting Information). 2‐DG suppressed the atrial glycolysis pathway and lactate production (Figure S7B,C, Supporting Information). Atrial electrophysiological testing revealed a decrease in AF induction and duration in mice administered with 2‐DG relative to PBS‐injection (Figure S7D–F, Supporting Information). These findings suggested that 2‐DG treatment decreased susceptibility to AF. Echocardiography demonstrated that inhibition of glycolysis reduced left atrial diameter (Figure S7G, Supporting Information). Hematoxylin and eosin (HE) staining of the left atrial tissues revealed well‐organized fibers in the 2‐DG group, characterized by a lack of intercellular spaces, whereas the PBS‐treated group displayed disordered myocardial fibers with hypertrophic and edematous cardiomyocytes. Masson's trichrome staining demonstrated significantly decreased left atrial collagen deposition and a lower collagen volume fraction in the left atrium of the 2‐DG group mice compared to the control group mice (Figure S7H, Supporting Information). Meanwhile, inhibition of glycolysis with 2‐DG ameliorated fibrosis‐related genes and proteins upregulation in atrial tissues (Figure S7I,J, Supporting Information). Taken together, our results suggest that enhanced glycolysis increases AF susceptibility in CREM mice.

### 2.2 PFKM‐Mediated Glycolysis Activation Drives AF Through Promoting Atrial Fibrosis

**Figure 2:**
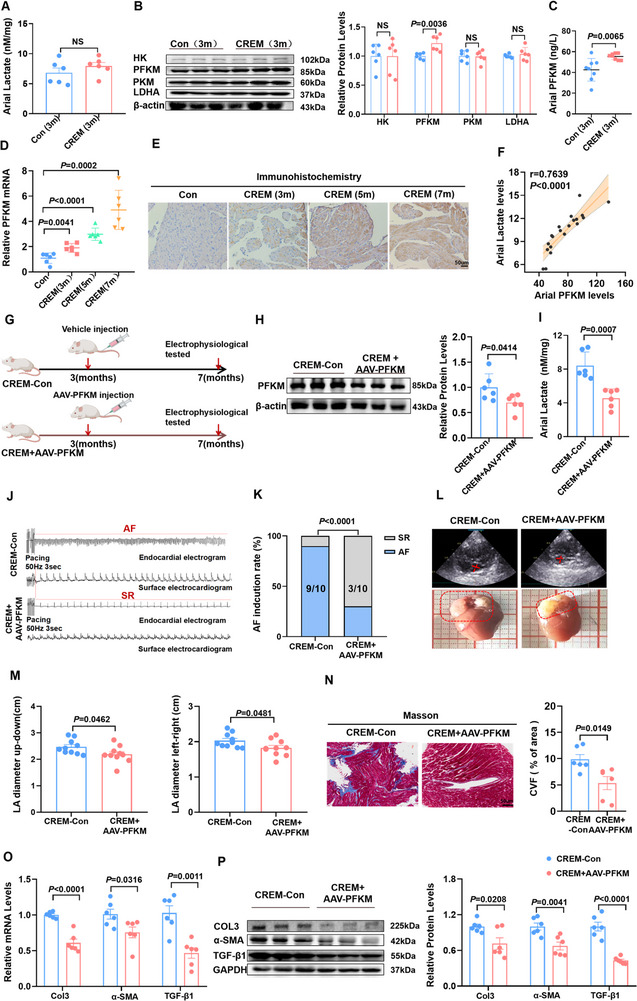
PFKM knockdown alleviates glycolysis and decreases AF susceptibility in CREM mice. A) The atrial concentration of lactate (n = 6). B) Representative bands and quantification of the protein levels of HK, PFKM, PKM, and LDHA in atrial tissue 3‐month control and 3‐month CREM mice (n = 6). C) The atrial concentration of PFKM in two groups (n = 8). D) qPCR validation of the relative abundance of PFKM in 3‐month, 5‐month, and 7‐month CREM mice left atrial tissue (n = 6). (E) Representative images of atrial Immunohistochemistry staining of mice. Scale bar = 50um **F)** The correlation between expression of PFKM and atrial lactate levels (n = 20). G) Schematic diagram showing the establishment of the PFKM‐knockout model in CREM mice. H) Representative bands and quantification of the protein levels of PFKM in two groups (n = 6). I) The atrial concentration of lactate (n = 6). J) Representative examples of AF induction attempt in the CREM‐Con and in the CREM + AAV‐PFKM group. K) AF inducibility. L) Representative echocardiography and images of LA dimensions in the CREM‐Con and CREM+AAV‐PFKM group. M) LA diameters in the two groups (n = 10). N) Representative images of atrial Masson staining and the collagen volume fraction in atria of two groups. (n = 6). Scale bar = 50um O) qPCR validation of the relative abundance of Col3, α‐SMA, and TGF‐β1 in atrial tissue with two groups (n = 6). P) Representative bands and quantification of the protein levels of Col3, α‐SMA and TGF‐β1 in atrial tissue with two groups (n = 6). AF = atrial fibrillation; Con = control; LA = left atrium. The data are given as mean ± SEM and compared by Student's *t* test (A, B, C, H, I, M, N, O, and P), one‐way ANOVA (D). AF inducibility **(K) **was compared by Fisher exact test. The diagram was created in https://BioRender.com.

Next, we attempted to explore the key enzymes regulating the enhanced glycolysis. At 3 months of age, the atrial lactate content in the CREM mice showed an upward trend compared to the control group, but it did not reach statistical significance (**Figure** **2**A). We further examined the atrial content of glycolytic enzymes and found that only PFKM protein levels showed a significant increase in expression (Figure 2B,C, Figure S8A–C, Supporting Information). Importantly, we observed an age‐dependent increase in PFKM expression, which was positively correlated with progressive atrial lactate accumulation in the atrium of CREM mice (Figure 2D–F, Figure S8D, Supporting Information). These results establish PFKM as a key enzyme contributing to the up‐regulation of glycolysis.

We further explored whether early intervention targeting PFKM expression in the atria could reduce the AF risk of CREM mice (Figure 2G). We knocked down PFKM by injecting adeno‐associated virus (AAV) (Figure 2H). We found that the atrial PFKM knockdown mice resulted in a reduction in atrial lactate (Figure 2I). Atrial electrophysiological testing revealed a decrease in AF induction and duration in CREM mice administered with AAV‐mediated PFKM knockdown compared to the control group (Figure 2J,K, Figure S8A, Supporting Information). Furthermore, AAV‐knockdown PFKM significantly attenuated left atrial enlargement (Figure 2L,M), while also markedly decreased left atrial fibrosis (Figure 2N–P), and led to an improvement in the disordered arrangement of myocardial fibers (Figure S9B, Supporting Information). AAV‐mediated knockdown of PFKM did not produce any significant effects on the overall function or volume of the ventricles (Figure S10A–H, Supporting Information). These findings suggest that the knockdown of PFKM plays a crucial role in alleviating glycolytic activity and subsequently reduces the susceptibility to AF in the CREM mouse model.

**Figure 3:**
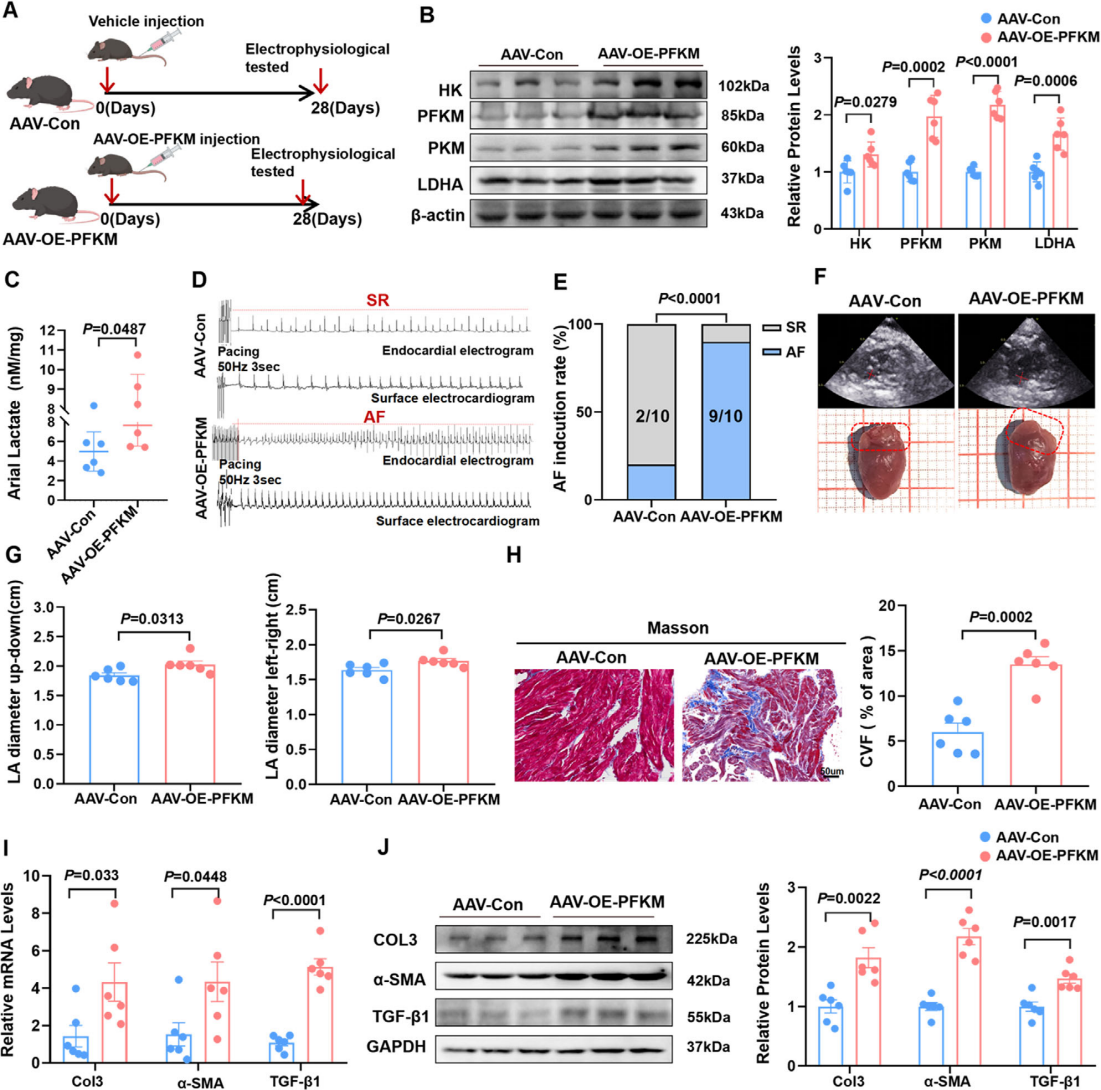
PFKM‐mediated activation of glycolysis is the primary driver of AF. A) Schematic diagram showing the establishment of atrial overexpression PFKM model in mice. Mice were injected with adeno‐associated virus through the tail vein, and AF susceptibility was tested 28 days later. The control group was treated with placebo. B) Representative bands and quantification of the protein levels of HK, PFKM, PKM and LDHA in two groups (n = 6). C) The atrial concentration of lactate (n = 6). D,E) Representative examples of AF induction attempts and AF inducibility in two groups (n = 10). F) Representative echocardiography and images of LA dimensions in the AAV‐Con and AAV‐OE‐PFKM group. G) LA diameters in the two groups (n = 6). H) Representative images of atrial Masson's staining and the collagen volume fraction in atria of two groups (n = 6). Scale bar = 50um I) qPCR validation of the relative abundance of Col3, α‐SMA, and TGF‐β1 in atrial tissue with two groups (n = 6). J) Representative bands and quantification of the protein levels of Col3, α‐SMA and TGF‐β1 in atrial tissue with two groups (n = 6). GAPDH was used for normalization. The data are given as mean ± SEM and compared by Student's *t* test (B,C,G, H, I and J). AF inducibility (E) was compared by the Fisher exact test. The diagram was created in https://BioRender.com.

To elucidate whether glycolysis activation is the primary driver of AF, we overexpressed atrial PFKM levels by injecting adeno‐associated virus in C57BL/6 mice (**Figure** **3**A). AAV‐mediated overexpression of atrial PFKM (AAV‐OE‐PFKM) enhanced atrial glycolysis, as evidenced by elevated levels of glycolytic enzymes and lactate accumulation (Figure 3B,C). In vivo electrophysiological study demonstrated that AAV‐OE‐PFKM mice exhibited higher AF inducibility and prolonged AF duration (Figure 3D,E, Figure S11A, Supporting Information). Echocardiography analysis revealed a significant increase in left atrium diameter in the AAV‐OE‐PFKM group compared to the control group (Figure 3F,G). HE staining of the left atrial tissues revealed well‐organized fibers in the control group, whereas the AAV‐OE‐PFKM group displayed disordered myocardial fibers with hypertrophic and edematous cardiomyocytes (Figure S11B, Supporting Information). Meanwhile, these AAV‐OE‐PFKM mice were observed to undergo significant atrial fibrotic remodeling, characterized by a notable increase in collagen deposition (Figure 3H) and upregulated fibrotic markers, including collagen III, TGF‐β1, and α‐SMA (Figure 3I,J). Notably, PFKM overexpression exerted no discernible influence on ventricular morphology or function, including IVSD, IVSS, LVIDD, LVIDS, LVPWD, LVPWS, and LVEF (Figure S12A–H, Supporting Information).

Building on our observations at the animal level, we found that enhanced glycolysis induces atrial fibrosis and ultimately increases AF susceptibility in mice. In contrast, the suppression of glycolysis appears to attenuate these detrimental pathological manifestations, suggesting a protective effect. Collectively, these findings establish that PFKM‐mediated glycolysis‐driven atrial fibrosis serves as a pivotal contributor to the pathogenesis of AF.

### 2.3 Lactate Induces Cardiac Fibroblasts Activation

**Figure 4:**
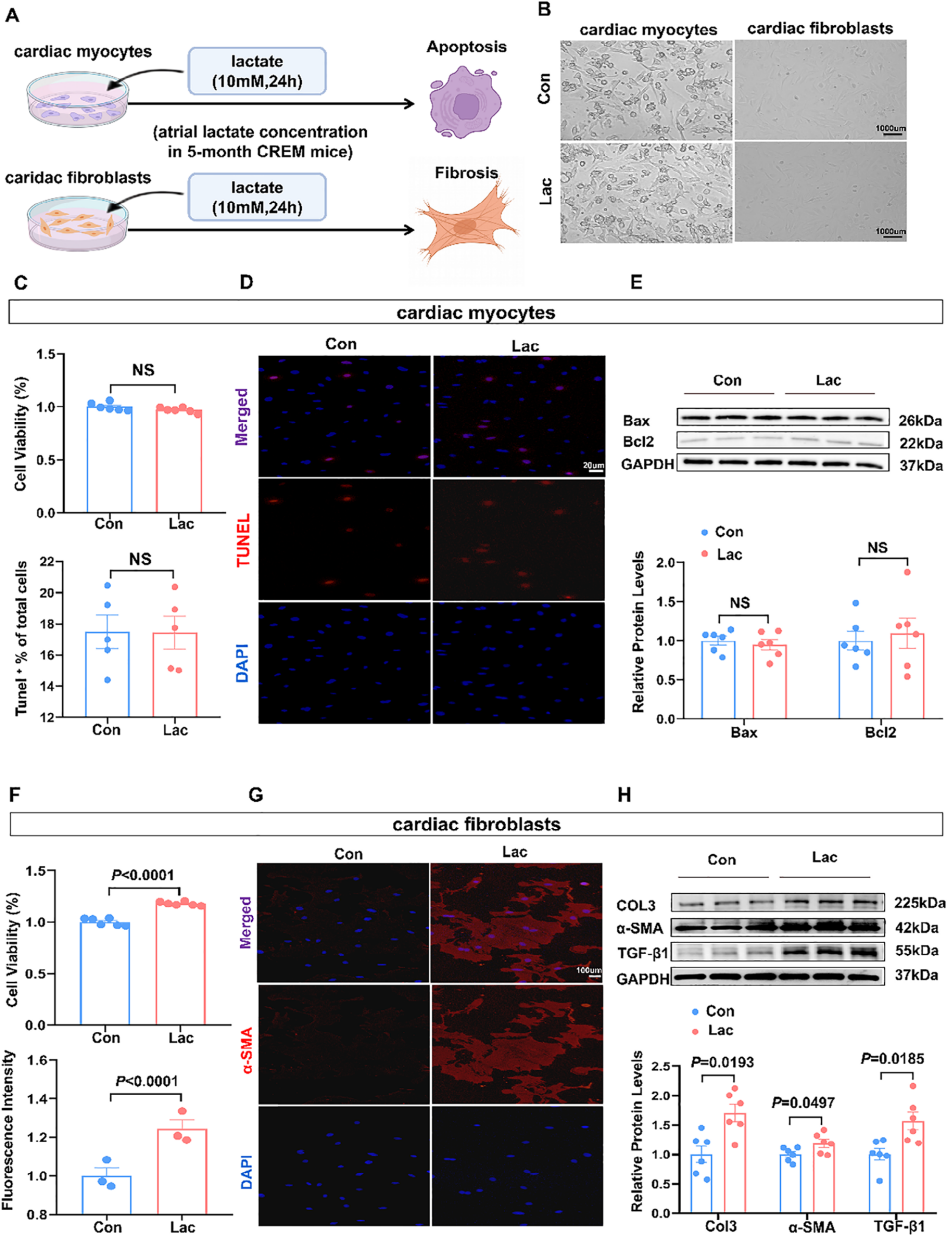
Lactate induces cardiac fibroblasts activation. A) Schematic representation of atrial‐derived lactate concentrations treated for cardiac fibroblasts and cardiac myocytes. B) Representative optical microscope image showed the morphology of cardiac fibroblasts and cardiac myocytes treated with control and lactate. Scale Bar = 1000um. C) The cell viability of CM (n = 6). **D)** Representative images of Tunel^+^ staining of CM (n = 5). Scale Bar = 20um. E) Representative bands and quantification of the protein levels of Bax and Bcl2 in CM (n = 6). F) The cell viability of CF in two groups (n = 6). G) Representative images of α‐SMA staining of CF (n = 3). Scale Bar = 100um. H) Representative bands and quantification of the protein levels of Col3, α‐SMA and TGF‐β1 in CF (n = 6). Con = control; lac = lactate. The data are given as mean ± SEM and compared by Student's *t* test (C, E, F, G, and H). The diagram was created in https://BioRender.com.

Lactate, the terminal metabolite of glycolysis, acts as an important signaling molecule that affects various cellular processes. To investigate the mechanism by which lactate promotes AF, we subjected primary cardiomyocytes (CM) and cardiac fibroblasts (CF) to lactate at concentration of 10 mm (a concentration equivalent to atrial lactate levels in 5‐month‐old CREM mice) (**Figure** **4**A). Morphological analysis under light microscopy revealed no structural alterations in either lactate‐treated CMs or CFs (Figure 4B). Concurrently, lactate exerted no effects on the viability and apoptosis of CM, indicated by unchanged expression of Bcl2, Bax, and Tunel positive cells in CM (Figure 4C–E). However, the viability of CF in the lactate group was significantly increased compared to the control group (Figure 4F). Immunofluorescent staining showed increased α‐SMA expression following lactate treatment (Figure 4G). Western blot analysis further confirmed upregulation of fibrosis‐related proteins in lactate group (Figure 4H). These findings indicated that lactate at a concentration comparable to 5‐month‐old CREM mice atrium is sufficient to trigger CF activation, but has no influence on CM apoptosis.

### 2.4 Enhanced Glycolysis Promotes Cardiac Fibroblasts Histone Lactylation in Atrium

**Figure 5:**
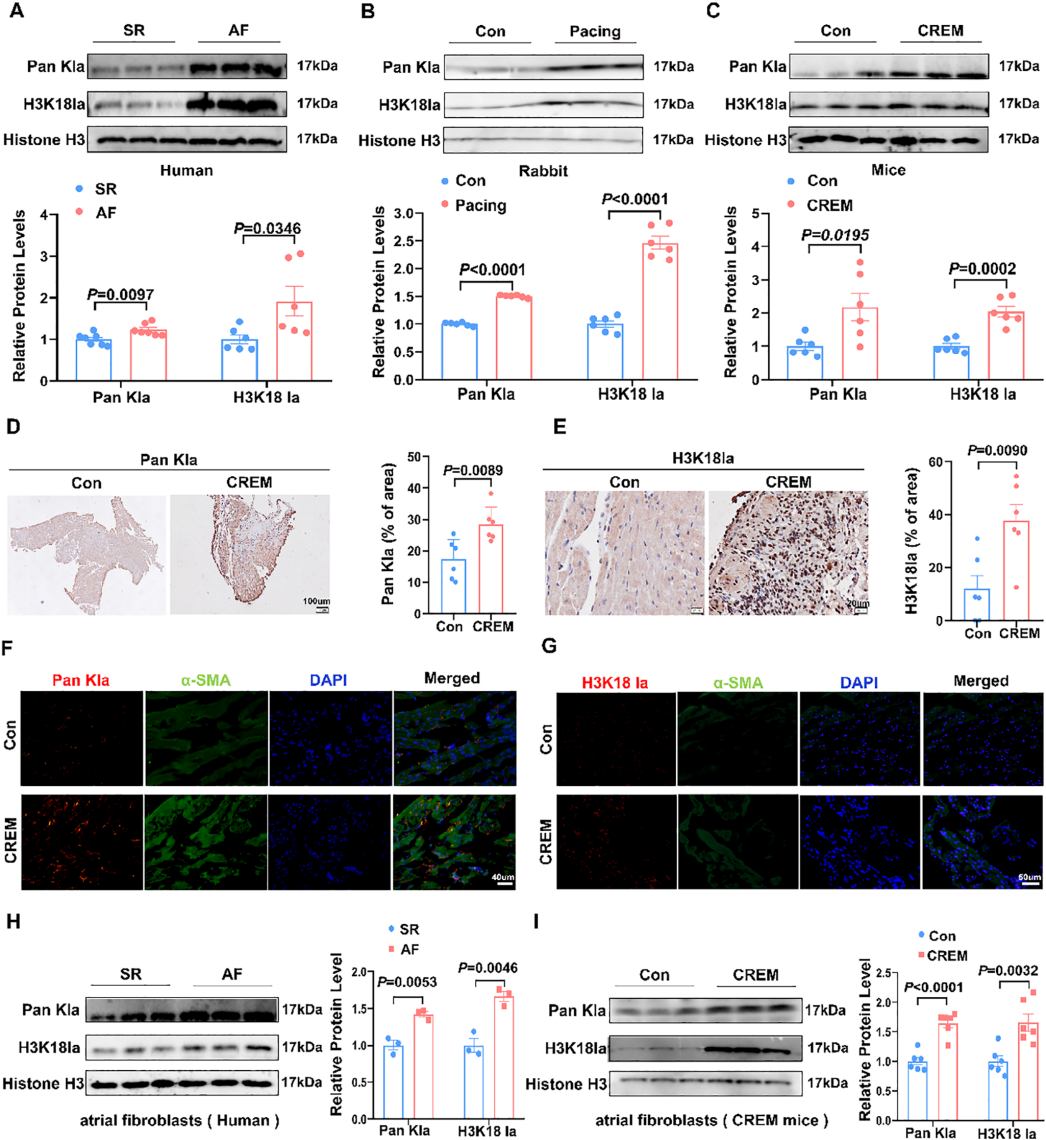
Enhanced glycolysis promotes cardiac fibroblasts histone lactylation in the atrium. A) Representative bands and quantification of the protein levels of Pan Kla (n = 7) and H3K18la (n = 6) in atria with SR and AF people. B) Representative bands and quantification of the protein levels of Pan Kla and H3K18la in atria with Con and rapid atrial pacing rabbit (n = 6). C) Representative bands and quantification of the protein levels of Pan Kla and H3K18la in atria with Con and 7‐month CREM mice (n = 6). D) Representative Pan Kla immunohistochemistry staining of Con and 7‐month CREM mice groups (n = 6). Scale bar = 100um. E) Representative H3K18 lactylation immunohistochemistry staining of Con and 7‐month CREM mice groups (n = 6). Scale bar = 20um. F) Immunofluorescence co‐staining for α‐SMA (green) with Pan Kla (red) in the atria with control and h CREM group. Scale bar = 40um. G) Immunofluorescence co‐staining for α‐SMA (green) with H3K18 la (red) in the atria with control and 7‐month CREM group. Scale bar = 50um. H) Representative bands and quantification of the protein levels of Pan Kla and H3K18la in CF isolated from SR and AF individuals (n = 3). I) Representative bands and quantification of the protein levels of Pan Kla and H3K18la in CF isolated from Con and 7‐month CREM mice (n = 6). The data are given as mean ± SEM and compared by Student's *t* test (A, B, C, D, E, H, and I) or Wilcoxon test (A, C, and E).

Lactate‐derived histone lactylation, an epigenetic modification, is implicated in cellular differentiation and activation across diverse cell types such as pulmonary smooth muscle cells, macrophages, and endothelial cells. We then explored whether histone lactylation is involved in AF development. Western blot analysis revealed that global lactylation levels in atrial tissue from AF patients, rapid atrial pacing rabbits and 7‐month‐old CREM mice were significantly increased compared to the sinus rhythm control group. We subsequently examined histone H3K18 lactylation (H3K18la), which exhibited a similar trend to global lactylation levels (**Figure** **5**A–C). Immunohistochemical staining of atrium from 7‐month‐old CREM mice showed significantly increased levels of global lactylation and H3K18la (Figure 5D,E). Interestingly, immunofluorescence staining of 7‐month‐old CREM atrial tissue showed that global lactylation and H3K18la were localized to the nuclear region of activated CF (Figure 5F,G). We further validated the expression of global lactylation and H3K18la in atrial fibroblasts isolated from individuals with AF and 7‐month‐old CREM mice through western blot analysis, which demonstrated elevated levels of these proteins compared to the control groups (Figure 5H,I). Additionally, the regulator effect of glycolysis on global lactylation and H3K18la levels in both atrial tissue and fibroblasts isolated from atrial tissue was confirmed by AAV‐mediated overexpression and knockdown of atrial PFKM, establishing a direct mechanistic link between metabolic remodeling and epigenetic in AF pathogenesis (Figure S13A–D, Supporting Information). Collectively, these data indicated that enhanced glycolysis promotes cardiac fibroblasts histone lactylation in the atrium.

### 2.5 H3K18la Regulates TGF‐β1 Transcription to Activate CF

**Figure 6:**
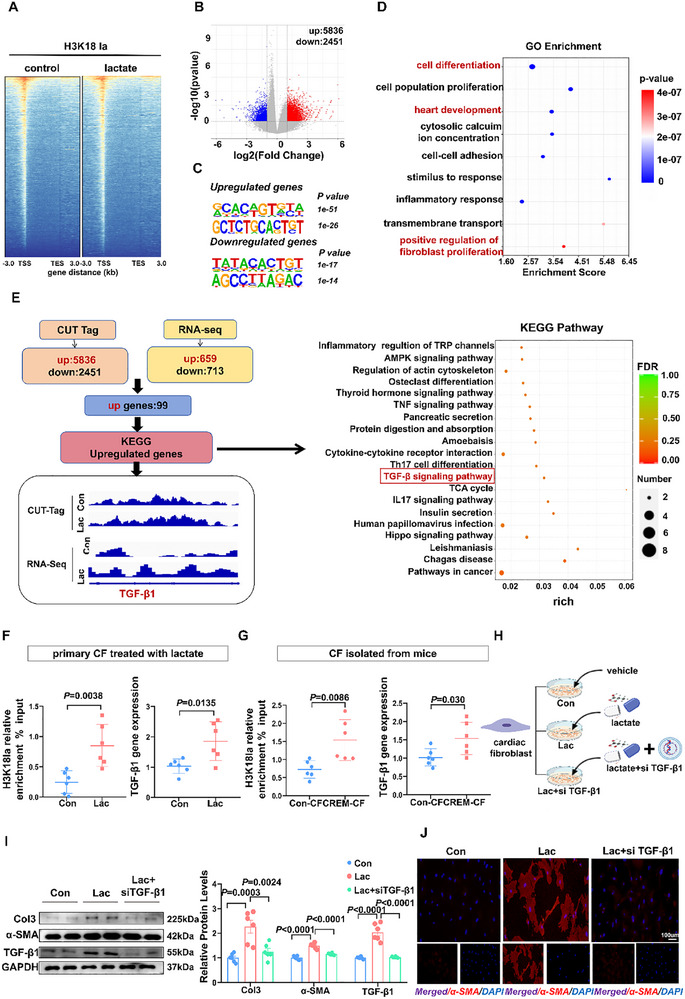
H3K18la regulates TGF‐β1 transcription to activate CF. A) Heatmaps for H3K18la binding peaks in control and lactate‐treated CF from mice. Color depth indicates the relative number of reads; genes with similar distribution patterns are clustered together through a clustering algorithm to show the binding trends of lactylation modifications on all genes. B) Volcano plot of differentially expressed genes in two groups, red represents up‐regulated genes and blue represents down‐regulated genes (n = 3). C) The top 2 enriched de novo motifs of the upregulated and downregulated genes with differential H3K18la modification. D) Bubble chart showing the top gene ontology terms of upregulated genes with increased H3K18la modification. E) Bioinformatics analysis filtered TGF‐β1 as downstream targets of H3K18la. Normalized read densities for H3K18la and RNA‐Seq at the TGF‐β1 gene. F) Gene expression analysis by RT‐qPCR and H3K18la occupancy analysis by ChIP‐qPCR in control and lactate treated CF (n = 6). G) Gene expression analysis by RT‐qPCR and H3K18la occupancy analysis by ChIP‐qPCR in CF isolated from the atrium in control and 7‐month CREM mice (n = 6). H) Schematic diagram about cells divided into control group, lactate group, and lactate +si TGF‐β1 group to observe the activation of CF induced by lactate. I) Representative bands and quantification of protein levels of Col3, α‐SMA and TGF‐β1 in CF (n = 6). J) Representative images of α‐SMA staining of CF. Scale bar = 100 µm. The data are given as mean ± SEM and compared by Student's *t* test (F and G), or one‐way ANOVA (I). The diagram was created in https://BioRender.com.

To identify candidate target genes regulated by H3K18la in lactate‐treated CF, we performed a CUT&Tag analysis using an anti‐H3K18la antibody. The data indicated that H3K18la was significantly enriched in the promoter regions and upstream areas of genes (**Figure** **6**A). ≈10% of the total H3K18la binding peaks were localized within ±1 kb of transcription start sites, whereas over 30% were distributed within ±10 kb (Figure S14A, Supporting Information). A total of 8287 differentially expressed genes were identified between lactate‐treated and control groups, with upregulated and downregulated genes displaying different enriched DNA sequences (Figure 6B,C). KEGG analysis revealed that H3K18la modified genes were predominantly associated with immune, endocrine and circulatory systems, as well as cardiovascular diseases (Figure S14B, Supporting Information). Additionally, Gene Ontology term analysis showed that genes modified by H3K18la were mainly involved in cell differentiation, cardiac development, and fibroblast proliferation (Figure 6D).

To uncover the potential mechanism responsible for histone lactylation mediated‐CF activation, we cross‐referenced the CUT&Tag and RNA‐seq data using KEGG analysis. We identified 99 significantly upregulated genes in lactate‐treated groups compared to controls, which were primarily enriched in TGF‐β signaling pathway, with marked elevation of TGF‐β1 expression (Figure 6E). Emerging evidence strongly implicates TGF‐β1 as a central regulator in cardiac pathophysiology processes, including fibrotic remodeling, fibroblast activation, and extracellular matrix deposition.^[^ ^24^, ^25^ ^]^ Subsequent validation through qRT‐PCR and ChIP‐qPCR assays confirmed the increased expression and H3K18la enrichment in the promoter regions of TGF‐β1. Compared to the control group, lactate promotes TGF‐β1 gene expression and H3K18la enrichment in the promoter regions in CF (Figure 6F). Similarly, we found the same trend in atrial fibroblasts extracted from AF mice (Figure 6G). We further suppressed TGF‐β1 expression by small interfering RNA (siRNA) to verify whether lactate acts by regulating TGF‐β1 (Figure 6H). We discovered that lactate‐induced CF activation was significantly reduced when the expression of TGF‐β1 was inhibited (Figure 6I,J). The data combined indicated that H3K18la regulates the activation of CF by TGF‐β1 transcription.

### 2.6 P300 Serves as a Writer for H3K18 la to Mediate TGF‐β1 Transcription

**Figure 7:**
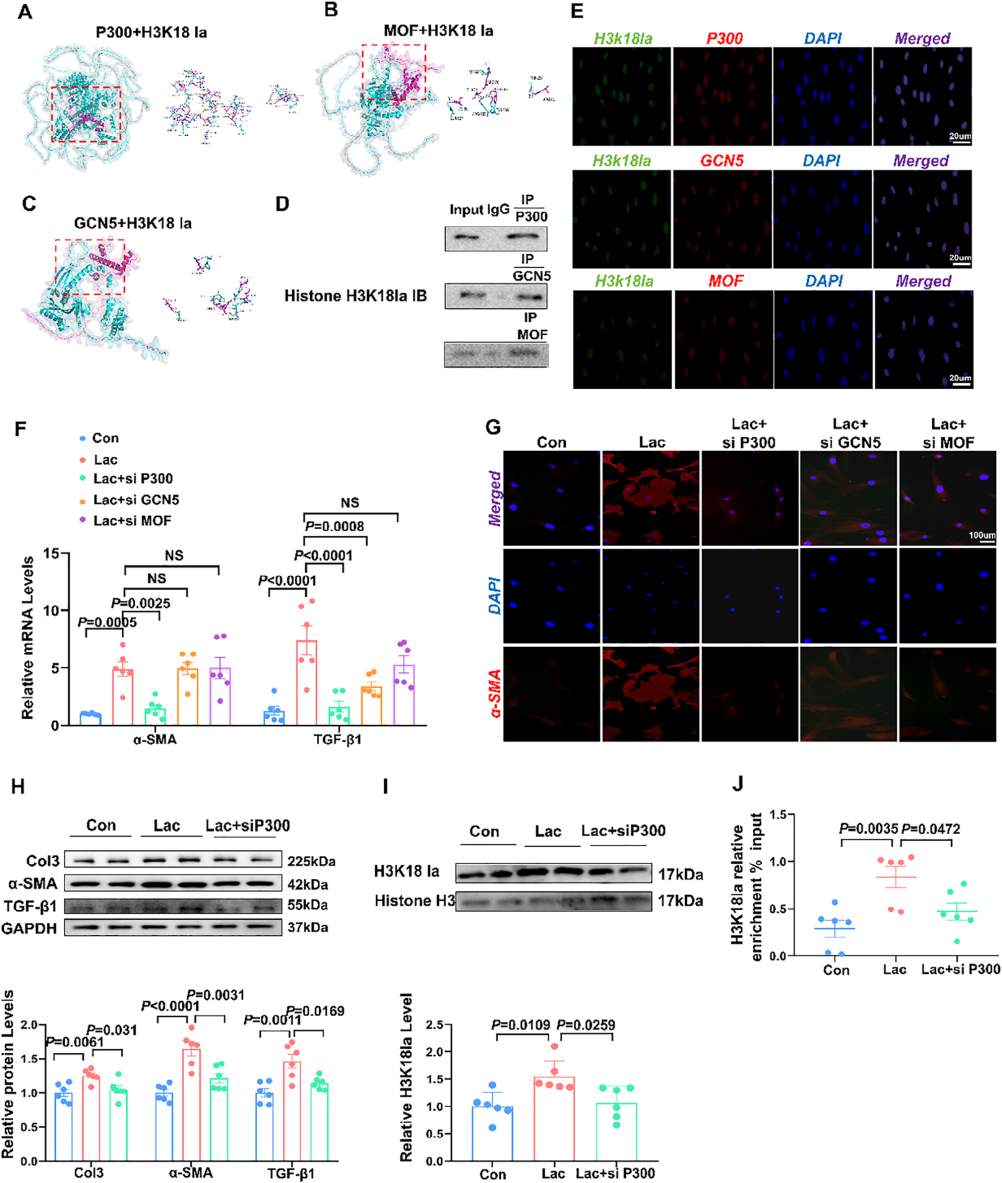
P300 serves as a writer for H3K18la to mediate TGF‐β1 transcription. A–C) ClusPro and ZDOCK 3.0 were performed to analyze the binding mode between the peptide and mucus P300(A)/MOF(B)/GCN5(C). D) Co‐immunoprecipitation (CoIP) analysis for H3K18la in the presence of P300, MOF, and GCN5 was conducted on cell lysates from CF treated with lactate. The lysates were immunoprecipitated using anti‐P300/MOF/GCN5 antibodies, followed by western blotting to assess H3K18la levels (n = 3). E) Immunofluorescence co‐staining for H3K18la (green) with P300/MOF/GCN5 (red) in CF treated with lactate. Scale bar = 20um. F) qPCR validation of the relative abundance of α‐SMA and TGF‐β1 in CF (n = 6). G) Representative images of α‐SMA staining of CF. Scale bar = 100 µm. H–I) Representative bands and quantification of the protein levels of Col3, α‐SMA, TGF‐β1, and H3K18la in CF, followed by lactate stimulation for 24 h (n = 6). J) Detection of H3K18la occupancy rates (n = 6). The data are given as mean ± SEM and compared by one way ANOVA (F, H, I, and J).

Histone acetyltransferase, including P300, MOF, and GCN5 (general control non‐depressible 5) has been reported as a potential writer protein for histone lactylation.^[^ ^26^ ^]^ To investigate the enzymatic process, we performed a molecular docking study to assess the binding modes between H3K18la and P300, MOF, and GCN5 (**Figure** **7**A–C). H3K18la was capable of interacting with these three transferases, which was confirmed by co‐immunoprecipitation (Co‐IP) (Figure 7D). Immunofluorescence staining further demonstrated that H3K18la and P300/MOF/GCN5 co‐localized within the nuclei of lactate‐treated fibroblasts (Figure 7E).

We further explored the role of acetyltransferase in regulating CF activation after incubating with lactate and alleviated the acetyltransferase effect through siRNA. Strikingly, P300 silencing markedly attenuated lactate‐induced CF activation, whereas siMOF and siGCN5 exhibited minimal effects (Figure 7F,G). Consistently, western blot analysis revealed a decrease in fibrosis‐related proteins in CF administered with siP300 relative to the lactate‐treated group (Figure 7H). We also assessed the regulatory effects of P300 on H3K18la enrichment and the expression level of the target gene TGF‐β1. The silencing of P300 prevented H3K18la enrichment and decreased TGF‐β1 expression after lactate treatment, indicating that P300 served as a “writer” for lactylation and mediated the regulation of target gene expression (Figure 7I,J). In conclusion, our results demonstrate that P300 mediates lactate‐driven upregulation of H3K18la and TGF‐β1expression in CF.

## 3 Discussion

Our study reveals glycolysis as a key contributor to AF and elucidates the mechanism through which lactate, a product of glycolysis, promotes AF development through histone lactylation. This provides a new perspective on the pathophysiology of AF. In the present study, we found that PFKM‐mediated activation of glycolysis promotes AF in mice mainly by inducing atrial fibrosis. Inhibiting glycolysis and atrial PFKM expression effectively mitigates atrial fibrosis and lowers the incidence of AF in CREM mice. Lactate, the terminal metabolite of glycolysis, promotes CF histone lactylation in the atrium, especially H3K18la. This modification of H3K18la activates CF by stimulating TGF‐β1 gene transcription, leading to atrial fibrosis and ultimately accelerating the development of AF. Additionally, we identified P300 as a key “writer” responsible for catalyzing histone lactylation. These findings suggest that targeting key glycolytic enzymes, lactate production, and histone‐modifying enzymes could represent novel therapeutic strategies for preventing AF. In conclusion, our study is the first to reveal the pathogenesis of AF through the lens of histone lactylation, highlighting the pivotal role of the PFKM/glycolysis/H3K18la/TGF‐β1 axis in promoting AF development.

A growing body of evidence supports the strong correlation between AF and metabolic reprogramming characterized by the Warburg effect.^[^ ^9^, ^27^, ^28^ ^]^ The Warburg effect, also known as aerobic glycolysis, prioritizes energy production through glycolysis rather than oxidative phosphorylation. This phenomenon is specifically reflected in upregulation of glycolytic enzymes and accumulation of their downstream metabolites, particularly lactate. In patients with AF, there is a notable increase in the expression of hexokinase, a critical enzyme in glycolysis, along with elevated levels of lactate in right atrial tissue.^[^ ^29^, ^30^ ^]^ Importantly, the up‐regulation of this glycolytic enzyme is positively correlated with markers of atrial structural remodeling. Studies in canine models of paroxysmal AF have demonstrated increased lactate dehydrogenase expression and subsequent lactate production in the atrial myocardium.^[^ ^31^ ^]^ Furthermore, sustained AF in sheep has been associated with enhanced glucose uptake in the atria and elevated lactate concentrations.^[^ ^32^ ^]^ Although glycolysis is recognized as essential for atrial energy demands in AF,^[^ ^27^ ^]^ its precise role in the development of AF is still unclear. Previous studies confirmed that glycolysis is considered associated with promoting various fibrosis diseases, including myocardial infarction‐induced fibrosis,^[^ ^14^, ^33^ ^]^ renal fibrosis,^[^ ^34^, ^35^ ^]^ pulmonary fibrosis,^[^ ^36^, ^37^, ^38^ ^]^ and liver fibrosis.^[^ ^39^, ^40^ ^]^ Our study revealed that enhanced glycolysis precedes both structural and electrical remodeling during the progression of AF. Of note, enhanced glycolysis markedly promoted atrial fibrosis and increased AF susceptibility, whereas inhibition of glycolysis effectively attenuated atrial structural remodeling and susceptibility to AF in mice. These findings underscore the critical biological role of glycolysis in fibrotic remodeling, which contributes to AF development. These findings corroborate previous studies demonstrating the effective reversal of fibrotic remodeling through targeting glycolytic suppression.^[^ ^34^, ^41^, ^42^, ^43^ ^]^ When the glycolysis route is suppressed by blocking the glycolytic enzymes, the pro‐fibrotic reaction is diminished. Our study identified PFKM as the pivotal enzyme driving glycolysis upregulation in CREM mice. Importantly, early intervention targeting PFKM significantly prevented atrial fibrosis and reduced AF susceptibility. Hence, our study highlights an important link between glycolysis and atrial fibrosis, indicating a previously unrecognized role of glycolysis in promoting AF.

Glycolysis‐derived lactate plays a critical role in cardiovascular diseases by adding lactoacyl groups to histone lysine (K) residues, which subsequently affects protein function and gene expression.^[^ ^44^, ^45^, ^46^ ^]^ Emerging evidence indicates that histone lactylation facilitates repair gene activation post‐myocardial infarction,^[^ ^47^ ^]^ participates in vascular calcification,^[^ ^15^ ^]^ and modulates heart failure progression.^[^ ^16^, ^48^ ^]^ However, its involvement in AF has not been fully explored. Our study revealed significant lactate accumulation in the atrium of AF patients and CREM mice, accompanied by increased histone lactylation particularly H3K18la in the atrium and atrial fibroblasts. As we know, histone lactylation regulates fibrosis‐related diseases through fibroblast activation.^[^ ^49^ ^]^ We further identified that H3K18la modification initiated TGF‐β1 transcription in CF, promoting CF activation and ultimately accelerating the progression of AF. TGF‐β1 gene is recognized as a central regulator of fibroblast activation in AF pathogenesis, with its inhibition demonstrating therapeutic potential for ameliorating atrial fibrosis and AF development.^[^ ^49^, ^50^ ^]^ Therefore, our study provides new insights into the metabolism‐epigenetic‐fibrosis axis in the context of AF. These findings contribute to our understanding of the molecular mechanisms underlying AF progression and highlight potential therapeutic targets for treating or preventing AF.

“Writer” plays a crucial regulatory role in epigenetic modifications. A “writer” enzyme crucially regulates epigenetic modifications by adding or importing specific modifications to substrate molecules, and its expression levels largely determine the rate of lactylation.^[^ ^33^, ^51^ ^]^ Currently, it has been observed that the acetyltransferases P300, GCN5, and MOF may function as histone lactylation writers.^[^ ^35^, ^36^ ^]^ By disrupting histone lactylation by silencing or inhibiting the writer, thereby suppressing the cellular proliferation and fibrosis capacity of CF under a high lactate environment. Remarkably, the ability of lactate‐mediated fibrosis was significantly eliminated upon inhibition of P300. These results demonstrate the significant catalytic effect of lactate‐dependent P300 on H3K18la and facilitation of TGF‐β1 transcription.

There are several limitations in our study. First, we did not assess the lactylation of non‐histone proteins. Furthermore, we were unable to establish a direct correlation between H3K18la and AF due to the inherent complexity and dynamic nature of histone modifications, which reduces the likelihood of point mutations in individual bases. Additionally, maintaining sterility during long‐term EF‐exposed cultures is inherently complex, as the open‐configuration imaging setup increases contamination risks. Besides, all in vivo experiments were indeed conducted using male animal models to maintain consistency in hormonal variables during our preliminary mechanistic exploration in the current study. Finally, the lack of direct comparative data between left and right atrial tissues for PFKM, H3K18la, and TGF‐β1 expression levels represents a critical limitation. Future research should incorporate mass spectrometry and site‐directed mutagenesis to elucidate the role of lactylation in regulating specific cellular proteins and their functions in multiple AF models.

In summary, our study uncovers a previously unrecognized importance of the PFKM‐mediated metabolome‐epigenome‐fibrosis signaling cascade in promoting AF. We found that enhanced glycolysis mediated CF histone lactylation upregulated TGF‐β1 expression, resulting in the development of AF. From an epigenetic perspective, we provided both a novel mechanistic insight and a potential therapeutic target for the prevention of AF.

## 4 Experimental Section

### Human and Animal Studies

The human study was approved by the Research Ethics Committees of the First Affiliated Hospital of Harbin Medical University (IRB‐AF/SC‐04/02.0). All patients provided written informed consent. All animal procedures in this study were conducted in strict accordance with the Health Guide for the Care and Use of Laboratory Animals and were approved by the Animal Care Committee at Harbin Medical University to ensure humane treatment (Ethical approval number: 2024012).

### Statistical Analysis

Statistical analyses were performed using GraphPad Prism 9.0 software (GraphPad Software, Inc., La Jolla, CA). The Shapiro‐Wilk test was employed to evaluate normality. Continuous variables were presented as mean ± standard error of the mean (SEM). Comparisons between the two groups were conducted using Student's unpaired *t*‐test. For variables with more than two groups, one‐way ANOVA was utilized, followed by Tukey's post hoc tests for further comparisons. Categorical variables were reported as counts and percentages and analyzed using either the Chi‐square test or Fisher's exact test. Statistical significance was established at *P* < 0.05.

## Conflict of Interest

The authors declare no conflict of interest.

## Author Contributions

N. F., N. Z., and X. J. contributed equally to this work. Y.G., N.F., and Y.Z. designed the research. S.Y., N.F., N.Z., Z.W., Q.G., L.M., X.L., S.Z., and C.J. performed the experiments. N.F. and Z.N. performed bioinformatic analyses. S.Y., Z.W., L.M., Q.G., and S.Z. assisted in mouse experiments. Q.G., N.Y., Z.N., and Y.Z. assisted in human experiments. S.Y., N.F., and N.Z. analyzed data. Y.G. and Z.Y. commented on the study and revised the paper. N.F. wrote the manuscript with input from all authors.

## Supporting information



Supporting Information

Supporting pdf

## Data Availability

The data that support the findings of this study are available from the corresponding author upon reasonable request.
