# Supplementary material for: PFKM‐Driven Lactate Overproduction Promotes Atrial Fibrillation via Triggering Cardiac Fibroblasts Histone Lactylation
Source: Adv Sci (Weinh). 2025 Jun 26;12(34):e00963. doi: 10.1002/advs.202500963 (PMC12442653; doi:10.1002/advs.202500963)
Supplement: Supplementary file 1 — Supporting Information [file ADVS-12-e00963-s001.docx]

**2. Methods:**

**2.1 Human studies**

Atrial samples, including SR and AF, were collected from patients undergoing mitral valve replacement under open chest vision at the Cardiac Surgery Department of the First Affiliated Hospital Medical Center of Harbin Medical University (**Figure S1**). Patients diagnosed with mitral stenosis were divided into sinus rhythm and atrial fibrillation. The atrial tissue specimens intraoperatively obtained from patients were immediately divided into two part for subsequent experimental analyses. One snap frozen in liquid nitrogen and stored at − 80°C until further analysis. Another portion of tissue was placed in sterile PBS to prepare atrial fibroblasts for extraction and further analysis. The baseline characteristics of the SR group (n = 11) and the AF group (n = 11) are shown in **Supplemental Table-S1**.

**2.2 Experimental Animals**

All animal procedures in this study were conducted in strict accordance with the Health Guide for the Care and Use of Laboratory Animals and were approved by the Animal Care Committee at Harbin Medical University to ensure humane treatment. CREM-IbDC-X (CREM) transgenic mice were on an FVB/N background, weighing between 20-24g and with a mean age of 8 weeks, were procured from Cyagen Biosciences Inc. (Suzhou.China)) ^[6]^. Twenty male C57BL/6 mice weighing 20-24g with a mean age of 8 weeks were obtained from Beijing Vital River Laboratory Animal Technology Co, Ltd (Beijing, China) and housed at the Experimental Animal Center of Harbin Medical University. Twenty New Zealand white rabbits (2.5−3.0 kg, male) were purchased from the Experimental Animal Center of the First Affiliated Hospital of Harbin Medical University (Harbin, China). The animals were randomly assigned to their conditions and individually housed, adhering to a 12:12-hour light-dark cycle. All mice were individually housed under specific pathogen-free (SPF) conditions.

**Experiment 1**

To create a PiggyBac transgenic line expressing CREM-IbΔC-X (CREM mice) under the control of alphaMHC-long promoter in FVB mouse. The alphaMHC-long promoter (Mouse α-cardiac myosin heavy chain promoter (5.4 kb)) is a tissue-specific promoter. In the PiggyBac vector, the “alphaMHC-long promoter-Kozak-HA tag-CREM-IbΔC-X-rBG pA” cassette will be flanked by two PiggyBac ITRs to facilitate transposes mediated transgene integration. The PiggyBac vector will be co-injected with transposes into fertilized eggs from FVB mice. The pups will be genotyped by PCR to identify the ones carrying the desired PiggyBac transgene. (Transgene PCR primer F1: TGACAGAGAAGCAGGCACTTTAC, R1:TGGCAAAGCAGCAGTAGGAGCT; Transgene PCR primer F2: CTTTGCCACAGGGAGTGGTGAT, R2: CTTTATTAGCCAGAAGTCAGATGC; Transgene PCR primer F3: GCTTGTCAATGCGGTAAGTGTCA, R3: CACCTAAAGGGCTGTTGCAAA; Transgene PCR primer F4: CTGCTGTCCATTCCTTATTCCATAG, R4: TTATCGGTCTGTATATCGAGG).

**Experiment 2**

The male three-month-old CREM mice were randomly assigned to two experimental groups, namely the control and knockout groups. The control group was administered a tail vein injection of placebo PBS (100 µl). Each knockout group mouse was then given 100 µl of AAV9 adeno-associated virus (5 × 10^11^ ug/ per mouse) via the same route. After injection, the general status of mice was monitored on a weekly basis until seven months of age, with routine indexes such as body weight, blood glucose and blood pressure. The group receiving a tail vein injection of placebo PBS will be designated the control group. The group receiving a tail vein injection of AAV9 adeno-associated virus will be referred to as the AAV-PFKM group. Electrophysiological tests were performed following a four-month period, concomitant with the acquisition of atrial tissue specimens for subsequent analytical investigations.

**Experiment 3**

Five-month-old male CREM mice were randomly divided into two experimental groups: control group and glycolysis inhibitor 2-DG injection group. The control group was given intraperitoneal injection of placebo PBS (100µl). The 2-DG group was intraperitoneally injected with 2-DG (0.5g/kg) twice a week. After injection, the general state of the mice was monitored weekly until the age of 7 months, including body weight, blood glucose, blood pressure and other routine indicators. The group receiving intraperitoneal injection of placebo PBS was designated as the control group. The group receiving intraperitoneal injection of 2-DG will be referred to as the 2-DG group. Electrophysiological tests were performed following a eight-week period, concomitant with the acquisition of atrial tissue specimens for subsequent analytical investigations.

**Experiment 3**

Eight-week-old male C57BL/6 mice were randomly divided into adeno-associated virus control (AAV-Con) group and adeno-associated virus overexpressing PFKM (AAV-OE-PFKM) group. In the AAV-control group, placebo PBS (100µl) was injected via the tail vein. Then, 100µl AAV9 adeno-associated virus (5 × 10^11^ ug/ per mouse) was injected in the same way in the adeno-associated virus over-expressing PFKM group. The general condition of mice was monitored weekly for 4 weeks after injection, including body weight, blood glucose, blood pressure and other routine indicators. The group receiving a tail vein injection of placebo PBS will be designated as the AAV-control group. The group receiving tail vein injection of AAV9 adeno-associated virus will be referred to as the AAV-OE-PFKM group. Electrophysiological tests were performed following a four-week period, concomitant with the acquisition of atrial tissue specimens for subsequent analytical investigations.

**Experiment 4**

The rabbit model of atrial fibrillation (AF) was established in accordance with our previous studies. Rabbits were anesthetized using a combination of ketamine (35 mg/kg; Sigma Aldrich, St. Louis, MO, USA) and xylazine (5 mg/kg; Sigma Aldrich). All procedures were conducted under sterile conditions following mechanical ventilation after a thoracotomy. A pacemaker (Harbin Polytechnic University, Harbin, China) was implanted into a subcutaneous pocket on the backs of the rabbits, with its electrode attached to the right atrium. Following surgery, the animals were allowed one week for recovery. Subsequently, the rabbits were randomly assigned into two groups (n = 6 per group): (1) sham-operated group (control group), where electrodes were sutured but no pacing occurred; (2) rapid atrial pacing group (RAP group), where electrodes were sutured and atrial rapid pacing was performed at 600 beats/min for 3 weeks.

**2.3 Electrophysiological study**

Atrial fibrillation (AF) was carried out in accordance with previously documented protocols^7,8^. Electrophysiological measurements were performed through jugular vein after intraperitoneal injection of 1% pentobarbital sodium (10 mg/kg).Then a 1.9-F octapolar electrophysiological catheter (Transonic Systems Inc., New York, USA) was put on the right atrium to deliver programmed stimulation using an automated stimulator interfaced with the data acquisition system (GY6000; HeNan HuaNan Medical Science & Technology Ltd).Induction of atrial fibrillation (AF) was achieved by stimulating at 15 V for 10 seconds, using atrial burst pacing set at twice the diastolic capture threshold (BCL: 20 ms; pulse width: 2 ms). This procedure was repeated ten times AF was deemed successfully induced if a period of rapid and fragmented atrial electrograms, characterized by irregular AV-nodal conduction and ventricular rhythm, persisted for at least 1 second. The duration of AF was defined as the interval between its initiation and spontaneous termination. Additionally, AF inducibility was assessed by calculating the number of AF episodes along with the total duration of AF divided by the total number of procedures conducted.

**2.4 Echocardiography**

Transthoracic echocardiographic studies were conducted using the Philips CX50 ultrasound system, which was equipped with a Philips S12-4 phased-sector ultrasound transducer, while the mice were under sedation. The following parameters were obtained using established protocols: left atrial (LA) diameter at end-diastole, interventional septum diastolic (IVSD), interventional septum systolic (IVSS), left ventricular internal dimension diastole (LVIDD), left ventricular internal dimension systole (LVIDS), left ventricular posterior wall in diastole (LVPWD), left ventricular posterior wall in systole (LVPWS), ejection fraction (EF) and fraction shorting (FS).

**2.5 Histological analysis and Masson's trichrome staining**

Fresh left atrial samples were fixed in 4% paraformaldehyde and embedded in paraffin after 48 hours, and cut into 4um slices continuously. Slices were prepared as follows: (1) deparaffinated: xylene I 10min, xylene II 10 min, 100% ethanol I 5 min, 95% ethanol II 5 min, 80% ethanol 5 min and distilled water 5 min; (2) performed hematoxylin and eosin (H&E) and Masson's trichrome staining according to the standard protocol; (3) dehydrated : 80% ethanol for 30 s, 95% ethanol for 1 min, 95% ethanol for 2 min, anhydrous ethanol for 3 min and anhydrous ethanol for 3 min; (4) transparentized : xylene for 5 min and xylene for 5 min; (5) sealed with neutral resin; The He staining images were analyzed with ImageScope x64 to evaluate the morphology of the heart. The fibrosis was quantified by software (Image-pro plus 6.0, Meida Cybernetics LP). Collagen volume fraction (CVF) was calculated as collagen area/total area × 100%.

**2.6 Immunohistochemical staining**

As mentioned above, the paraffin sections after dewaxing were stained by immunohistochemical staining. The antigen was repaired by high temperature. Incubated primary antibody: H3k18la, Pan kla and PFKM, then stayed overnight at 4 in the refrigerator. The next day, after the slices were restored to room temperature, the secondary antibodies were incubated for 1 hour according to the origin of the primary antibody. When DAB reagent was dyed, the tissue staining was observed under the microscope. After cleaning, the cells were stained with hematoxylin, then fixed and sealed. The antibody binding region is brownish yellow. The staining results were observed and captured by microscope (Zeiss, Jena, Germany). The H3K18la positive rate is determined by the ratio of brown number to nuclear number. The PFKM and global lactylation positive rate is determined by the ratio of brown area to total atrial area.

**2.7 Immunofluorescence**

Frozen left atrial tissue sections were washed with PBS and fixed with 4% paraformaldehyde for 30 min. After washing three more times with PBS, the cells were permeabilized with 0.1% Triton for 10 min and blocked with goat antiserum for 1 h. Primary antibodies included α-SMA, H3K18 la, and Pan Kla and were incubated overnight at 4° C. The next day, frozen sections with primary antibodies were returned to room temperature and secondary antibodies were incubated for 1 hour depending on the source of the antibody. After washing by PBS, DAPI was incubated in the dark for 5 minutes for nuclear staining. After washing with PBS, images were taken under a fluorescence microscope. Primary cardiac fibroblasts were seeded into confocal culture dishes. When the cell density reached 80%, cells were treated with lactate and subjected to fluorescent staining. The cell staining procedure was the same. Finally, the binding and expression of antibody were evaluated by fluorescence intensity. The staining results were observed and captured by immunofluorescence microscope (Zeiss, Jena, Germany).

**2.8 Isolation and culture of primary mice cardiomyocytes and fibroblasts**

**Experiment 1**

The primary cardiomyocytes and fibroblasts were isolated from Kunming mouse of 1-3 days as previously described^[9]^. The primary cells were extracted by trypsin digestion. The heart was cut into 1mm^3^ tissue and digested with 0.25% trypsin. The cell suspension digested by trypsin was mixed with DMEM containing 10% fetal bovine serum (FBS, Sciencell, San Diego, California, USA). After filtration, the isolated cells were centrifugated at 1200 rpm for 5 minutes. The cells were resuspended in DMEM with 10% FBS, 1% penicillin (100 IU/mL) and streptomycin (100 mg/mL) and inoculated in six-well plates. After 45 minutes, cardiac fibroblasts adhered to the wall, and the unattached cardiomyocytes were taken out and inoculated in six-well plates. Cells were cultured at the temperature of 37℃ and in the atmosphere of 5% CO2 and 95% O2. After 48h, the cardiomyocytes and fibroblasts that adhered onto the culture dish. When the cell density reached 80% , it was used for follow-up experiments. The extracted primary cells were used for subsequent experiments such as lactate treatment, CCK8, PCR, western blotting, cell transfection and immunofluorescence.

**Experiment 2**

A tissue block culture approach was used to isolate atrial fibroblasts. As previously mentioned, the intraoperative human atrial tissue was cleaned in a sterile 1×PBS petri dish. Tissue pieces were washed and maintained in fresh sterile PBS containing 5% v/v Penicillin-Streptomycin (PBS-PS) while they were cut into small pieces (<1 mm^3^). The washed fragments were transferred to another Petri dish, and the tissues were soaked in 0.25% trypsin digest solution and digested at room temperature for 3-5min. Repeat this step 2 times. The supernatant was centrifuged for 1 min at 350g to remove cardiomyocytes and debris. After the digestion was completed, several milliliters of FBS were added to terminate the digestion. Tissue blocks were seeded on the culture surface of a Petri dish with 200ul aspirator, after which the flask was placed in a carbon dioxide incubator at 37°C for 2-4h. The Petri dishes were supplemented with 3ml DMEM medium containing 10% fetal bovine serum (FBS), 1% penicillin (100 IU/mL), and streptomycin (100 mg/mL) and placed at the temperature of 37℃ and in the atmosphere of 5% CO2 and 95% O2 for 7 days until the fibroblasts could limb out. The isolation protocol employed for atrial fibroblasts derived from 7-month old CREM mice strictly mirrored that established for human atrial fibroblasts. When the cell density reached 80%, it was used for follow-up experiments such as western blotting.

**2.9 Cardiac fibroblasts treated with lactate**

When primary cardiac fibroblasts reached 70 to 80% confluence, they were treated with or without 10mM lactate (Sigma-Aldrich) at 37°C with 5% CO_2_ and 95% O_2_ for 24 hours for follow-up experiments such as western blotting.

**2.10 TUNEL staining**

Apoptotic cells were identified using the One Step TUNEL Apoptosis Assay Kit (Beyotime, C1089) in accordance with the manufacturer's instructions^10^. Simply put, the control group and lactate treated cells were added with 20μg/ml proteinase K (without DNase) and incubated at 37℃ for 30 min. After PBS washing, the cells were incubated with the TUNEL reaction mixture at 37 C for 1 hour. Next, 50ul Streptavidin-HRP solution and 100ul DAB working solution was added dropwise to each sample. Subsequently, each specimen received sequential dropwise administration of 50μL streptavidin-horseradish peroxidase (HRP) conjugate followed by 100 μL 3,3'-diaminobenzidine (DAB) chromogenic substrate. The enzymatic chromogenic reaction was subsequently initiated and maintained under ambient temperature conditions (25±2°C) for precisely 10 minutes. Staining results were observed and captured with immunofluorescence microscopy (Zeiss, Jena, Germany).

**2.11 CCK8 assay**

The CCK8 Cell Proliferation Assay (Beyotime, C0037) was employed to evaluate cellular viability. For CCK-8 assays, cardiomyocytes and fibroblasts were seeded into 96-well plates at the density of 1000 cells/well. After lactate treatments, 10ul CCK8 was added to the medium and incubated for 4h at 37 °C. Following aspiration of the culture medium, 100 μL of dimethyl sulfoxide (DMSO) was added to each well and incubated for 10 minutes at ambient temperature. Absorbance measurements were subsequently conducted at 450 nm using a microplate reader (Thermo Fisher Scientific, Waltham, MA, USA) within 1 hour of treatment. Statistical analyses were performed with GraphPad Prism 9.0 software. A threshold of *P* < 0.05 was defined as statistically significant for pairwise comparisons.

**2.12 Measurement of ROS in atrial tissues**

ROS production was assessed using dihydroethidium (DHE) as previously described^11^. Superoxide generation in cryosections of atrial tissues was evaluated with a ROS detection kit (Beyotime, S0033S). In brief, frozen sections were washed thrice with PBS and then incubated with 10% goat serum for 30 min at room temperature, and then were loaded with DHE at a concentration of 10μmol/L for 30 minutes at 37°C. Following the dye incubation, we conducted more than three washing steps to thoroughly remove any excess dye that had not penetrated the cells. After washing three times with PBS, the fluorescence intensity of ROS was measured using immunofluorescence microplates (Zeiss Jena) and analyzed utilizing ImageJ software.

**2.13 Cell transfection**

TGF-β1, P300, GCN5, and MOF siRNAs were synthesized by Gene Chem (China). When primary cardiac fibroblasts reached 70 to 80% confluence, according to the manufacturer's instructions, the plasmid vectors and siRNAs were transfected into cardiac fibroblasts by using Lipofectamine 2000 (ThermoFisher). After incubating the transfection reagent for 4 hours, the cell culture medium was changed to DMEM with 10% FBS, 1% penicillin (100 IU/mL) and streptomycin (100 mg/mL). After incubation for 24 hours, the cells were extracted for subsequent experiments.

**2.14 Protein extraction and western blot**

**Experiment 1 Total protein extraction**

After the left atrial tissues and cells were cleaned by PBS twice, added protease lysate (Beyotime, P0013B) (the ratio of tissue to protease lysate was 1:5, and 80ul of protease lysate was added per six-well plate according to the amount of cells) , mixed well and grinded into homogenate. After being frozen for 30 minutes, the supernatant was collected by centrifugation at the speed of 13500rpm for 15 minutes and added protein loading buffer with a volume of 1/4 of the supernatant.

**Experiment 2 Histone protein extraction**

Based on the kit protocol, tissues and cells mixed with 1X Presplitting Buffer (the ratio of tissue to Presplitting Buffer was 1:6, and 120ul of protease lysate was added per six-well plate according to the amount of cells) and rest for 10 minutes on ice. After being centrifuged for 1 minute, drawed the supernatant and mixed 200ul Lysis Buffer. After staying overnight at 4℃, the supernatant was sucked and DTT was added to adjust the PH to 7. Finally, the total liquid was added protein loading buffer and denatured the protein at 100°C for 10 minutes.

**Experiment 3 Co‐immunoprecipitation**

In accordance with the manufacturer's protocol, co-precipitation (Co-IP) was conducted using the immune co-precipitation kit (Beyotime Biotechnology, P2197 M). Following treatment with lactate, cells were harvested with immunoprecipitation (IP) buffer on ice. After centrifugation at 12,000 rpm for 15 minutes, the supernatant was harvested. For pre-clearing, 500 μg of total protein supernatant was incubated with 20 μL Protein A/G agarose beads (Santa Cruz Biotechnology, Dallas, TX, USA) for 1 hour at 4°C with gentle rotation. After bead removal by centrifugation, the pre-cleared lysate was incubated overnight at 4°C with 2 ug of target-specific primary antibody or isotype-matched control antibody (Cell Signaling Technology, Danvers, MA, USA). Antigen-antibody complexes were captured by adding 30 uL fresh Protein A/G beads and incubating for 2 hours at 4°C. Bound proteins were eluted in 2× Laemmli sample buffer (Bio-Rad Laboratories, Hercules, CA, USA) by boiling at 95°C for 10 minutes. Eluates were resolved by SDS-PAGE and subjected to immunoblotting analysis.

Different concentrations of gels (8%-12% SDS-PAGE) were seperated by proteins according to different molecular weights. The total protein were adjusted to the same concentration、electrophoned, transferred onto PVDF membranes, blocked with 5% non-fat milk for 1 hour at room temperature, and then incubated with primary antibodies at the temperature of 4℃ overnight. Antibodies against collagen III, α-SMA, TGF-β1, Bcl2, and Bax were obtained from Abcam Biotechnology Company (Cambridge, UK), while antibodies against HK, PFKM, LDHA, GLUT1, PKM were obtained from Cell Signaling Technology Inc. (Bioss, MA, USA). Pan Kla and H3K18 lactylation were obtained from PTM-biolab. The secondary antibodies including anti-HRP Goat Anti-Mouse IgG(H+L) (ZSGB-Bio, cat#ZB-2305, RRID: AB_2747415), HRP Goat Anti-Rabbit IgG(H+L) (ZSGB-Bio, cat#ZB-2301, RRID: AB_2747412). Finally, the bands were detected by ECL kit、captured by ChemiDoc XRS gel documentation system (Bio-Rad, Hercules, CA, USA) and analyzed by ImageJ software for gel image analysis.

**2.15 Quantitative reverse transcription polymerase chain reaction (qRT-PCR)**

The extraction of RNA from tissues and cells was conducted using the AxyPrep Multisource Total RNA Miniprep Kit, in accordance with the provided instructions. The concentration of RNA was measured and reverse transcription carried out in accordance with the corresponding ratio using a Yeasen RT Kit. Following the conversion of total RNA into cDNA, 0.5 µL of pre-primer, 0.5 µL of post-primer, and 10 µL of SYBR Green supermix were added, respectively, to create a 20 µL system. GAPDH was employed as the internal reference for fibrosis-related indexes. Gene expression levels were determined using the 2-ΔΔCT method. The primers are listed in the additional file.

**2.16** **Enzyme-Linked Immunosorbent Assay**

The content of lactate in left atrial tissue was detected by ELISA kit (Solarbio, BC2235). At the same time, the concentrations of LDH, HK, PFKM and PKM in atrial tissue were determined by ELISA kits (Jiangsu Meimian Industrial Co., Ltd). All measurements were performed according to the instructions of the corresponding kits provided by the manufacturer.

**2.17 Tail Biopsy Genotyping**

We used TaKaRa MiniBEST Universal Genomic DNA Extraction kit (Ver.5.0_Code No. 9765) to gain high purity of genomic DNA. After the mice tail were pretreated, Add 180 μL of Buffer GL, 20 μL of Proteinase K and 10 μL of RNase A per tail piece (2-5 mm) in a microcentrifuge tube. Be careful not to cut too much tail. Incubate the tube at 56 ℃ overnight. Spin in microcentrifuge at 12,000 rpm for 2 minutes to remove impurities. Add 200 μL Buffer GB and 200 μL absolute ethyl alcohol with sufficient mixing. Place the spin Column in a collection tube. Apply the sample to the spin and centrifuge at 12,000 rpm for 2 min. Discard flow-through. Add 500 μL Buffer WA to the spin column and centrifuge at 12,000 rpm for 1 min. Discard flow-through. Add 700 μL Buffer WB to the spin column and centrifuge at 12,000 rpm for 1 min. Discard flow-through. Place the spin Column in a new 1.5ml tube. Add 50~200 μL sterilized water or elution buffer to the center of the column membrane and let the column stand 5min. Quantify to genomic DNA. Eluted genomic DNA can be quantified by electrophoresis.

**2.18 ChIP-qPCR assay**

The extracted primary fibroblasts (1x10⁶) were inoculated on a 6-well plate and treated with lactate (10mM) and si P300 for a period of 24 hours. The cells were cross-linked with 1% formaldehyde for 10 minutes, after which the cross-linking was quenched with 0.125 ml of 0.1 M Tris-HCl, pH 7.5, at room temperature for 5 minutes. Subsequently, the cleaned battery was washed on three occasions with cold PBS and collected. The cells were lysed on ice in lysis buffer (50 mM Tris-HCl pH 8.0, 10 mM EDTA pH 8.0, 1% sodium dodecyl sulfate) for 10 minutes. Subsequently, the Missoni Ultrasonic Instrument 3000 (9W; 20 seconds to open; 40 seconds off) was employed to confirm the average fragment size, which was observed to be 200-500 bp. The total length agarose gel electrophoresis was conducted for this purpose. Immunoprecipitation was conducted using anti-H3K18la (PTM-1427rm; 2 mg) and anti-IgG (3900s; 2 mg) antibodies. The quantity of DNA fragments precipitated, which contained the TGF-β1 promoter, was determined by qPCR using primers described in the Resource Table.

**2.19 Cut&Tag**

The genes regulated by histones were identified using Cut Tag technology. First, concanavalin A-coated magnetic beads bind to cells due to their ability to attach to glycoproteins on cell membranes or nuclei. Next, digitalis saponin (a nonionic detergent) makes the cell membrane permeable, allowing a primary antibody specific to the target protein to enter and incubate with it. A secondary antibody is then added for enhanced binding. Protein A/G-Tn5 (pA/G-Tn5) is introduced for incubation. Tn5 is brought near the target protein through Protein A and antibody interaction. Mg2+ addition cuts sequences close to the target protein, and separating it from chromatin and causing dissociation outside the cell. The DNA is extracted; fragments below 700 bp are sorted using magnetic beads, followed by PCR amplification to create a database for high-throughput sequencing. Finally, the sequencing library is sequenced on the NovaSeq 6000 platform (Illumina) at Shanghai Personal Biotechnology Co., Ltd.

**2.20 RNA‐sequencing**

Total RNA was extracted using Trizol Reagent (Invitrogen Life Technologies), and its concentration, quality, and integrity were assessed with a NanoDrop spectrophotometer (Thermo Scientific). Three micrograms of RNA served as the input for sample preparations. Sequencing libraries were generated through the following steps: mRNA was purified from total RNA using poly-T oligo-attached magnetic beads. Fragmentation occurred with divalent cations at elevated temperatures in an Illumina buffer. First-strand cDNA was synthesized using random oligonucleotides and Super Script II reverse transcriptase, followed by second-strand synthesis with DNA polymerase I and RNase H. Overhangs were converted to blunt ends via exonuclease/polymerase activities, after which enzymes were removed.After adenylating the 3′ ends of DNA fragments, Illumina PE adapter oligonucleotides were ligated for hybridization. To select cDNA fragments of 400–500 bp, library fragments were purified using AMPure XP system (Beckman Coulter). Fragments with ligated adapters on both ends underwent selective enrichment via a 15-cycle PCR reaction using Illumina PCR Primer Cocktail. The products were then purified again with AMPure XP system and quantified using Agilent high-sensitivity DNA assay on Bioanalyzer 2100 (Agilent). Finally, the sequencing library was sequenced on NovaSeq 6000 platform (Illumina) at Shanghai Personal Biotechnology Cp. Ltd.

**2.21 GEO Database analysis**

The GSE128188 dataset from the Gene Expression Omnibus (GEO) database (https://www.ncbi.nlm.nih.gov/geo/) was screened, and RNA data were downloaded for analysis. Ten samples were selected: five patients in sinus rhythm and five in atrial fibrillation. The dataset was generated using the Illumina NextSeq 500 on the GPL18573 platform (PMID: 31172864). The EdgeR package was used to normalize data and calculate fold change and p-values between groups. The R package ClusterProfiler performed Gene Ontology (GO) (http://geneontology.org/) and Kyoto Encyclopedia of Genes and Genomes (KEGG) pathway enrichment analysis on differentially expressed genes (DEGs). Statistical significance was defined as two-tailed *P* < 0.05. All analyses adhered to these pre-specified cutoffs without post hoc threshold adjustments.

**2.22 Statistical analysis**

Statistical analyses were performed using GraphPad Prism 9.0 software (GraphPad Software, Inc., La Jolla, CA). The Shapiro-Wilk test was employed to evaluate normality. Continuous variables are presented as mean ± standard error of the mean (SEM). Comparisons between two groups were conducted using Student's unpaired t-test. For variables with more than two groups, one-way ANOVA was utilized followed by Tukey's post hoc tests for further comparisons. Categorical variables are reported as counts and percentages and analyzed using either the Chi-square test or Fisher's exact test. Statistical significance was established at *P* < 0.05.

**Supplemental Figure Legends**

**Table-S1. Clinical characteristics of atrial samples donors for atrial lactate detection.**

| Characteristics | SR (n=11) | AF (n=11) | *P value* |
| --- | --- | --- | --- |
| Age, y | 62.82±4.18 | 61.91±6.91 | 0.594 |
| Male, n (%) | 5 (45.4) | 6(54.6) | ＞0.999 |
| Body mass index*, kg/m2 | 21.66±2.27 | 21.24±2.01 | 0.550 |
| Current smoker, n (%) | 4 (36.3) | 4 (36.3) | ＞0.999 |
| Presence of diabetes, n (%) | 3 (27.2) | 4 (36.3) | ＞0.999 |
| SBP (mmHg) | 119.1±12.91 | 123. 3±25.96 | 0.387 |
| DBP (mmHg) | 76.55±9.11 | 74.48±11.48 | 0.541 |
| Total cholesterol, mmol/L | 4.95±0.27 | 4.87±0.49 | 0.580 |
| Triglycerides, mmol/L | 1.50±0.13 | 1.46±0.17 | 0.450 |
| HDL-C（mmol/L） | 1.09±0.17 | 1.14±0.23 | 0.279 |
| LDL-C (mmol/L) | 3.12±0.21 | 3.20±0.32 | 0.230 |
| Glucose (fasting, mmol/L) | 6.84±2.14 | 6.96±2.26 | 0.711 |

SR = sinus rhythm; AF = atrial fibrillation; SBP = Systolic blood pressure; DBP = Diastolic blood pressure; HDL-C = high-density lipoprotein cholesterol; LDL-C = low-density lipoprotein cholesterol.

**Table-S2. Clinical features of atrial samples donors for AF patients.**

| Characteristics | Paroxysmal AF  ( n = 7 ) | Perstistent AF  ( n = 4 ) | *P value* |
| --- | --- | --- | --- |
| AF duration (years) | 5.71±4.71 | 9±8 | 0.775 |
| Resting Heart Rate | 81.71±29.71 | 106.75±20.75 | 0.079 |
| Exercise Heart Rate | 110.72±51.28 | 135.25±32.25 | 0.1231 |
| Therapy, n (%) | 5 (71.4) | 4 (100) | 0.4909 |
| Anticoagulation Therapy (Warfarin), n (%) | 5 (71.4) | 4 (100) | 0.4909 |
| Rate Control (Amiodarone), n (%) | 3 (42.9) | 4 (100) | 0.1939 |


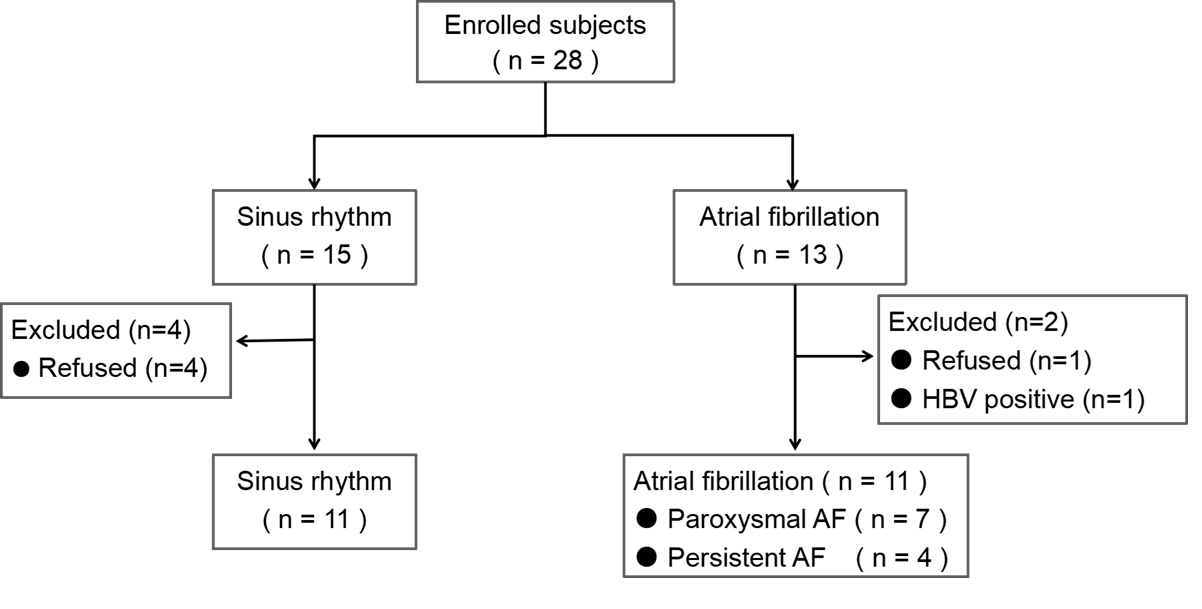


**Figure S1. Participant** **flowchart displaying the study design of lactate detection test.**

This test was carried out in patients hospitalized in the First Affiliated Hospital of Harbin Medical University. 28 individuals were enrolled to collect atrial samples. After excluding people who were refused, or having HBV positive, sinus rhythm (n = 11), and AF patients (n = 11) were included. There no subjects failed during the process of the study.


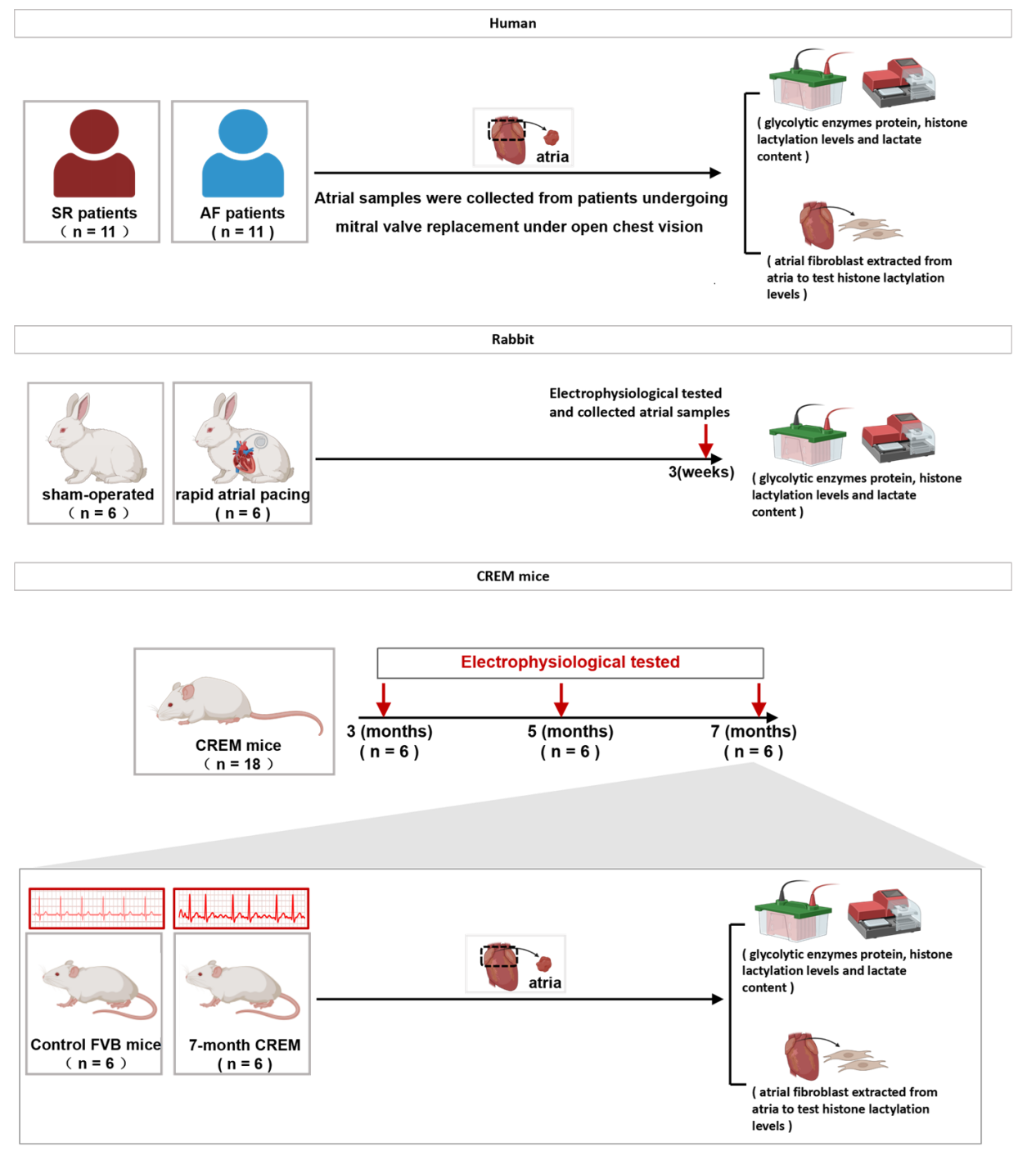


**Figure S2:** **Schematic depicting validation of atrial glycolysis-related enzymes across atrial fibrillation models including AF patients, rapid pacing rabbit and spontaneous AF model.**

Atrial tissue samples were collected from patients with postoperative atrial fibrillation (AF) and patients in sinus rhythm (SR). These samples were divided into two portions: one portion was used to quantify glycolytic activity, lactate levels, and histone lactylation content; the other portion was used to isolate primary atrial fibroblasts for subsequent validation studies. Rabbits were randomly divided into two groups: a sham-operated control group and a rapid atrial pacing group. After 3 weeks of pacing, electrophysiological measurements were performed, followed by atrial tissue collection for analysis of glycolytic activity, lactate levels, and histone lactylation. Longitudinal monitoring of atrial fibrillation substrate markers was performed in a mouse model of spontaneous AF (CREM mice). A significant increase in AF incidence was observed at 7 months of age. Atrial tissue from 7-month CREM mice and age-matched control mice was subsequently harvested for quantification of glycolytic activity, lactate levels, and histone lactylation content. The diagram was created in https://BioRender.com.


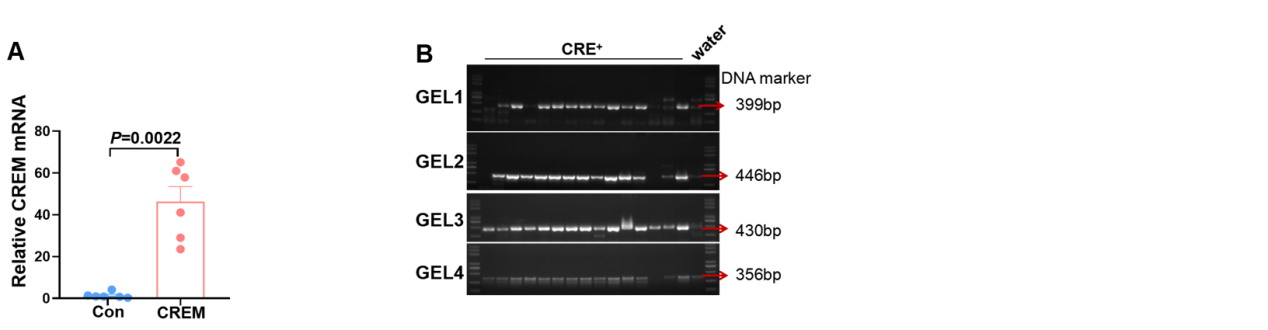


**Figure S3:** **Verification of successful modeling of spontaneous atrial fibrillation mice.**

**(A)** qPCR validation of the relative expression of CREM in control and CREM mice (n = 6).

**(B)** Agarose gel electrophoresis analysis of CREM gene. The pups were screened by the following PCR assay. Out of those pups screened, 4 positive gels were identified as successful CREM mice model , which were then confirmed by the same PCR with recut samples. (Transgene PCR primer F1:TGACAGAGAAGCAGGCACTTTAC, R1:TGGCAAAGCAGCAGTAGGAGCT; Transgene PCR primer F2: CTTTGCCACAGGGAGTGGTGAT, R2: CTTTATTAGCCAGAAGTCAGATGC; Transgene PCR primer F3: GCTTGTCAATGCGGTAAGTGTCA, R3: CACCTAAAGGGCTGTTGCAAA; Transgene PCR primer F4: CTGCTGTCCATTCCTTATTCCATAG, R4: TTATCGGTCTGTATATCGAGG)

The data are given as mean ± SEM and compared by Student’s *t* test.


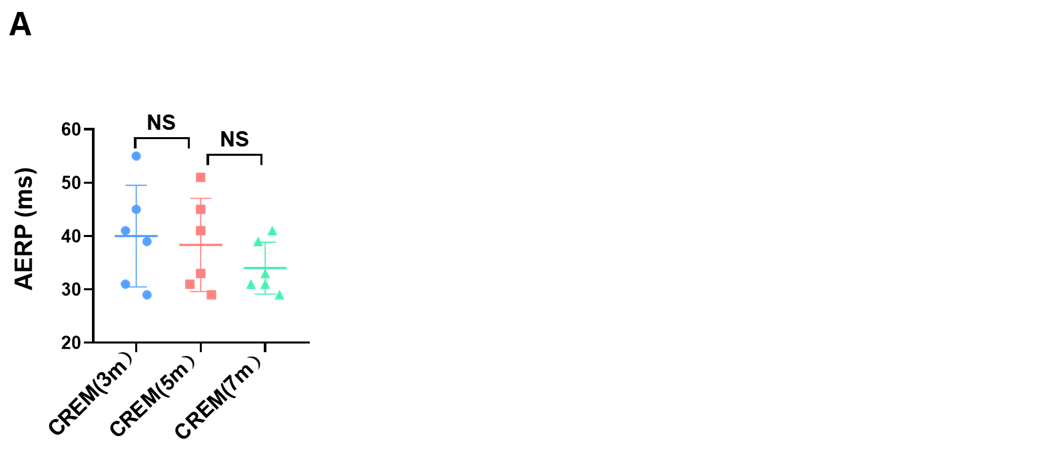


**Figure S4:** **Atrial fibrillation susceptibility test was performed at different ages of CREM mice.**

1. AERP in CREM mice from 3-months, 5-months and 7-months (n = 6).

AERP = Atrial Effective Refractory Period.

The data are given as mean ± SEM and compared by one-way ANOVA .


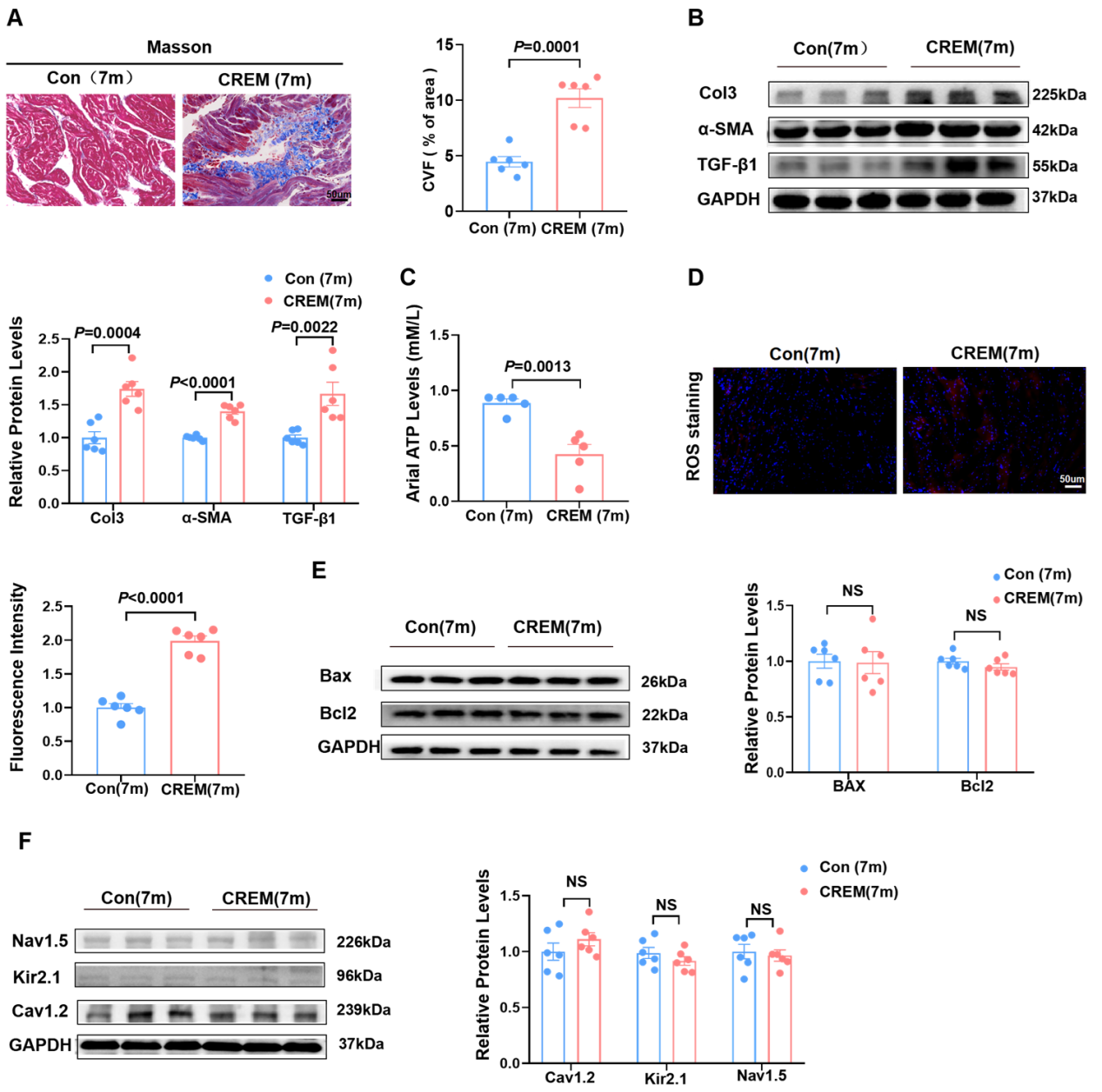


**Figure S5:** **7-month-old CREM mice were accompanied by increased atrial fibrosis and oxidative stress, but apoptosis and ion channel levels did not change significantly.**

1. Representative images and collagen volume fraction of atrial Masson’s staining of 7-month mice (n = 6). Scale bar = 50um.
2. Representative bands and quantification of the protein levels of Col3, α-SMA and TGF-β1 in atrial tissue with control group and 7-month group (n = 6).
3. The atrial concentration of ATP production in the control and CREM group (n = 5).
4. Representative images of ROS staining in atria with control mice and 7-month CREM mice. Scale bar = 50um.
5. Representative bands and quantification of the protein levels of Bax and Bcl2 in atrial tissue with control group and 7-month CREM group (n = 6).
6. Representative bands and quantification of the protein levels of Nav1.5、Kir2.1 and Cav1.2 in atrial tissue with control group and 7-month group (n = 6).

The data are given as mean ± SEM and compared by Student’s [*t* test](https://www.sciencedirect.com/topics/medicine-and-dentistry/student-t-test)**（A, B, C, D, E and F)**.


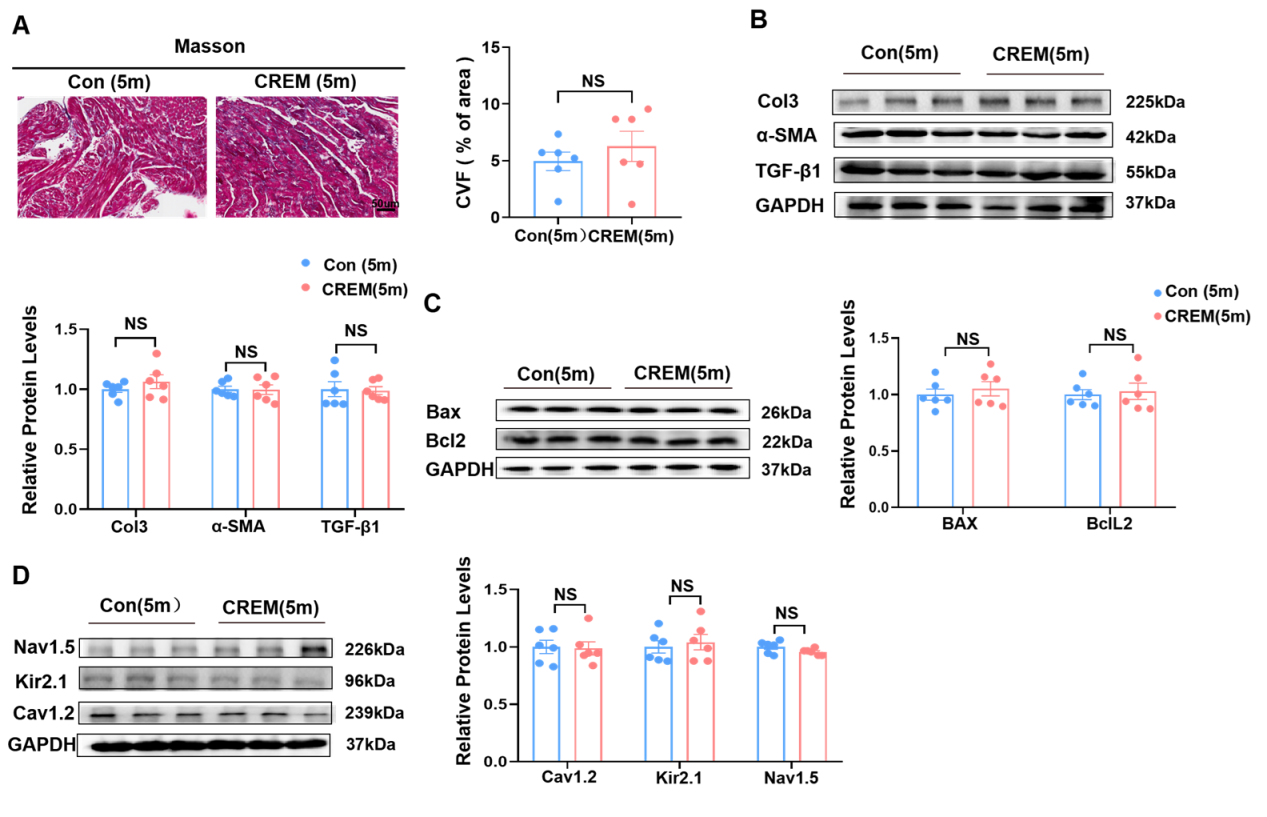


**Figure S6:** **Expression of electrical remodeling and structural remodeling in 5-month CREM mice.**

1. Representative images and collagen volume fraction of atrial Masson’s staining of 5-month mice (n = 6). Scale bar = 50um.
2. Representative bands and quantification of the protein levels of Col3, α-SMA and TGF-β1 in atrial tissue with control group and 5-month CREM group (n = 6).
3. Representative bands and quantification of the protein levels of Bax and Bcl2 in atrial tissue with control group and 5-month group (n = 6).
4. Representative bands and quantification of the protein levels of Nav1.5, Kir2.1 and Cav1.2 in atrial tissue with control group and 5-month group (n = 6).

The data are given as mean ± SEM and compared by Student’s [*t* test](https://www.sciencedirect.com/topics/medicine-and-dentistry/student-t-test) **(A, B, C and D)**.


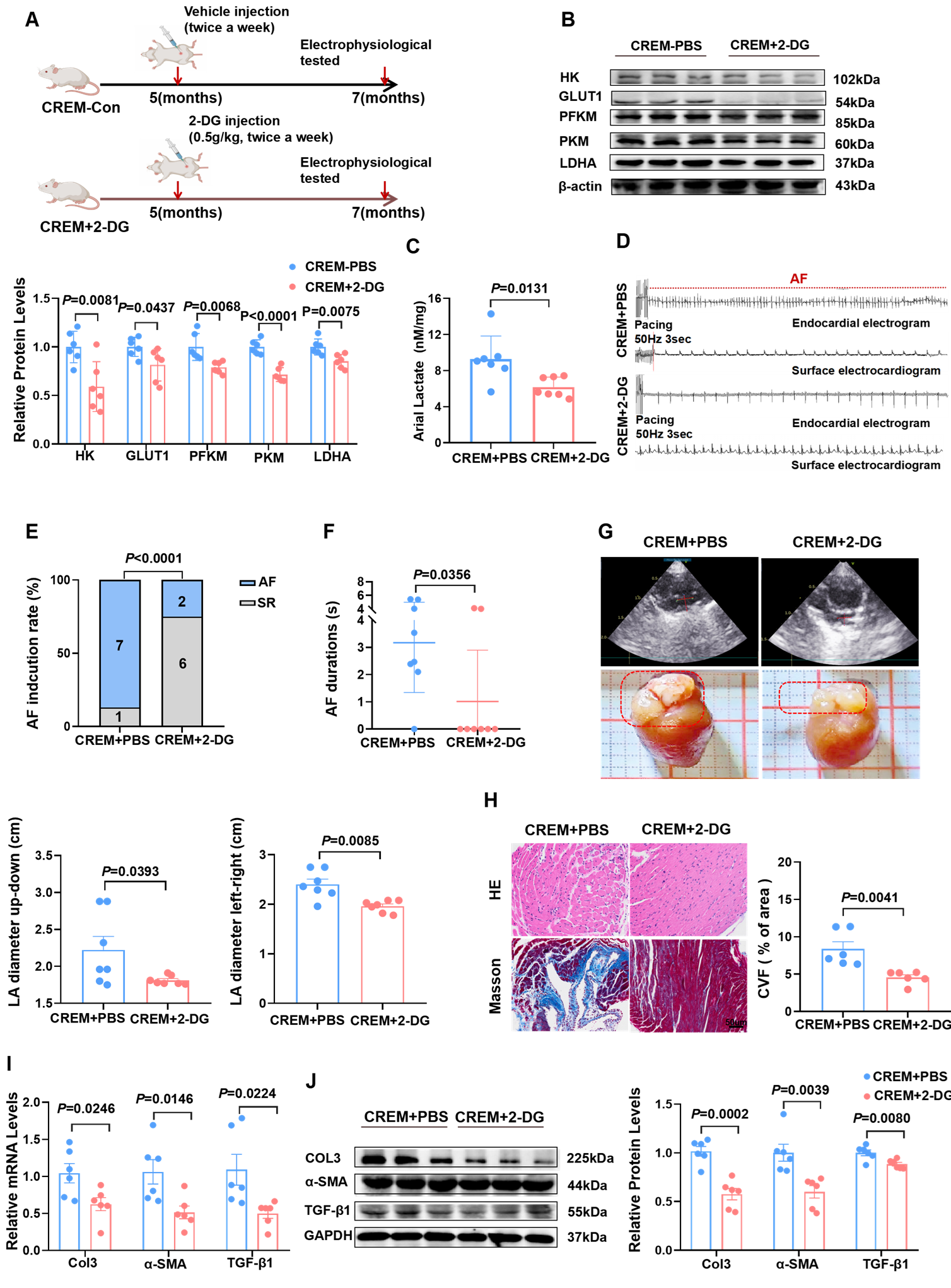


**Figure S7: Reduction of glycolysis with 2-DG would ameliorate AF risk in CREM mice.**

1. Schematic diagram showing the establishment of 2-DG and PBS model in CREM mice.
2. Representative bands and quantification of the protein levels of HK, GLUT1, PFKM, PKM and LDHA in atrial tissue with two groups (n = 6).
3. The atrial concentration of lactate (n = 7).
4. Representative examples of AF induction attempt in the CREM-PBS and CREM+2-DG group.

**(E-F)** AF inducibility and AF duration (s) (n = 8).

**(G)** Representative echocardiography and images of LA dimensions in the two groups (n = 7).

**(H)** Representative HE and atrial Masson staining of mice images (n = 6). Scale bar = 50 um.

**(I)** qPCR validation of relative abundance of Col3, α-SMA and TGF-β1 in two groups (n = 6).

**(J)** Representative bands and quantification of the atrial protein levels of Col3, α-SMA and TGF-β1 in two groups (n = 6).

The data are given as mean ± SEM and compared by Student’s *t* test (**B, C, G, H, I and J)** or Wilcoxon test **(F)** AF inducibility **(E)**was compared by Fisher exact test. The diagram was created in https://BioRender.com.


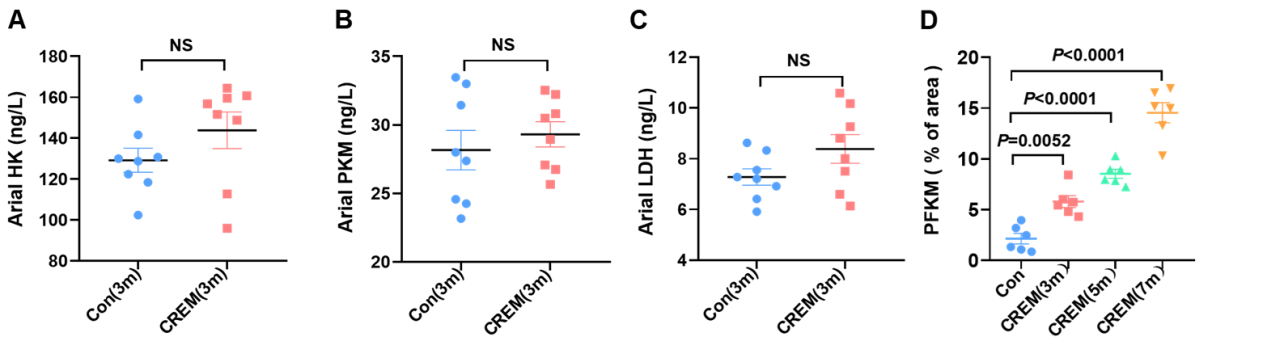


**Figure S8:** **Contents of glycolysis-related enzymes in atrial tissue of CREM mice at different months of age.**

**(A)** The atrial HK concentrations in mice from Control group and CREM group with 3 months of age (n = 8).

**(B)** The atrial PKM concentrations in mice from Control group and CREM group with 3 months of age (n = 8).

**(C)** The atrial LDH concentrations in mice from Control group and CREM group with 3 months of age (n = 8).

**(D)** Statistical map of PFKM immunohistochemical staining of atrial tissue in control group, 3, 5, and 7 months of age (n = 6).

The data are given as mean ± SEM and compared by Student’s t test**（A, B and C）**and one-way ANOVA **(D)**.


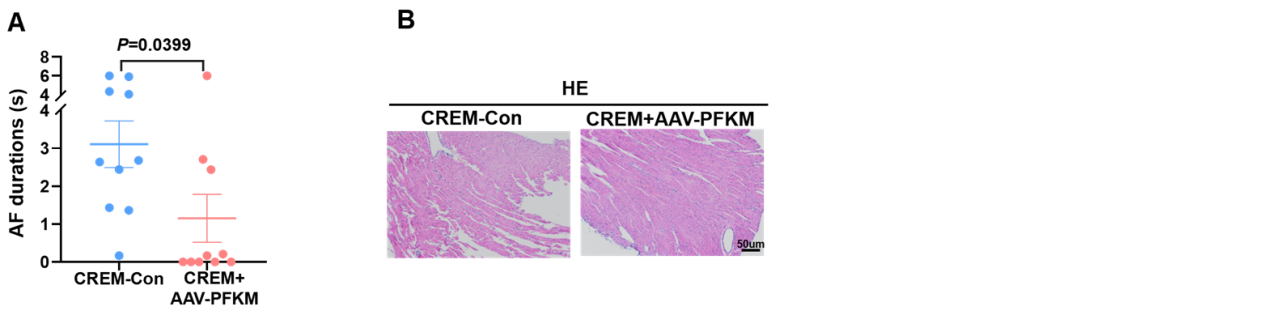


**Figure S9:** **Inhibition of PFKM alleviates AF susceptibility and improve myocardial fibers.**

**(A)** AF duration in mice from CREM with PBS and AAV-PFKM (n = 10).

**(B)** Representative images of atrial HE staining of two groups. Scale bar = 50um.

The data are given as mean ± SEM and compared by Wilcoxon test .


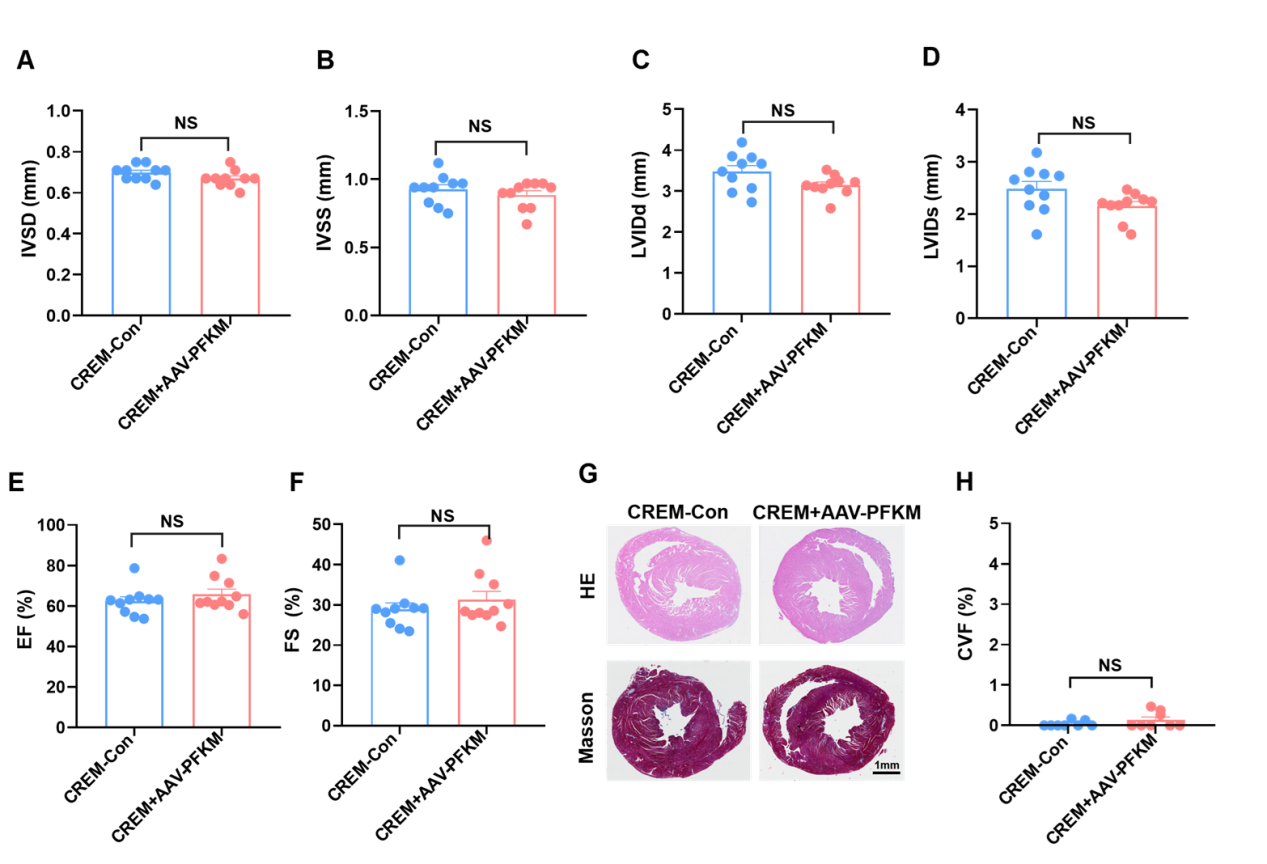


**Figure S10:** **Reduction of PFKM expression had no effect on the ventricles volume and function in CREM mice.**

**(A-F)** IVSD、IVSS、LVIDd、LVIDs、EF and FS in the two groups (n = 10).

**(G)** Representative images of atrial HE and Masson staining of two groups. Scale bar = 1 mm.

(**H**)The collagen volume fraction in ventricle. (n = 8).

IVSD = Interventricular septum diastolic; IVSS = interventricular septum systolic; LVIDd = left ventricular internal dimension diastole; LVIDs = left ventricular internal dimension systole; EF= ejection fraction; FS = fraction shortening.

The data are given as mean ± SEM and compared by Student’s *t* test.


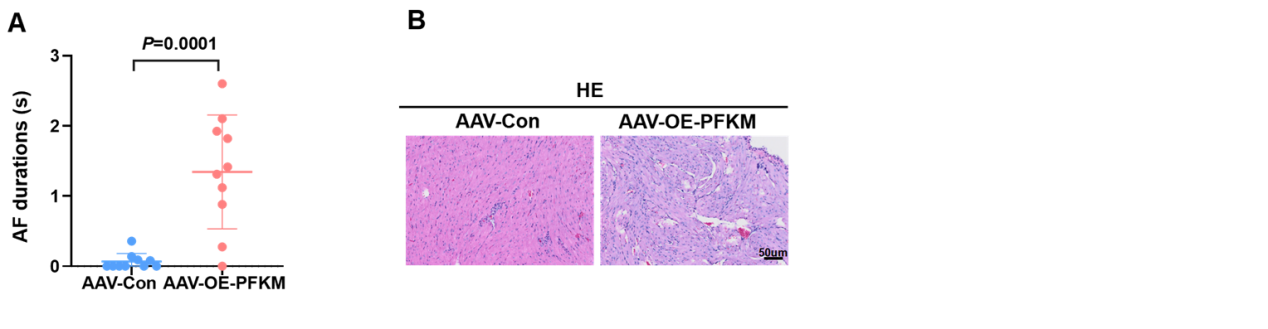


**Figure S11:** **Overexpression of PFKM increases AF susceptibility and displayed disordered myocardial fibers.**

**(A)** AF durations in mice from CREM with AAV-Con and AAV-OE-PFKM (n = 10).

**(B)** Representative images of left atrial HE staining of two groups. Scale bar = 50um.

The data are given as mean ± SEM and compared by Student’s *t* test.


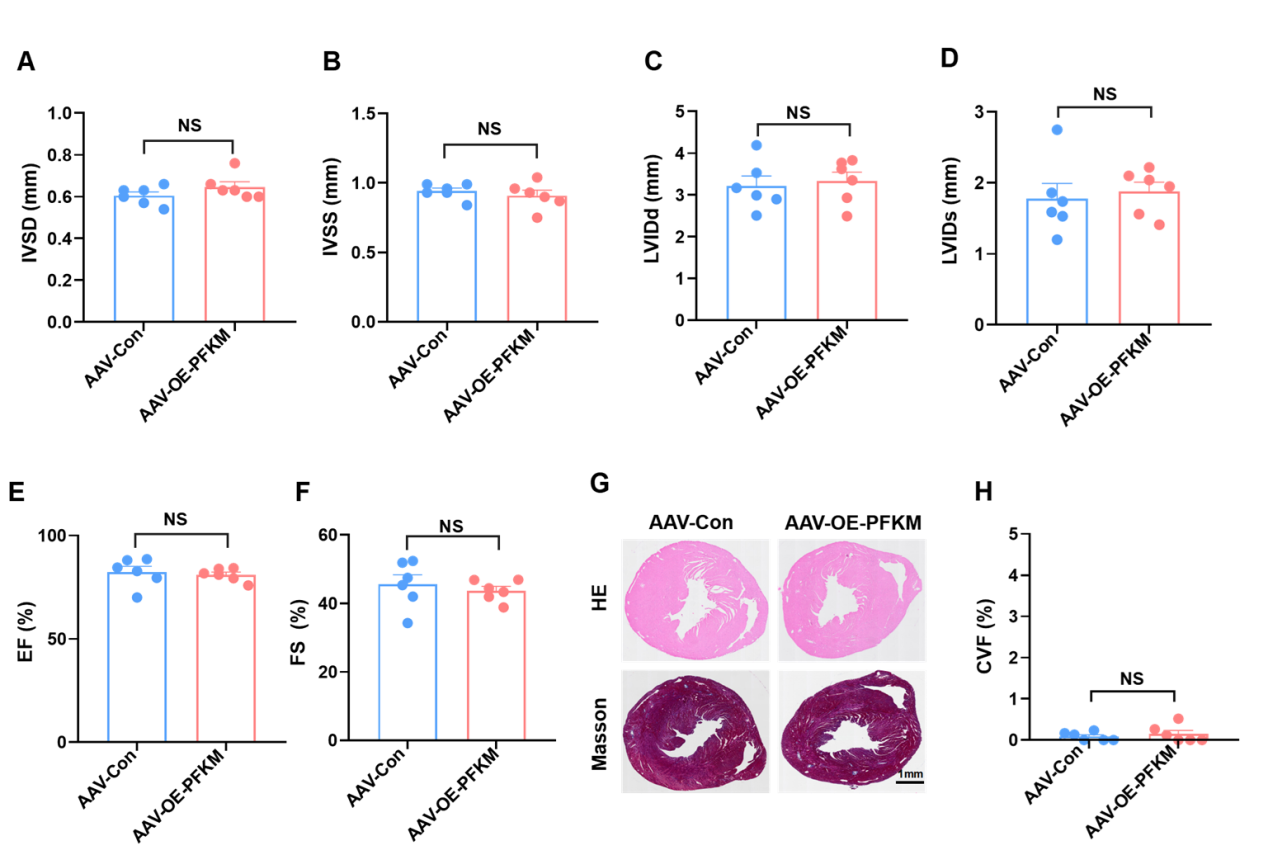


**Figure S12:** **Augmentation of atrial PFKM expression had no effect on the ventricles volume and function in mice.**

**(A-F)** IVSD、IVSS、LVIDd、LVIDs、EF and FS in the two groups (n = 6)

**(G)** Representative images of atrial HE and Masson staining of two groups. Scale bar = 1mm.

(**H**) The collagen volume fraction in ventricles (n = 6).

IVSD = Interventricular septum diastolic; IVSS = interventricular septum systolic; LVIDd = left ventricular internal dimension diastole; LVIDs = left ventricular internal dimension systole; EF= ejection fraction; FS = fraction shortening.

The data are given as mean ± SEM and compared by Student’s *t* test.


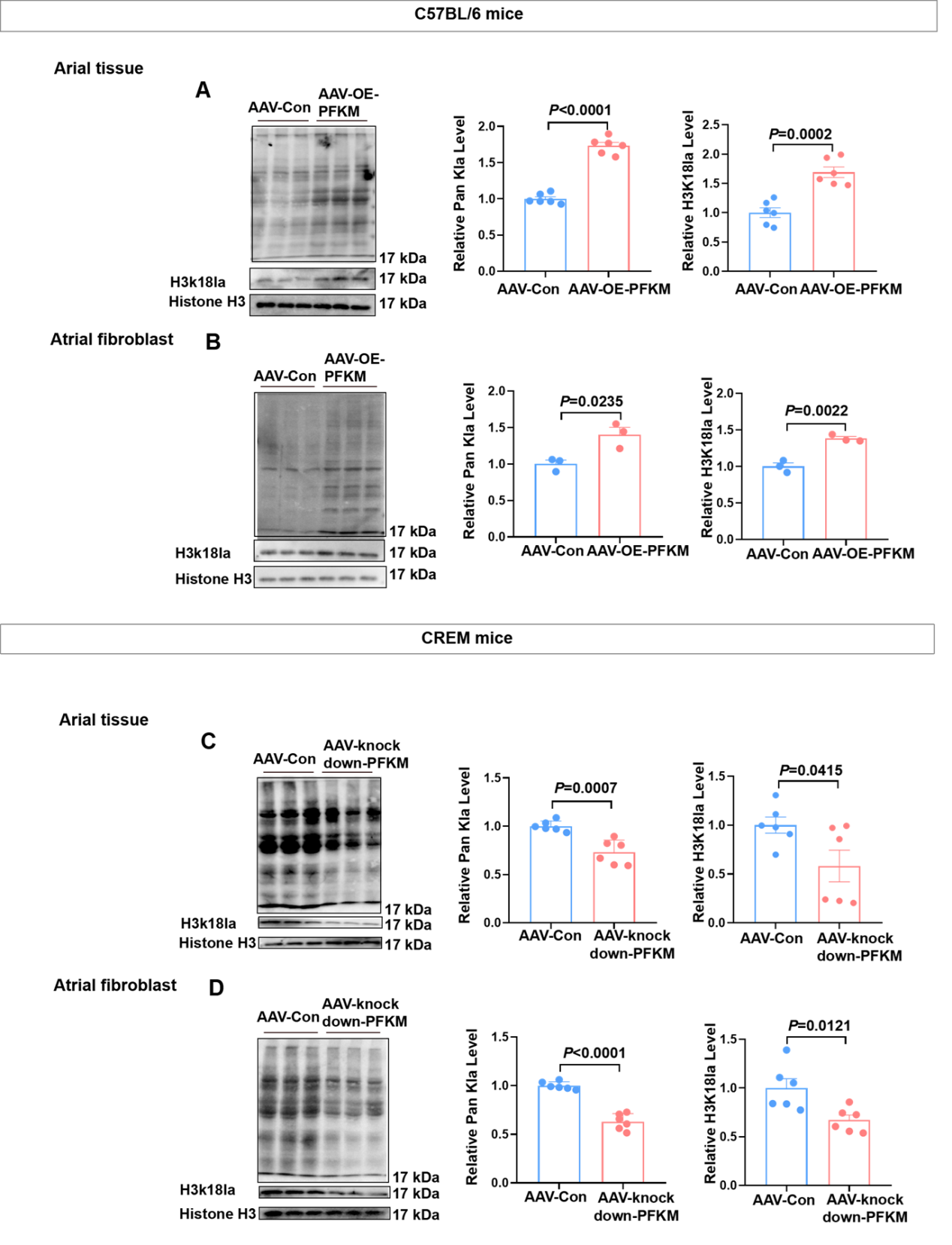


**Figure S13:** **Modulation of atrial PFKM levels altered glycolysis regulated histone lactylation expression.**

1. Representative bands and quantification of the protein levels of Pan Kla and H3K18la in atrial tissues in AAV-Con group and AAV-OE-PFKM group (n = 6).
2. Representative bands and quantification of the protein levels of Pan Kla and H3K18la in atrial fibroblasts in AAV-Con group and AAV-OE-PFKM group (n = 3).
3. Representative bands and quantification of the protein levels of Pan Kla and H3K18la in atrial tissues in CREM-Con group and CREM+ AAV-PFKM group (n = 6).
4. Representative bands and quantification of the protein levels of Pan Kla and H3K18la in atrial fibroblasts in AAV-Con group and AAV-OE-PFKM group (n = 6).

The data are given as mean ± SEM and compared by Student’s*t* test.

**
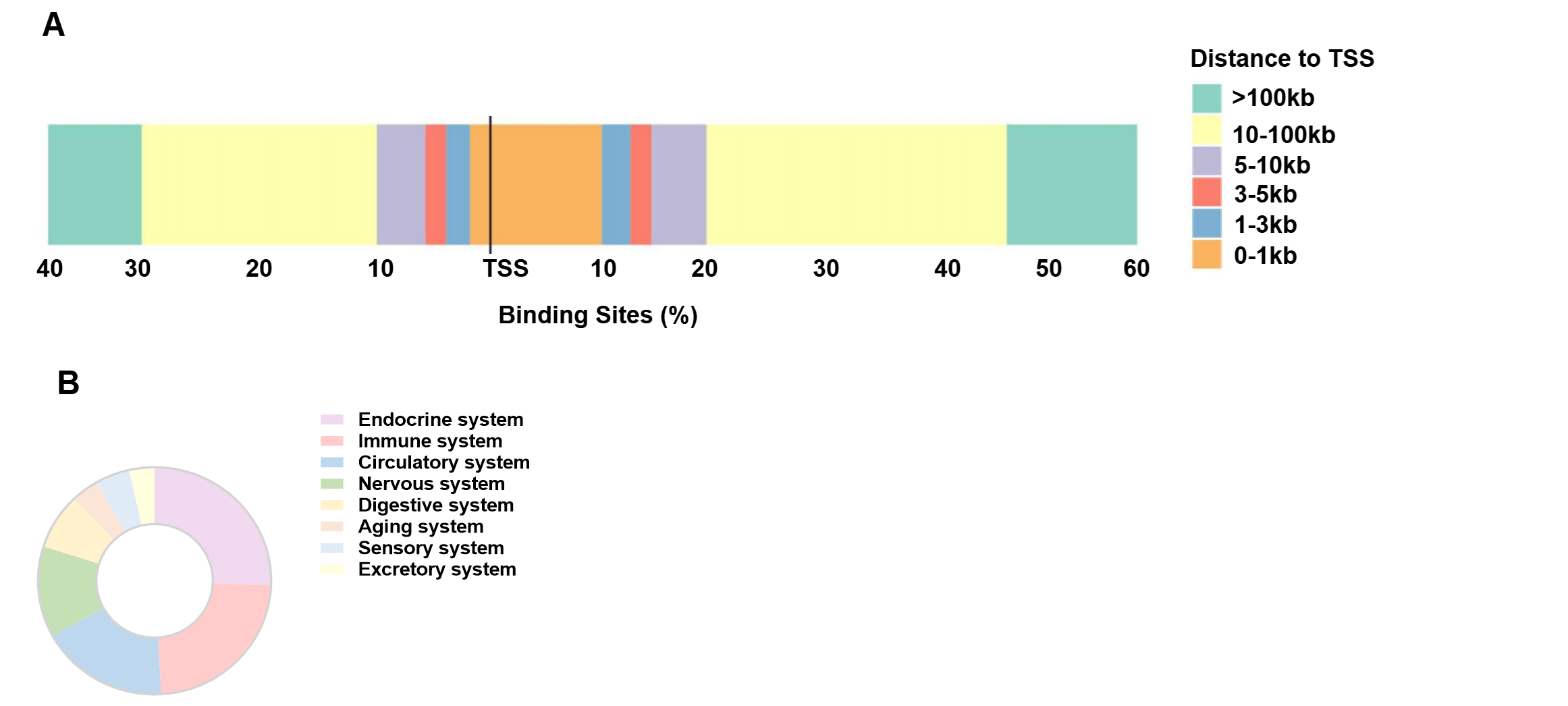
**

**Figure S14:** **H3K18la was enriched in the promoter and upstream regions of genes.**

1. Distribution of H3K18la peaks across gene regions (upper) and relative to TSS (bottom).
2. Relative diseases enriched by H3k18la -modified genes.

**Supplemental Resources-1. Sequences of primers for PCR in mice**

|  | Forward primer (5’-3’) | Reverse primer (3’-5’) |
| --- | --- | --- |
| Collagen III | CCACGGAAACACTGGTGGAC | GCCAGCTGCACATCAAGGAC |
| α*-*SMA | GACAATGGCTCTGGGCTCTGTAA | TGTGCTTCGTCACCCACGTA |
| TGF-β1 | TGGAGCAACATGTGGAACTC | GTCAGCAGCCGGTTACCA |
| BAX | CCCGAGAGGTCTTCTTCC | GCCTTGAGCACCAGTTTG |
| Bcl2 | TTCAGGATGGGGTGAACTG | CACAGGGCGATGTTGT |
| GAPDH | GATGCCCCCATGTTTGTGAT | GGCATGGACTGTGGTCATGAG |
| PFKM | GGAGTGCGTGCAGGTGACCAAA | ATCACGGCCACTGTGTGCAACC |
| CREM | TGACAGAGAAGCAGGCACTTTAC | TGGCAAAGCAGCAGTAGGAGCT |
| P300 | GATCCCGCGGAATACTATCACCTCCTA | TTTTTGGATTAGGAGGTGATAGTATTCCGC |
| GCN5 | GCTCTTGGGAATGGTAGTAGATG | GCAAGAGCTTGAAGAGGTAGAA |
| MOF | GACGAATGGGTGGACAAGAA | GCCAGCTCACTCAGGTATTT |
