# Supplementary material for: PFKM‐Driven Lactate Overproduction Promotes Atrial Fibrillation via Triggering Cardiac Fibroblasts Histone Lactylation
Source: Adv Sci (Weinh). 2025 Jun 26;12(34):e00963. doi: 10.1002/advs.202500963 (PMC12442653; doi:10.1002/advs.202500963)

Fig1C Human:SR AF (n = 6)

HK:102k Da

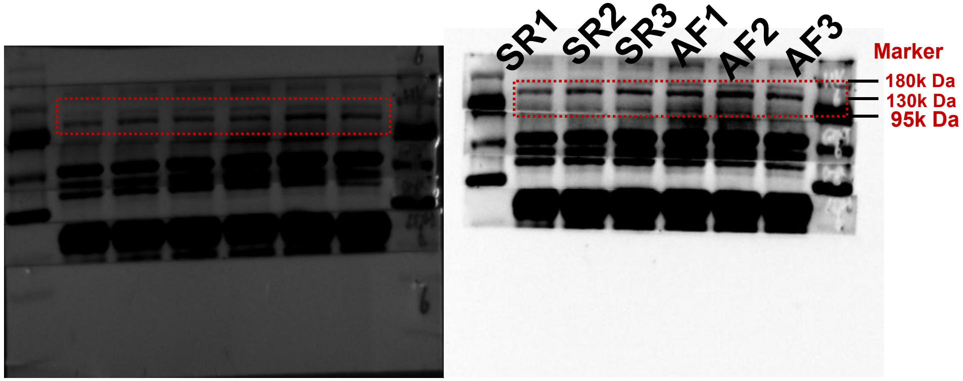

HK	BA			
13545330	19853992	0. 682247177	0. 684834613	0. 996222
15876307	23925787	0. 663564672		0. 968941
14391489	20307114	0. 708691988		1. 034837
16975212	19108846	0. 888343127		1. 302084
16320258	18170347	0. 898180866		1. 353569
13725310	16850337	0. 814542166		1. 14936

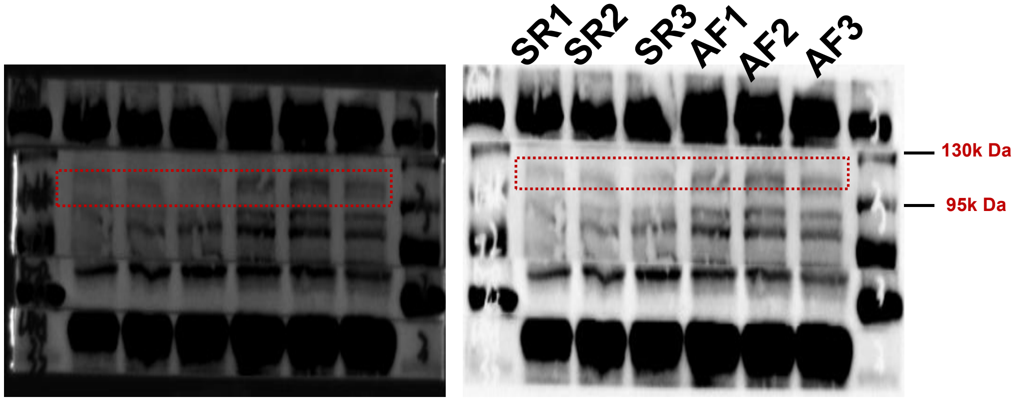

	HK	B-A			
N1	14932. 933	25767. 296	0. 579530464	0. 687755442	0. 842640317
N2	19143. 782	26350. 196	0. 726513837		1. 056354908
N3	20366. 782	26896. 711	0. 757222026		1. 101004775
C1	24059. 882	22387. 66	1. 074693916		1. 854421784
C2	27337. 539	16485. 418	1. 658286068		2. 189960158
C3	20330. 761	12821. 589	1. 585666254		2. 182568553

SR	AF
0.99622181	1.30208399
0.968941493	1.353569447
1.034836697	1.149359919
0.842640317	1.854421784
1.056354908	2.189960158
1.101004775	2.182568553

Fig1C Human SR AF(n = 6)

GLUT1：54k Da

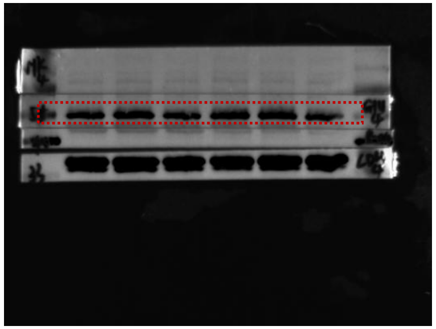

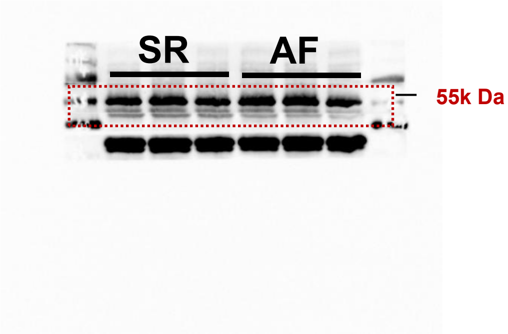

	GLUT	BA			
SR1	31995381	19853992	1.611534	1.483450358	1.086341638
SR2	28836292	23925787	1.205239		0.812456589
SR3	33173258	20307114	1.633578		1.101201773
AF1	38871670	19108846	2.034224		1.687817775
AF2	38010588	18170347	2.091902		1.298081389
AF3	34684647	16850337	2.058395		1.280564468

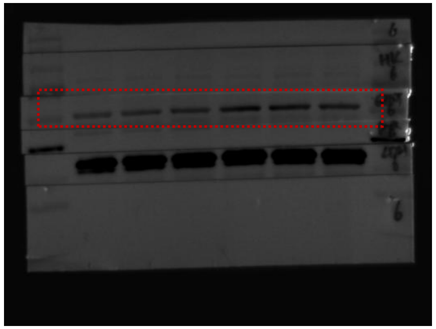

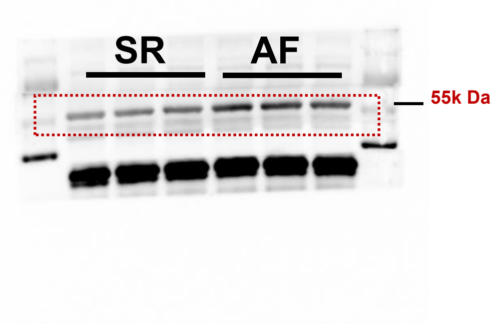

	GLUT	B-A			
SR1	16965. 347	25767. 296	0. 658406183	0. 755170948	0. 871863761
SR2	19177. 711	26350. 196	0. 727801455		0. 963757223
SR33	23650. 418	26896. 711	0. 879305206		1. 164379017
AF1	28424. 125	22387. 66	1. 269633584		1. 928343957
AF2	27230. 761	16485. 418	1. 65180895		2. 269587314
AF3	24772. 711	12821. 589	1. 932109273		2. 197313584

GLUT/β-actin	SR	AF
	1.086342	1.928343957
	0.812457	2.269587314
	1.101202	2.197313584
	0.871863761	1.687817775
	0.963757223	1.298081389
	1.164379017	1.280564468

Fig1C

PFKM：85k Da

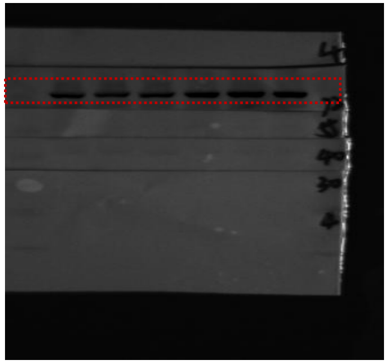

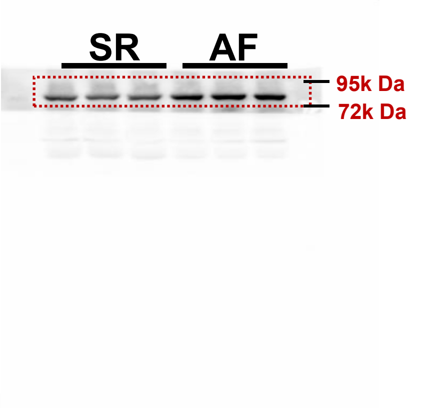

	PFKM	BA			
SR1	17014.539	27797.4	0.612091089	0.575414458	1.063739502
SR2	15425.468	28191.52	0.547166988		0.95090935
SR3	16672.468	29405.47	0.566985297		0.985351148
AF1	21854.589	22572.2	0.968208366		1.769493384
AF2	27221.589	21287.47	1.278761241		2.089168204
AF3	24285.225	20561.88	1.181079874		2.083087305

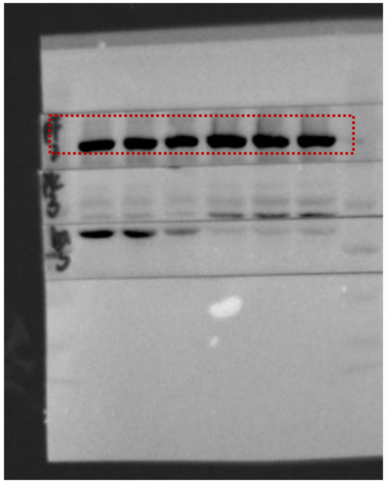

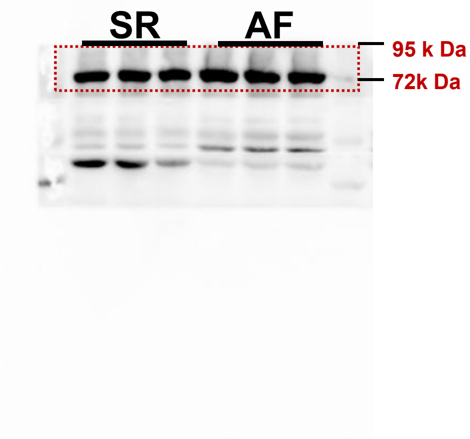

	PFKM	BA			
SR1	18857.35	28232.88	0.667921433	0.71998747	0.927684801
SR2	18933.18	27770.18	0.681780903		0.946934401
SR3	17646.52	21778.83	0.810260073		1.125380797
AF1	23552.02	13375.64	1.760814735		2.173147603
AF2	21841.37	15640.59	1.396454315		2.048244983
AF3	20891.08	17057.88	1.22471682		1.833624076

PFKM/β-actin	SR	AF
	0.927684801	2.173147603
	0.946934401	2.048244983
	1.125380797	1.833624076
	1.063739502	1.769493384
	0.95090935	2.089168204
	0.985351148	2.083087305

Fig1C

PKM：60k Da

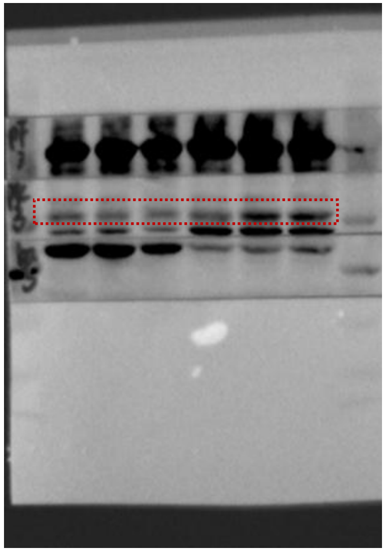

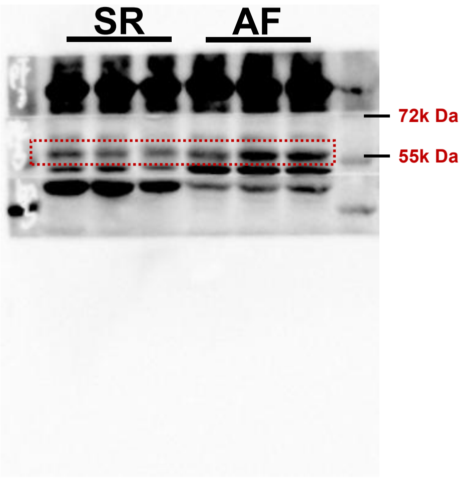

	PKM	BA			
SR1	19004. 589	28232. 882	0. 673136699	0. 69410356	0. 969792893
SR2	17724. 175	27770. 175	0. 638244988		0. 919524153
SR3	16789. 933	21778. 832	0. 770928992		1. 110682954
AF1	17478. 953	13375. 64	1. 306775078		1. 695065423
AF2	20871. 782	15640. 589	1. 33446266		1. 730979991
AF3	20964. 246	17057. 882	1. 229006391		1. 594188835

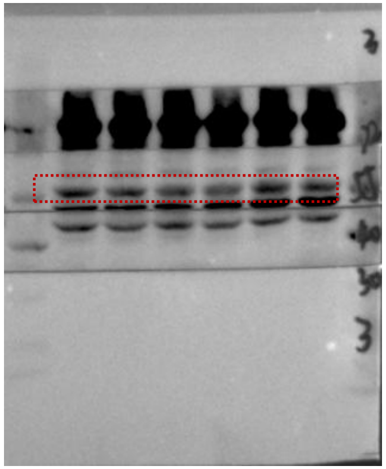

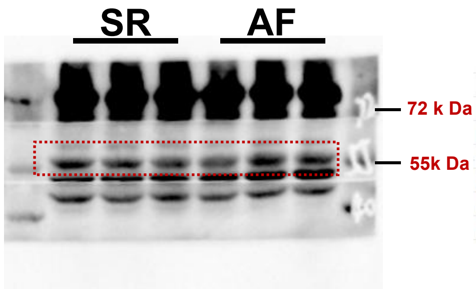

	PKM	BA			
SR1	19156. 761	21245. 76	0. 901674503	0. 841501217	1. 07150707
SR2	18048. 246	21068. 95	0. 856627569		1. 017975437
SR3	18252. 418	23821. 95	0. 766201579		0. 910517493
AF1	19742. 933	20477. 54	0. 964126256		1. 258319328
AF2	24330. 418	20318. 95	1. 197424789		1. 397835922
AF3	25160. 296	19831. 3	1. 268716679		1. 655852342

PKM/β-actin	SR	AF
	0. 969792893	1. 695065423
	0. 919524153	1. 730979991
	1. 110682954	1. 594188835
	1. 07150707	1. 258319328
	1. 017975437	1. 397835922
	0. 910517493	1. 655852342

Fig1C

LDHA：37k Da

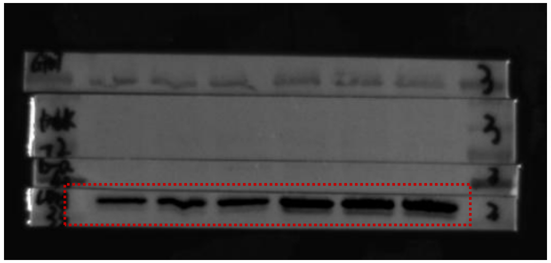

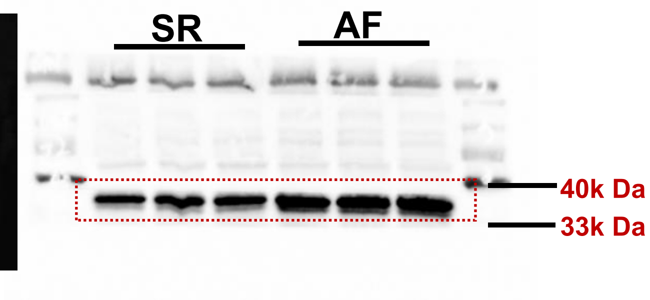

3	LDH	BA			
SR1	19985898	21614074	0.924671	1.009245	0.916199931
SR2	24980638	24595765	1.015648		1.006343888
SR3	27388809	25187018	1.087418		1.077456181
AF1	38561699	30320803	1.27179		1.375398128
AF2	39691028	29902692	1.32734		1.3068895
AF3	43178000	28776847	1.500442		1.379821537

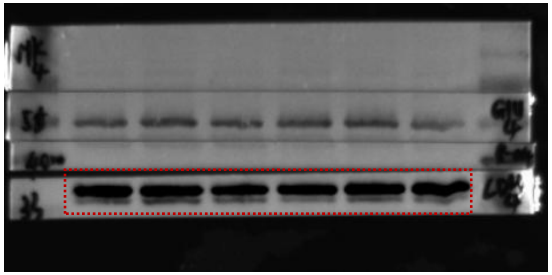

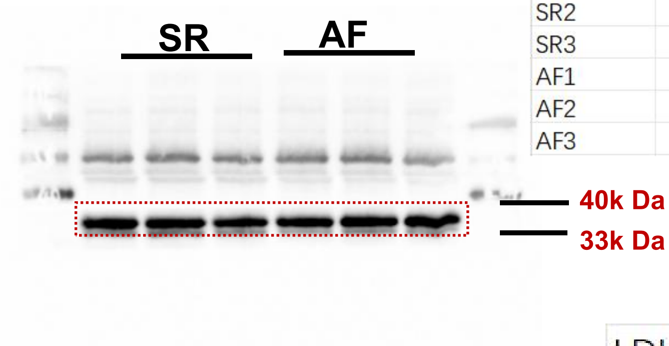

4	LDH	BA			
SR1	55719754	19853992	2.806476	2.648792	1.059530645
SR2	62356884	23925787	2.606263		0.983943923
SR3	51450850	20307114	2.533637		0.956525432
AF1	51404195	19108846	2.690073		1.061743852
AF2	57292585	18170347	3.153082		1.123501997
AF3	54849027	16850337	3.25507		1.248941693

LDH/β-actin	SR	AF
	0.91619993	1.375398
	1.00634389	1.306889
	1.07745618	1.379822
	1.05953064	1.061744
	0.98394392	1.123502
	0.95652543	1.248942

Fig1E：Pacing Rabbit (n=6)

HK：102k Da

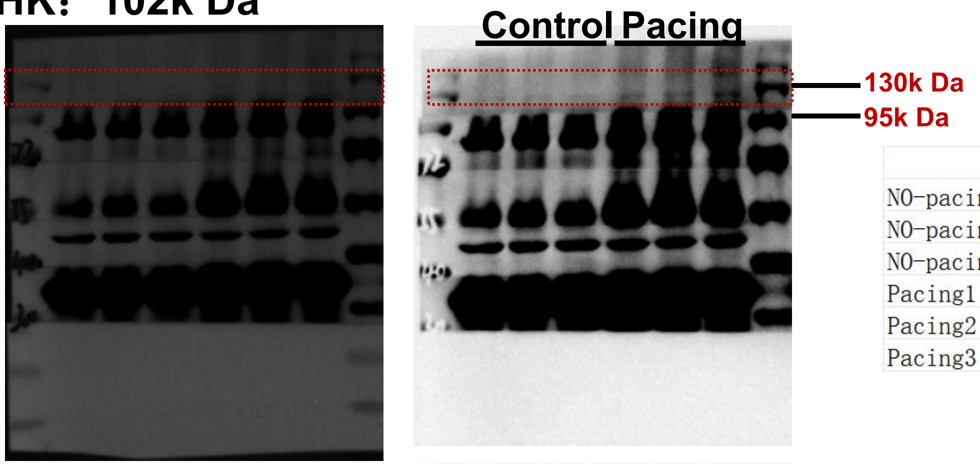

	HK	b-a			
NO-pacing1	9094.831998	20764.95332	0.437989523	0.356750376	1.227719864
NO-pacing2	7709.468037	22699.88225	0.339625904		0.951998729
NO-pacing3	7367.932504	25177.832	0.2926357		0.820281407
Pacing1	11707.19596	22648.24621	0.516914018		1.522009988
Pacing2	11023.78175	20586.76093	0.535479174		1.222584437
Pacing3	10579.24621	25336.10408	0.417556155		1.426880434

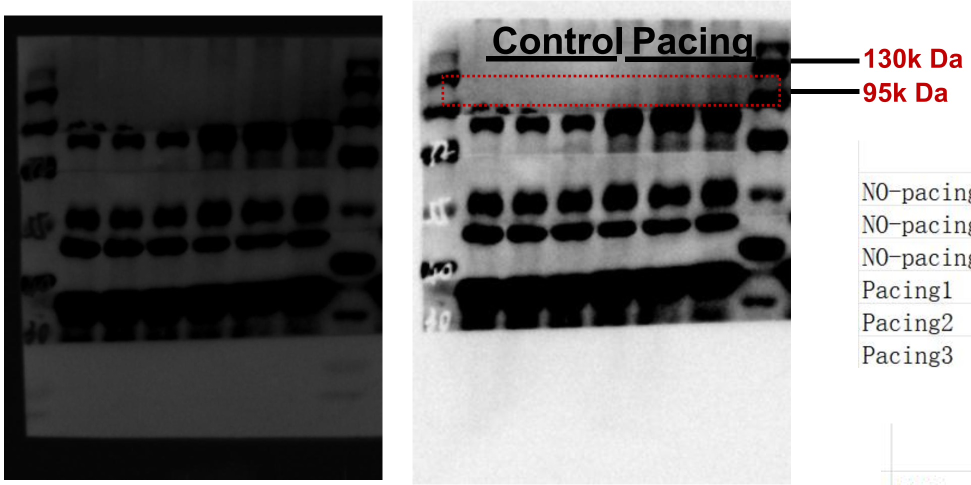

	HK	b-a			
NO-pacing1	8798.305087	26096.34672	0.337147003	0.283907549	1.187523909
NO-pacing2	5199.346717	21784.63961	0.238670311		0.840662081
NO-pacing3	6021.459415	21824.36753	0.275905334		0.97181401
Pacing1	10199.34672	17629.15433	0.578549971		2.096914771
Pacing2	10332.610173	20607.4386	0.501401963		2.100814134
Pacing3	11963.811183	20631.9325	0.579868666		1.719928283

	Non-Pacing	Pacing
HK	1.187523909	2.096914771
	0.840662081	2.100814134
	0.97181401	1.719928283
	1.227719864	1.522009988
	0.951998729	1.222584437
	0.820281407	1.426880434

Fig1E

GLUT1：54k Da

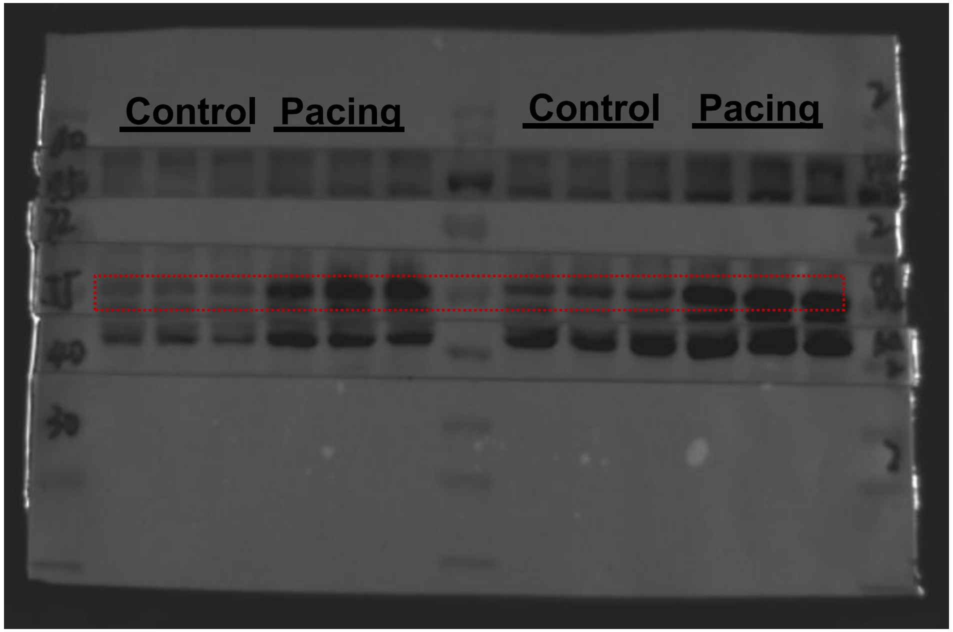

	GLUT	ba			
NO-pacing	8886.104076	13708.74012	0.648207202	0.598521586	1.083013909
NO-pacing	7633.325902	14597.9325	0.522904589		0.873660369
NO-pacing	7901.861436	12654.05382	0.624452965		1.043325722
Pacing1	19974.12489	27929.95332	0.715150672		1.367650403
Pacing2	24039.10408	25637.46804	0.937655155		1.501562499
Pacing3	23706.00357	23314.51829	1.016791481		1.568621078

	GLUT	ba			
NO-pacing	15211.58936	19093.246212025	0.796700005	0.770387376	1.03415506[illegible]
NO-pacing	14228.10408	16804.104076401	0.846704115		1.099062811
NO-pacing	14988.51829	22446.033008589	0.667758008		0.86678212[illegible]
Pacing1	27534.2254	26535.225396744	1.037648069		1.2255143[illegible]
Pacing2	25623.86144	25988.396969620	0.985973143		1.23757140[illegible]
Pacing3	23878.05382	25300.417784900	0.943781009		1.4133578[illegible]

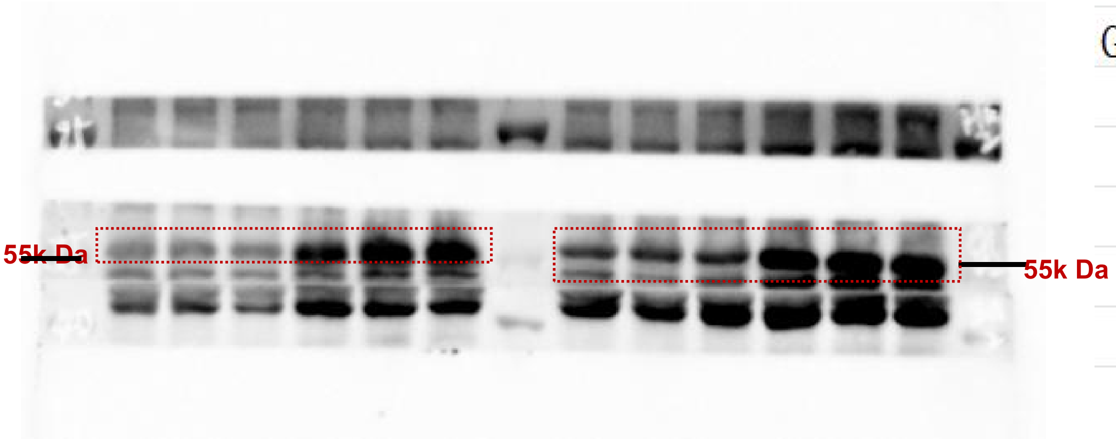

GLUT/b-actin	NO-pacing	Pacing
	1.08301391	1.2255144
	0.87366037	1.2375714
	1.04332572	1.4133578
	1.03415506	1.3676504
	1.09906281	1.5015625
	0.86678213	1.5686211

Fig1E

PFKM：85k Da

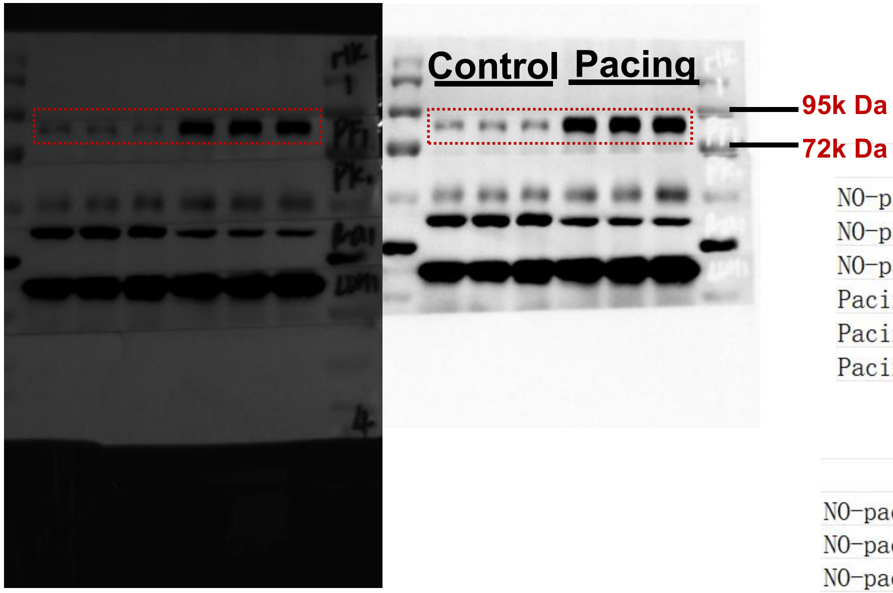

	PFKM	b-a			
NO-pacing	9592.003571	28249.29646	0.339548406	0.344596783	0.9853499
NO-pacing	9506.124892	28546.29646	0.333007292		0.966367967
NO-pacing	8793.175144	24342.00357	0.36123465		1.048282132
Pacing1	16027.003571	20203.00357	0.793298062		2.382224293
Pacing2	14845.710678	18161.76093	0.817415819		2.407361676
Pacing3	16273.76093	18392.63961	0.884797467		2.449370419

	PFKM	b-a			
NO-pacing1	14544.78175	26096.34672	0.557349345	0.562523832	0.990801302
NO-pacing2	12857.34672	21784.63961	0.590202406		1.04920427
NO-pacing3	11785.58936	21824.36753	0.540019744		0.959994427
Pacing1	24757.07464	17629.15433	1.404325708		2.379396786
Pacing2	27289.50967	20607.4386	1.324255294		2.375987891
Pacing3	25282.63961	20631.9325	1.225413063		2.269200484

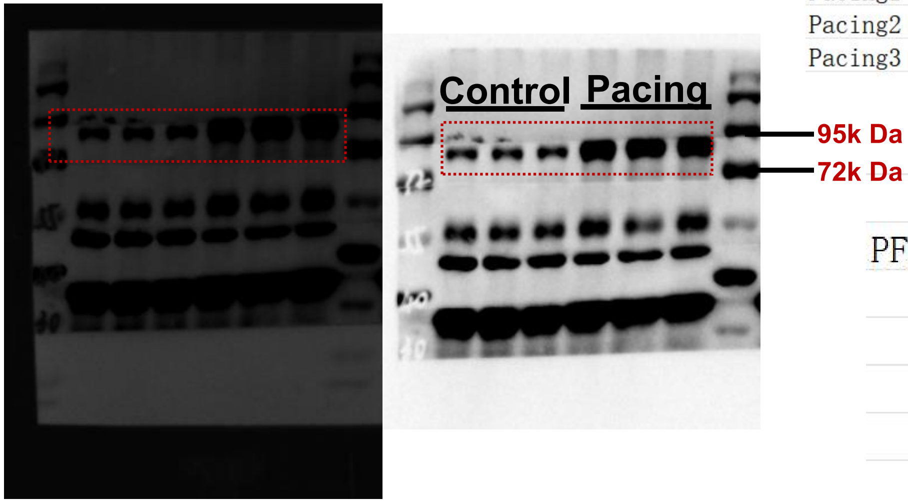

	Non-Pacing	Pacing
PFKM	0.990801302	2.379396786
	1.04920427	2.375987891
	0.959994427	2.269200484
	0.9853499	2.382224293
	0.966367967	2.407361676
	1.048282132	2.449370419

Fig1E：Pacing Rabbit

PKM：60k Da

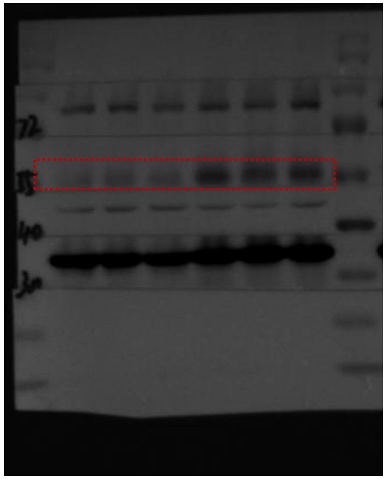

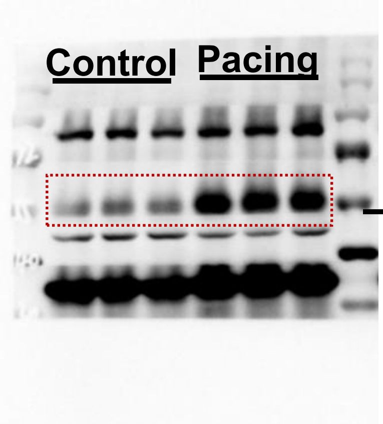

55k Da

	PKM	b-a			
NO-pacing1	11877.41778	20764.95332	0.571993474	0.609307596	0.938759795
NO-pacing2	14888.76093	22699.88225	0.655895954		1.076461148
NO-pacing3	15107.53911	25177.832	0.600033359		0.984779056
Pacing1	26633.00357	22648.24621	1.1759411		1.959792873
Pacing2	27617.2254	20586.76093	1.341504158		2.045300249
Pacing3	26080.63961	25336.10408	1.029386347		1.799647012

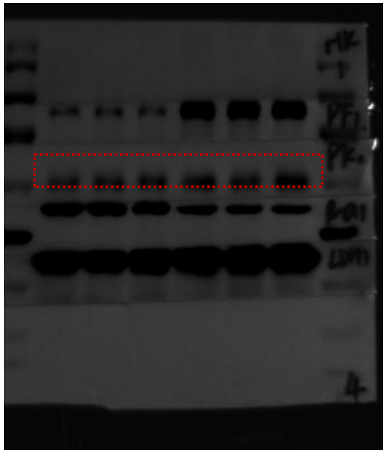

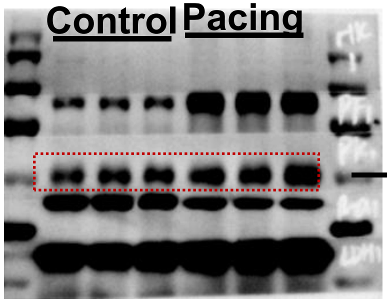

55k Da

	PKM	b-a			
NO-pacing	14191.53911	28249.29646	0.502367877	0.547058691	0.918307095
NO-pacing	15327.95332	28546.29646	0.536950681		0.981522987
NO-pacing	14650.41778	24342.00357	0.601857515		1.100169918
Pacing1	19390.41778	20203.00357	0.959778961		1.910510215
Pacing2	17943.53911	18161.76093	0.987984545		1.839991232
Pacing3	22261.225396	18392.63961	1.210333365		2.010996514

	Non-Pacing	Pacing
PKM	0.918307095	1.910510215
	0.981522987	1.839991232
	1.100169918	2.010996514
	0.938759795	1.959792873
	1.076461148	2.045300249
	0.984779056	1.799647012

Fig1E： Pacing Rabbit

LDHA： 37k Da

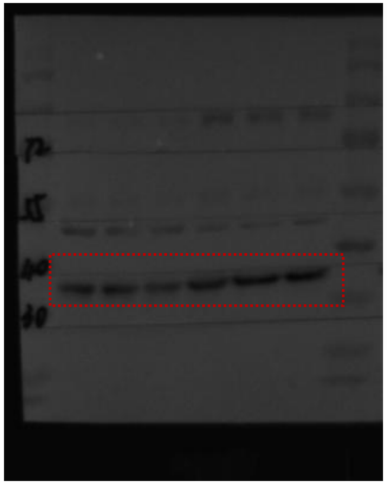

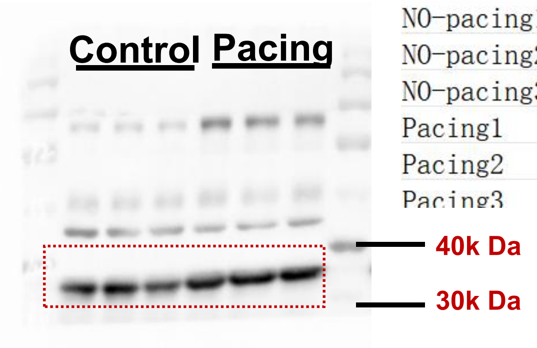

	LDH	b-a			
NO-pacing1	19650.24	26096.34672	0.752988156	0.874809812	0.860744982
NO-pacing2	22987.93	21784.63961	1.055235726		1.206245874
NO-pacing3	17813.17	21824.36753	0.816205554		0.933009144
Pacing1	25725.17	17629.15433	1.459240161		1.787834147
Pacing2	30481.9	20607.4386	1.47916976		1.401743443
Pacing3	28995.07	20631.9325	1.405349208		1.866362965

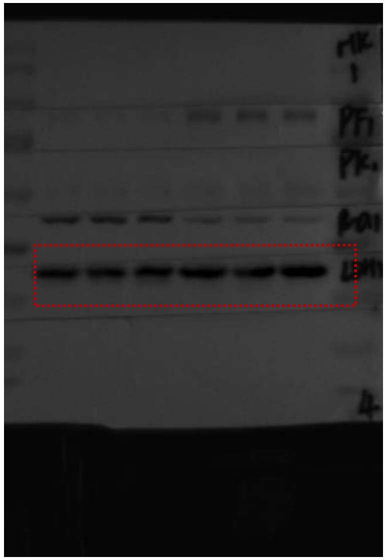

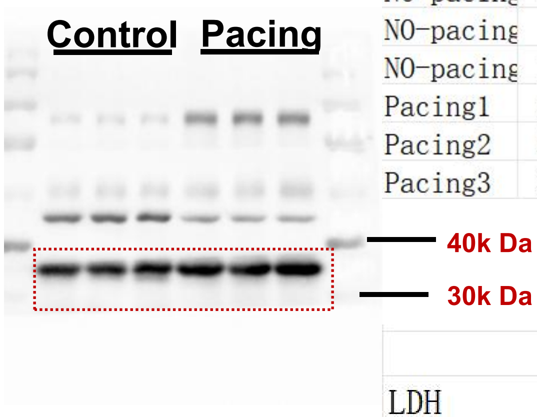

	LDH	b-a			
NO-pacing	16853.88225	28249.29646	0.59661246	0.651707505	0.915460471
NO-pacing	15976.9325	28546.29646	0.55968495		0.858797767
NO-pacing	19445.00357	24342.00357	0.798825106		1.225741762
Pacing1	24384.61017	20203.00357	1.206979452		2.156533693
Pacing2	22853.53911	18161.76093	1.258332779		2.109129232
Pacing3	30040.95332	18392.63961	1.633313867		2.044645136

	Non-Pacing	Pacing
LDH	0.860744982	1.787834147
	1.206245874	1.401743443
	0.933009144	1.866362965
	0.915460471	2.156533693
	0.858797767	2.109129232
	1.225741762	2.044645136

Fig1I：CREM mice （7month）(n=6)

HK：102k Da

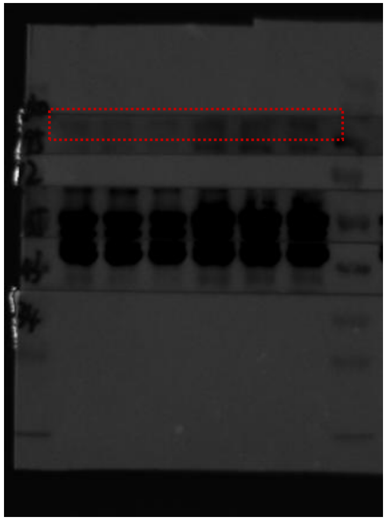

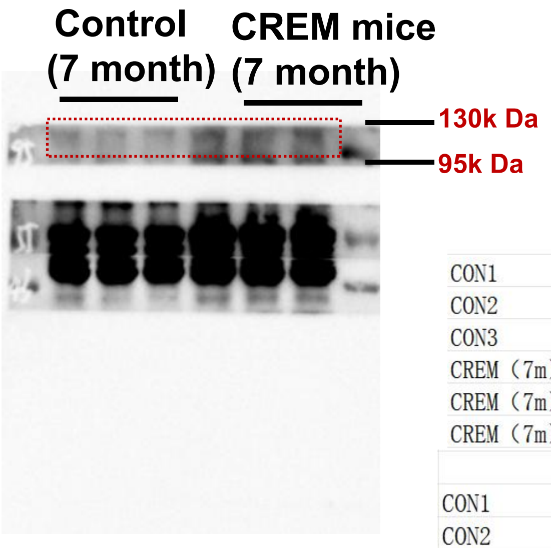

	HK	bactin			
CON1	11641.2254	22659.81118	0.513738853	0.422093675	1.217120472
CON2	7666.225397	20969.12489	0.365595867		0.866148651
CON3	8007.175144	20693.24621	0.386946304		0.916730876
CREM（7m）1	25836.4386	24563.48885	1.05182284		2.718265631
CREM（7m）2	26843.36753	24652.24621	1.088881204		2.119522786
CREM（7m）3	24125.51829	24652.58936	0.978620052		2.529084893

	HK	bactin			
CON1	9018.376154	22579.58936	0.399403905	0.390231487	1.023505069
CON2	9061.982756	25679.90307	0.35288228		0.904289614
CON3	9096.053824	21739.66043	0.418408275		1.072205318
CREM（7m）1	24024.38835	22096.29646	1.087258599		2.598559024
CREM（7m）2	22651.71068	21585.29646	1.049404659		2.627427141
CREM（7m）3	17027.98276	23685.00357	0.718935199		2.037323036

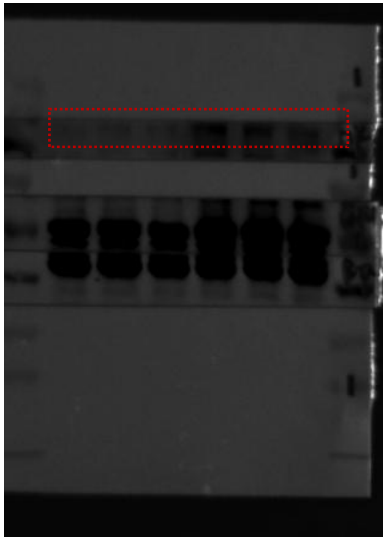

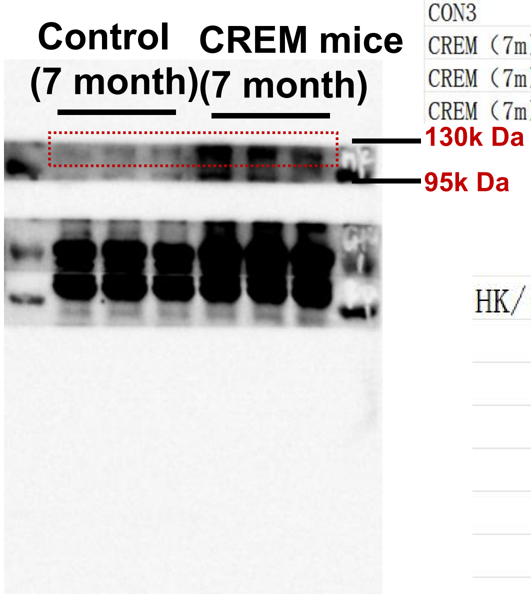

HK/β-act	CON	CREM（7m）
	1.217120472	2.718265631
	0.866148651	2.119522786
	0.916730876	2.529084893
	1.023505069	2.598559024
	0.904289614	2.627427141
	1.072205318	2.037323036

Fig1I：CREM mice （7month）

GLUT1：54k Da

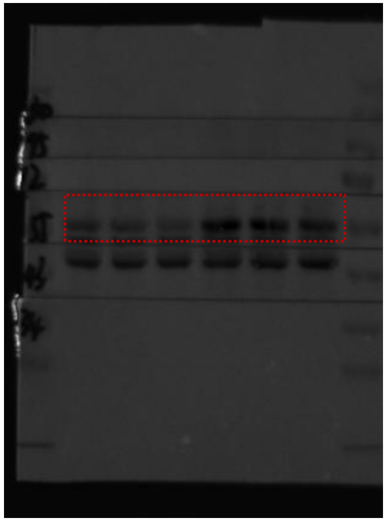

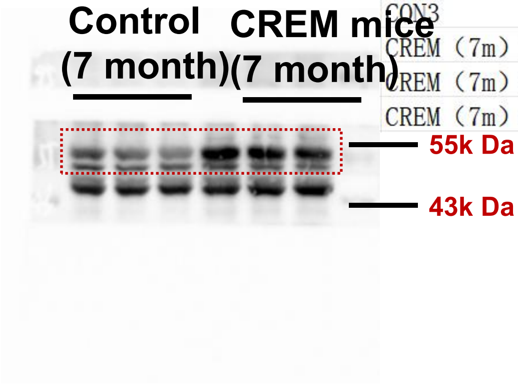

	glut	bactin			
CON1	14588.34672	22659.81118	0.643798247	0.594420479	1.083068753
CON2	12620.19596	20969.12489	0.601846573		1.012492999
CON3	11125.03301	20693.24621	0.537616616		0.904438248
CREM（7m）1	25666.61017	24563.48885	1.044908984		1.623037945
CREM（7m）2	21896.07464	24652.24621	0.888197954		1.475788005
CREM（7m）3	19334.46804	24652.58936	0.784277374		1.458804192

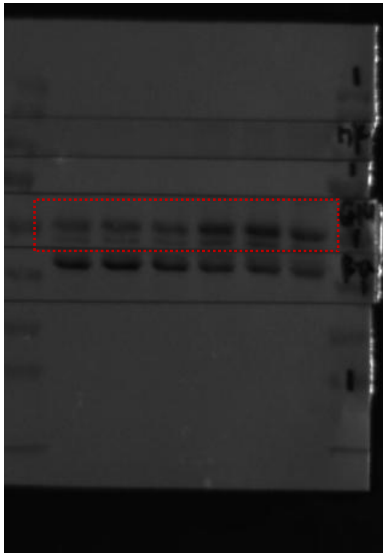

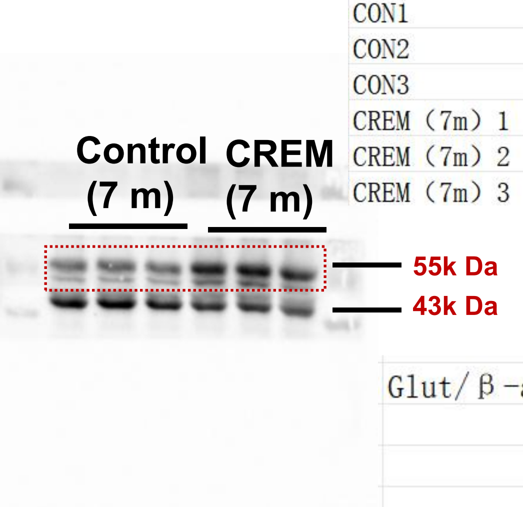

	glut	bactin			
CON1	17612.51829	22579.58936	0.780019424	0.764587394	1.020183474
CON2	19798.07464	25679.90307	0.770955972		1.008329431
CON3	16147.9325	21739.66043	0.742786786		0.971487095
CREM（7m）1	27725.05382	22096.29646	1.254737592		1.60859788
CREM（7m）2	26590.71068	21585.29646	1.231889991		1.597873336
CREM（7m）3	21711.71068	23685.00357	0.916685979		1.23411724

Glut/β-acti	CON	CREM（7m）
	1.083068753	1.623037945
	1.012492999	1.475788005
	0.904438248	1.458804192
	1.020183474	1.60859788
	1.008329431	1.597873336
	0.971487095	1.23411724

Fig1I：CREM mice （7month）

PFKM：85k Da

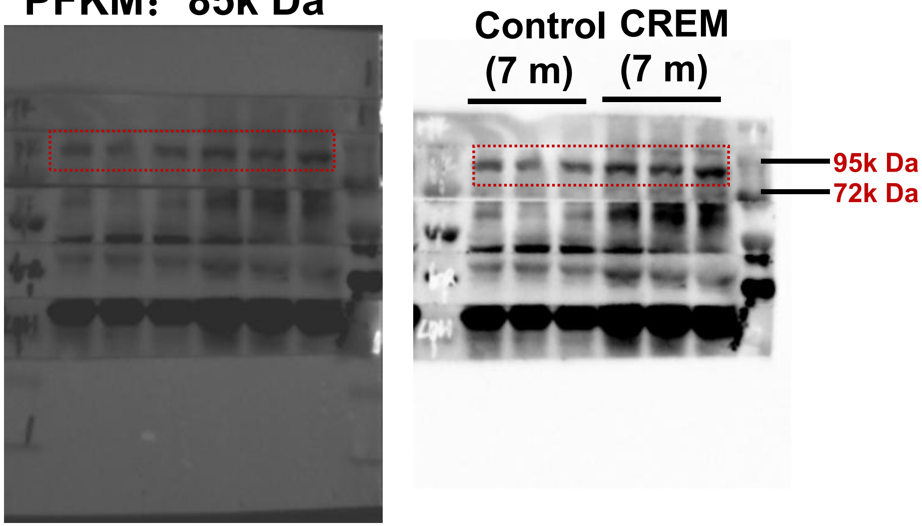

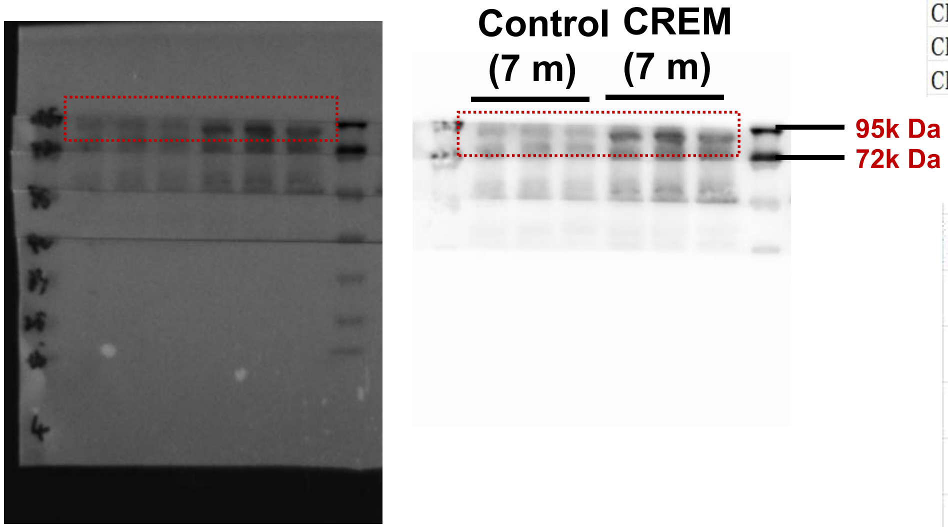

右	PFKM	b-a			
Con1	19533.104	15877.518	1.230236615	1.0576451	1.16318471
Con2	16028.054	20749.125	0.772468911		0.73036684
Con3	19034.418	16265.539	1.170229772		1.10644844
CREM1	27755.004	23534.66	1.179324622		1.52669525
CREM2	27843.246	21494.175	1.295385657		1.10694983
CREM3	27940.761	18531.418	1.507750837		1.22557792

	PFKM	BA			
Con1	14104.225	20623.782	0.683881598	0.79659668	0.85850420
Con2	15722.225	19483.974	0.806931122		1.01297324
Con3	16184.882	18003.66	0.898977319		1.12852255
CREM1	21745.125	19772.539	1.09976392		1.60812035
CREM2	22957.347	18047.539	1.272048616		1.57640296
CREM3	20389.933	18360.983	1.110503343		1.23529628

PFKM/b-a	CON	CREM
	1.163184716	1.526695255
	0.730366842	1.106949838
	1.106448442	1.225577924
	0.858504204	1.608120357
	1.012973243	1.576402968
	1.128522553	1.235296285

Fig1I：CREM mice （7month）

PKM：60k Da

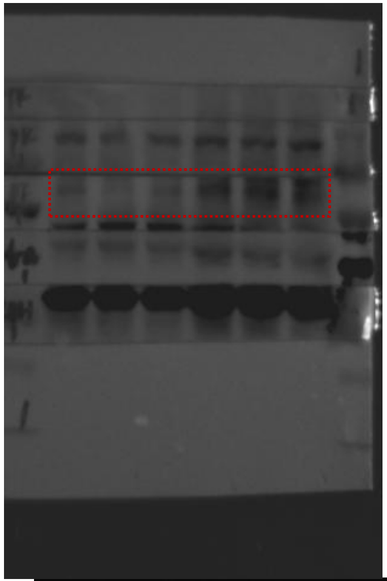

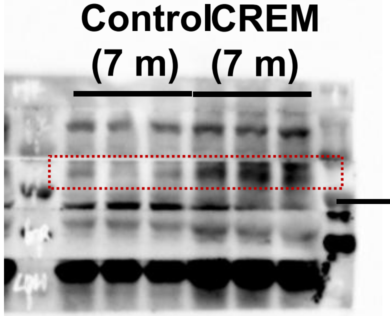

	PKM	b-a			
Con1	17500.69	15877.518	1.10223084	0.898775589	1.226369355
Con2	13025.589	20749.125	0.627765701		0.69846768
Con3	15717.882	16265.539	0.966330227		1.075162965
CREM1	23758.983	23534.66	1.009531601		1.123229885
CREM2	26924.891	21494.175	1.252659895		1.296306231
CREM3	24474.355	18531.418	1.320695211		1.198202013

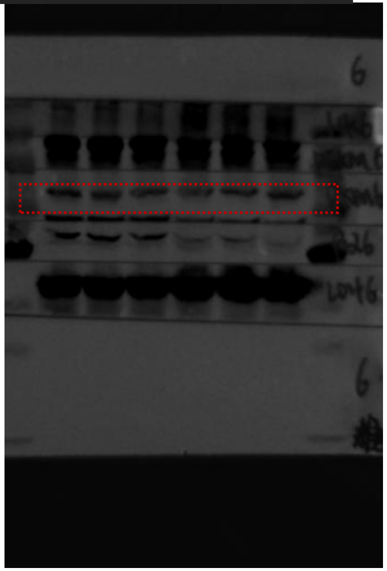

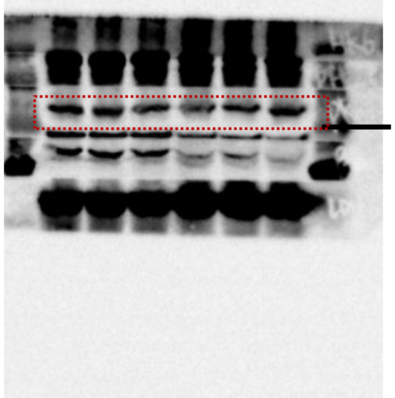

	PKM	BA			
Con1	20769.933	20623.782	1.007086528	1.062515963	0.947831903
Con2	20769.933	19483.974	1.066000858		1.003279852
Con3	20064.368	18003.66	1.114460504		1.048888245
CREM1	18426.075	11772.539	1.565174259		1.468267353
CREM2	20337.075	11047.539	1.840869265		1.651803055
CREM3	21760.104	9360.983	2.324553308		2.308196212

PKM/b-a	CON	CREM
	0.947831903	1.468267353
	1.003279852	1.651803055
	1.048888245	2.308196212
	1.226369355	1.123229885
	0.69846768	1.296306231
	1.075162965	1.198202013

Fig1I：CREM mice （7month）

LDHA：37k Da

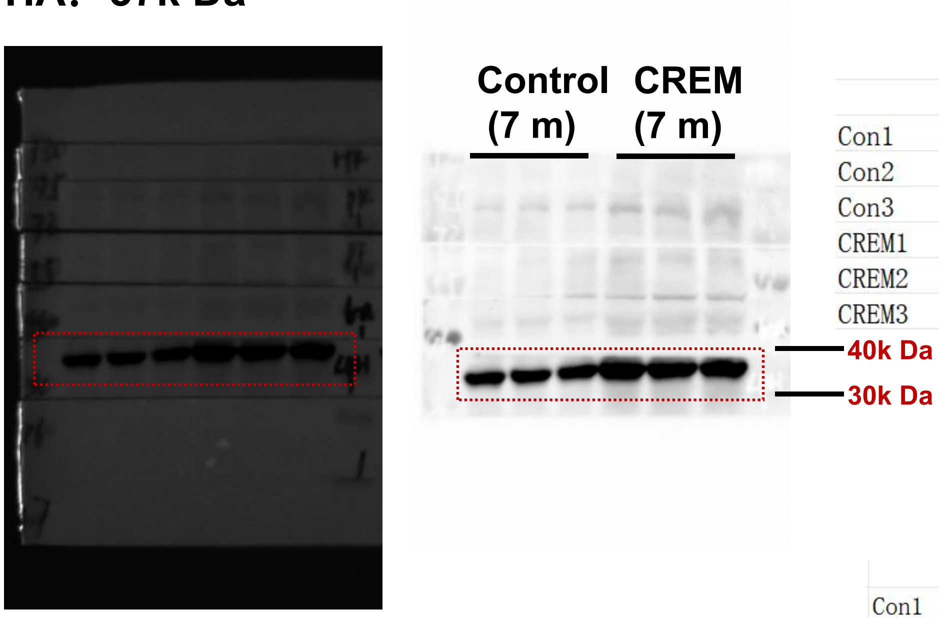

	LDHA	b-a			
Con1	13590. 6396	11198. 983	1. 213560161	1. 15728569	1. 048626256
Con2	13731. 2756	13765. 296	0. 997528542		0. 861955307
Con3	14365. 3259	11394. 104	1. 260768368		1. 089418437
CREM1	32667. 8614	16292. 933	2. 005032577		1. 59032589
CREM2	30311. 9325	16425. 782	1. 845387483		1. 520639473
CREM3	29450. 2964	18040. 347	1. 632468403		1. 636512977

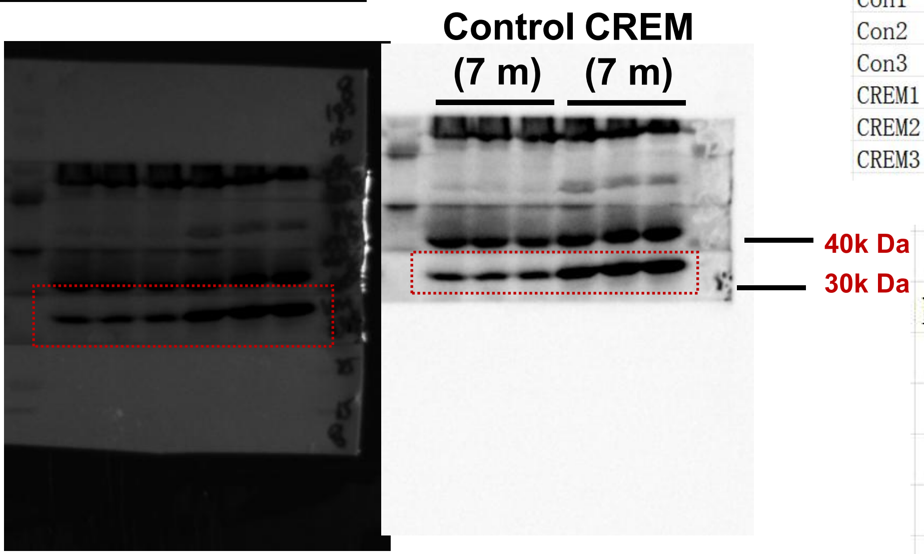

	LDHA	b-a			
Con1	12913. 831	29095. 246212025	0. 443846802	0. 460741607	0. 96333128
Con2	11377. 932	27302. 003571337	0. 416743499		0. 904505894
Con3	11767. 275	22558. 468037432	0. 52163452		1. 132162826
CREM1	24064. 346	28327. 003571337	0. 84951967		2. 038471317
CREM2	27018. 124	25205. 296464556	1. 07192252		2. 415073204
CREM3	30500. 539	24655. 124891681	1. 237087187		2. 371559281

	Con （7 month）	CREM（7 month）
LDHA/β-actin	1. 048626256	1. 59032589
	0. 861955307	1. 520639473
	1. 089418437	1. 636512977
	0. 96333128	2. 038471317
	0. 904505894	2. 415073204
	1. 132162826	2. 371559281

Fig1K：CREM mice （5month）

HK：102k Da

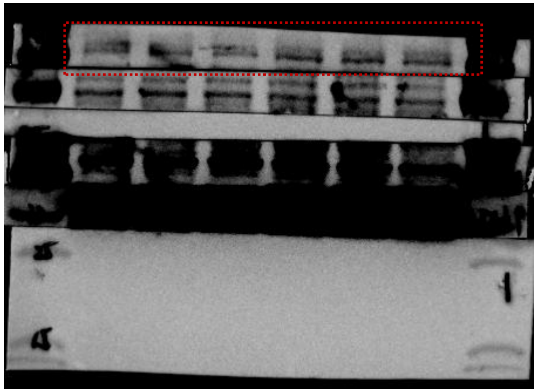

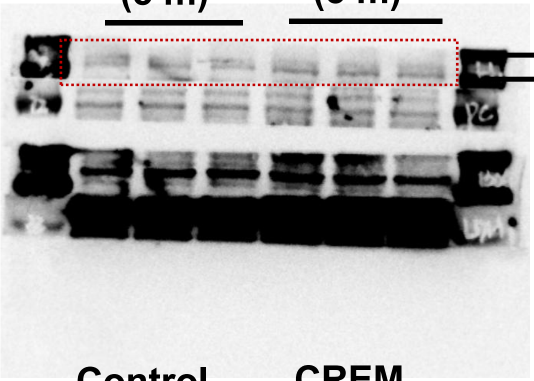

1	HK	BA			
C1	75527	160101	0.471746	0.454514	1.03791366
C2	78045	156766	0.497844		1.09533318
C3	63839	162048	0.393951		0.86675316
CREM1	105542	187323	0.563423		1.43018367
CREM2	114989	169037	0.680259		1.36641088
CREM3	102681	180078	0.570203		1.20870757

2	HK	BA			
C1	71867	205286	0.350082	0.384083	0.91147482
C2	76724	183558	0.417982		1.08825936
C3	85721	223124	0.384185		1.00026583
CREM1	131802	164046	0.803445		2.2950184
CREM2	111913	152589	0.733428		1.75468592
CREM3	128260	203932	0.628935		1.63749638

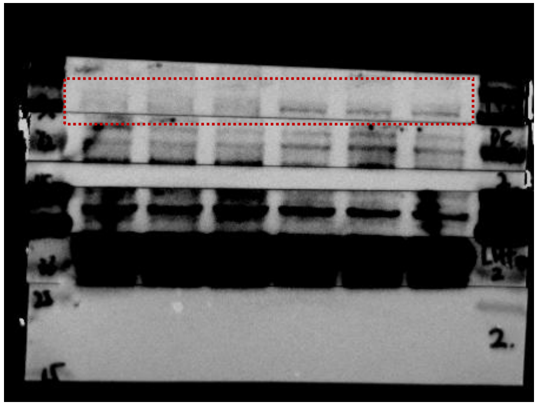

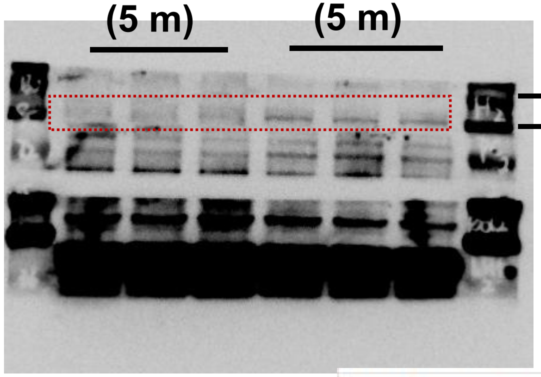

HK/b-actin	Con	Crem
	1.037913655	1.430183671
	1.09533318	1.366410885
	0.866753165	1.208707567
	0.911474816	2.295018399
	1.088259357	1.754685921
	1.000265827	1.637496375

Fig1K：CREM mice （5month）

GLUT1：54k Da

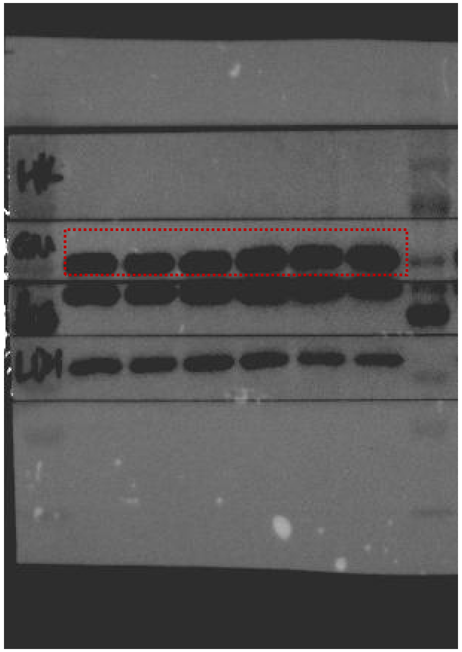

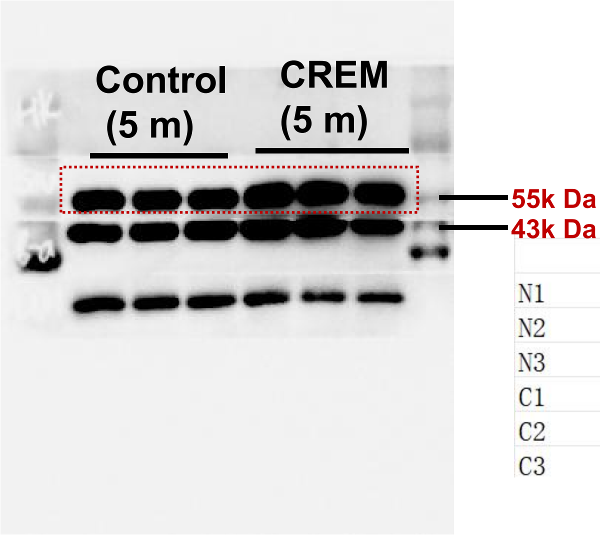

	GLUT	b-a			
N1	25735. 59	28608. 3	0. 899584827	0. 924670228	0. 972870975
N2	26828. 93	27569. 4	0. 973141814		1. 052420403
N3	25635. 95	28443. 81	0. 901284044		0. 974708622
C1	26962. 88	29642. 91	0. 909589517		1. 011121453
C2	25080. 42	26140. 45	0. 959448704		1. 064535327
C3	26348. 1	23082. 91	1. 141454943		1. 17295848

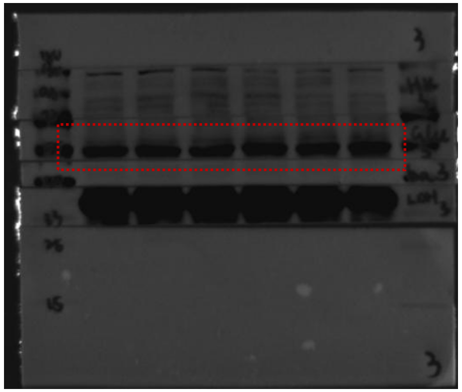

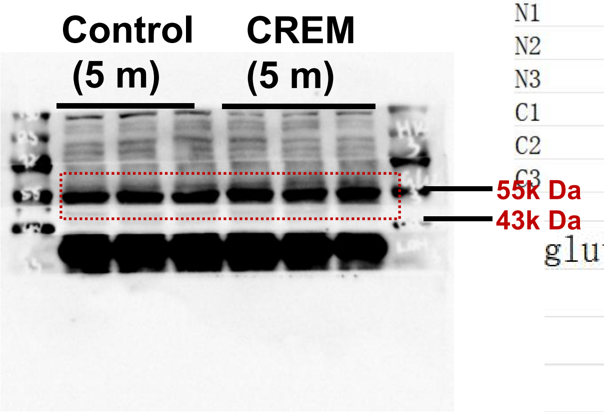

	GLUT	b-a			
N1	22782. 35	25851	0. 881294475	0. 915874468	0. 962243741
N2	21942. 69	22593. 64	0. 971188795		1. 060395096
N3	24834. 86	27744. 1	0. 895140135		0. 977361162
C1	26553. 18	28764. 18	0. 923133551		1. 047474569
C2	26553. 71	25253. 47	1. 051487701		1. 17466267
C3	24787. 38	20631. 76	1. 201418386		1. 237059563

glut/bactin	CON	CREM
	0.972871	1.0111215
	1.0524204	1.0645353
	0.9747086	1.1729585
	0.9622437	1.0474746
	1.0603951	1.1746627
	0.9773612	1.2370596

Fig1K：CREM mice （5month）

PFKM：85k Da

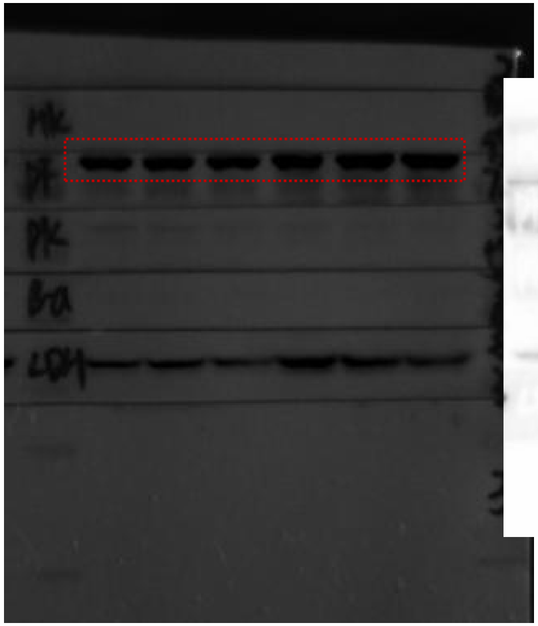

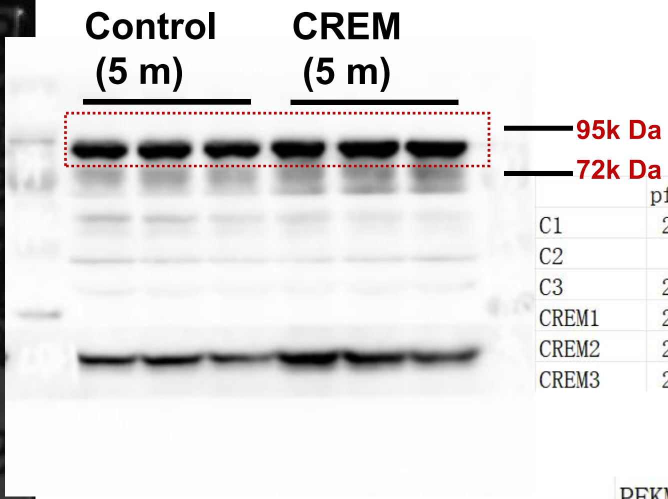

	pfkm	b-a			
C1	22310.347	23536.075	0.947921308	1.162294862	0.815560095
C2	22379.64	18937.953	1.1817349		1.016725565
C3	22430.589	16526.761	1.357228376		1.167714339
CREM1	25994.861	17206.175	1.510786738		1.113141137
CREM2	28725.397	20990.075	1.368522838		1.15806247
CREM3	28260.004	21852.711	1.293203576		1.364252037

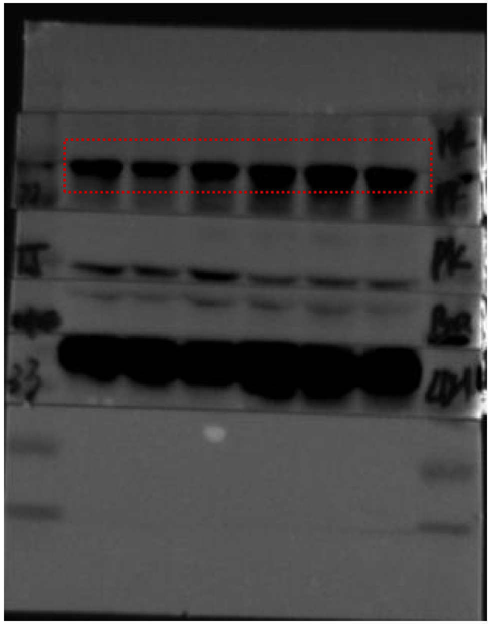

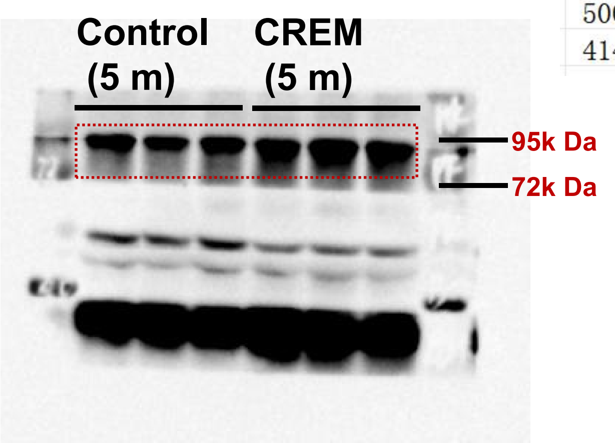

PFKM	ba			
37132246	44173523	0.8406	0.829716	1.013117244
26223160	37570190	0.697978		0.841224977
37785908	39750764	0.950571		1.145657779
45484108	39812751	1.142451		1.359090299
50001382	42422853	1.178643		1.239931671
41432786	37315539	1.110336		1.590789751

	con	CREM
PFKM/β-actin	0.8155601	1.1131411
	1.0167256	1.1580625
	1.1677143	1.364252
	1.0131172	1.3590903
	0.841225	1.2399317
	1.1456578	1.5907898

Fig1K：CREM mice （5month）

PKM：60k Da

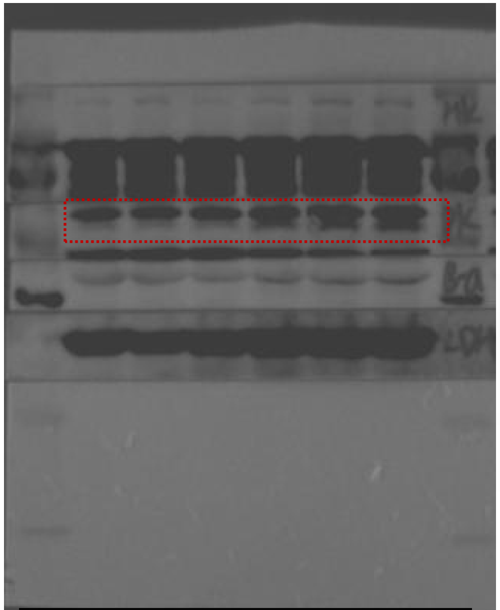

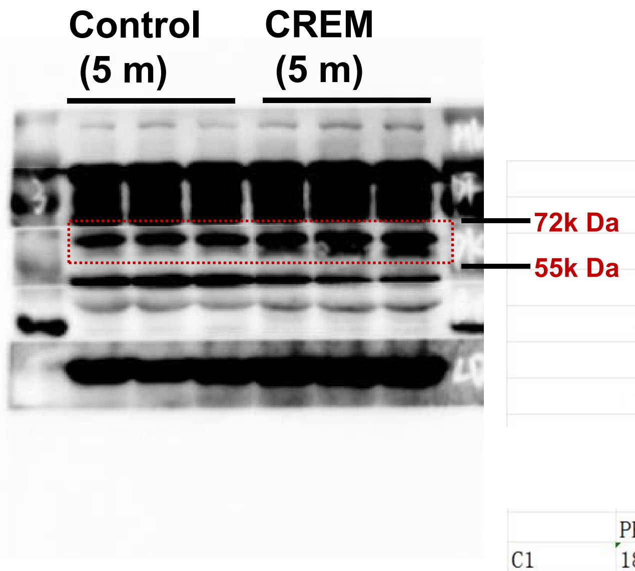

	PKM				
1	19587. 88	22149. 13	0. 884364	0. 923442	0. 957681526
2	18108. 1	20805. 66	0. 870345		0. 942500714
3	19278. 35	18981. 88	1. 015618		1. 09981776
4	23939. 69	21769. 05	1. 053775		1. 037570139
5	25907	24985. 59	1. 036878		1. 172456383
6	26846. 03	26663. 37	1. 006851		1. 156840887

	PKM	b-a			
C1	18400. 66[illegible]	22951. 660425587	0. 801713692	0. 771588608	1. 039042936
C2	19424. 58[illegible]	26873. 003571337	0. 722829114		0. 936806359
C3	17490. 29[illegible]	22133. 367532368	0. 790223017		1. 024150705
CREM1	23849. 63[illegible]	23185. 346717088	1. 028651411		1. 423090729
CREM2	24741. 66[illegible]	22863. 417784900	1. 082150563		1. 369424251
CREM3	27514. 93[illegible]	25144. 710678119	1. 094263237		1. 364905262

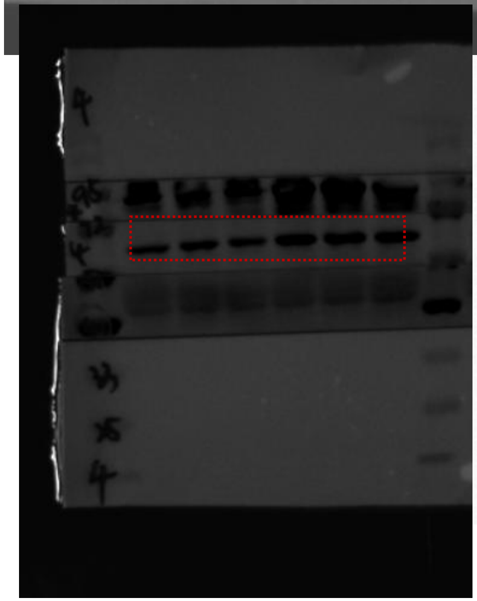

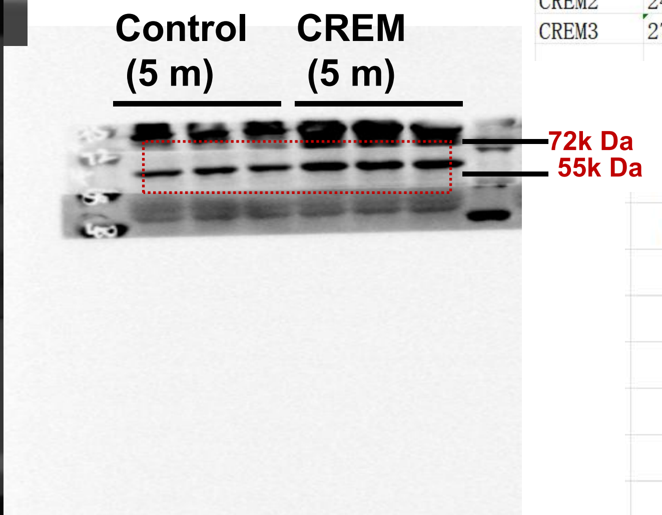

PFKM/β-actin	Control	CREM(5m)
	0.957681526	1.037570139
	0.942500714	1.172456383
	1.09981776	1.156840887
	1.039042936	1.423090729
	0.936806359	1.369424251
	1.024150705	1.36905262

Fig1I：CREM mice （5month）

PFKM：85k Da

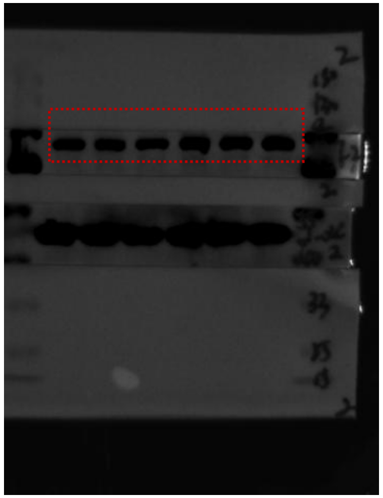

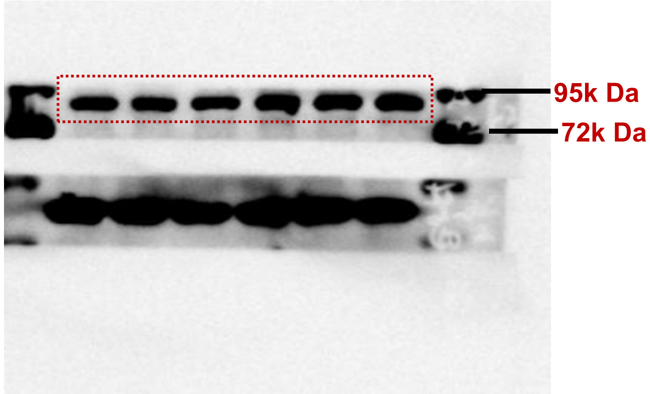

	PFKM	β-actin				
C1	11.982756	61.175144213		0.677276481	0.754776046	0.897321113
C2	51.468037	55.740115370		0.768243128		1.017842489
C3	21.710678	10.518289963		0.818808527		1.084836399
CREM1	55.346717	70.589357775		0.969131444		1.183587386
CREM2	75.417784	92.568542495		0.881645871		1.301751788
CREM3	26.225396	56.831998462		1.016161181		1.24102418

Fig1I：CREM mice （5month）

LDHA：37k Da

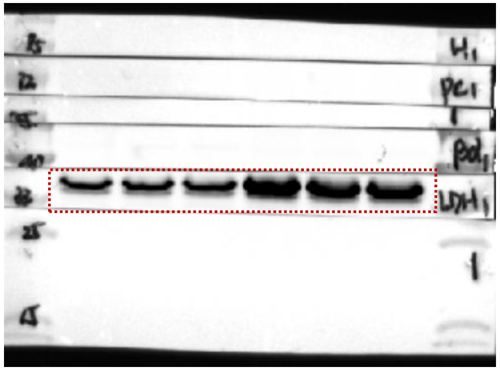

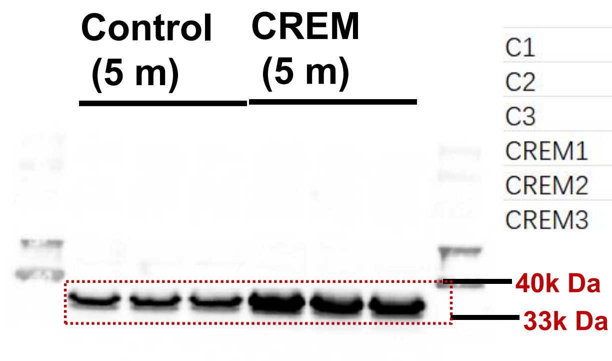

	LDH	BA	**		
C1	109802	160101	0.68583	0.7368	0.930821
C2	115090	156766	0.734152		0.996405
C3	128086	162048	0.79042		1.072774
CREM1	272185	187323	1.453025		2.118639
CREM2	299692	169037	1.772937		2.243032
CREM3	292569	180078	1.624679		2.213003

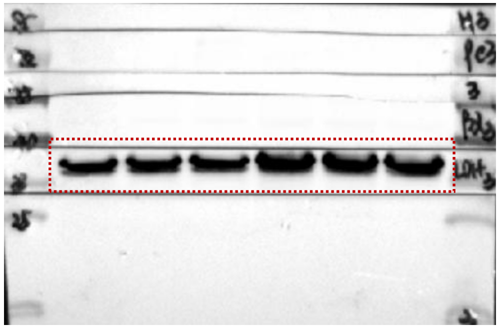

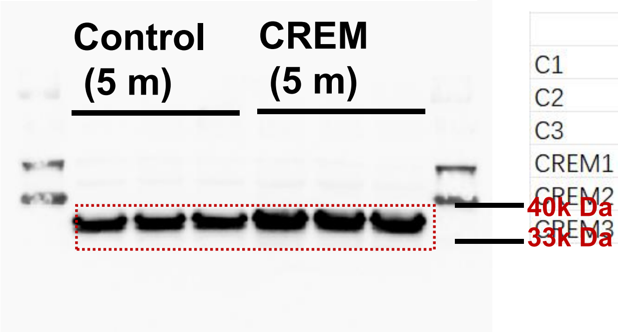

	LDH	BA			
C1	171799	122338	1.404298	1.454927	0.965201
C2	179560	123233	1.457077		1.001478
C3	182709	121530	1.503407		1.033321
CREM1	236263	120695	1.957521		1.343457
CREM2	211172	115735	1.824617		1.299309
CREM3	215535	116834	1.844797		1.227078

LDHA/b-a	con	crem
	0.930821	2.118639
	0.996405	2.243032
	1.072774	2.213003
	0.965201	1.343457
	1.001478	1.299309
	1.033321	1.227078

Figure 2B：CREM mice （3month）(n = 6)

HK：102k Da

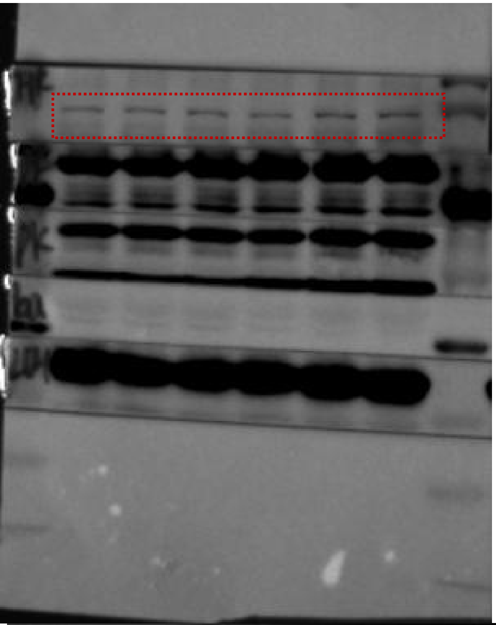

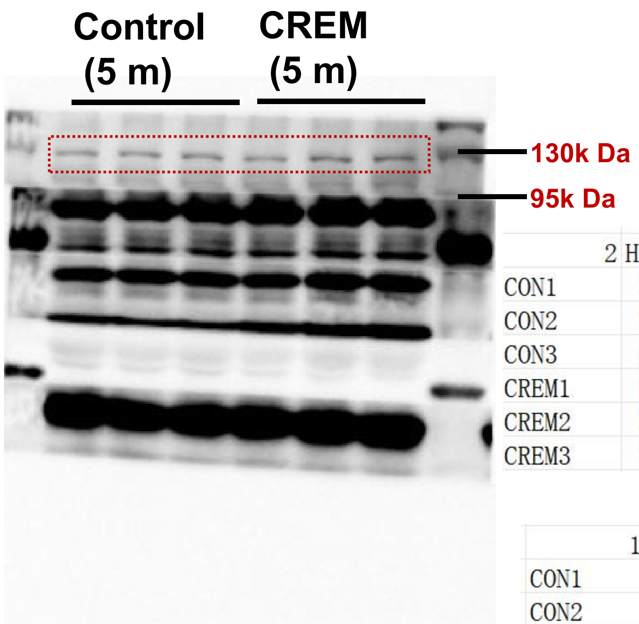

	2 HK	ba			
CON1	11996.761	25060.861	0.478705061	0.524689621	0.912358549
CON2	12135.246	21701.104	0.559199477		1.065771943
CON3	12115.761	22597.104	0.536164324		1.021869508
CREM1	11908.832	18779.761	0.63413118		1.133998164
CREM2	15457.489	24215.175	0.638338934		1.190565849
CREM3	16081.589	25818.125	0.622879818		1.301176587

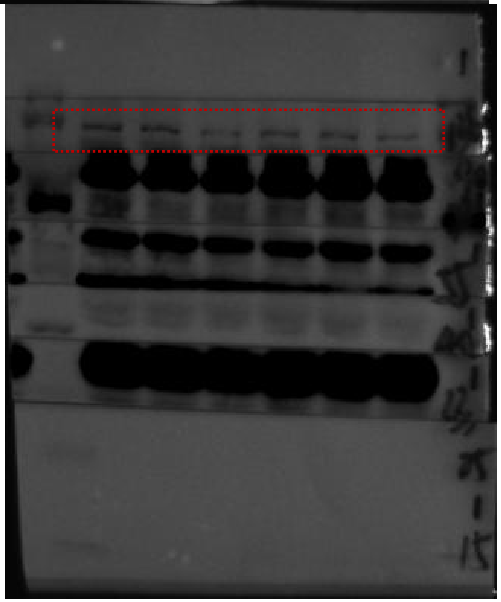

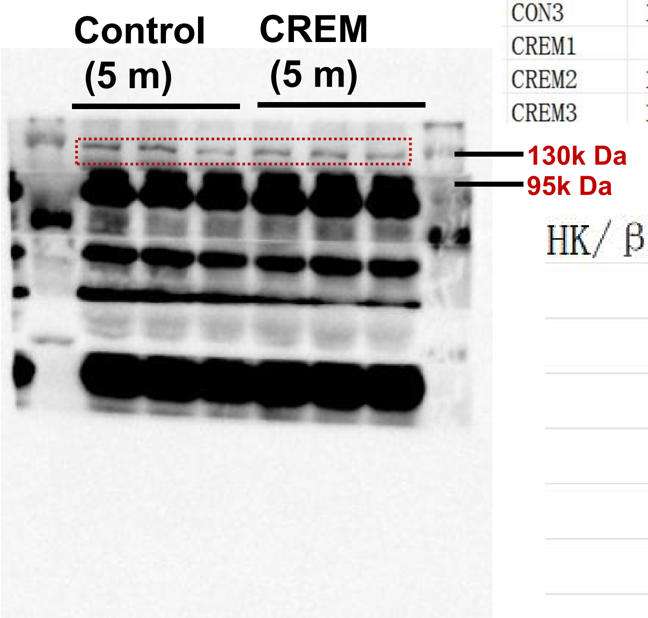

	1 HK	bactin			
CON1	23428.711	24298.95	0.964186029	0.817425763	1.179539565
CON2	22163.66	22919	0.967042896		1.183034521
CON3	10451.953	20059.47	0.521048365		0.637425915
CREM1	14322.66	25194.37	0.568486576		0.589602586
CREM2	13716.125	21120.83	0.649412154		0.67154431
CREM3	11349.933	20002.71	0.567419736		1.08899629

HK/β-actin	Con	Crem
	1.17954	0.589603
	1.183035	0.671544
	0.637426	1.088996
	0.912359	1.133998
	1.065772	1.190566
	1.02187	1.301177

Figure 2B：CREM mice （3month）(n = 6)

PFKM：85k Da

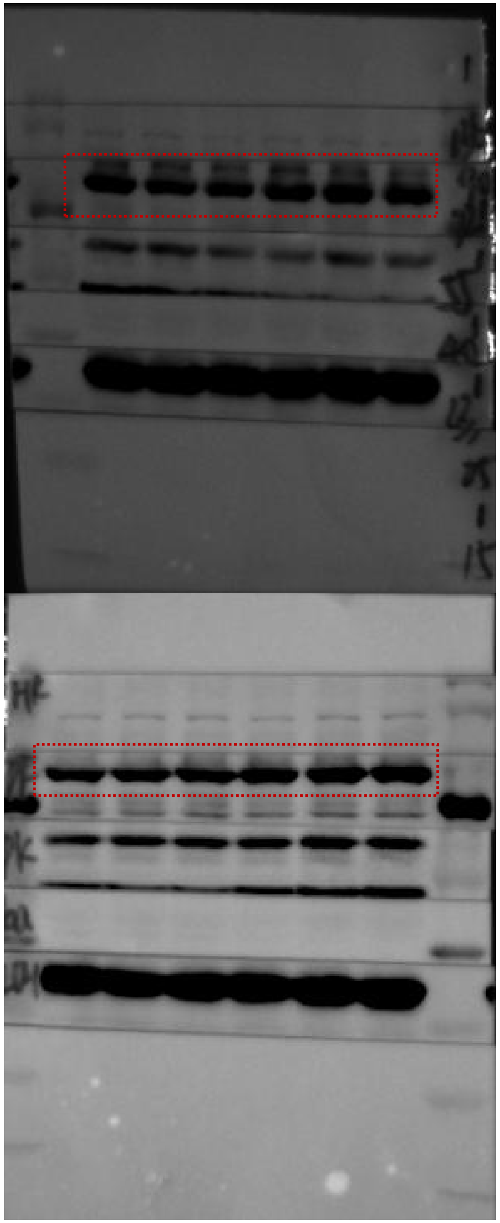

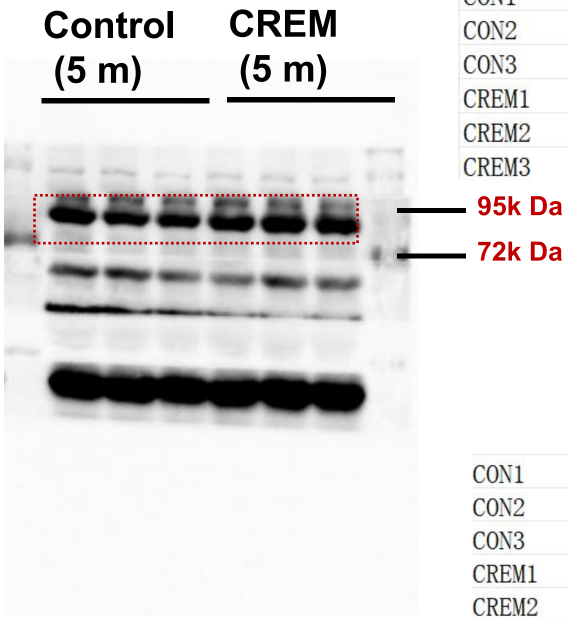

	PFKM	bactin			
CON1	21346.276	24298.95	0.878485423	0.884784441	0.992880731
CON2	19303.054	22919	0.842229182		0.951903247
CON3	18728.296	20059.47	0.933638719		1.055216022
CREM1	23863.246	25194.37	0.94716589		1.124594006
CREM2	26171.711	21120.83	1.239142047		1.32721793
CREM3	24237.711	20002.71	1.211721301		1.379330003

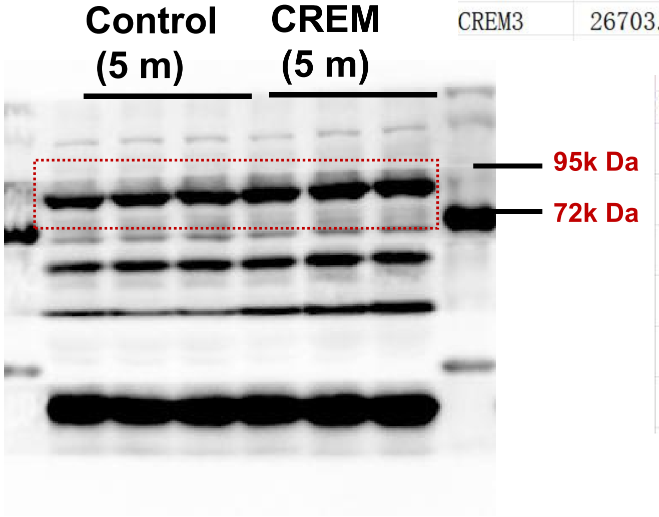

	PFKM	ba			
CON1	23283.832	25060.861	0.929091463	1.002880275	0.926423109
CON2	22600.882	21701.104	1.041462315		1.038471232
CON3	23457.761	22597.104	1.038087049		1.035105659
CREM1	25671.418	18779.761	1.366972562		1.312551153
CREM2	26851.589	24215.175	1.108874456		1.068190242
CREM3	26703.589	25818.125	1.034296216		1.113234012

PFKM/β-actin	Con	Crem
	0.9928807	1.124594
	0.9519032	1.3272179
	1.055216	1.37933
	0.9264231	1.3125512
	1.0384712	1.0681902
	1.0351057	1.113234

Figure 2B：CREM mice （3month）(n = 6)

PKM：60k Da

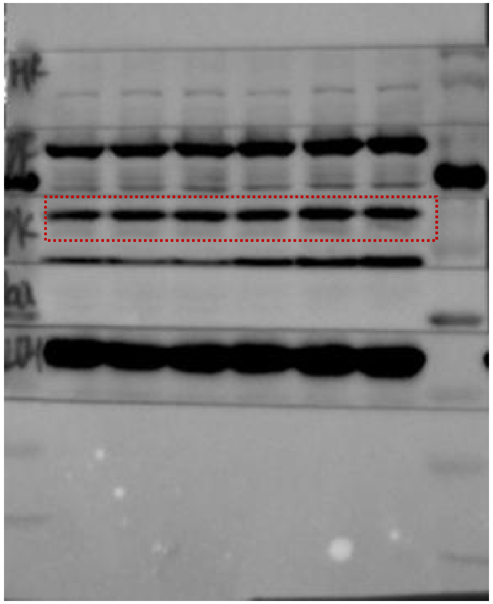

Control (5 m)　　CREM (5 m)

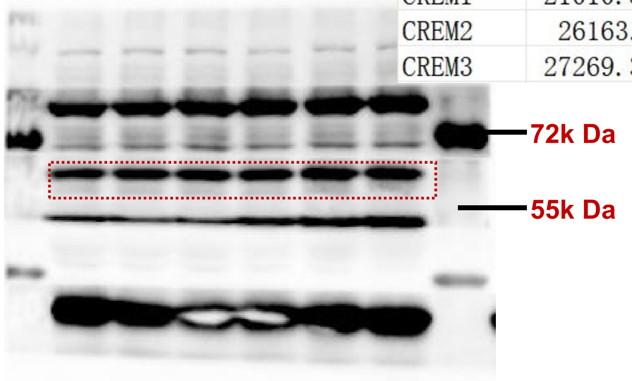

	PKM	ba			
CON1	24005. 175	25060. 861	0. 957875111	1. 046374924	0. 915422463
CON2	24821. 569	21701. 104	1. 143792915		1. 093100464
CON3	23443. 518	22597. 104	1. 037456747		0. 991477073
CREM1	21010. 861	18779. 761	1. 118803429		0. 978152089
CREM2	26163. 64	24215. 175	1. 080464626		1. 041455106
CREM3	27269. 347	25818. 125	1. 056209427		1. 102658807

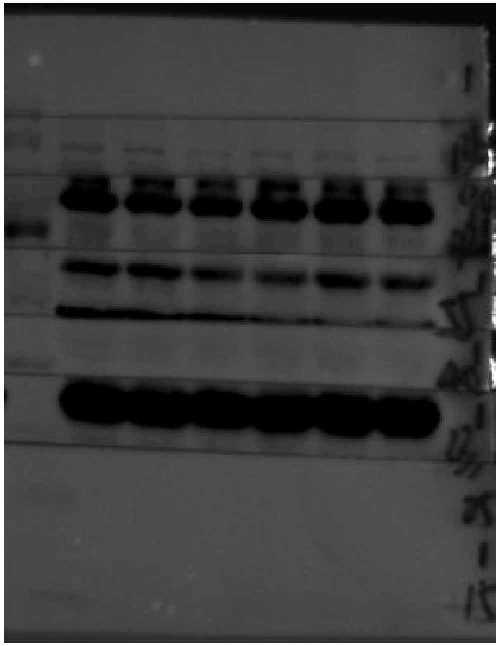

Control (5 m)　　CREM (5 m)

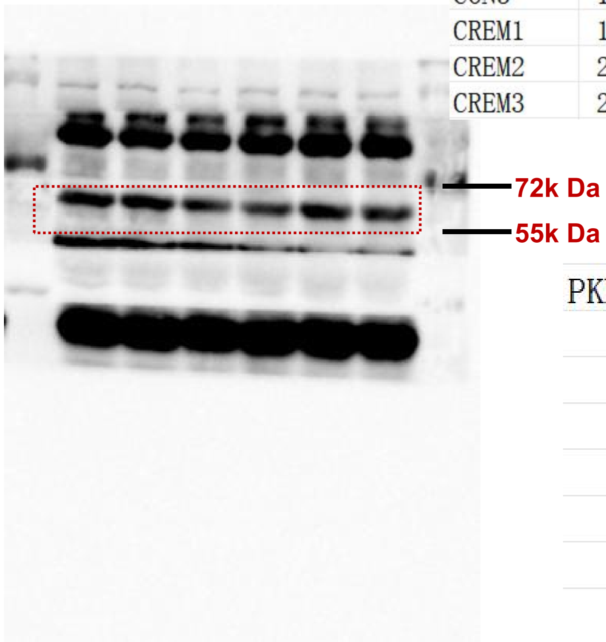

	PKM	ba			
CON1	25729. 296	24298. 95	[illegible]8864388	1. 018239067	1. 039897625
CON2	25365. 246	22919	1. 106734219		1. 086909995
CON3	17835. 246	20059. 47	0. 889118595		0. 87319238
CREM1	18200. 104	25194. 37	0. 722387797		0. 812476312
CREM2	24755. 368	21120. 83	1. 172082994		1. 059046493
CREM3	20235. 761	20002. 71	1. 011650921		0. 955411224

PKM/β-actin	Con	Crem
	1. 039897625	0. 812476312
	1. 086909995	1. 059046493
	0. 87319238	0. 955411224
	0. 915422463	0. 978152089
	1. 093100464	1. 041455106
	0. 991477073	1. 102658807

Figure 2B：CREM mice （3month）(n = 6)

LDHA：37k Da

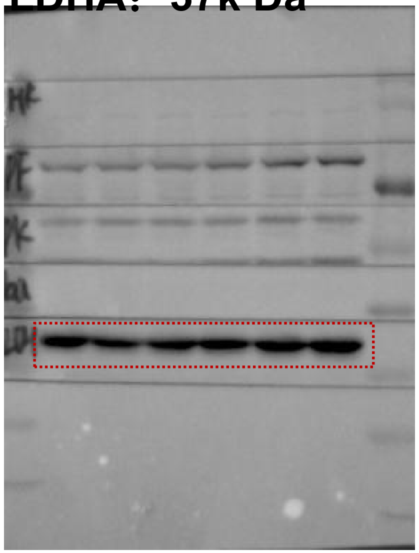

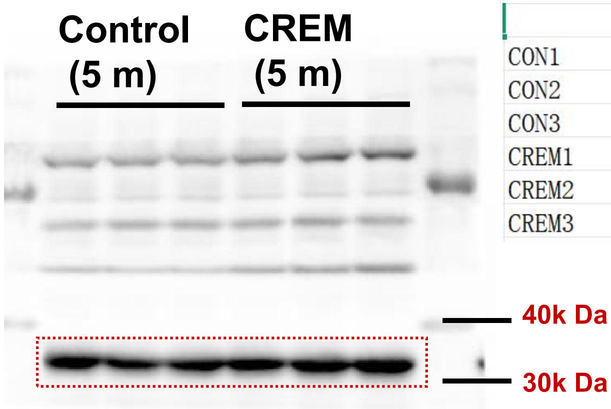

	LDHA	ba			
CON1	29587.347	25060.861	1.180619732	1.192039064	0.990420338
CON2	26364.933	21701.104	1.214912062		1.019188128
CON3	26677.811	22597.104	1.180585397		0.990391534
CREM1	27922.397	18779.761	1.486834524		1.223820695
CREM2	29203.154	24215.175	1.205985668		1.021485272
CREM3	28329.933	25818.125	1.097288552		0.929444456

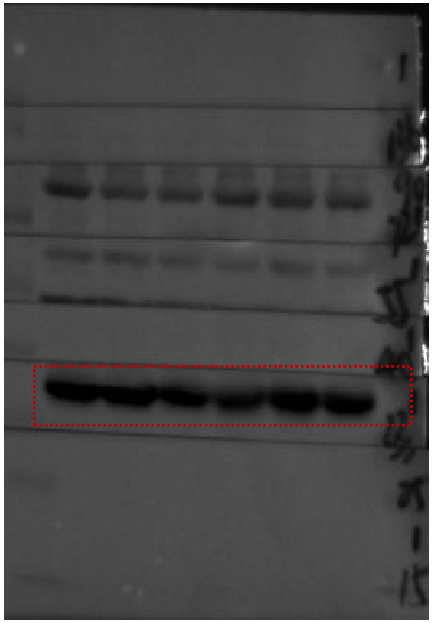

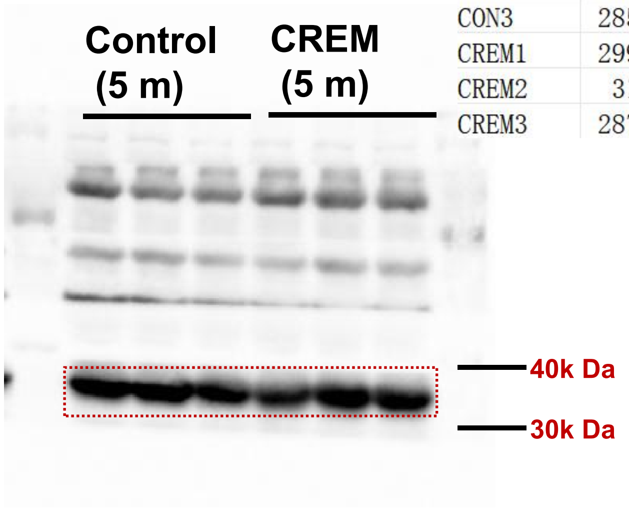

	LDH	bactin			
CON1	32335.882	24298.95	1.33075207	1.355321723	0.981871719
CON2	30013.79	22919	1.309559089		0.966234855
CON3	28597.861	20059.47	1.42565401		1.051893426
CREM1	29934.083	25194.37	1.188125973		0.907271755
CREM2	31126.79	21120.83	1.473748288		1.033734887
CREM3	28797.326	20002.71	1.439671153		1.081847765

LDHA/β-actin	Con	Crem
	0.981871719	0.907271755
	0.966234855	1.033734887
	1.051893426	1.081847765
	0.990420338	1.223820695
	1.019188128	1.021485272
	0.990391534	0.929444456

Fig2H：CREM mice AAV-PFKM (n = 6)

PFKM：85k Da

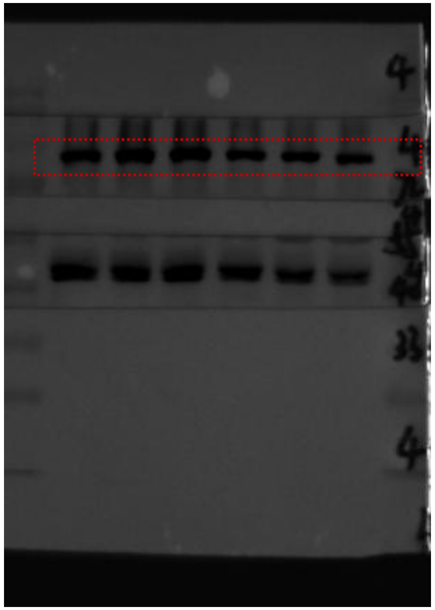

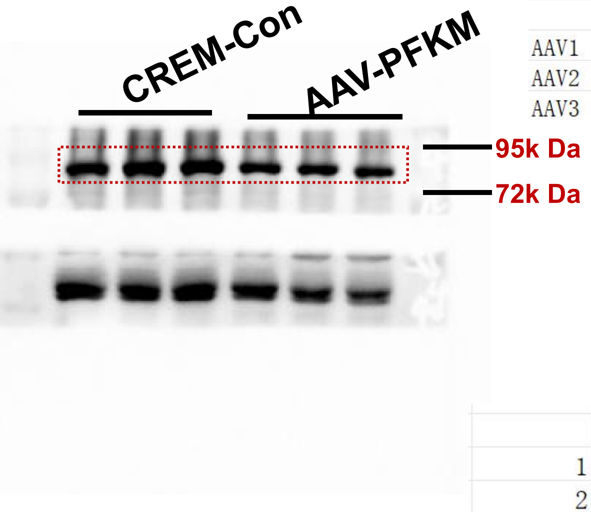

	PFKM	BA			
1	19472.539	25095.246	0.775945332	0.878674825	0.883085881
2	22078.246	22307.104	0.989740578		1.126401428
3	22018.953	25299.296	0.870338566		0.990512691
AAV1	14625.418	22602.681	0.647065629		0.833906207
AAV2	14120.832	20421.125	0.691481591		0.698649329
AAV3	12652.004	19036.518	0.664617552		0.763631049

	PFKM	BA			
1	17131.711	9350.154	1.832238378	1.248271998	1.46781981[illegible]
2	19774.953	19990.418	0.989221586		0.79247278[illegible]
3	22475.953	24341.589	0.923356031		0.73970739[illegible]
AAV1	19379.832	26500.468	0.7313015		0.39913010[illegible]
AAV2	18114.711	27436.225	0.660247939		0.66744190[illegible]
AAV3	16593.811	27214.054	0.609751528		0.66036448[illegible]

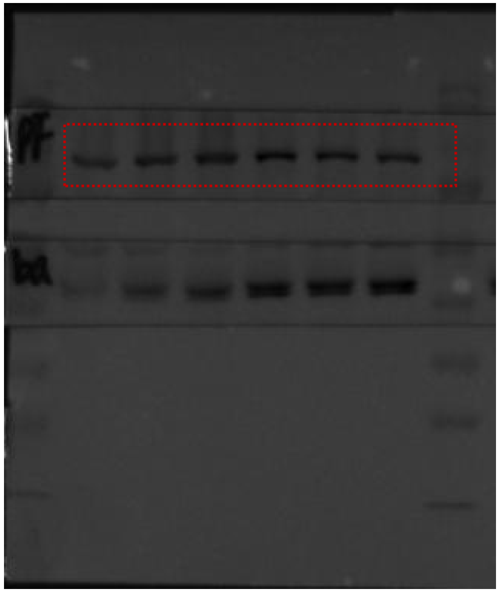

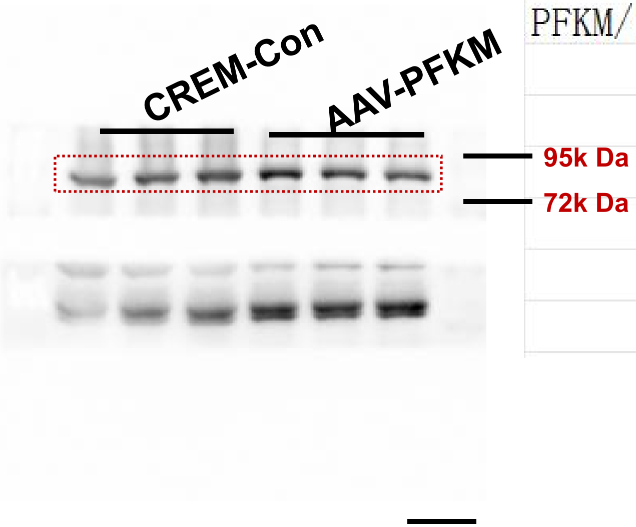

PFKM/β-actin	CREM	CREM+AAV-PFKM
	1.46782	0.399130107
	0.792473	0.667441904
	0.739707	0.660364483
	0.883086	0.833906207
	1.126401	0.698649329
	0.990513	0.763631049

Fig2P：CREM mice AAV-PFKM (n = 6)

Col3：225k Da

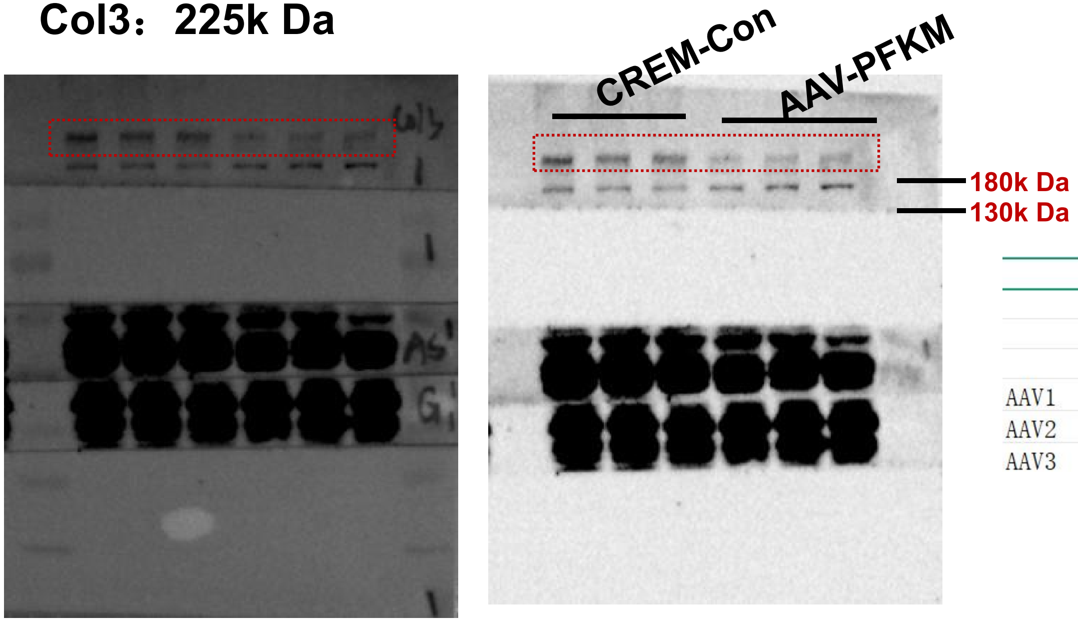

	col3	gap			
1	20563.51828	17391.07463914	1.182417919	1.270074771	0.93098292
2	18292.90306	16117.39696962	1.13497875		0.893631442
3	19252.61017	12896.74011537	1.492827645		1.175385638
AAV1	12758.71067	18078.98275605	0.705720607		0.472740848
AAV2	12583.24621	19224.46803743	0.654543272		0.576700905
AAV3	13903.05382	20607.17514421	0.674670532		0.570585511

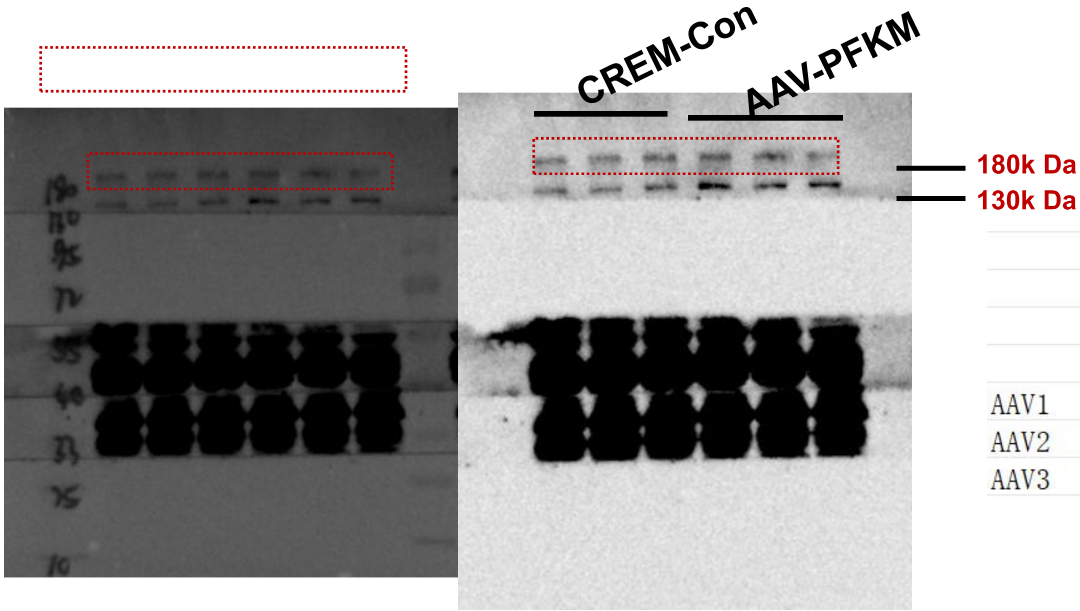

	col3	gap			
1	15714.711	17776.589	0.884011607	0.88081	1.00363
2	15354.418	17957.711	0.855032025		0.97073
3	15023.368	16629.882	0.903395947		1.02564
AAV1	16653.711	19113.175	0.871321013		1.01905
AAV2	16715.468	18482.246	0.904406748		1.00112
AAV3	12084.447	21232.004	0.569161865		0.64384

Fig2P：CREM mice AAV-PFKM（n = 6)

α-SMA：42k Da

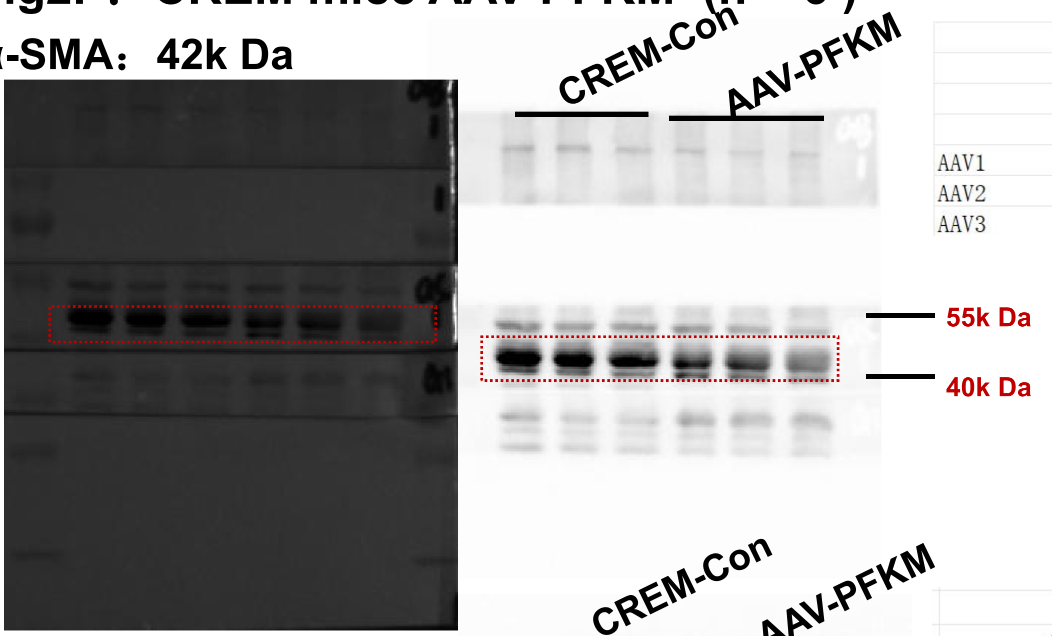

	asma	gap			
1	26500.811	17776.589	1.490770305	1.838571252	0.810830857
2	23082.64	10957.711	2.106520239		1.145737614
3	26147.882	13629.882	1.918423212		1.043431528
AAV1	19181.175	19313.175	0.993165287		0.517698744
AAV2	20225.882	18182.246	1.112397335		0.528073414
AAV3	14172.447	17232.004	0.822449148		0.551694077

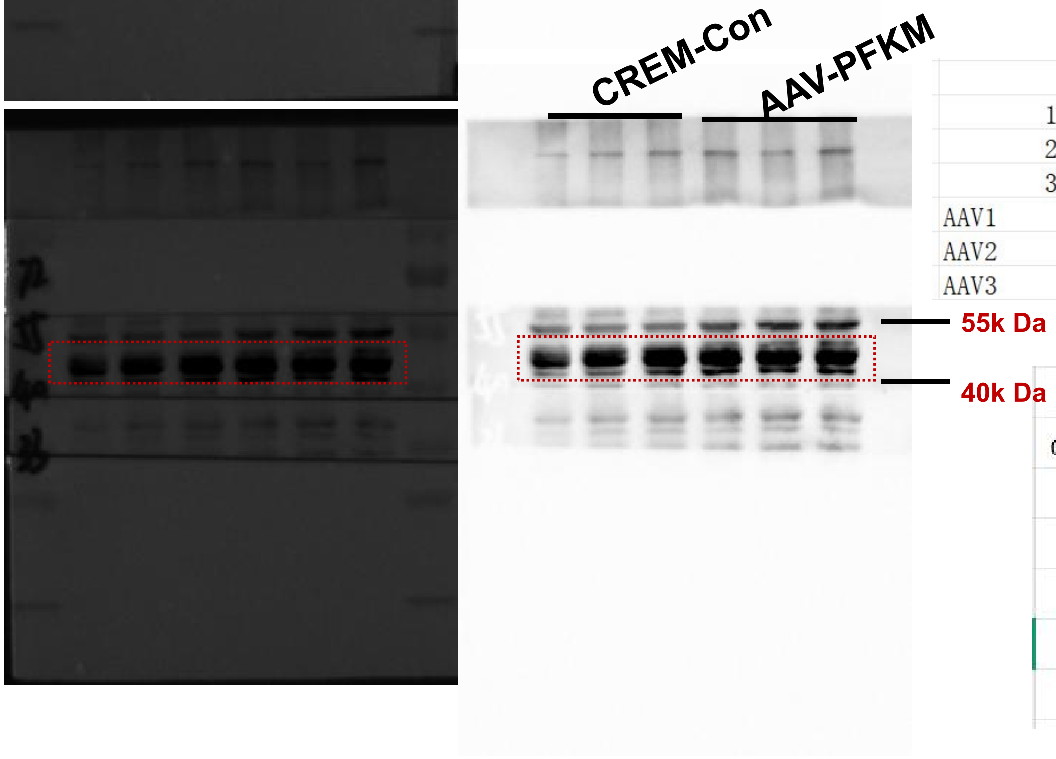

	asma	gap			
1	14005.76	7798.569	1.795939871	1.530009339	1.173809744
2	19018.52	13837.368	1.374431756		0.898315926
3	23055.64	16240.296	1.41965639		0.92787433
AAV1	22255.47	16680.782	1.334198121		0.742896877
AAV2	23529.37	20930.368	1.124173641		0.81791885
AAV3	22469.64	17960.711	1.251044015		0.881230151

	crem	crem+aav-pfkm
α-SMA/gap	0.810830857	0.517698744
	1.145737614	0.528073414
	1.043431528	0.551694077
	1.173809744	0.742896877
	0.898315926	0.81791885
	0.92787433	0.881230151

Fig2P：CREM mice AAV-PFKM （n = 6 ）

TGF-β1：55k Da

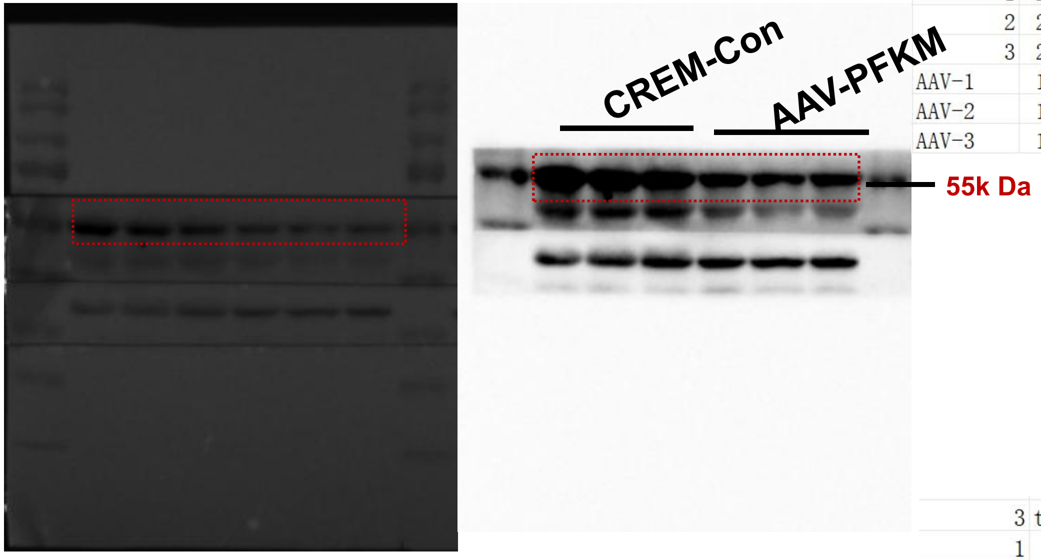

	tgf-β1	gap			
1	25516.00357	18794.832	1.35760743	1.082042022	1.254671633
2	23964.46804	22498.58936	1.065154248		0.984392682
3	21926.29646	26630.12489	0.823364387		0.760935685
AAV-1	11923.10408	23487.63961	0.507633133		0.373917468
AAV-2	10903.90307	25047.9325	0.435321481		0.408693371
AAV-3	10629.76093	24664.81118	0.430968672		0.52342399

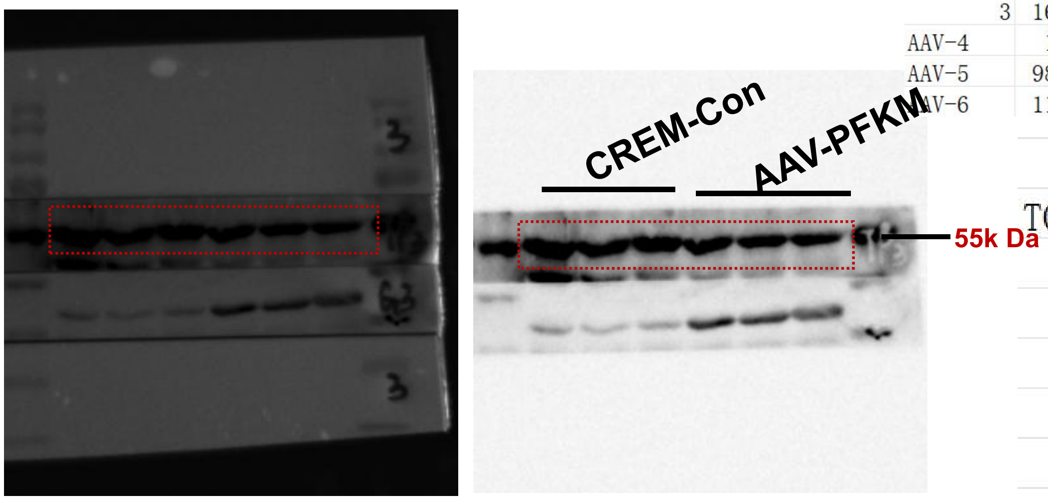

3	tgf-β1	gap			
1	27910.71068	21068.53911	1.32475776	1.349151638	0.981919099
2	24215.00357	15587.71068	1.553467605		1.151440329
3	16517.36753	14126.71068	1.169229547		0.866640572
AAV-4	11937.9325	20296.832	0.588167282		0.378615737
AAV-5	9892.811183	17355.63961	0.570005566		0.430271543
[illegible]AV-6	11607.00357	21504.66043	0.539743634		0.461623327

	CREM	CREM+AAV-PFKM
TGF-β1/gap	0.981919099	0.378615737
	1.151440329	0.430271543
	0.866640572	0.461623327
	1.254671633	0.373917468
	0.984392682	0.408693371
	0.760935685	0.52342399

Figure 3B: OE-HK (n = 6)

HK:102k Da

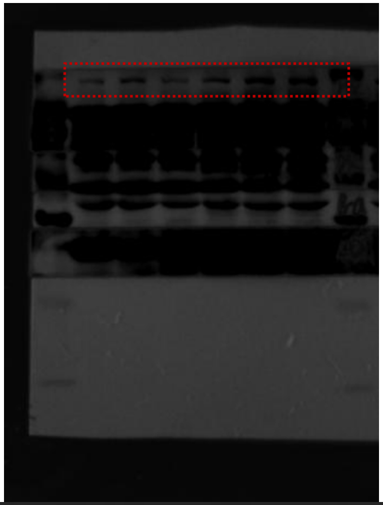

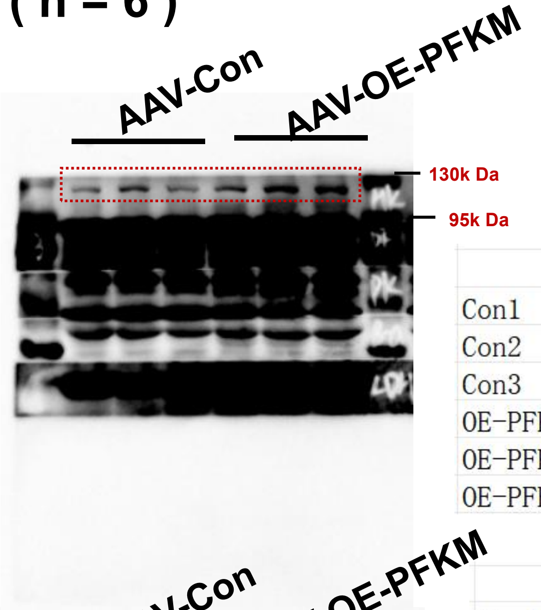

	HK	β-actin			
Con1	8047.69	19222.074	0.418669161	0.637046669	0.657203281
Con2	12696.559	18060.074	0.703018131		1.103558288
Con3	10908.660	13818.003	0.789452714		1.239238431
OE-PFKM1	13714.296	19236.831	0.712918659		1.702821046
OE-PFKM2	20574.317	21542.195	0.955070566		1.209788184
OE-PFKM3	21604.296	24199.589	0.892754672		1.269888546

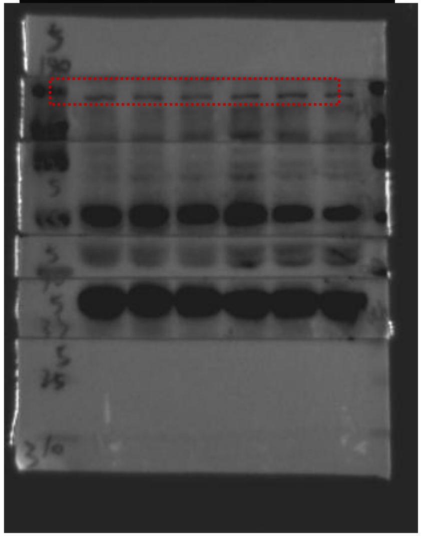

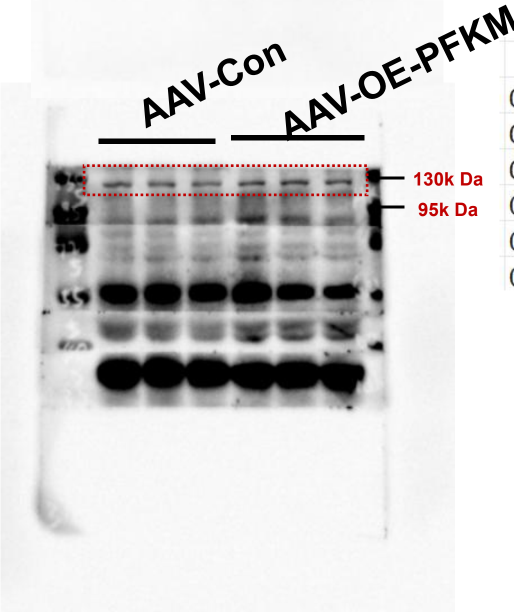

	HK	β-actin			
Con4	18649.053	19640.953	0.949498404	0.952157105	0.997207707
Con5	15555.882	16468.831	0.944564997		0.992026413
Con6	14747.660	15323.710	0.962407914		1.01076588
OE-PFKM4	22715.660	21608.074	1.051257958		1.112954599
OE-PFKM5	26127.660	19398.417	1.346896469		1.399506852
OE-PFKM6	22587.932	20820.275	1.084900742		1.142604072

	Con	AAV-OE-PFKM
HK/β-act	0.657203281	1.702821046
	1.103558288	1.209788184
	1.239238431	1.269888546
	0.997207707	1.112954599
	0.992026413	1.399506852
	1.01076588	1.142604072

Figure 3B

PFKM：85k Da

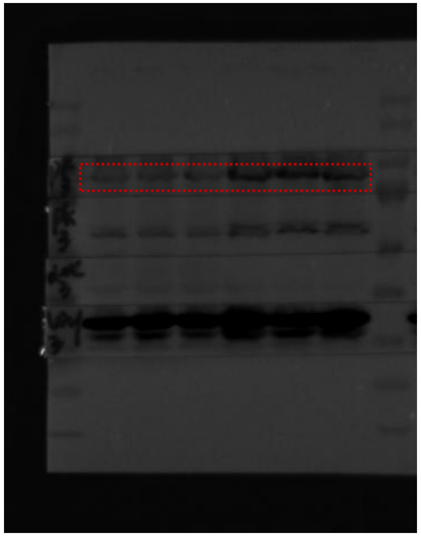

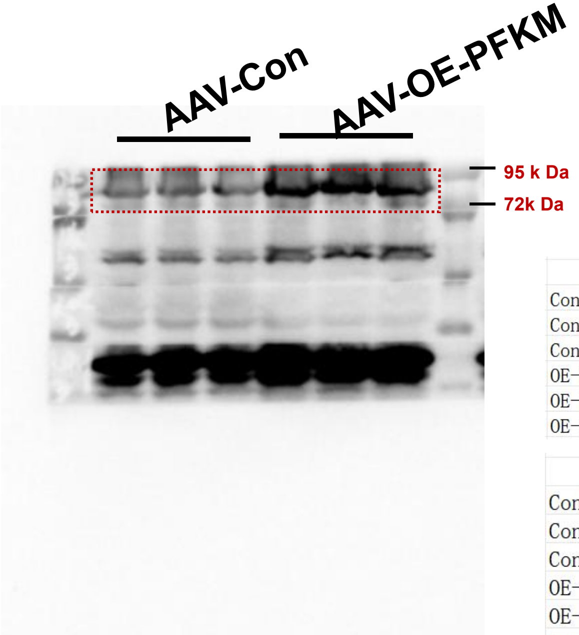

	PFKM	β-actin				
Con1	13225.346	23907.831998462		0.553180511	0.652683344	0.847548073
Con2	14502.589	22711.225396744		0.638564811		0.978368479
Con3	11680.468	15242.589357775		0.766304711		1.174083448
OE-PFKM1	28421.417	24293.074639149		1.169939096		1.526728309
OE-PFKM2	27315.417	27087.953318806		1.008397256		1.579161955
OE-PFKM3	26886.417	21551.882250994		1.247520633		2.255178208

	PFKM	b-a				
Con4	9986.861	21981.053823869		0.45433952	0.488604409	0.929871919
Con5	9814.983	24016.296464556		0.408680113		0.836423302
Con6	14889.296	24700.488852712		0.602793595		1.233704779
OE-PFKM4	25844.246	23096.124891681		1.118986251		1.856334011
OE-PFKM5	28173.346	26012.831998462		1.083055729		2.383802601
OE-PFKM6	23334.296	25617.831998462		0.910861484		2.228788371

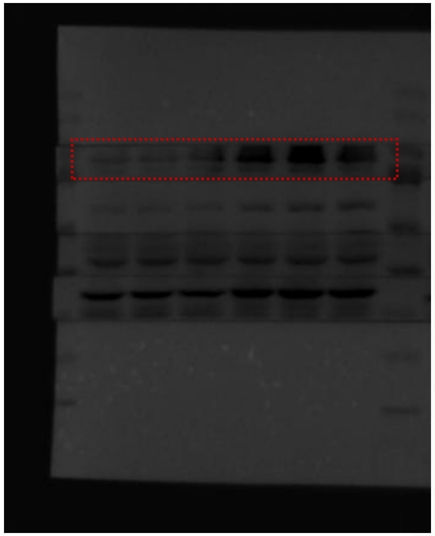

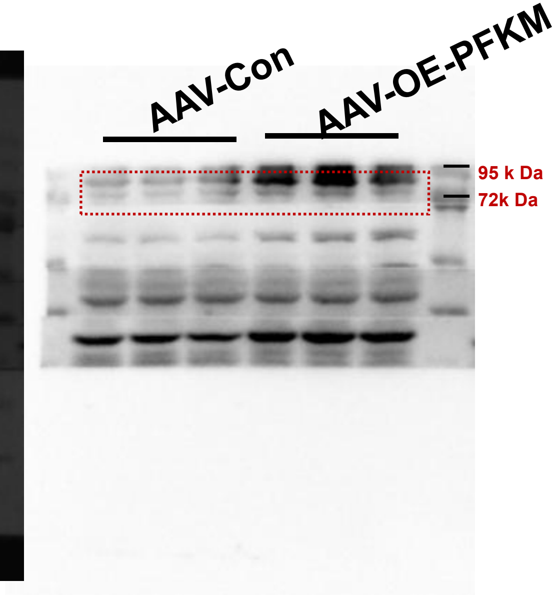

	Con	OE-PFKM
PFKM/β-actin	0.847548073	1.526728309
	0.978368479	1.579161955
	1.174083448	2.255178208
	0.929871919	1.856334011
	0.836423302	2.383802601
	1.233704779	2.228788371

Figure 3B

PKM：60k Da

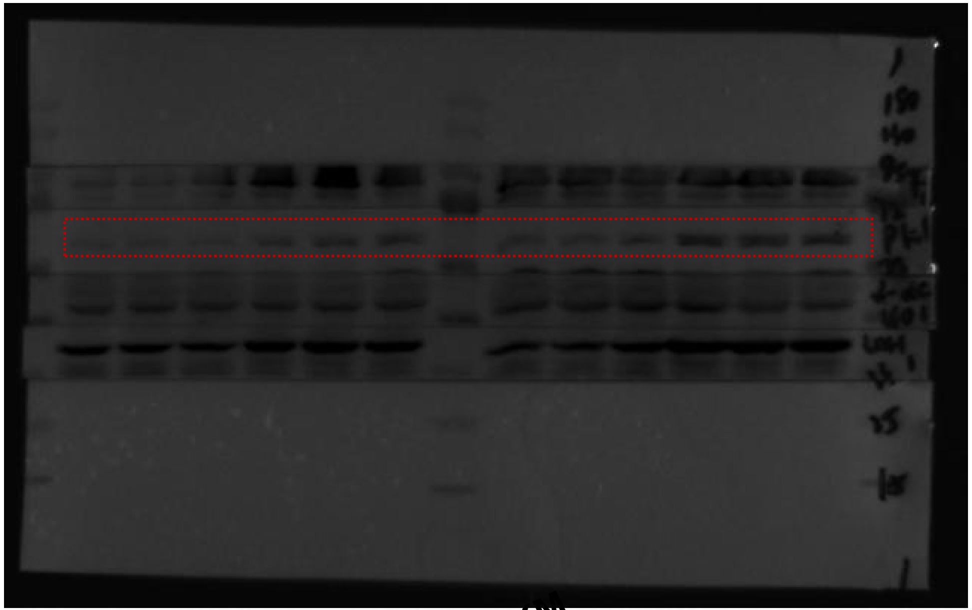

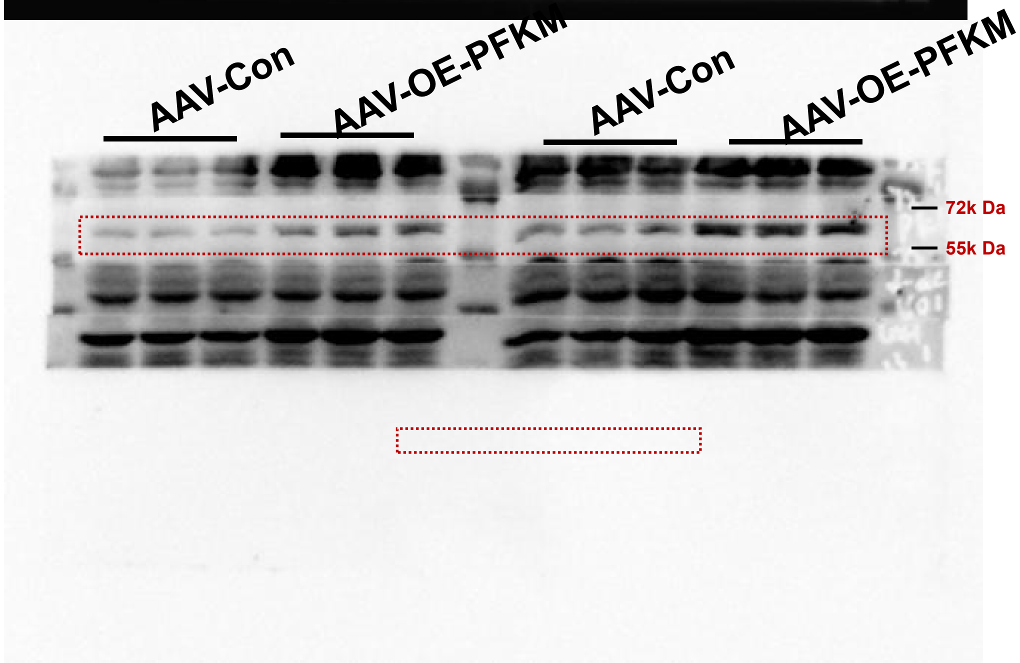

	PKM	β-actin				
Con1	7571. 518	21981. 053823869		0. 344456565	0. 30587279	1. 126143208
Con2	6928. 004	24016. 296464556		0. 288470938		0. 943107553
Con3	7032. 004	24700. 488852712		0. 284690866		0. 930749239
OE-PFKM1	12683. 831	23096. 124891681		0. 549175763		1. 929024874
OE-PFKM2	18563. 003	26012. 831998462		0. 713609482		2. 473765596
OE-PFKM3	19737. 124	25617. 831998462		0. 770444778		2. 236696458

	PKM	β-actin				
Con4	10265. 396969	25269. 246212025		0. 406240728	0. 394013797	1. 031031733
Con5	7953. 39697	21829. 175144213		0. 364347114		0. 924706488
Con6	10449. 296464	25396. 053823869		0. 411453549		1. 044261779
OE-PFKM4	19419. 831998	27389. 539105243		0. 709023687		1. 946011535
OE-PFKM5	18786. 074639	22884. 468037432		0. 820909387		2. 020746152
OE-PFKM6	20650. 589357	20803. 639610307		0. 992643102		2. 412527746

	Con	OE-PFKM
PKM/β-actin	1. 126143208	1. 929024874
	0. 943107553	2. 473765596
	0. 930749239	2. 236696458
	1. 031031733	1. 946011535
	0. 924706488	2. 020746152
	1. 044261779	2. 412527746

Figure 3B

LDHA：37k Da

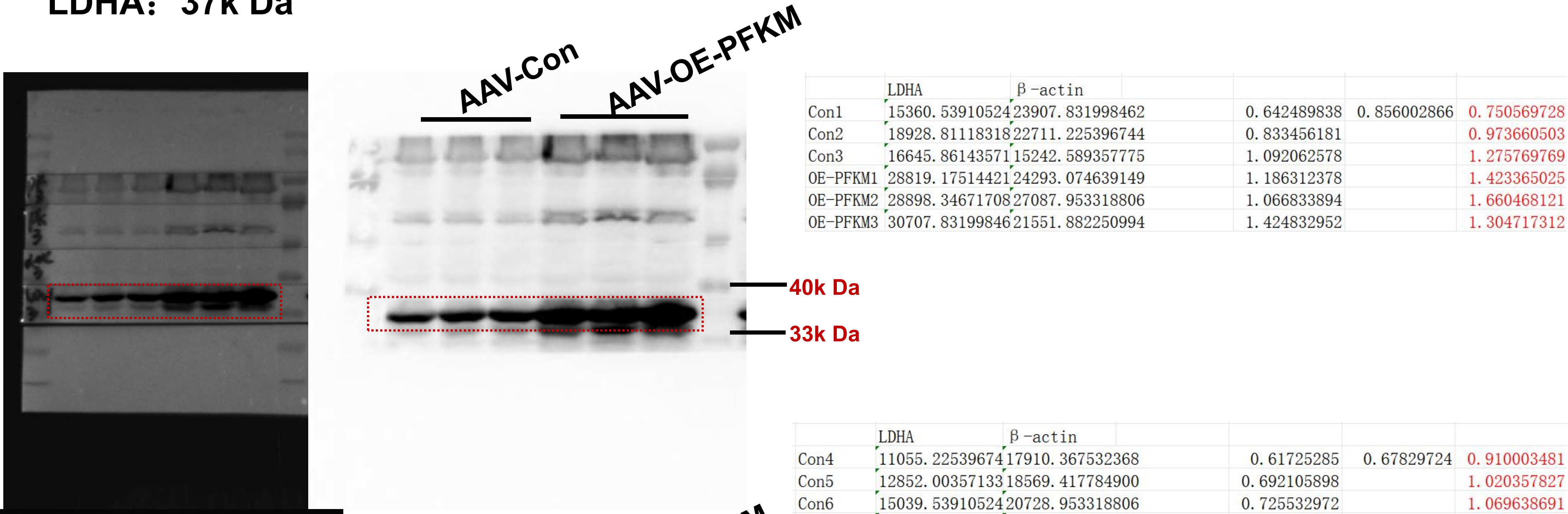

	LDHA	β-actin				
Con1	15360. 53910524	23907. 831998462		0. 642489838	0. 856002866	0. 750569728
Con2	18928. 81118318	22711. 225396744		0. 833456181		0. 973660503
Con3	16645. 86143571	15242. 589357775		1. 092062578		1. 275769769
OE-PFKM1	28819. 17514421	24293. 074639149		1. 186312378		1. 423365025
OE-PFKM2	28898. 34671708	27087. 953318806		1. 066833894		1. 660468121
OE-PFKM3	30707. 83199846	21551. 882250994		1. 424832952		1. 304717312

	LDHA	β-actin				
Con4	11055. 22539674	17910. 367532368		0. 61725285	0. 67829724	0. 910003481
Con5	12852. 00357133	18569. 417784900		0. 692105898		1. 020357827
Con6	15039. 53910524	20728. 953318806		0. 725532972		1. 069638691
OE-PFKM4	27328. 12489168	24002. 953318806		1. 138531769		1. 844514398
OE-PFKM5	27188. 71067811	17959. 589357775		1. 513882647		2. 086580081
OE-PFKM6	25112. 90306627	20957. 539105243		1. 198275377		1. 651579491

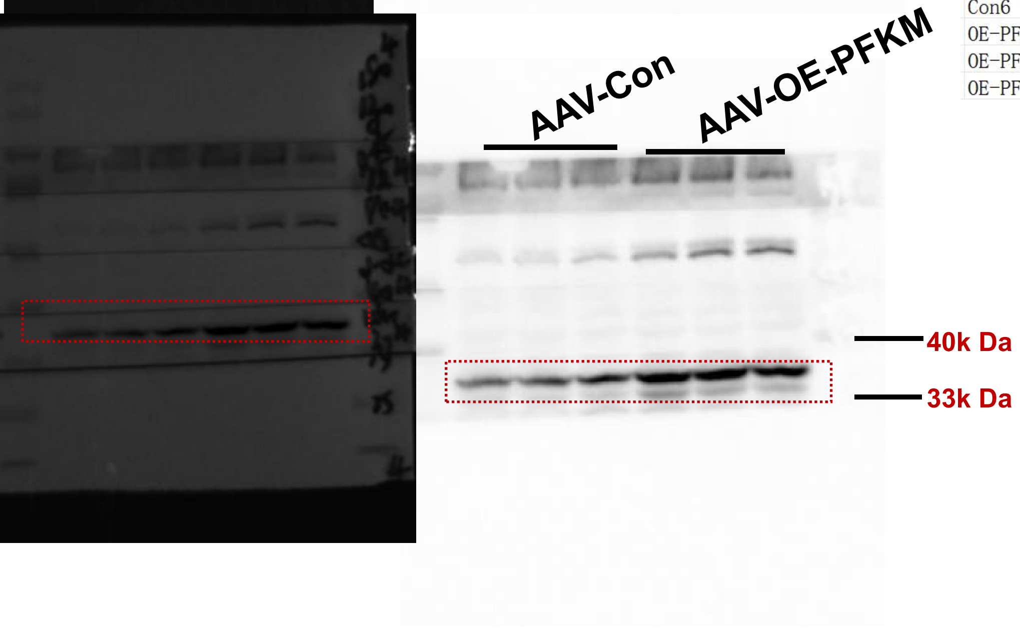

	Con	OE-PFKM
LDHA/β-acti	0. 750569728	1. 423365025
	0. 973660503	1. 660468121
	1. 275769769	1. 304717312
	0. 910003481	1. 844514398
	1. 020357827	2. 086580081
	1. 069638691	1. 651579491

Figure 3J (n = 6)

Col3：225k Da

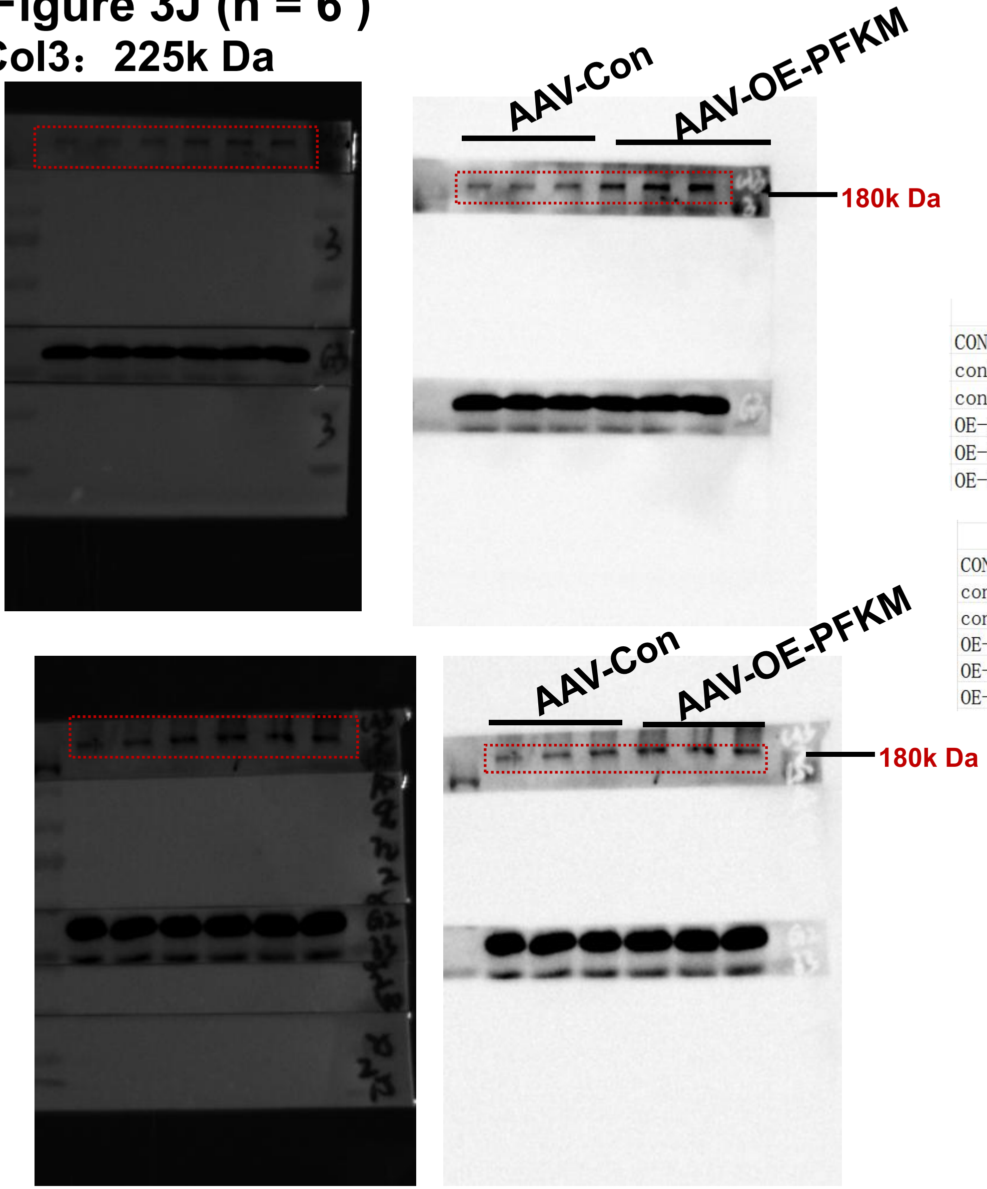

	Col3	gap				
CON1	9979. 861436	29505. 074639149		0. 338242203	0. 455974	0. 741802
con2	10857. 760930(	20724. 932503526		0. 523898494		1. 148966
con3	11212. 367532:	22168. 417784900		0. 505781136		1. 109233
OE-PFKM1	16347. 639610:	20124. 982756057		0. 81230577		2. 401551
OE-PFKM2	24595. 246212(	21353. 932503526		1. 151790014		2. 27725
OE-PFKM3	24318. 689862!	28927. 488852712		0. 840677529		1. 662137

	Col3	Gap				
CON1	8488. 811183	24330. 417784900		0. 348897058	0. 556004273	0. 627507872
con2	14387. 831998	25520. 275649276		0. 563780431		1. 013985788
con3	20314. 417784	26894. 568542495		0. 755335329		1. 35850634
OE-PFKM1	25148. 195959	23785. 396969620		1. 057295617		1. 399769845
OE-PFKM2	21927. 296464	24613. 689862838		0. 890857754		1. 580150188
OE-PFKM3	13712. 124891(	24292. 882250994		0. 564450309		1. 617813324

	Con-PFKM	Con-OE-PFKM
Col3/GAPDH	0. 627507872	1. 399769845
	1. 013985788	1. 580150188
	1. 35850634	1. 617813324
	0. 741801603	2. 401550615
	1. 148965858	2. 277249845
	1. 109232539	1. 662136979

Figure 3J (n = 6)

α-SMA：42k Da

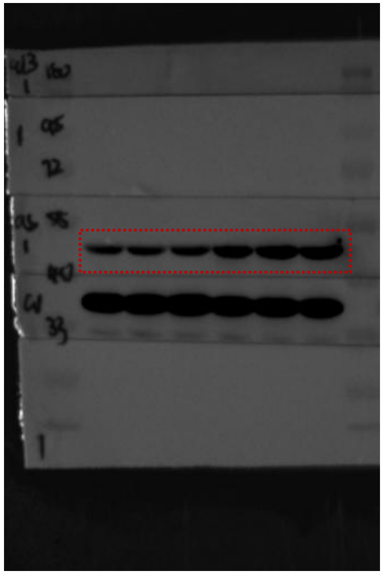

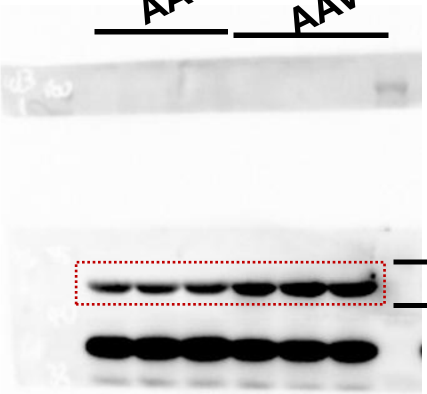

	asma	gap			
Con1	10424.932503526	29.589357775	0.391479281	0.394188293	0.993127619
Con2	11040.74012	52.275649276	0.394985376		1.002022087
Con3	12469.932503526	81.760930650	0.396100222		1.004850294
OE-PFKM1	19614.417784900	38.225396744	0.73083885		1.866864697
OE-PFKM2	24593.417784900	54.861435714	0.915790158		2.318541932
OE-PFKM3	24720.074639149	72.710678119	0.955449738		2.412141383

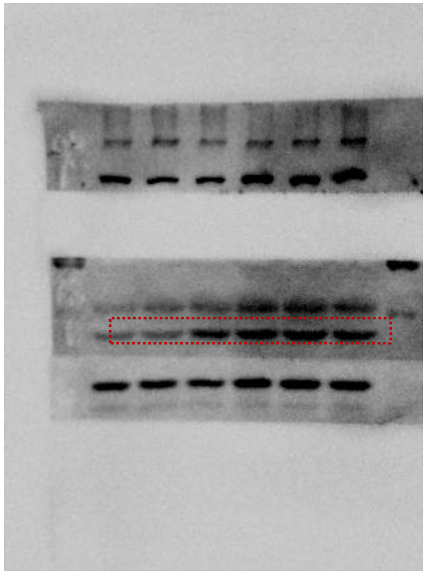

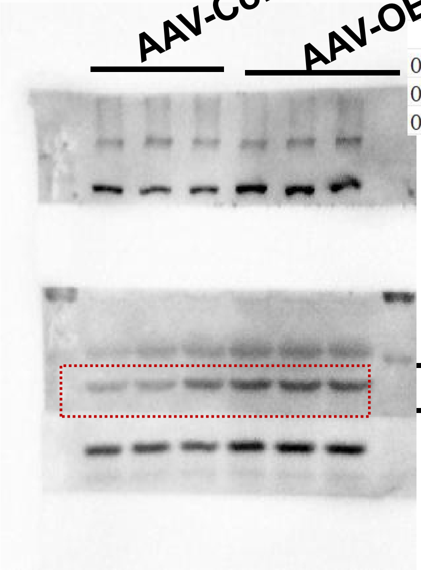

	[illegible]	gap			
[illegible]	12043.853426487	23005.053823869	0.523530765	0.504253166	1.038230001
Con2	8096.053824	21900.589357775	0.369672875		0.733109675
Con3	11865.811183182	19152.124891681	0.619555859		1.228660325
OE-PFKM1	22940.882250994	23683.882250994	0.968628454		2.620231347
OE-PFKM2	28233.296464556	25930.903066274	1.088789557		2.079705014
OE-PFKM3	29962.953318806	27475.831998462	1.090520328		1.760164661

	AAV-Con	AAV-OE-PFKM
α-SMA/GAPDH	1.038230001	2.620231347
	0.733109675	2.079705014
	1.228660325	1.760164661
	0.993127619	1.866864697
	1.002022087	2.318541932
	1.004850294	2.412141383

Figure 3J (n = 6)

TGF-β1：55k Da

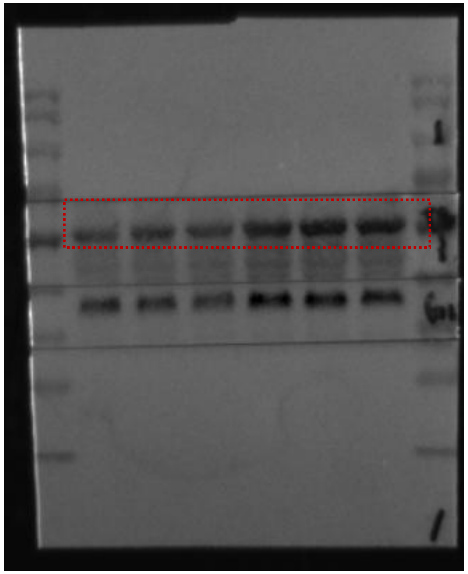

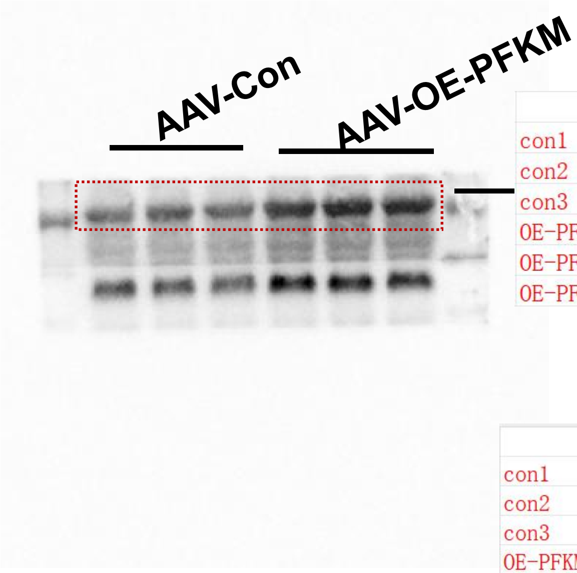

	TGF-β1	GAP			
con1	20900.7106	22835.4888527	0.915273188	0.694502047	1.317884
con2	15529.2462	23353.8319984	0.664954951		0.957456
con3	13634.4177	27091.2253967	0.503278002		0.72466
OE-PFKM1	27206.6604	22697.5182899	1.198662342		1.309622
OE-PFKM2	26856.2964	25962.9325035	1.034409209		1.555608
OE-PFKM3	26118.7817	29021.8822509	0.899968566		1.788214

	TGF-β1	GAP			
con1	16522.761	22184.125	0.744801113	0.812212728	0.917002513
con2	17928.154	21460.296	0.835410378		1.028561052
con3	19435.69	22693.933	0.856426693		1.054436435
OE-PFKM1	26228.004	26491.246	0.990063057		1.329298573
OE-PFKM2	27703.64	25059.711	1.105505167		1.323307917
OE-PFKM3	28934.761	22428.004	1.290117525		1.506395742

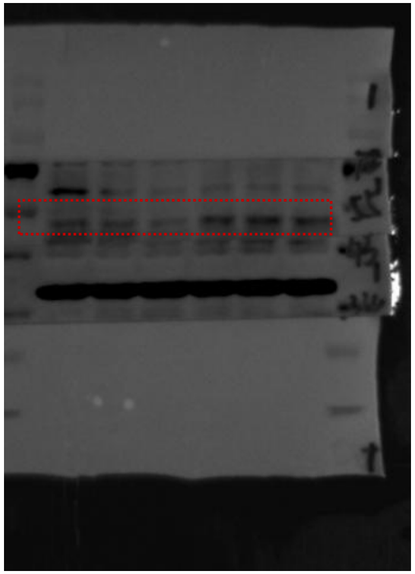

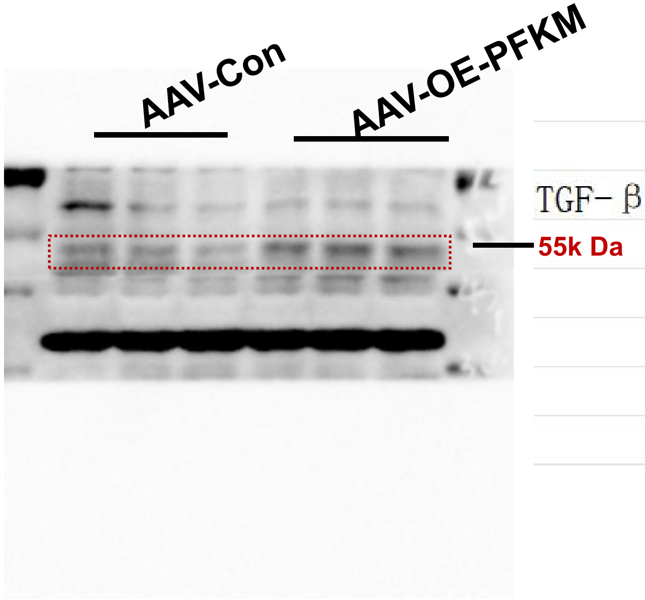

	Con	OE-PFKM
TGF-β1/GAPDH	0.917002513	1.329298573
	1.028561052	1.323307917
	1.054436435	1.506395742
	1.317884075	1.30962248
	0.957455711	1.555607951
	0.724660214	1.788213597

Fig4E：CM CM+LAC (n = 6)

Bax：26k Da

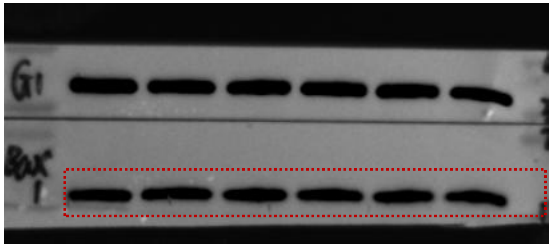

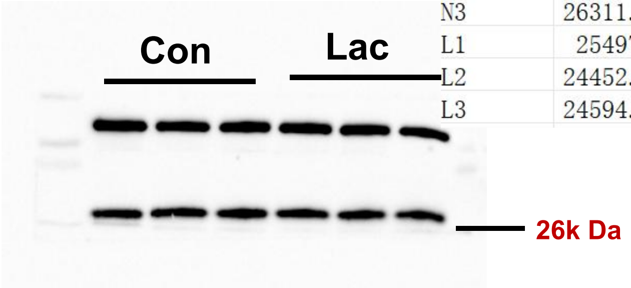

	bax	gap			
N1	25548.35	29891.933	0.854690361	0.970697523	0.880490926
N2	27585.83	26482.104	1.041678259		1.073123434
N3	26311.37	25904.054	1.015723948		1.046385639
L1	25497.3	26543.004	0.960603253		1.123919606
L2	24452.71	24763.983	0.987430455		0.972144505
L3	24594.54	21205.347	1.159827236		1.113421755

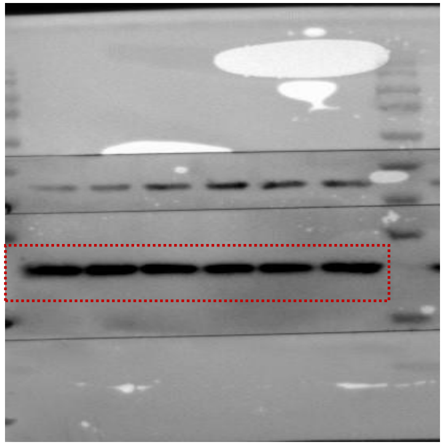

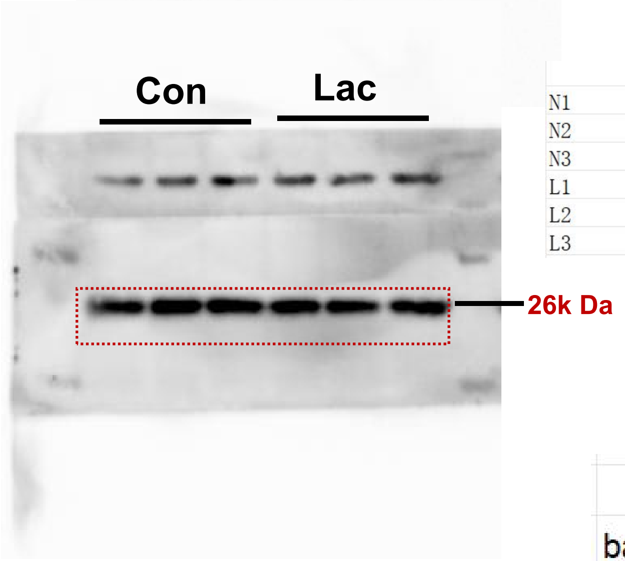

	bax	gap			
N1	20613.539	25811.004	0.798633753	1.014913793	0.786898118
N2	29458.418	25492.338	1.155579296		1.138598475
N3	28205.296	25863.882	1.090528328		1.074503407
L1	28375.368	25818.589	1.099028611		0.951062912
L2	18523.347	27947.004	0.662802603		0.829920599
L3	20262.589	26421.004	0.766912151		0.703248261

	con	Lac
bax/gap	0.880490926	1.12392
	1.073123434	0.972145
	1.046385639	1.113422
	0.786898118	0.951063
	1.138598475	0.829921
	1.074503407	0.703248

Fig4E：CM CM+LAC (n = 6)

Bcl2：22k Da

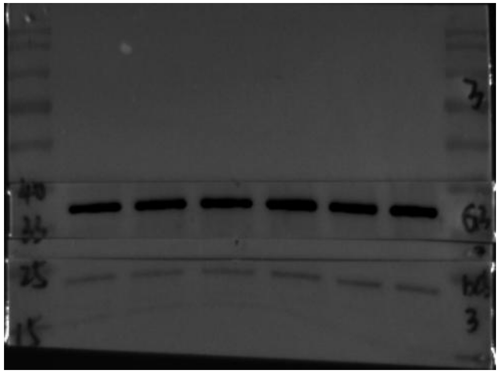

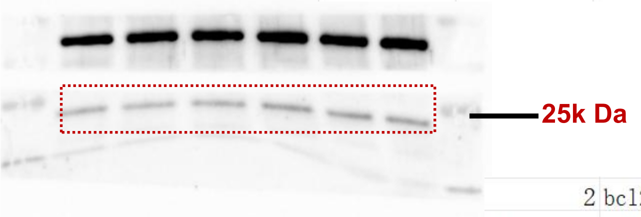

1	bc12	Gap			
N1	15262.76	20688.13	0.737754678	0.786022233	0.938592635
N2	17177.2	24326.13	0.706121341		0.898347797
N3	22999.92	25158.78	0.914190679		1.163059569
L1	26661.61	26989.35	0.987856801		1.080580697
L2	20910.88	23474.1	0.890806397		1.207456114
L3	19919.66	23758.23	0.838432164		1.187376894

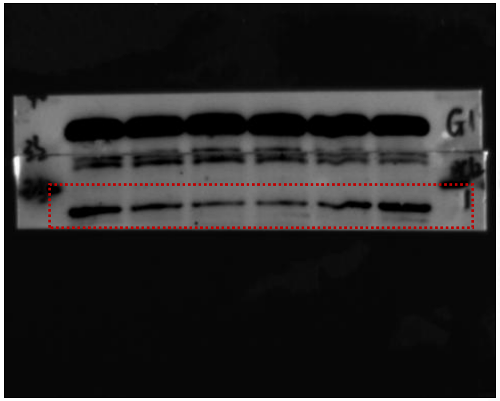

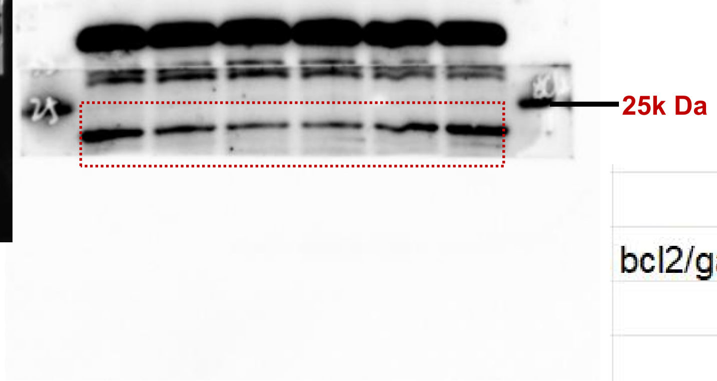

2	bc12	Gap			
N1	18854667	66328574	0.284261606	0.191843855	1.481734226
N2	10340408	63355465	0.163212566		0.850757328
N3	8422789	65773547	0.128057394		0.667508446
L1	7384078	66652220	0.110785177		0.678778479
L2	10600954	69109261	0.153394116		0.539623053
L3	15089301	62832364	0.240151731		1.875344519

	con	Lac
bcl2/gap	1.481734226	0.678778479
	0.850757328	0.539623053
	0.667508446	1.875344519
	0.938592635	1.080580697
	0.898347797	1.207456114
	1.163059569	1.187376894

Fig4H：CF CF+LAC (n = 6)

Col3：225k Da

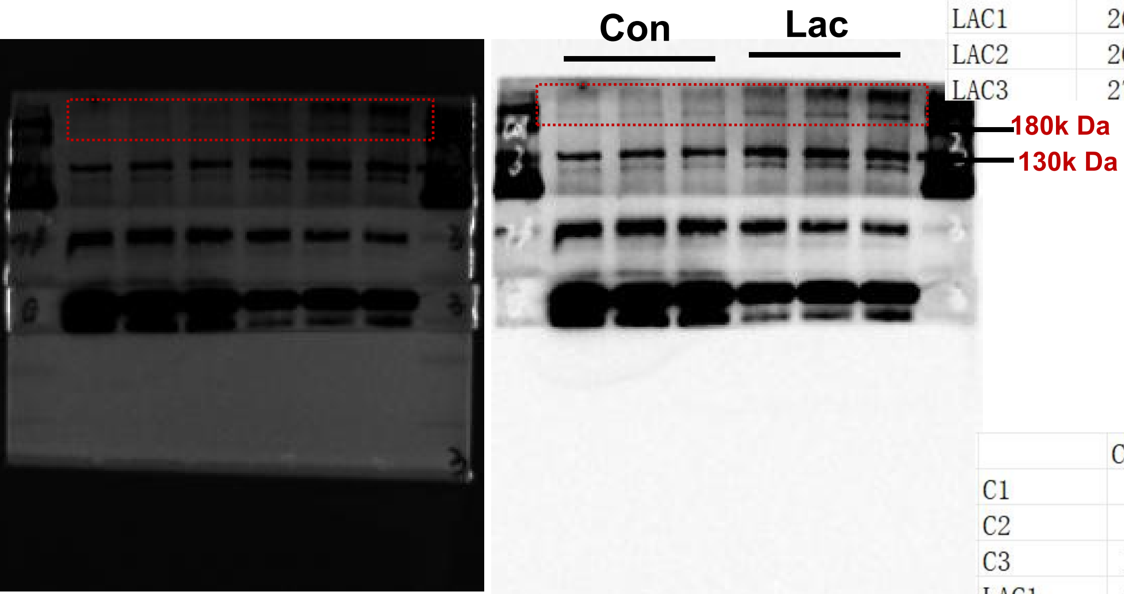

	COL3	G			
C1	1364031	22639384	0.060250358	0.100452399	0.599790137
C2	2096218	17194961	0.121908855		1.213598235
C3	2051576	17211499	0.119197985		1.186611627
LAC1	2628185	12226929	0.214950541		2.139824854
LAC2	2674832	14818799	0.180502617		1.514309298
LAC3	2720435	14290232	0.190370247		1.561578509

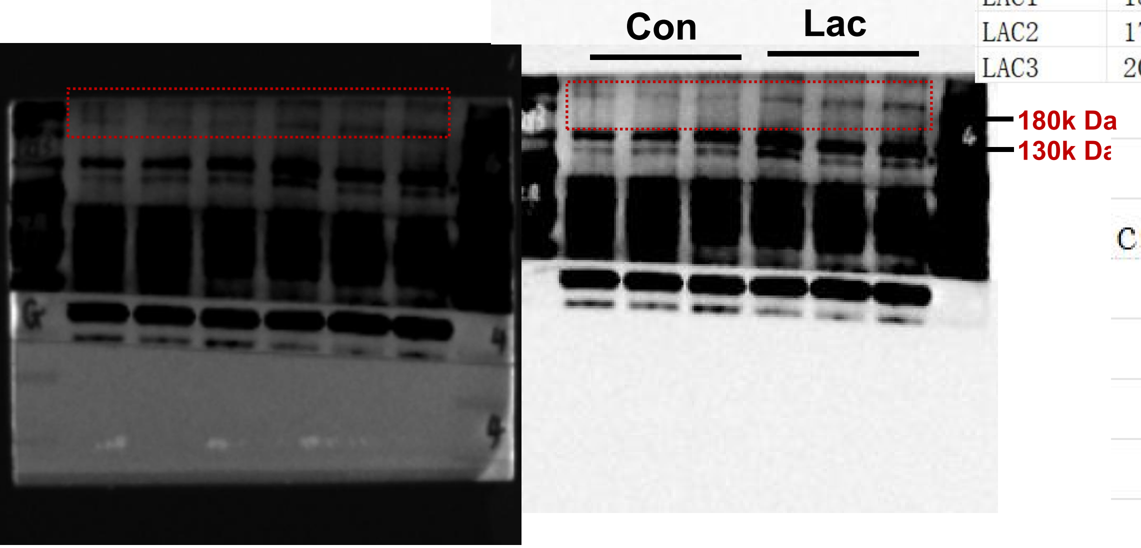

	COL3	G			
C1	7695049	15930594	0.483035912	0.716921391	0.673764123
C2	9389850	14924696	0.629148493		0.877569705
C3	15205298	14640472	1.038579767		1.448666172
LAC1	18595967	15507506	1.199159104		1.154614351
LAC2	17019330	17600499	0.96697997		2.001880081
LAC3	20528463	17457316	1.175923206		1.869071004

	con	lac
col3/gap	0.6737641	1.1546144
	0.8775697	2.0018801
	1.4486662	1.869071
	0.5997901	2.1398249
	1.2135982	1.5143093
	1.1866116	1.5615785

Fig4H：CF CF+LAC (n = 6)

α-SMA：42k Da

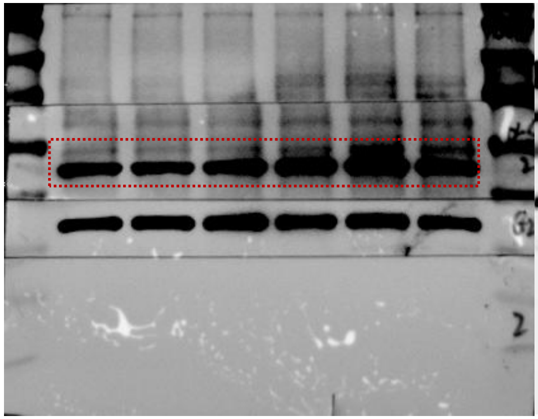

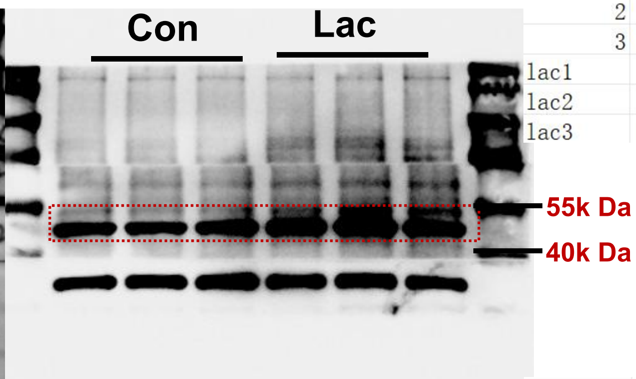

	asma	gap			
1	20668.711	22238.761	0.929400293	0.83496572	1.11309994
2	18351.711	24536.832	0.747925038		0.895755382
3	23949.246	28939.175	0.82757183		0.991144678
lac1	27506.711	25271.761	1.088436655		1.31521714
lac2	29768.589	26478.246	1.124265897		1.209668111
lac3	26507.711	25133.61	1.054671852		1.410130426

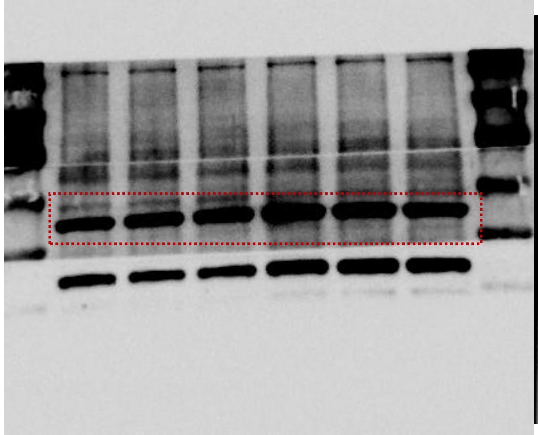

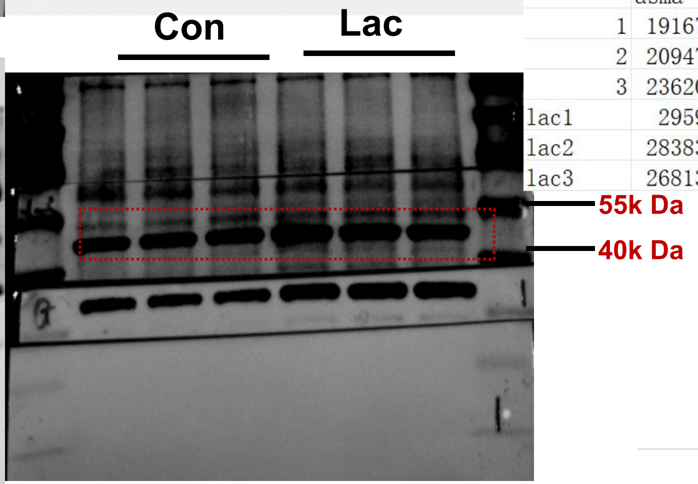

	asma	gap			
1	19167.246	24561.711	0.78037096	0.945146008	0.825661807
2	20947.246	20965.296	0.999139053		1.057126672
3	23620.004	22368.953	1.055928009		1.117211521
lac1	29591.66	28096.66	1.053209171		0.997425168
lac2	28383.196	27965.246	1.014945336		1.015819903
lac3	26813.882	29140.589	0.920155801		1.179126143

	con	lac
asma/gap	0.825661807	0.997425168
	1.057126672	1.015819903
	1.117211521	1.179126143
	1.11309994	1.31521714
	0.895755382	1.209668111
	0.991144678	1.410130426

Fig4H：CF CF+LAC (n = 6)

TGF-β1：55k Da

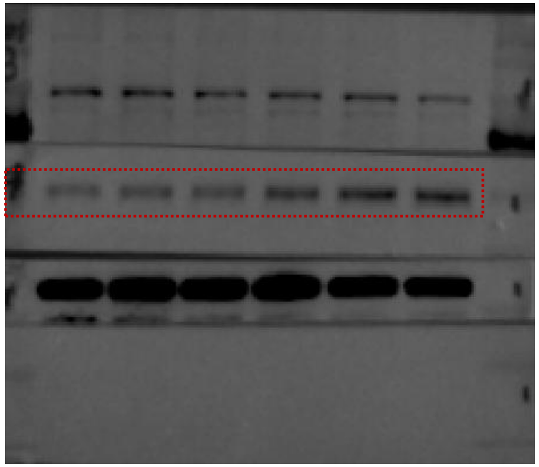

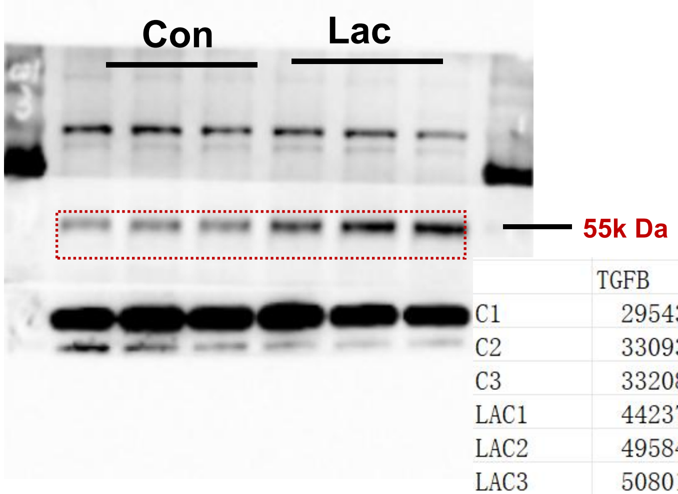

	TGFB	G			
C1	2954384	15078857	0.195928909	0.1952779	1.003333758
C2	3309379	17380393	0.190408755		0.975065564
C3	3320809	16645990	0.199496035		1.021600678
LAC1	4423793	19510490	0.226739205		1.190802413
LAC2	4958456	15679363	0.316240909		1.614059458
LAC3	5080102	14223974	0.357150681		1.79026456

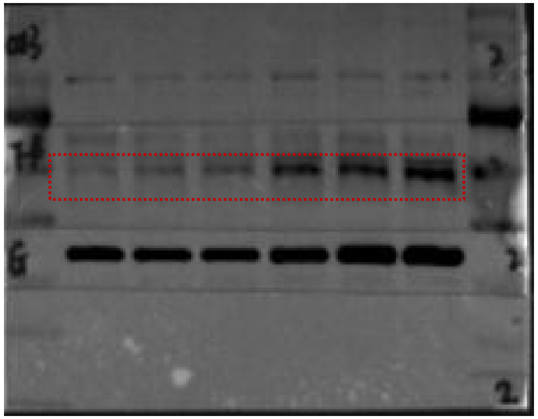

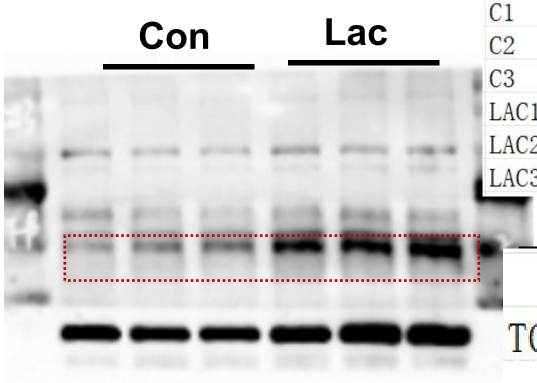

	TGFB	G			
C1	3098247	9119219	0.339749161	0.621697664	0.546486146
C2	6440570	8526788	0.755333661		1.214953353
C3	6593815	8563283	0.77001017		1.238560501
LAC1	11235281	10500768	1.069948503		1.389525157
LAC2	11027092	14991251	0.735568499		2.165034045
LAC3	13869373	14871985	0.932583848		1.234664752

	CON	LAC
TGF-β/gap	1.003333758	1.190802413
	0.975065564	1.614059458
	1.021600678	1.79026456
	0.546486146	1.389525157
	1.214953353	2.165034045
	1.238560501	1.234664752

Fig5A：HUMAN

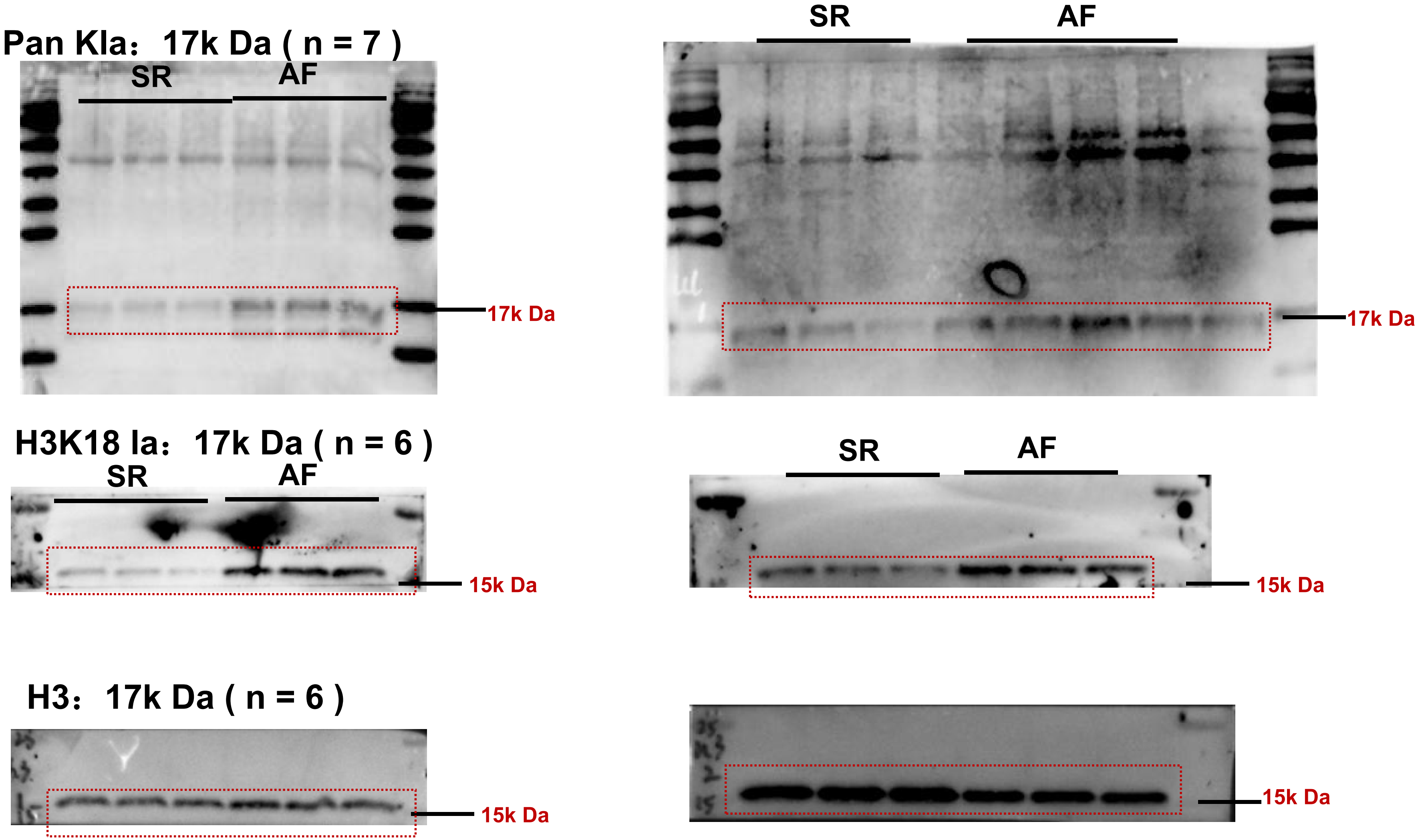
Pan Kla：17k Da (n = 7)
SR
AF
17k Da
SR
AF
17k Da
H3K18 la：17k Da (n = 6)
SR
AF
15k Da
SR
AF
15k Da
H3：17k Da (n = 6)
15k Da
15k Da

Fig5B：Rabbit

Pan Kla：17k Da (n = 6)

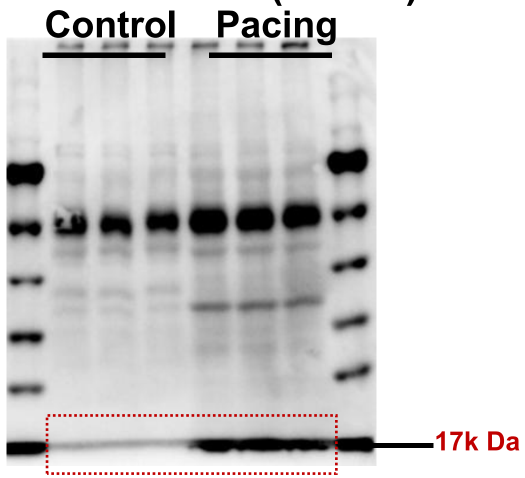

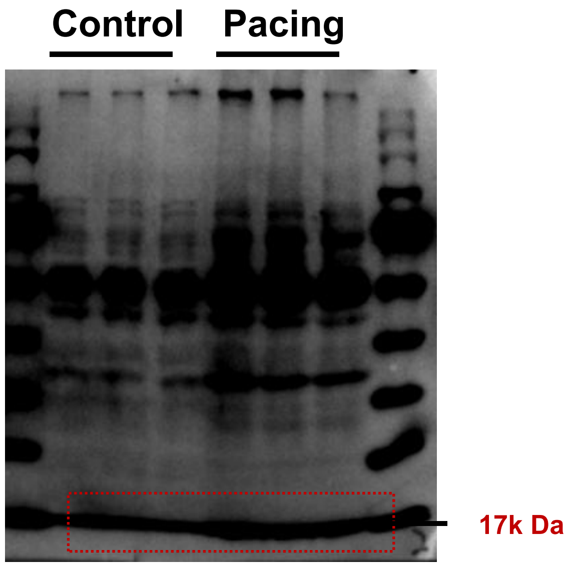

H3K18 la：17k Da (n = 6)

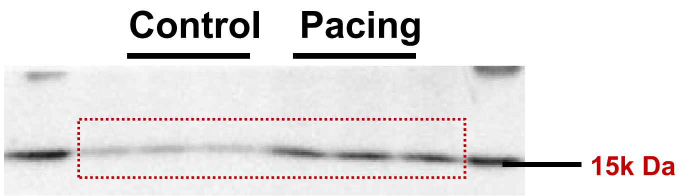

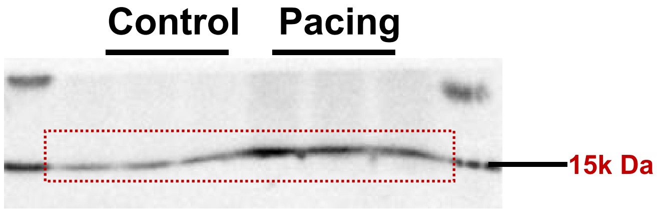

H3：17k Da (n = 6)

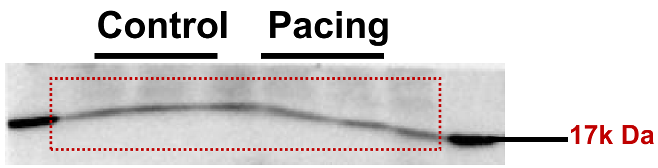

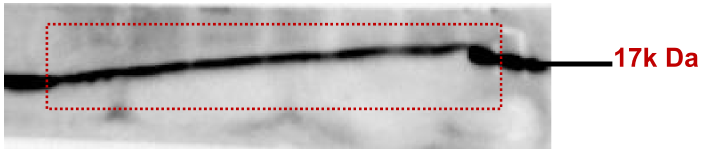

Fig 5C：CREM mice (n = 6)

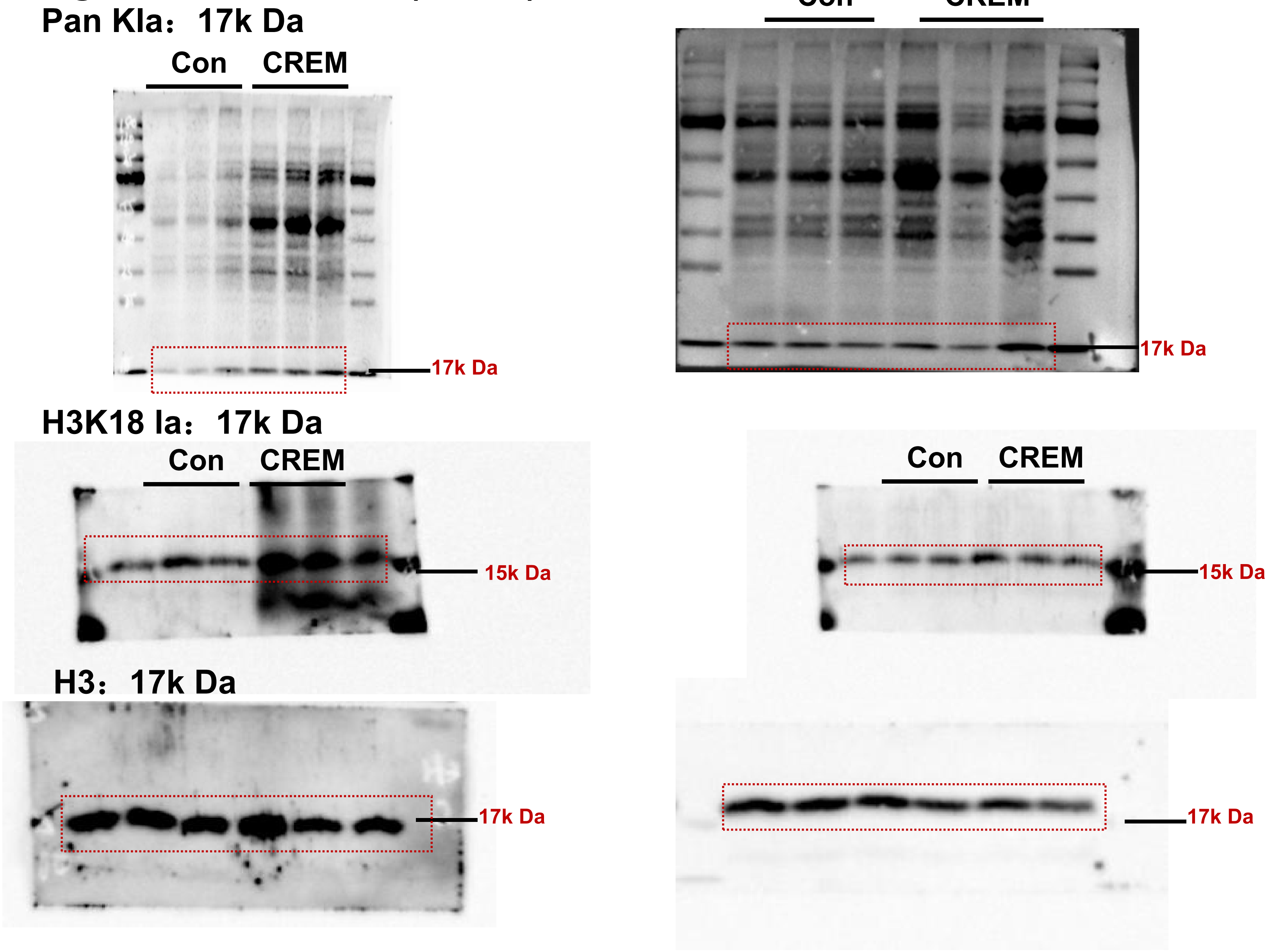

Fig5H：atrial fibroblasts isolated from human （n=3）

Pan Kla：17k Da

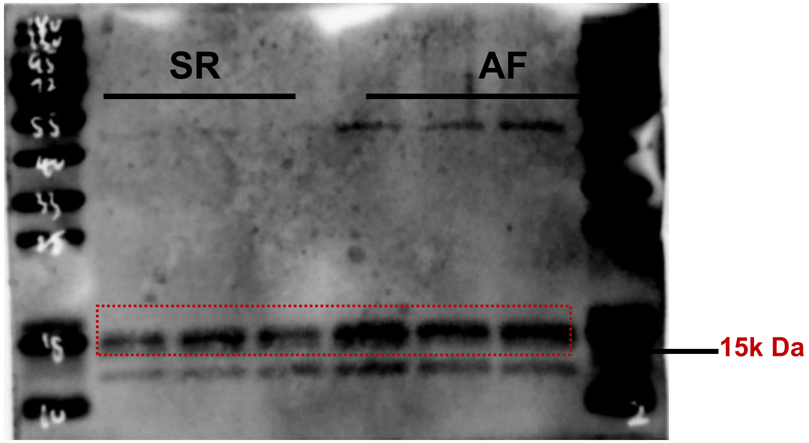

H3K18 la：17k Da

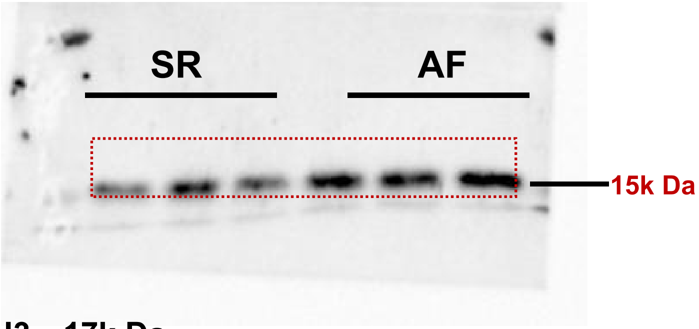

H3：17k Da

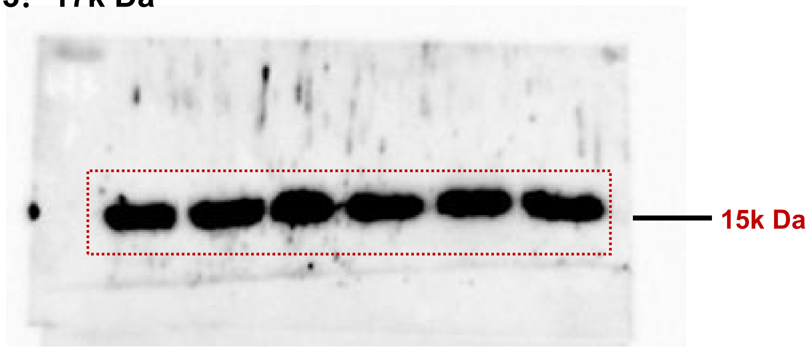

Fig4I：atrial fibroblasts isolated from 7-month CREM mice （n = 6）

Pan Kla：17k Da

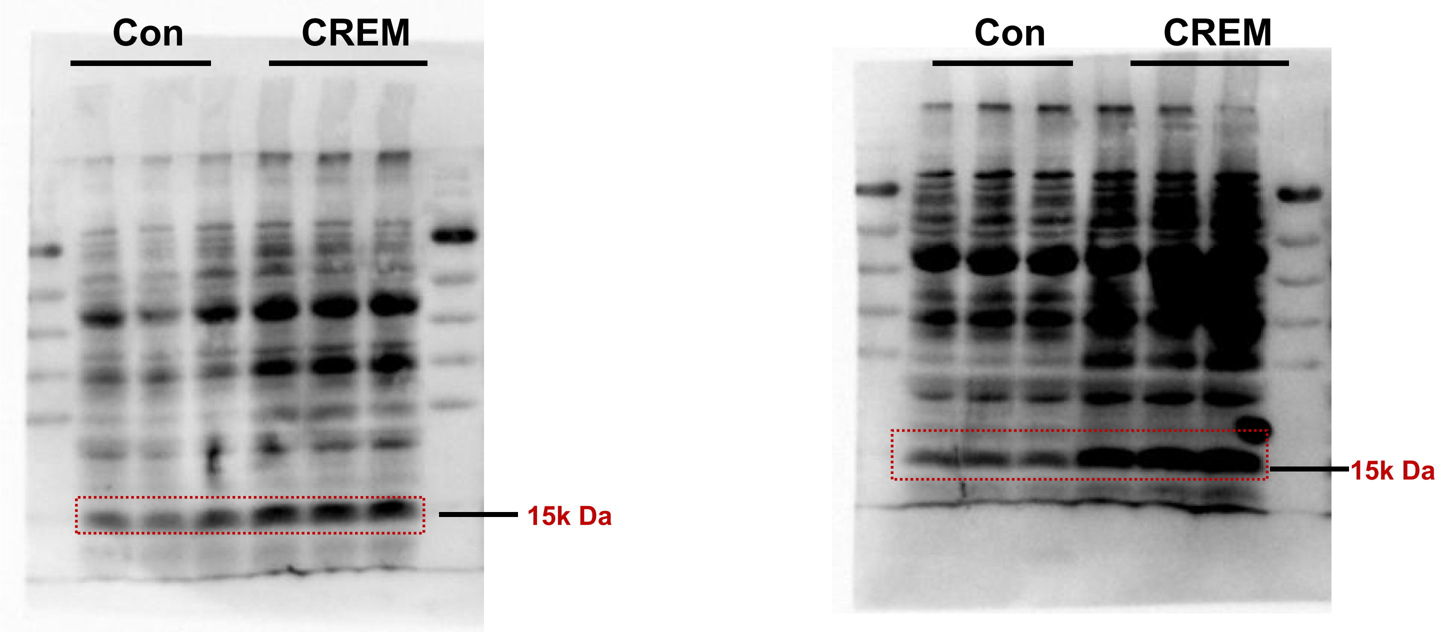

H3K18 la：17k Da

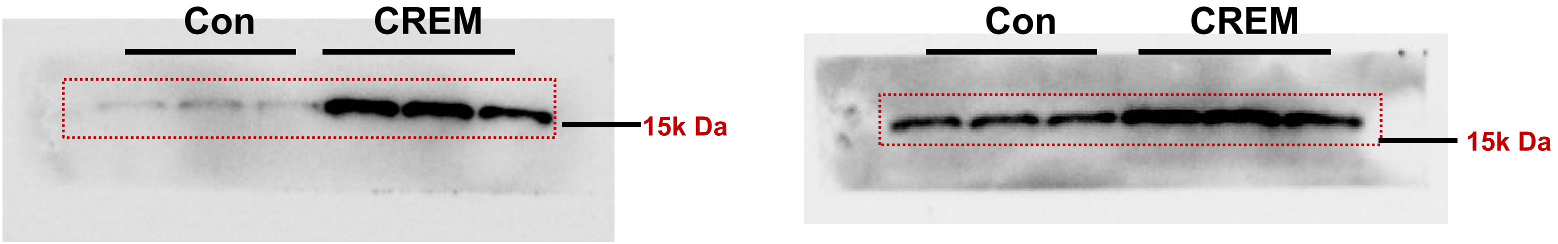

H3：17k Da

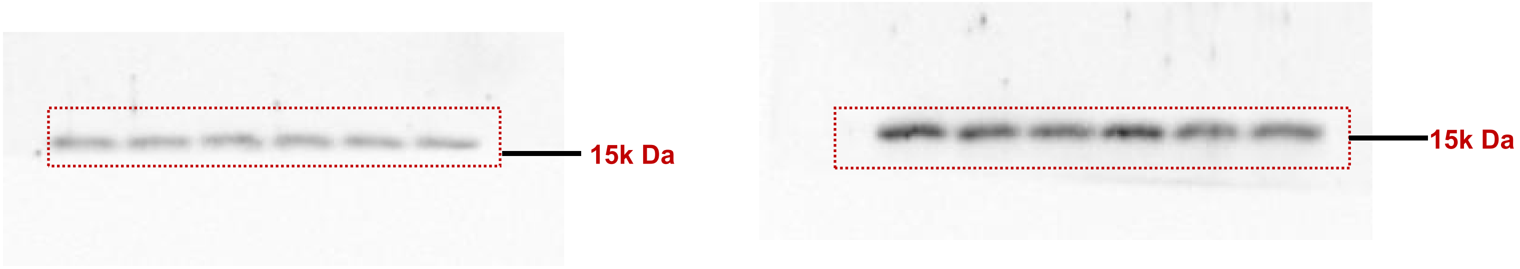

Fig6I：Con lac lac+si TGF-β1 (n = 6)

Col3：225k Da

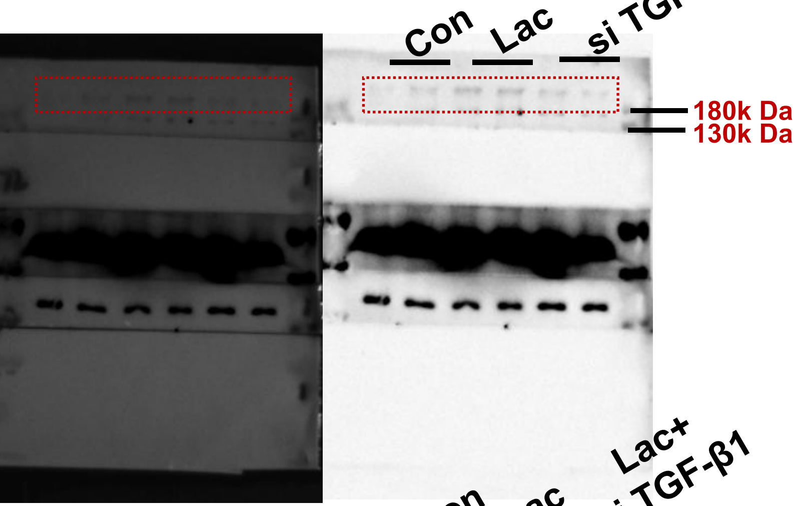

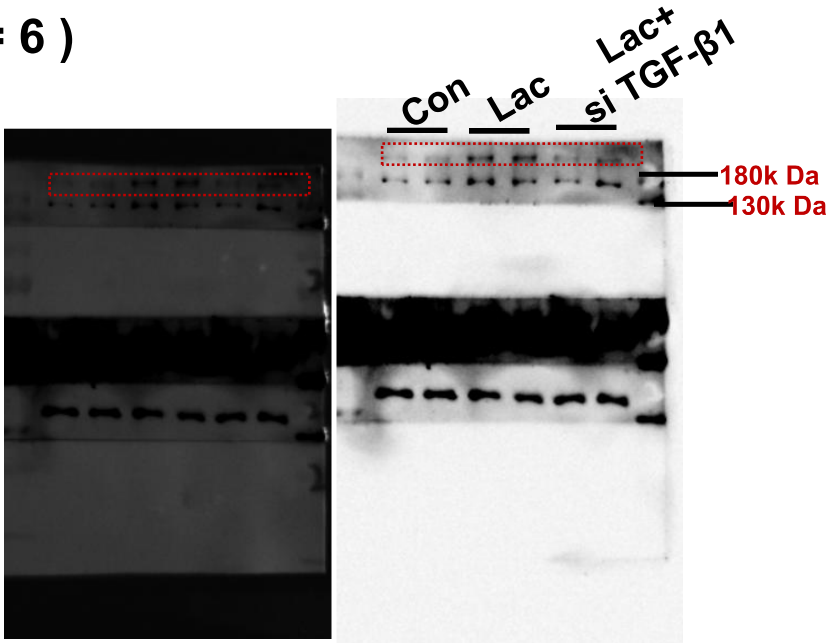

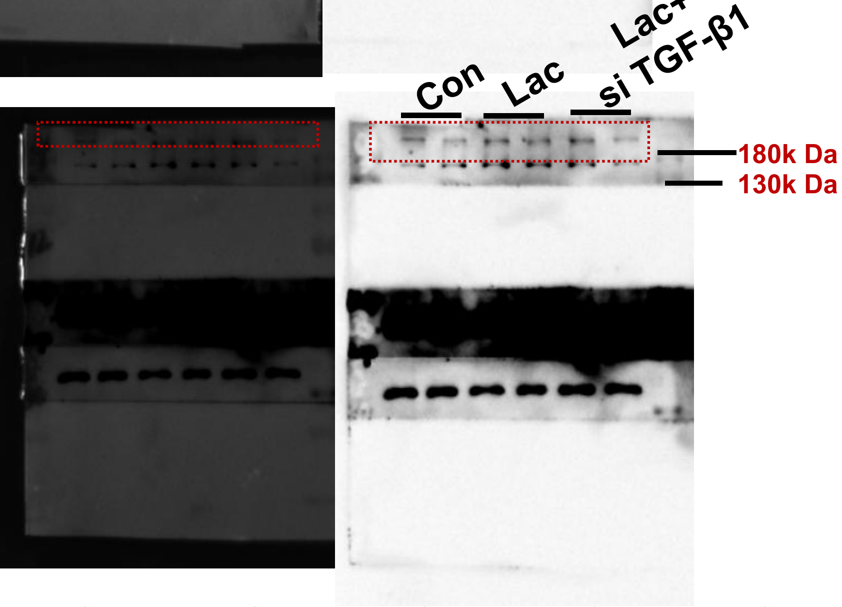

	col3	gap			
C1	19749.56	20140.539	0.980587461	0.990934066	0.989558736
C2	17334.983	17312.811	1.00128067		1.010441264
LAC1	21036.024	15118.782	1.391383512		1.418928518
LAC2	23771.974	15480.347	1.535622813		1.533658702
SI1	23837.903	20978.368	1.136308744		1.134855369
Si2	15924.569	20020.711	0.795404769		0.811151274

	col3	gap			
C1	7961.468	21418.296	0.371713417	0.435145354	0.854228166
C2	9581.61	19217.903	0.49857729		1.145771834
LAC1	25965.024	22286.024	1.165081039		2.336811287
LAC2	22828.782	21376.539	1.067936302		2.873009829
SI1	16140.125	18873.418	0.855177637		1.715235839
Si2	7344.589	17339.861	0.423566775		1.139498213

	col3	gap			
C1	8425.054	19970.439	0.421876254	0.549669814	0.767508499
C2	11249.761	16605.711	0.677463374		1.232491501
LAC1	21409.146	18115.903	1.181787405		2.801265522
LAC2	23226.146	13191.882	1.760639308		2.598870103
SI1	13232.589	15960.539	0.829081587		1.223802819
Si2	9876.104	15730.054	0.627849339		1.488231045

	CON	LAC	LAC+SI TGF-β1
col3/gapdh	0.767508499	2.801265522	1.223802819
	1.232491501	2.598870103	1.488231045
	0.989558736	1.418928518	1.134855369
	1.010441264	1.533658702	0.811151274
	0.854228166	2.336811287	1.715235839
	1.145771834	2.873009829	1.139498213

Fig6I：Con lac lac+si TGF-β1 (n = 6)

α-SMA：42k Da

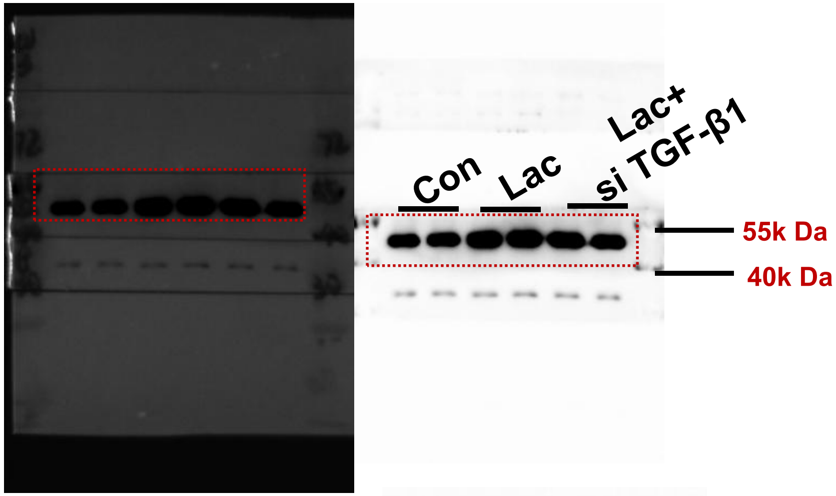

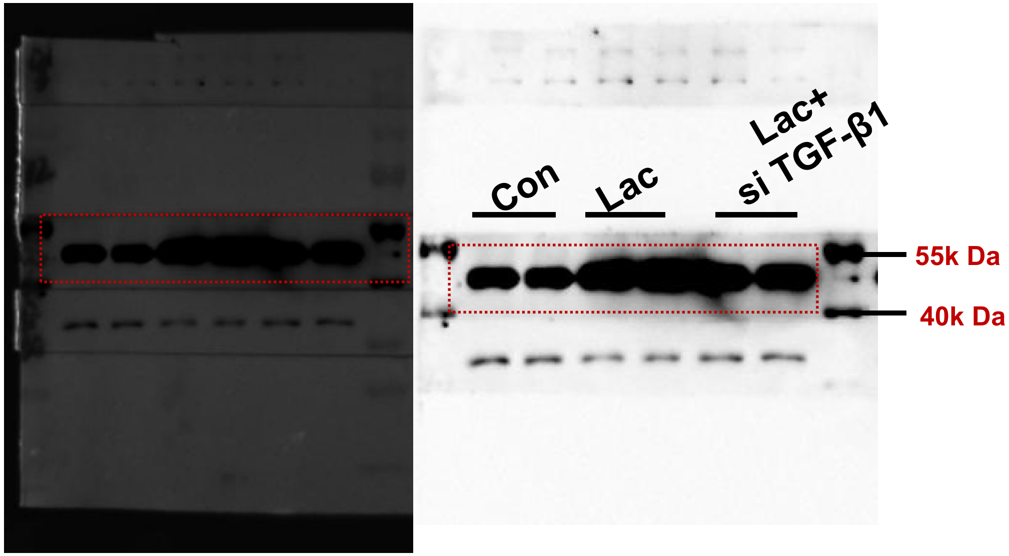

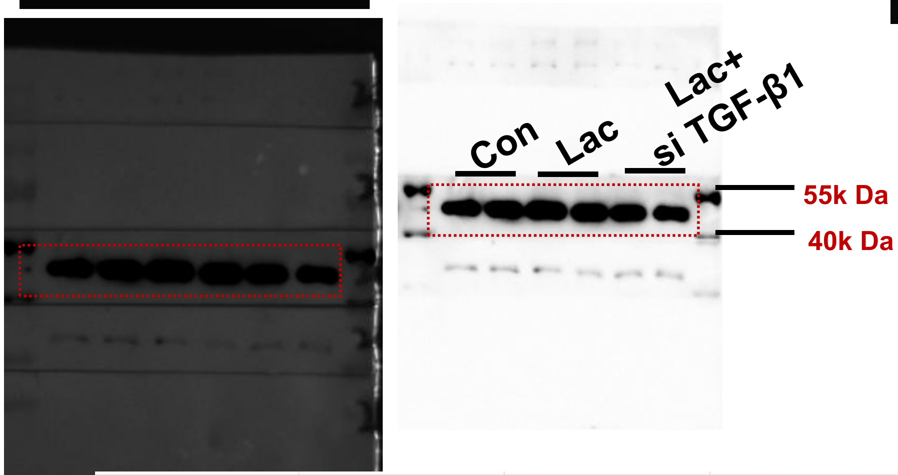

	asma	gap			
C1	18125. 518	19205. 882	0. 943748275	0. 959899298	0. 983174252
C2	20873. 983	21386. 175	0. 976050322		1. 016825748
LAC1	28294. 468	22492. 489	1. 257951843		1. 332931542
LAC2	28834. 69	21379. 66	1. 348697313		1. 381790757
SI1	23579. 518	22135. 368	1. 065241743		1. 128735037
Si2	23938. 983	20845. 882	1. 148379474		1. 17655765

	ASMA	gap			
C1	19994. 933	20140. 539	0. 992770501	1. 088738191	0. 911854208
C2	20510. 589	17312. 811	1. 184705881		1. 088145792
LAC1	22955. 882	15118. 782	1. 518368477		1. 529425456
LAC2	27729. 811	15480. 347	1. 791291306		1. 512013518
SI1	27935. 803	20978. 368	1. 331648058		1. 124032622
Si2	22028. 66	20020. 711	1. 100293591		1. 108306088

	ASMA	gap			
C1	22885. 66	19970. 439	1. 145976811	1. 24137969	0. 923147705
C2	22198. 225	16605. 711	1. 336782568		1. 076852295
LAC1	33865. 418	18115. 903	1. 869375101		1. 631250373
LAC2	26593. 004	13191. 882	2. 01586127		1. 507994881
SI1	24735. 125	15960. 539	1. 549767524		1. 15932655
Si2	21871. 589	15730. 054	1. 39043318		1. 213317029

	CON	LAC	LAC+SI TGF-β1
α-SMA/gapdh	0. 983174252	1.332931542	1.128735037
	1. 016825748	1.381790757	1.17655765
	0. 911854208	1. 529425456	1.124032622
	1. 088145792	1. 512013518	1.108306088
	0. 923147705	1. 631250373	1. 15932655
	1. 076852295	1. 507994881	1. 213317029

Fig6I：Con lac lac+si TGF-β1 (n = 6)

TGF-β1：55k Da

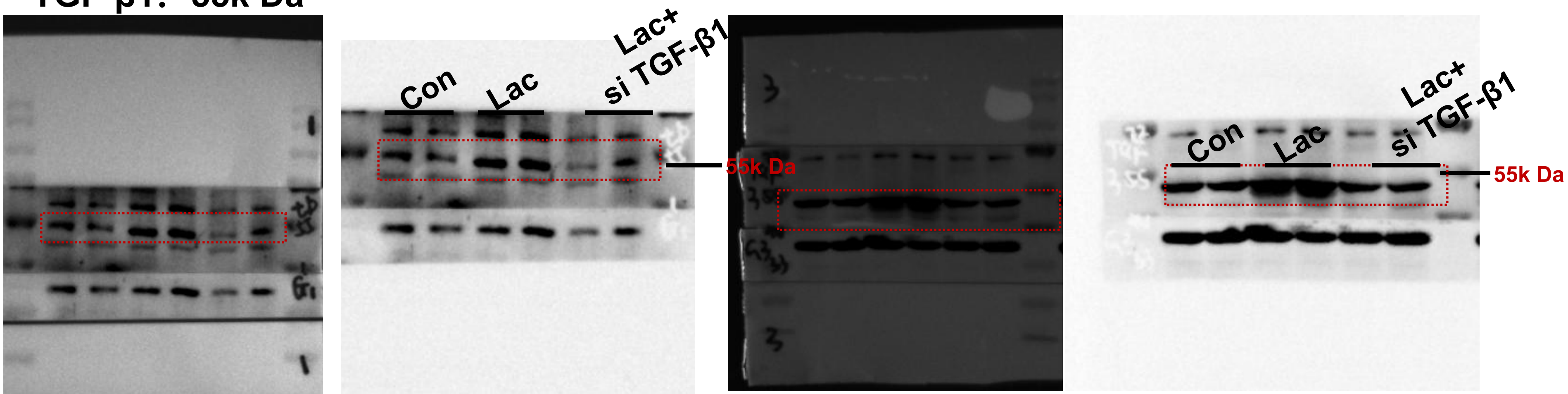

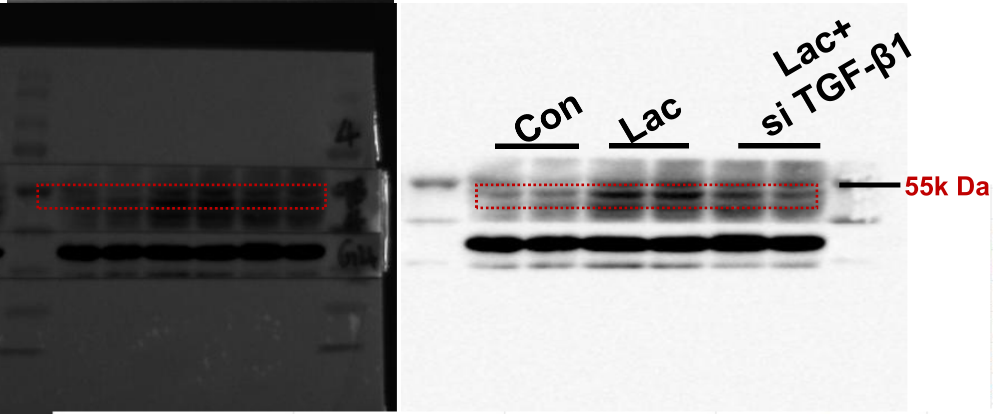

	Con	Lac	Lac+si-TGF-β1
TGF-β1/GAPDH	1.061768906	1.825183091	1.003152299
	0.938231094	2.067020591	1.022511079
	0.93824658	2.359453631	1.077905308
	1.06175342	2.445328066	1.032026833
	0.987313108	1.659806965	1.062441166
	1.012686892	1.82690487	1.043896472

	tgfb	gap			
C1	19677.589	17813.004	1.104675494	1.096026327	1.007891387
C2	16467.903	15144.61	1.087377159		0.992108613
LAC1	28908.317	14936.075	1.935469459		1.779943088
LAC2	32583.589	19267.66	1.691102552		1.530859117
SI-TGFB1	14755.953	13145.711	1.122491815		1.016128103
SI-TGFB2	17597.125	15480.761	1.1367093		1.045368013

C1	9980.175144	29618.831	0.336953704	0.359131289	0.93824658
C2	11154.811183	29254.003	0.381308875		1.06175342
LAC1	24062.903066	28397.710	0.847353624		2.359453631
LAC2	23802.417784	27103.831	0.878193821		2.445328066
SI-TGFB1	11978.417784	29492.175	0.406155793		1.065162182
SI-TGFB2	10137.175144	28964.882	0.349981576		1.038663687

	tgfb	gap			
C1	19804.154	21408.468037432	0.925061723	0.871245821	1.061768906
C2	18382.589	22488.275649276	0.81742992		0.938231094
L1	34648.861	23223.731493399	1.491959268		1.825183091
L2	36705.053	19195.982756057	1.912121629		2.067020591
SI1	19977.124	21527.589357775	0.927977794		1.003152299
SI2	20836.539	24929.124891681	0.835831149		1.022511079

Fig7H：Con lac lac+si P300 (n = 6)

Col3：225k Da

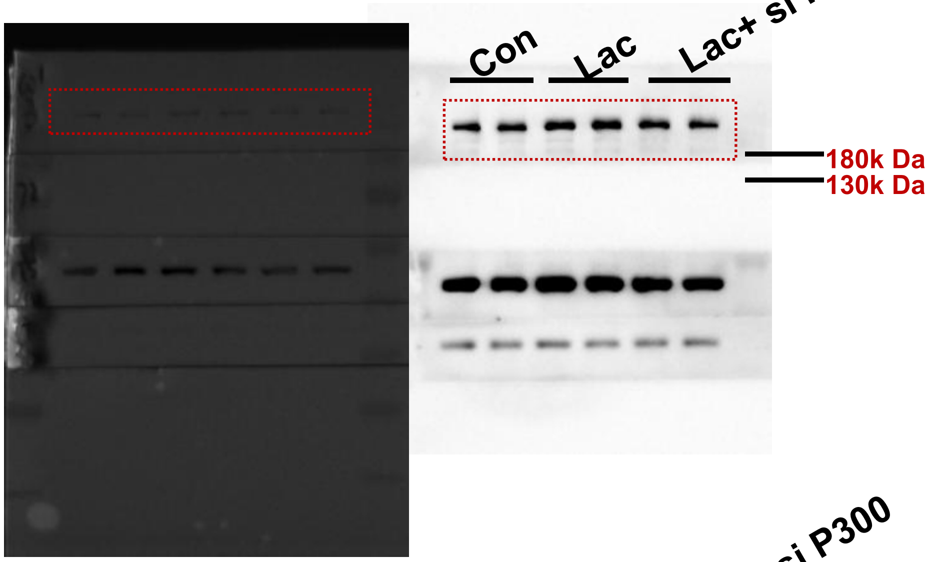

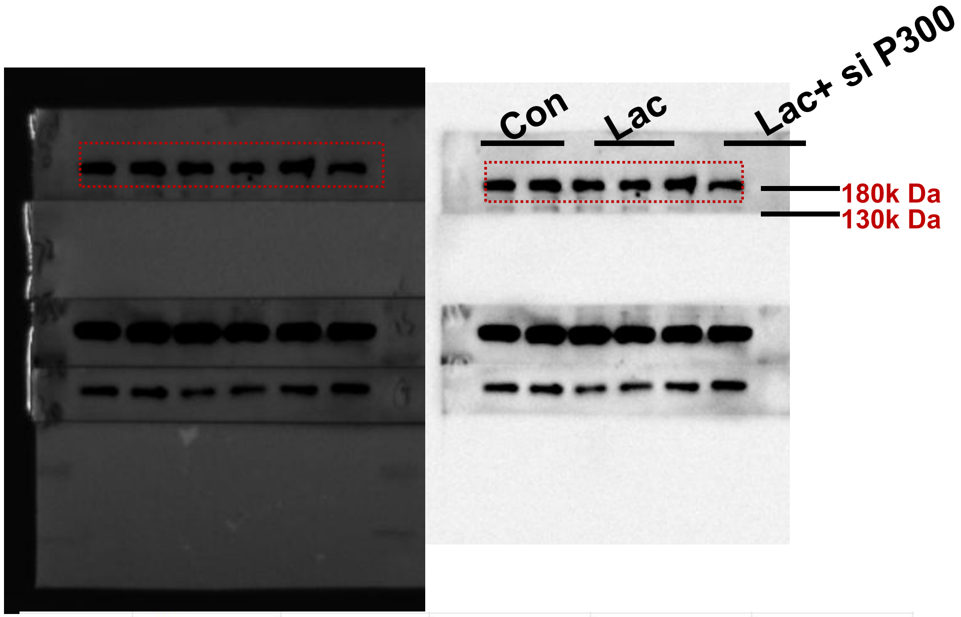

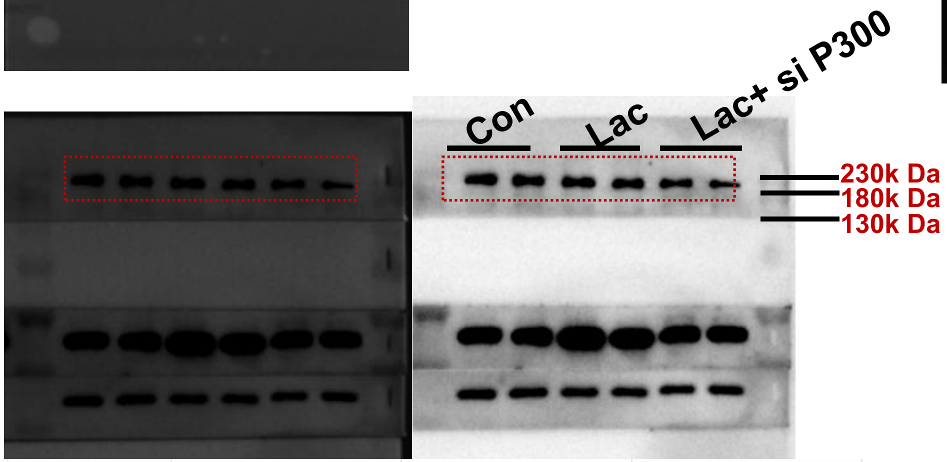

	Con	Lac	Lac+si P300
Col3/gap	0.840122613	1.31979996	1.024014837
	1.159877387	1.24229579	1.273551526
	0.948967854	1.268027986	1.090338512
	1.051032146	1.228780546	0.835825817
	0.875784308	1.085626643	1.036002185
	1.124215692	1.359239143	1.021599865

	col3	gap			
C1	15230.418	19892.782	0.765625341	0.911325714	0.840122613
C2	18967.589	17944.296	1.057026088		1.159877387
LAC1	20870.125	20653.832	1.010472294		1.31979996
LAC2	22508.296	17140.832	1.313139059		1.24229579
SIP3001	20513.66	18951.832	1.082410397		1.024014837
SIP3002	18799.175	19279.953	0.975063321		1.273551526

	col3	gap			
C1	15825.761	17896.489	0.884294176	0.9318484	0.948967854
C2	17686.175	18058.125	0.979402623		1.051032146
LAC1	16045.418	12919.953	1.241909936		1.268027986
LAC2	15271.711	14054.539	1.086603481		1.228780546
SIP3001	18185.953	17029.953	1.067880399		1.090338512
SIP3002	15220.004	20592.175	0.739115902		0.835825817

	col3	gap			
C1	17935.004	22668.953	0.791170373	0.903384961	0.875784308
C2	20014.075	19706.66	1.015599549		1.124215692
LAC1	18883.125	17126.589	1.102561929		1.085626643
LAC2	18061.489	16795.296	1.07538974		1.359239143
SIP3001	16259.317	15453.225	1.052163351		1.036002185
SIP3002	13222.933	16359.761	0.808259546		1.021599865

Fig7H：Con lac lac+si P300 (n = 6)

α-SMA：42k Da

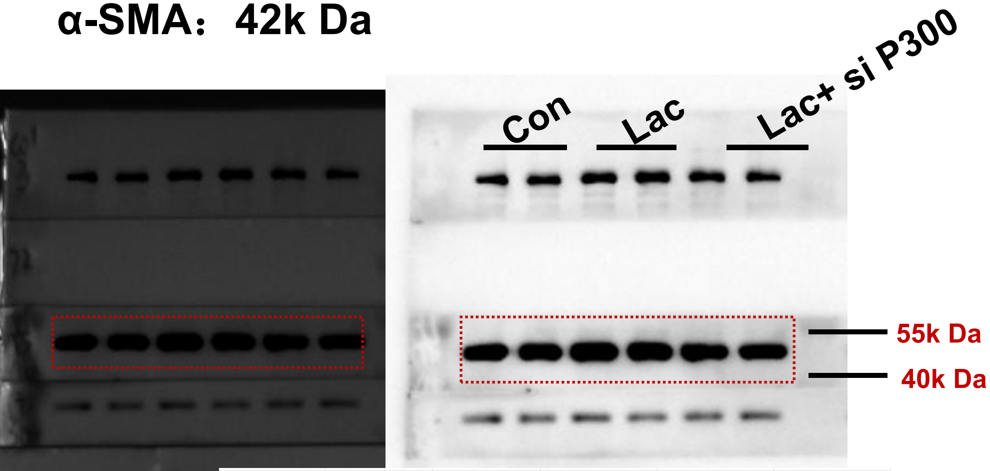

	asma	gap			
C1	20076. 296	22668. 953	0. 88562961	0. 955861714	0. 926524828
C2	20220. 882	19706. 66	1. 026093818		1. 073475172
LAC1	29823. 175	17126. 589	1. 741337694		1. 697055049
LAC2	26284. 225	16795. 296	1. 564975395		1. 76707664
SIP3001	22603. 108	15453. 225	1. 46267902		1. 425482733
SIP3002	20302. 154	16359. 761	1. 240981088		1. 401241641

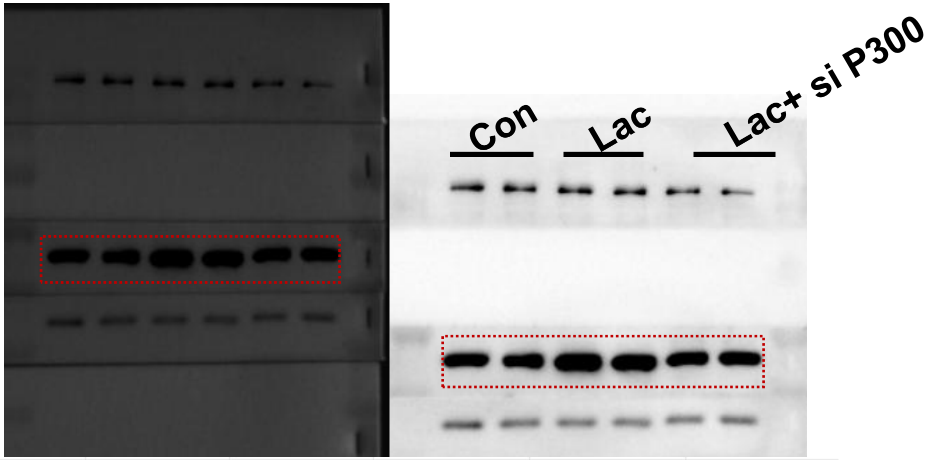

	asma	gap			
C1	20889. 711	19892. 782	1. 050115112	1. 157804502	0. 906988278
[illegible]	22708. 397	17944. 296	1. 265493893		1. 093011722
LAC1	30998. 033	20653. 832	1. 500836891		1. 429211782
LAC2	27823. 64	17140. 832	1. 623237425		1. 282690841
SIP3001	25715. 539	18951. 832	1. 35688935		1. 072221176
SIP3002	21562. 397	19279. 953	1. 118384313		1. 06501116

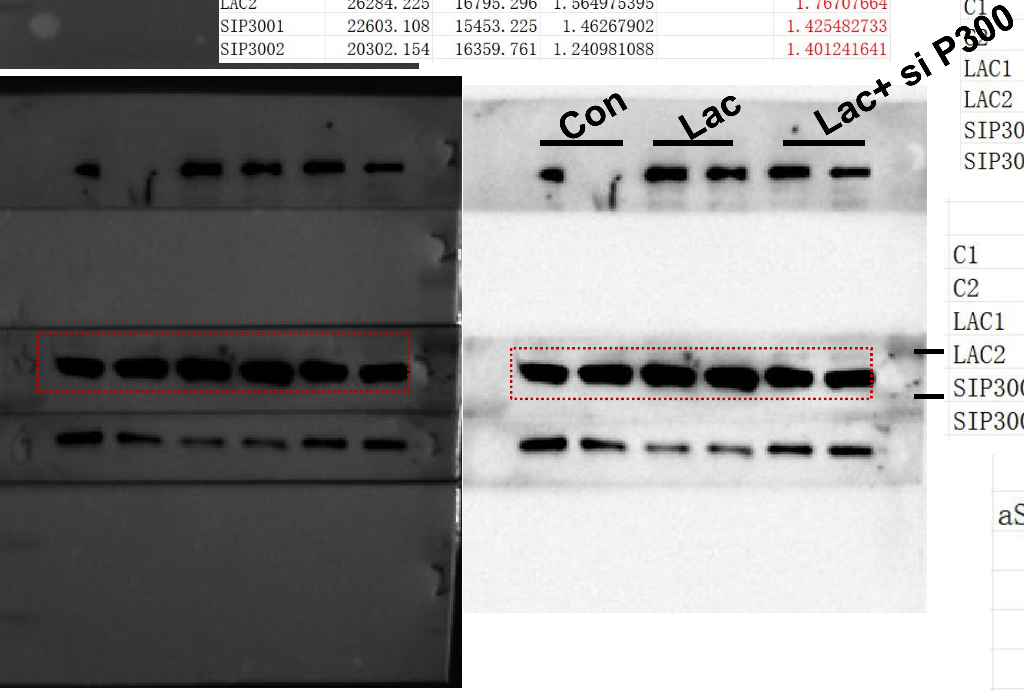

	asma	gap			
C1	17944. 468	18159. 953	0. 988134055	1. 164323494	0. 848676558
C2	20876. 761	15573. 711	1. 340512932		1. 151323442
LAC1	23495. 246	10189. 539	2. 305820312		1. 720102997
LAC2	21031. 468	10873. 418	1. 934209464		1. 957436295
SIP3001	19386. 468	13651. 832	1. 420063476		1. 059343361
SIP3002	18874. 69	15253. 64	1. 23738924		1. 252248349

	Con	Lac	Lac+si P300
aSMA/gap	0.926524828	1.697055049	1.425482733
	1.073475172	1.76707664	1.401241641
	0.906988278	1.429211782	1.072221176
	1.093011722	1.282690841	1.06501116
	0.848676558	1.720102997	1.059343361
	1.151323442	1.957436295	1.252248349

Fig5I：

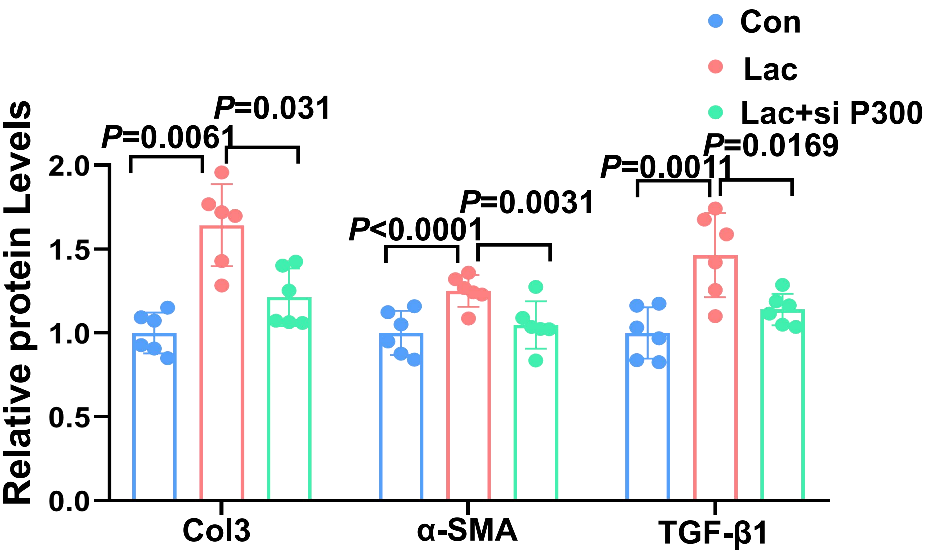

	Con	Lac	Lac+si P300
Col3/gap	0.840122613	1.31979996	1.024014837
	1.159877387	1.24229579	1.273551526
	0.948967854	1.268027986	1.090338512
	1.051032146	1.228780546	0.835825817
	0.875784308	1.085626643	1.036002185
	1.124215692	1.359239143	1.021599865

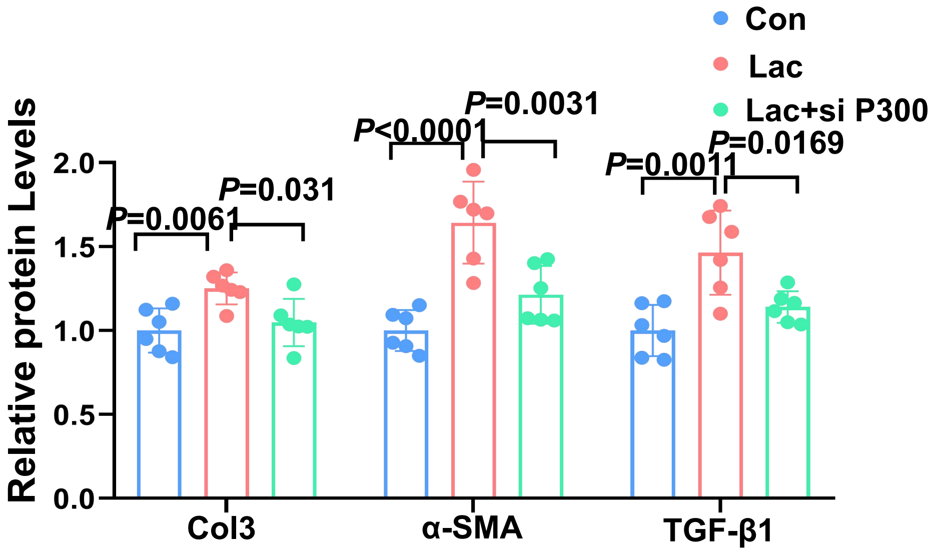

	Con	Lac	Lac+si P300
aSMA/gap	0.926524828	1.697055049	1.425482733
	1.073475172	1.76707664	1.401241641
	0.906988278	1.429211782	1.072221176
	1.093011722	1.282690841	1.06501116
	0.848676558	1.720102997	1.059343361
	1.151323442	1.957436295	1.252248349

Fig7H：Con lac lac+si P300 (n = 6)

TGF-β1 ：55k Da

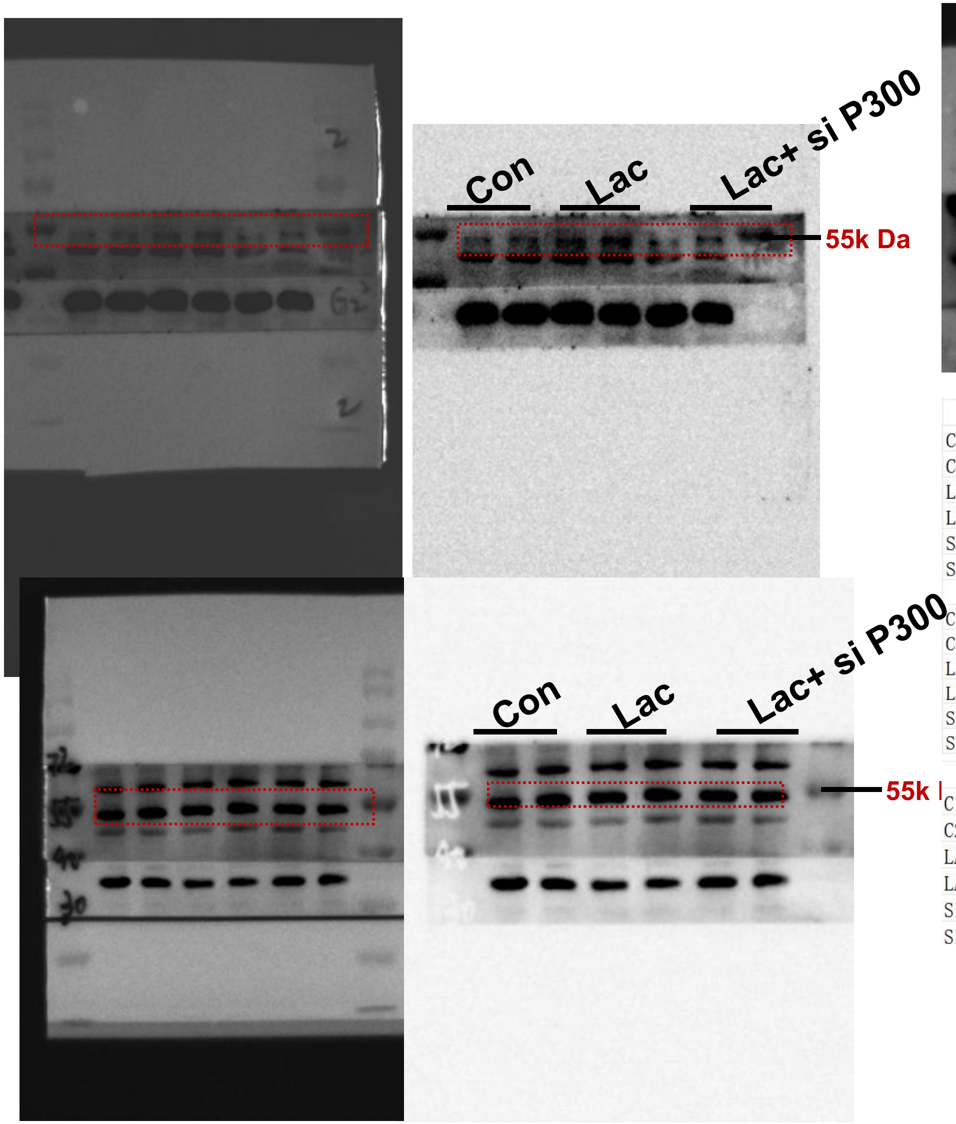

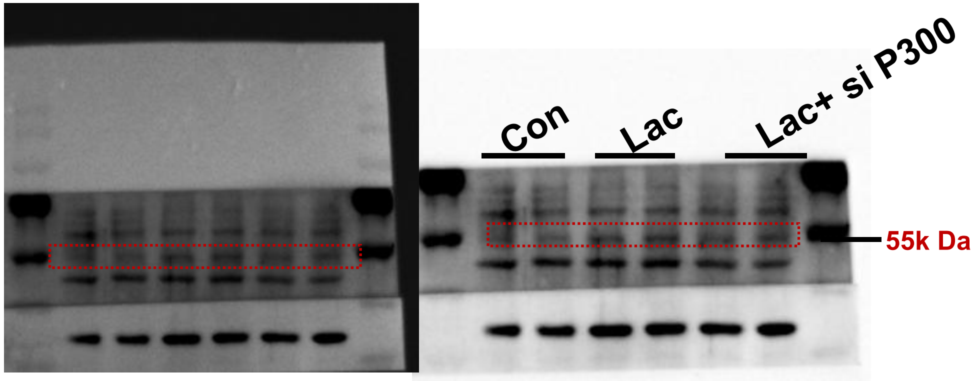

	tgfb	gap			
C1	12457.710678	27402.660425	0.454616832	0.530978748	0.856186493
C2	15965.417784	26287.417784	0.607340664		1.143813507
LAC1	21511.317279	28182.246212	0.763293214		1.437521212
LAC2	23259.267027	26226.417784	0.886864047		1.67024396
SI-P300 3	12636.053823	26979.589357	0.468356047		1.030221527
SI-P300 4	16384.710678	25703.296464	0.637455616		1.049584942

	tgfb	gap			
C1	10593.276	14096.882	0.751462345	0.744619629	1.009189544
C2	11638.903	16775.64	0.693797852		0.990810456
LAC1	19454.368	17930.004	1.085017494		1.56388131
LAC2	21957.539	16696.569	1.315092879		1.750044947
SI-P300 1	14786.66	16576.64	0.892017924		1.28570292
SI-P300 2	14978.104	16813.761	0.890824129		1.185454116

	tgfb	gap			
C1	20307.861	22543.468	0.900831274	1.043300101	0.863444059
C2	22710.054	19152.175	1.185768927		1.136555941
LAC1	20035.66	17582.589	1.139517053		1.092223659
LAC2	22473.368	15812.832	1.421210824		1.198556305
SI-P300 5	22352.832	21472.004	1.041022161		1.155623912
SI-P300 6	22349.175	17591.882	1.270425472		1.071393796

TGF-β1/GAPDH	C	LAC	LAC+si P300
	0.856186493	1.437521212	1.030221527
	1.143813507	1.67024396	1.049584942
	1.009189544	1.56388131	1.28570292
	0.990810456	1.750044947	1.185454116
	0.863444059	1.092223659	1.155623912
	1.136555941	1.198556305	1.071393796

Fig7I：Con lac lac+si P300 (n = 6)

H3K18 la：17k Da

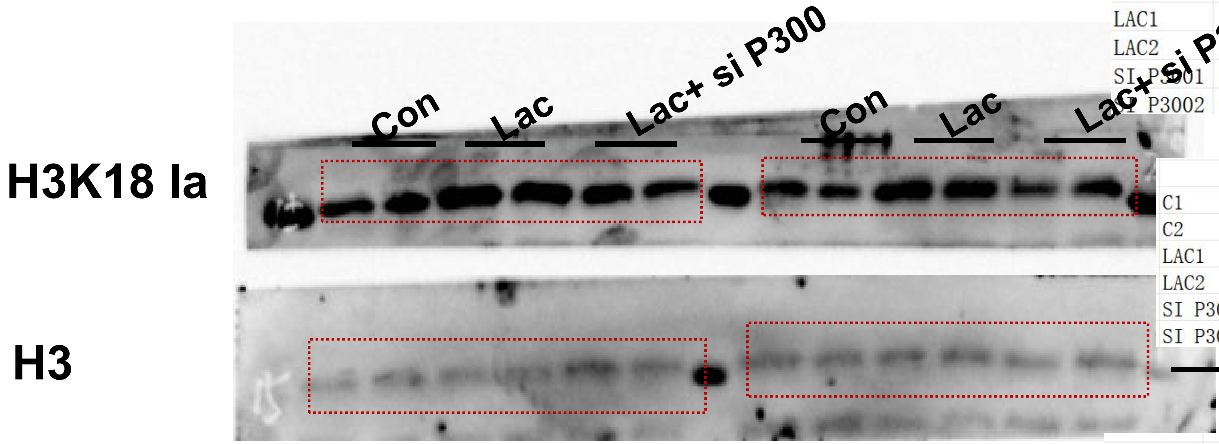

	LYS18	H3			
C1	18982. 518	10339. 782	1. 835872168	1. 70845331	1. 074581411
C2	20422. 69	12917. 296	1. 581034452		0. 925418589
LAC1	[illegible]	10448. 347	3. 0123052		1. 640803348
LAC2	30942. 933	13763. 296	2. 248221138		1. 421993769
SI P3001	27993. 569	21774. 489	1. 285613132		0. 813146817
[illegible] P3002	23543. 104	13072. 761	1. 800928205		0. 980966015

	LYS18	H3			
C1	26435. 761	29118. 075	0. 907881479	0. 881286009	1. 030178024
C2	21049. 175	24627. 832	0. 854690539		0. 969821976
LAC1	31185. 903	25356. 711	1. 229887543		1. 354678525
LAC2	26095. 761	21935. 175	1. 189676444		1. 39193824
SI P3001	18104. 711	14902. 539	1. 214874257		1. 338141911
SI P3002	20939. 175	18307. 782	1. 143730846		1. 338181242

	LYS18	H3			
C1	5793. 811	4183. 225	1. 385010608	2. 270206878	0. 610081232
C2	13642. 761	4323. 619	3. 155403147		1. 389918768
LAC1	22954. 296	7896. 66	2. 906836055		2. 098782521
LAC2	20815. 004	4831. 468	4. 308215226		1. 365345417
SI P3001	15164. 468	7285. 589	2. 081433361		0. 659641023
SI P3002	13424. 225	7486. 941	1. 793018671		1. 294588403

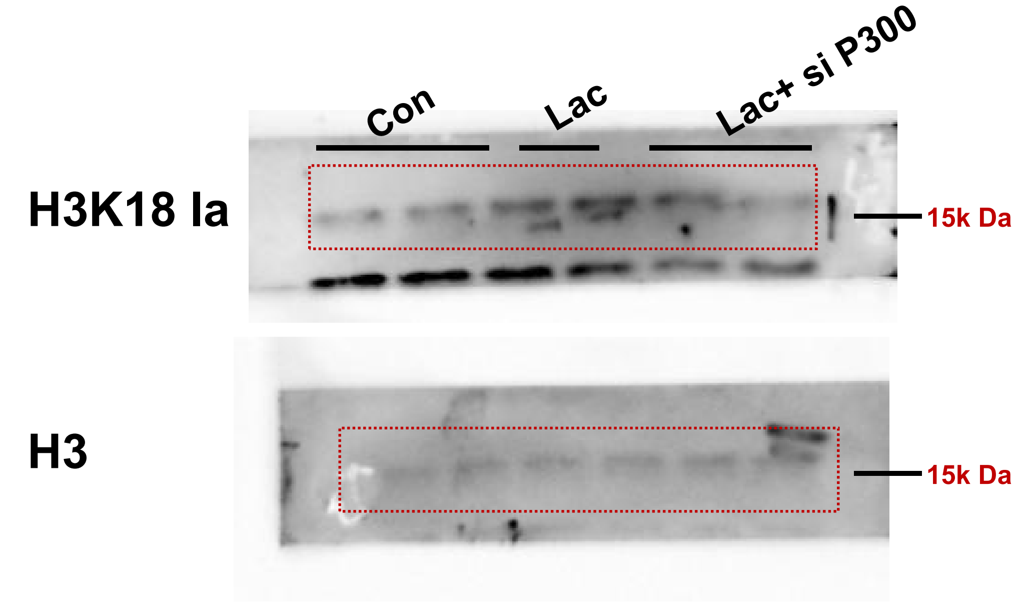

	Con	Lac	Lac+si P300
H3k18la/H3	1.074581411	1.640803348	0.813146817
	0.925418589	1.421993769	0.980966015
	1.030178024	1.354678525	1.338141911
	0.969821976	1.39193824	1.338181242
	0.610081232	2.098782521	0.659248948
	1.389918768	1.365345417	1.294588403

Figure S4B：CREM mice （7month）(n = 6)

Col3：225k Da

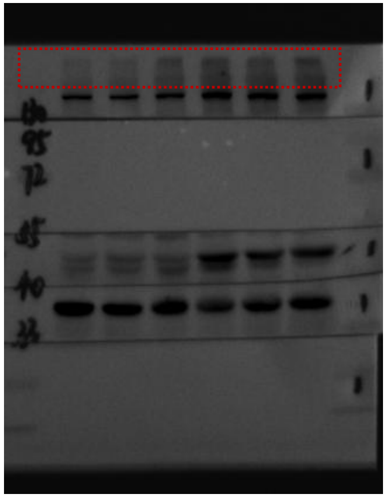

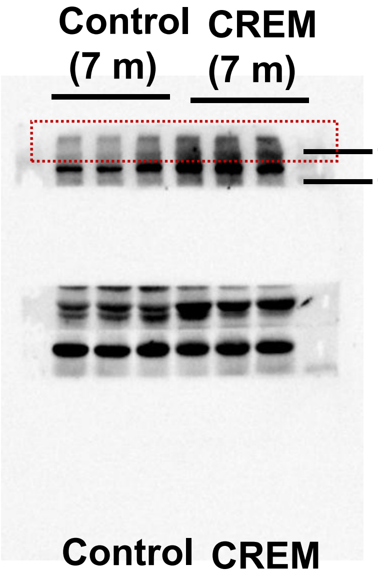

	col3	gap			
FVB1	10341. 64	24648. 64	0. 419562296	0. 526368522	0. 797088501
FVB2	11770. 104	23013. 761	0. 511437657		0. 971634198
FVB3	15296. 054	23601. 175	0. 648105613		1. 231277301
CR1	20849. 004	22721. 761	0. 917578704		1. 415785768
CR2	22098. 418	24336. 64	0. 908030772		1. 775447622
CR3	19247. 418	25220. 468	0. 763166568		1. 818958887

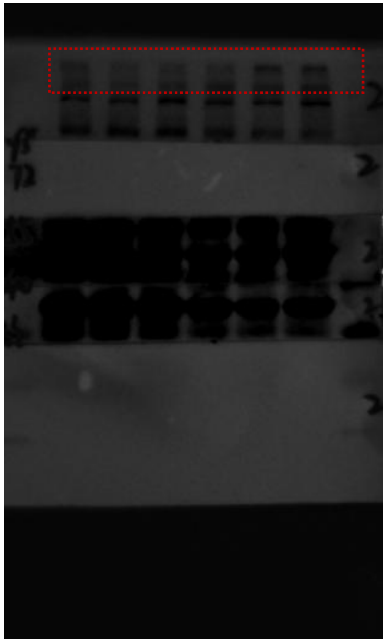

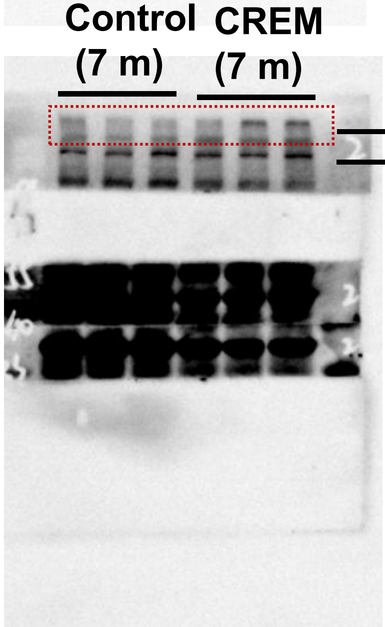

	col3	gap			
FVB1	17757. 489	28279. 761	0. 627922174	0. 477019366	1. 316345244
FVB2	11238. 66	28424. 397	0. 395387807		0. 828871604
FVB3	11010. 296	27002. 69	0. 407748117		0. 854783151
CR1	13336. 125	21663. 761	0. 615596018		1. 55694234
CR2	19164. 246	18311. 518	1. 046567849		1. 666715865
CR3	19525. 761	21644. 933	0. 902093853		2. 212380181

col3/gap	FVB	CREM
	0.797088501	1.415785768
	0.971634198	1.775447622
	1.231277301	1.818958887
	1.316345244	1.55694234
	0.828871604	1.666715865
	0.854783151	2.212380181

Figure S4B：CREM mice （7month）(n = 6)

α-SMA：42k Da

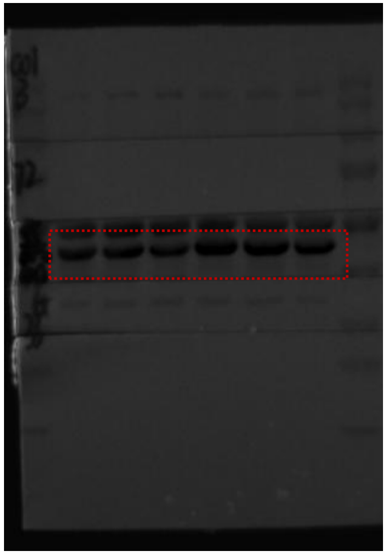

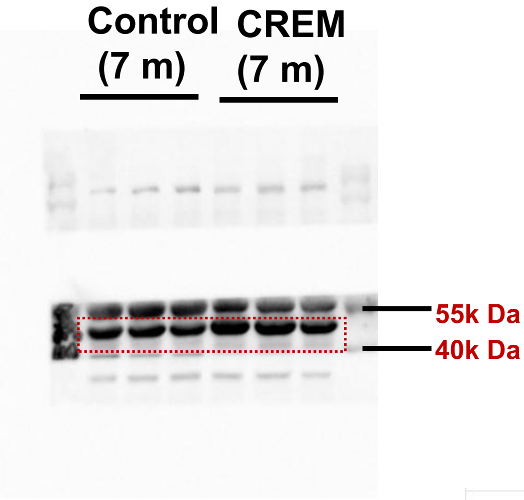

	asma	gap			
FVB1	19239. 882	18314. 276	1. 050540136	0. 99775952	1. 0528991
FVB2	21274. 66	21228. 953	1. 00215305		1. 0044033
FVB3	23045. 903	24501. 66	0. 940585373		0. 9426974
CR1	31223. 589	22059. 075	1. 415453232		1. 4124122
CR2	28234. 075	23036. 146	1. 225642301		1. 3030633
CR3	29045. 903	18816. 296	1. 543656786		1. 4693934

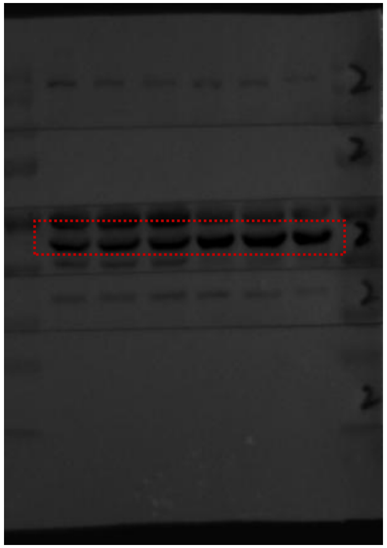

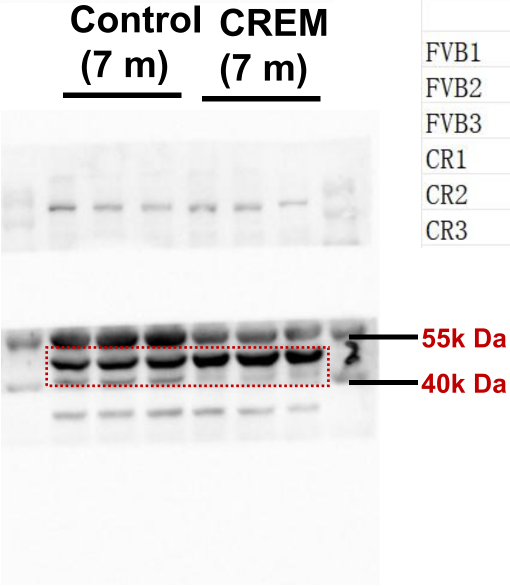

	asma	gap			
FVB1	17021. 64	19239. 882	0. 88470605	0. 897638087	0. 985593261
FVB2	18992. 518	21274. 66	0. 892729567		0. 994531737
FVB3	23844. 468	26045. 903	0. 915478646		1. 019875001
CR1	25273. 882	23223. 589	1. 088284933		1. 230109067
CR2	25565. 953	19234. 075	1. 329201066		1. 488917939
CR3	22419. 054	16372. 104	1. 36934471		1. 495769144

α -SMA/gap	FVB	CREM
	1.052899136	1.4124122
	1.004403396	1.3030633
	0.942697468	1.4693934
	0.985593261	1.2301091
	0.994531737	1.4889179
	1.019875001	1.4957691

Figure S4B：CREM mice （7month）(n = 6)

TGF-β1：55k Da

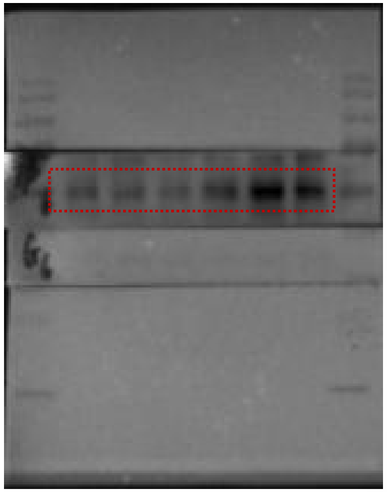

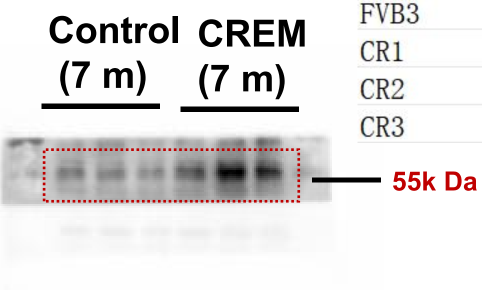

	tgfb	gap			
FVB1	5490. 205	12686. 69	0. 432753145	0. 381990807	1. 132888899
FVB2	5293. 205	14895. 154	0. 355364235		0. 930295253
FVB3	4129. 912	11540. 74	0. 357855042		0. 936815848
CR1	9085. 447	16337. 205	0. 556120034		1. 564929669
CR2	14318. 376	16015. 083	0. 894055685		2. 065971547
CR3	11733. 376	14087. 134	0. 832914346		2. 327518821

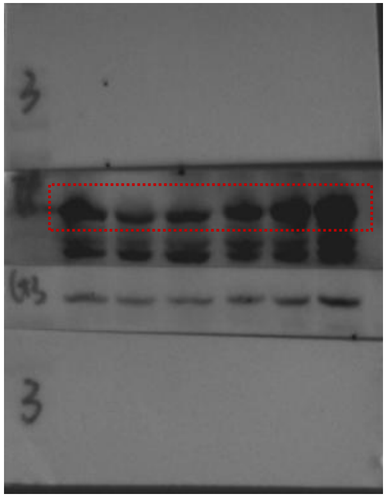

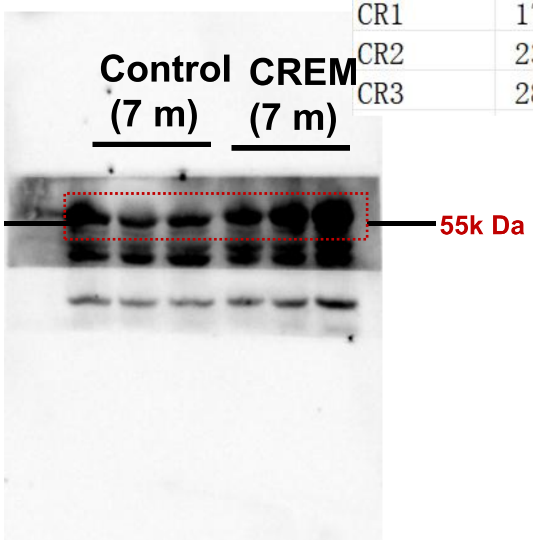

	tgfb	gap			
FVB1	17519. 832	16974. 953	1. 032098999	0. 923043108	1. 11814821
FVB2	9939. 033	12139. 832	0. 818712565		0. 886971105
FVB3	14541. 761	15835. 217	0. 91831776		0. 994880685
CR1	17127. 539	16010. 539	1. 069766546		1. 306644836
CR2	23374. 468	17309. 296	1. 350399693		1. 30840132
CR3	28669. 518	21819. 589	1. 313934832		1. 430806295

TGF-β 1/gap	FVB	CREM
	1.1181482	1.3066448
	0.8869711	1.3084013
	0.9948807	1.4308063
	1.1328889	1.5649297
	0.9302953	2.0659715
	0.9368158	2.3275188

Figure S4F：CREM mice （7month）(n = 6)

Nav 1.5：226k Da

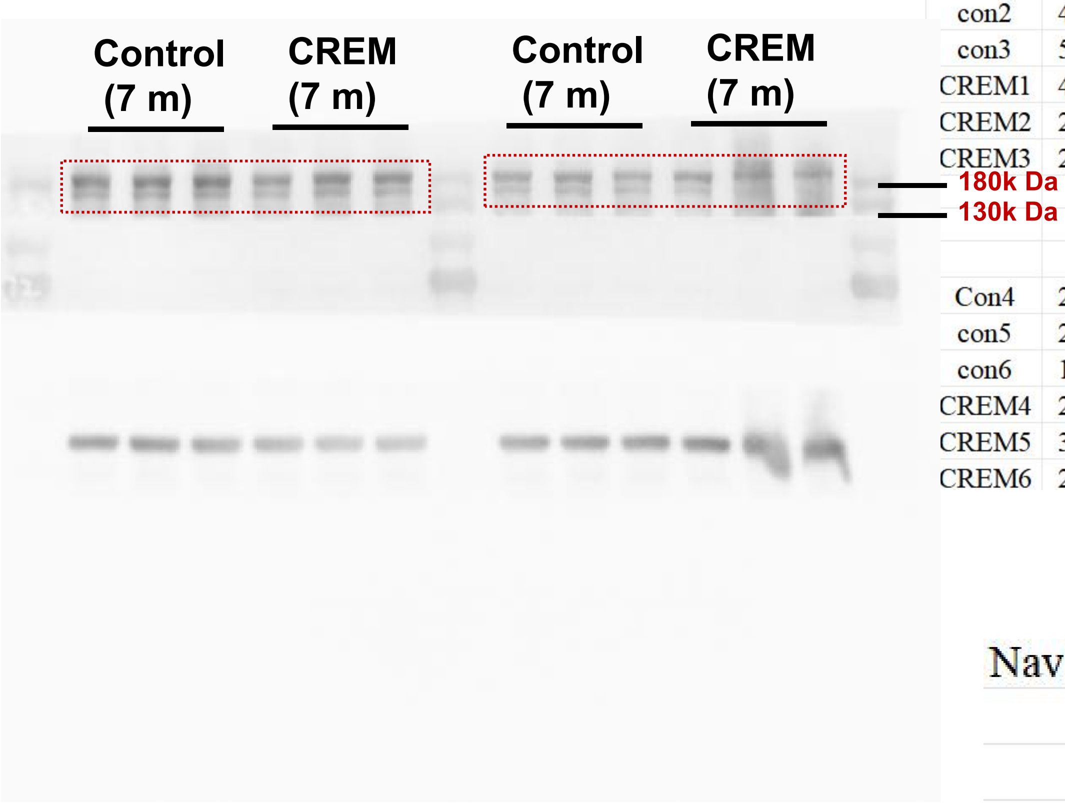

	Na	gap			
Con1	4358347	2675185	1.629175926	1.714854815	0.950037235
con2	4565334	2949049	1.548069903		0.902741089
con3	5130222	2607723	1.967318615		1.147221676
CREM1	4088771	2222077	1.840067198		0.935317332
CREM2	2882804	1812270	1.590714408		0.976392041
CREM3	2979360	1975339	1.50827782		0.97429568

	Na	gap			
Con4	2592289	2561707	1.011938133	0.900050636	1.124312447
con5	2722441	2695331	1.010058134		1.122223677
con6	1980226	2920017	0.678155641		0.753463876
CREM4	2642558	3291904	0.802744551		1.183717281
CREM5	3143849	3415059	0.920584095		0.909723692
CREM6	2952344	3618795	0.815836211		0.807712134

Nav1.5/GAPDH	Con(7 month)	CREM(7 month)
	0.950037235	0.935317332
	0.902741089	0.976392041
	1.147221676	0.97429568
	1.124312447	1.183717281
	1.122223677	0.909723692
	0.753463876	0.807712134

Figure S4F：CREM mice （7month）(n = 6)

Kir 2.1：96k Da

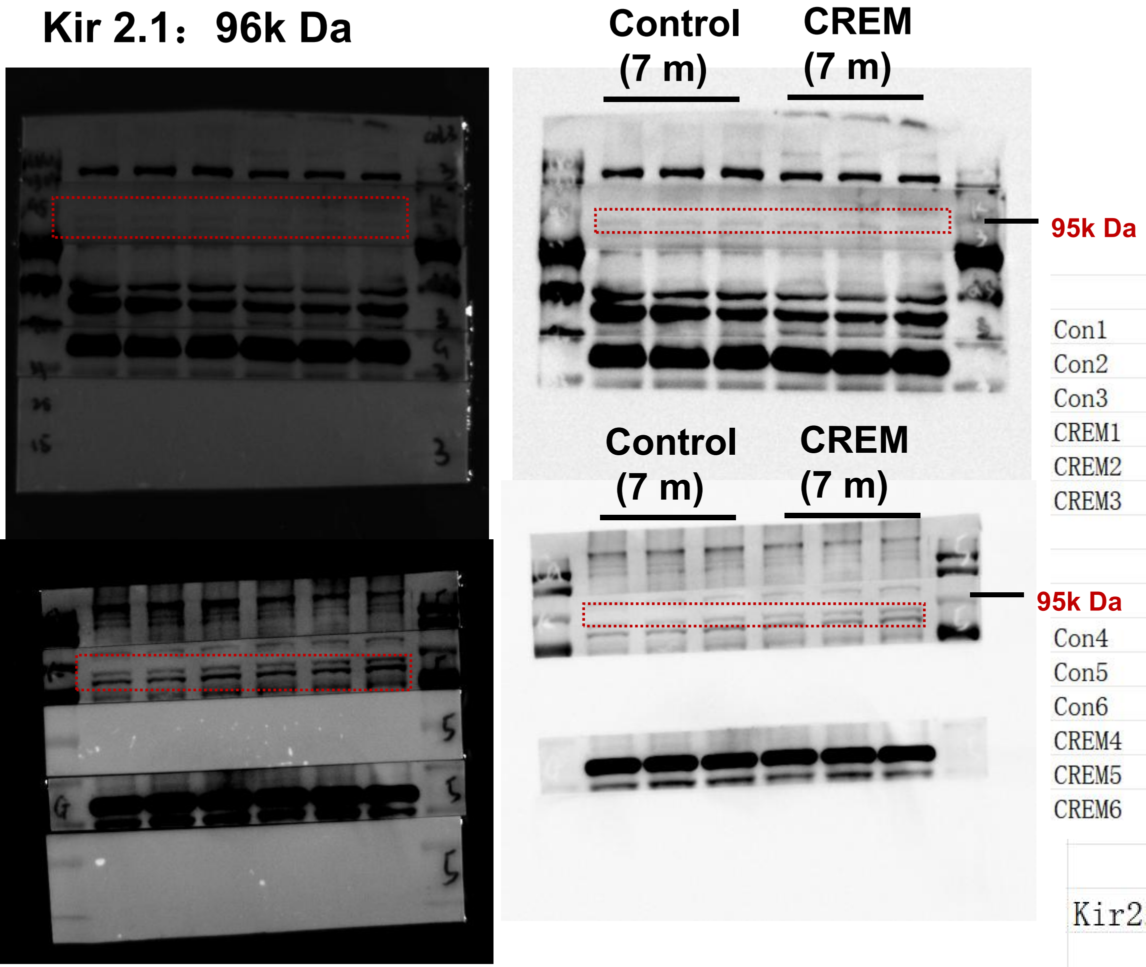

	Kir 2.1	Gap			
Con1	12944.54	25314.225	0.511354	0.440909959	1.159770462
Con2	10254.37	25682.418	0.399276		0.905572196
Con3	10713.95	25998.447	0.4121		0.934657342
CREM1	8989.075	29178.154	0.308076		0.771585758
CREM2	14088.37	28147.447	0.50052		0.978812991
CREM3	12790.88	29328.64	0.436123		1.058293765

	Kir 2.1	Gap			
Con4	11586.84	14633.205	0.791818	0.876751517	0.903127496
Con5	12825.89	13156.477	0.974873		1.111914536
Con6	12102.79	14014.941	0.863563		0.984957968
CREM4	10829.08	13527.305	0.800535		0.821168849
CREM5	10988.01	14144.234	0.776855		0.981101866
CREM6	13641.03	13829.376	0.986381		1.142221817

Kir2.1/GAPDH	Con （7 month）	CREM （7 month）
	1.159770462	0.771585758
	0.905572196	0.978812991
	0.934657342	1.058293765
	0.903127496	0.821168849
	1.111914536	0.981101866
	0.984957968	1.142221817

Figure S4F：CREM mice （7month）(n = 6)

Cav 1.2：239k Da

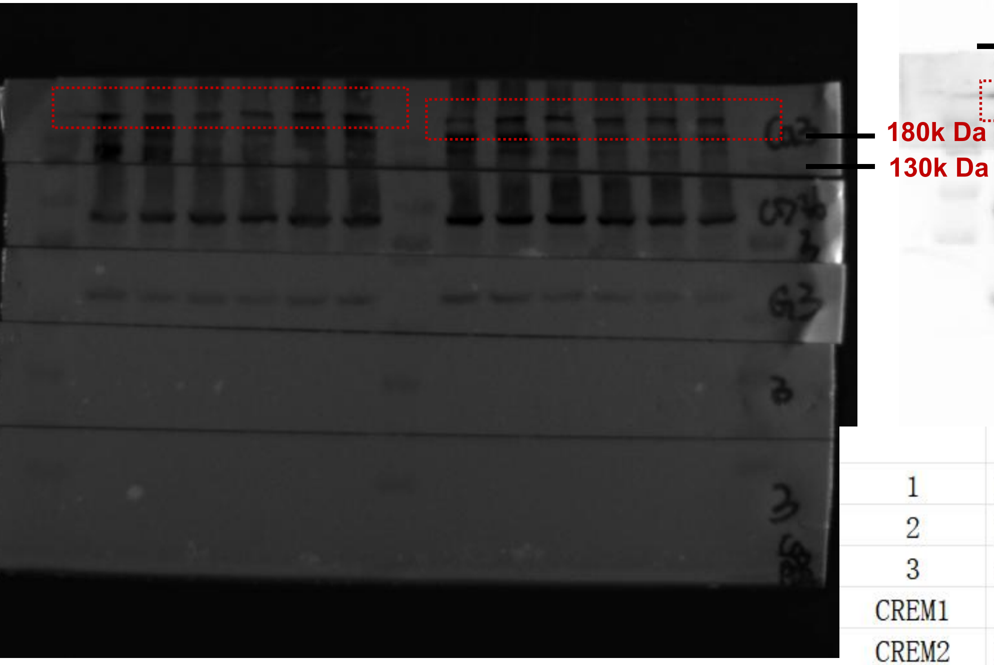

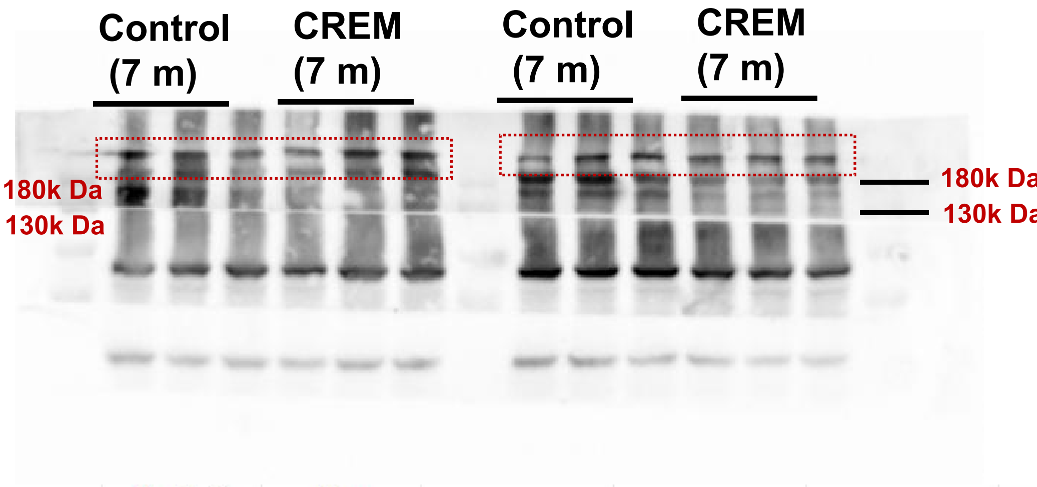

	Cav1. 2	Gap			
1	16920. 296	27402. 418	0. 61747456	0. 636356239	0. 970328445
2	16055. 347	20234. 711	0. 793455711		1. 246873468
3	11286. 933	22658. 225	0. 498138446		0. 782798087
CREM1	10494. 64	20215. 225	0. 519145347		1. 042170808
CREM2	18149. 953	23982. 296	0. 756806312		0. 953810404
CREM3	16300. 004	23446. 246	0. 695207412		1. 125888347
	Ca	Gap			
4	14333. 175	24359. 518	0. 588401421	0. 71755256	0. 820011597
5	20879. 539	24436. 296	0. 854447785		1. 190780763
6	16059. 125	22624. 589	0. 709808474		0. 98920764
CREM4	16654. 489	19975. 539	0. 833744161		1. 174604405
CREM5	15706. 296	18109. 933	0. 867275213		1. 015012535
CREM6	14453. 69	18139. 175	0. 796821796		1. 354214602

Cav1. 2/GAPDH	Con	CREM
	0. 970328445	1. 042170808
	1. 246873468	0. 953810404
	0. 782798087	1. 125888347
	0. 820011597	1. 174604405
	1. 190780763	1. 015012535
	0. 98920764	1. 354214602

Figure S5B：CREM mice （5month）(n = 6)

Col3：37k Da

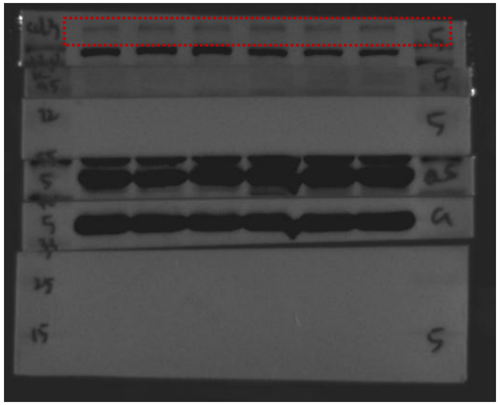

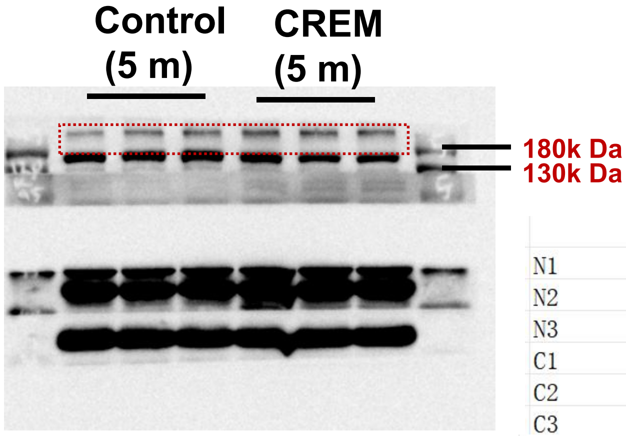

	col3	gap			
N1	21142. 64	29673. 93	0. 712498744	0. 699071775	1. 019206854
N2	19840. 13	27817. 4	0. 71322723		1. 020248929
N3	18538. 71	27608. 35	0. 671489351		0. 960544217
C1	17731. 37	27194. 47	0. 652021139		0. 915119001
C2	21552. 76	26695. 64	0. 80735135		1. 131969331
C3	19346. 66	30846. 15	0. 627198451		0. 934040801

	col3	gap			
N1	22204. 59	20310. 25	1. 093270313	1. 047609325	1. 043585893
N2	21969. 13	23489. 47	0. 935275546		1. 063642801
N3	23159. 76	20784. 47	1. 114282117		0. 892771306
C1	23176. 47	19804. 83	1. 1702431		1. 070405998
C2	26070. 71	18028. 83	1. 446056572		1. 297747267
C3	24238. 76	25226. 47	0. 960846401		1. 027340452

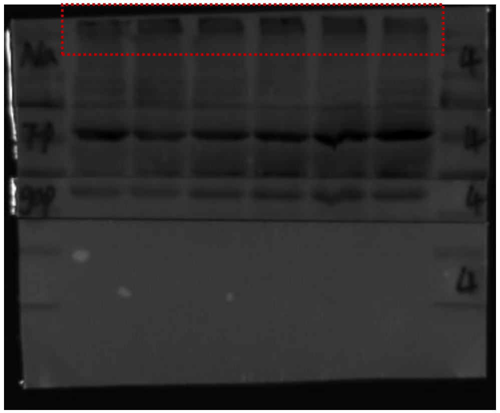

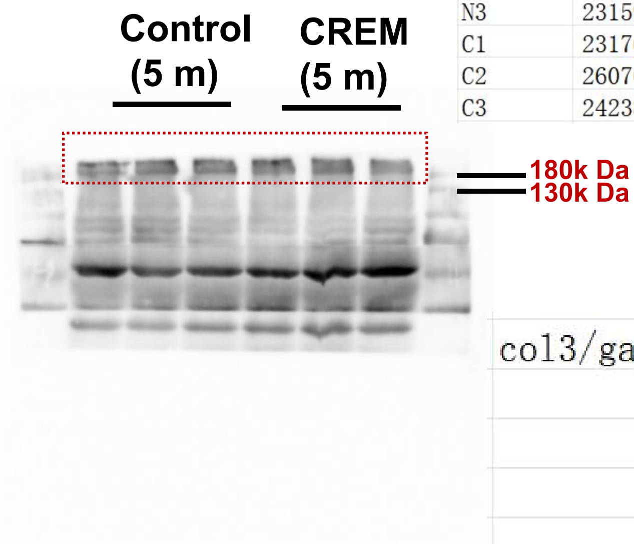

col3/gapdh	CON	CREM
	1. 019206854	0. 915119001
	1. 020248929	1. 131969331
	0. 960544217	0. 934040801
	1. 043585893	1. 070405998
	1. 063642801	1. 297747267
	0. 892771306	1. 027340452

Figure S5B：CREM mice （5month）(n = 6)

α-SMA：42k Da

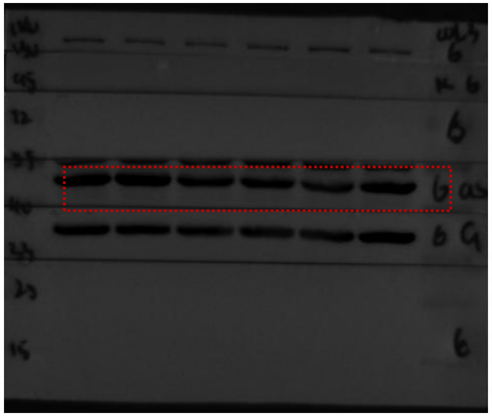

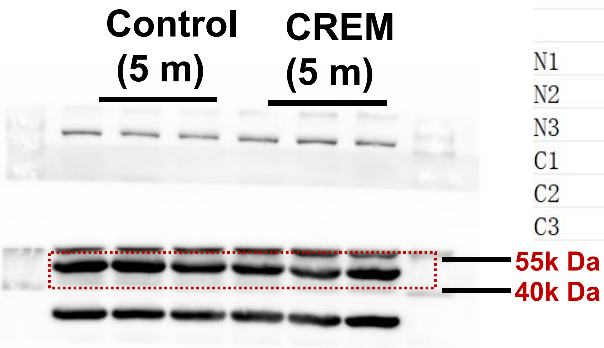

	asma	gap			
N1	29609.25	29673.93	0.997820073	1.010674212	0.98728162
N2	29909.69	27817.4	1.075215269		1.063859408
N3	26476.05	27608.35	0.958987295		0.948858973
C1	25638.64	27194.47	0.942788805		0.944848506
C2	23392.13	26695.64	0.876252639		0.913727058
C3	29100.3	30846.15	0.943401113		0.877406729

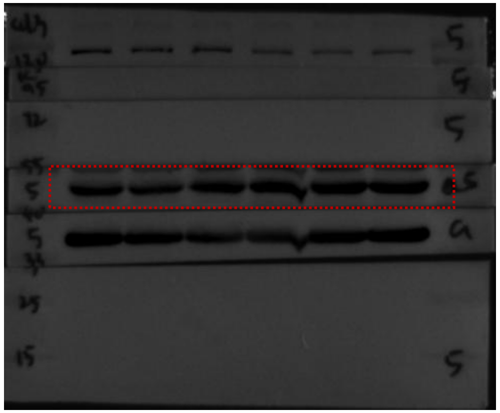

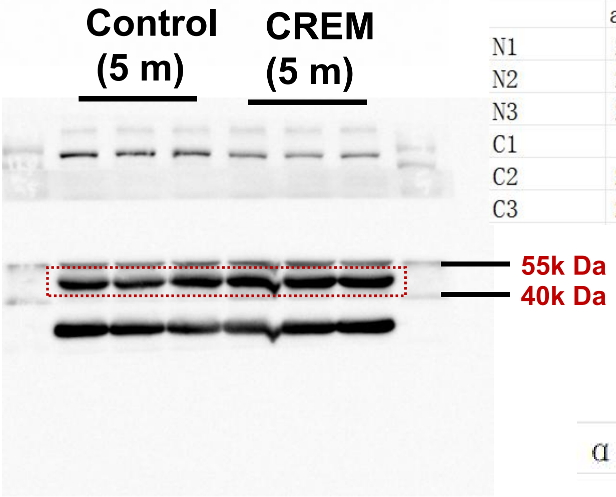

	asma	gap			
N1	25137.47	29631.983	0.848322166	0.876298631	0.968074279
N2	22564.93	27380.033	0.824138269		0.940476499
N3	24284.28	25390.397	0.956435459		1.091449221
C1	27681.4	29665.861	0.933106138		1.09994313
C2	26723.91	26914.548	0.99291699		1.038143224
C3	26576.28	29098.711	0.913314545		1.108205479

α-SMA/gapdh	CON	CREM
	0.98728162	1.09994313
	1.063859408	1.038143224
	0.948858973	1.108205479
	0.968074279	0.944848506
	0.940476499	0.913727058
	1.091449221	0.877406729

Figure S5B：CREM mice （5month）(n = 6)

TGF-β1：55k Da

Control (5 m) | CREM (5 m)

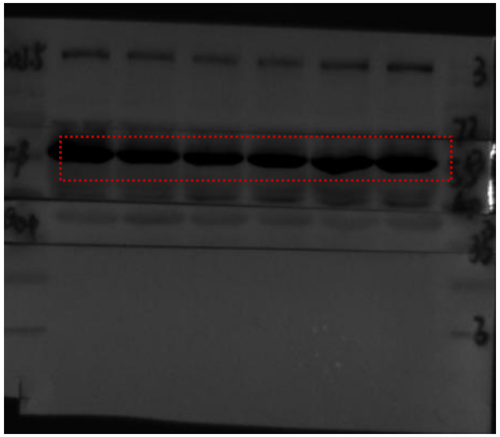

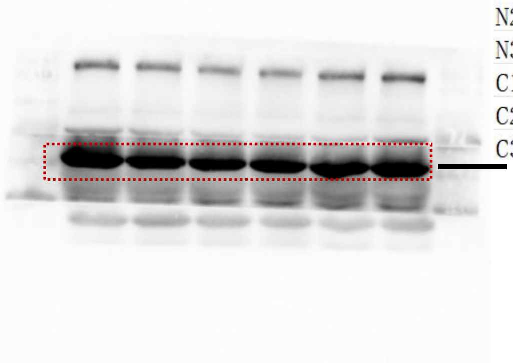

	tgfb	gap			
N1	31188. 42	20310. 246	1. 535600209	1. 237225106	1. 241164766
N2	25476. 45	23489. 468	1. 084590209		0. 876631264
N3	22685. 93	20784. 468	1. 091484901		0. 882203971
C1	23342. 69	19804. 832	1. 178636103		1. 079846456
C2	25418. 23	18028. 832	1. 40986532		0. 918120037
C3	25755. 93	25226. 468	1. 020988471		0. 941358739

Control (5 m) | CREM (5 m)

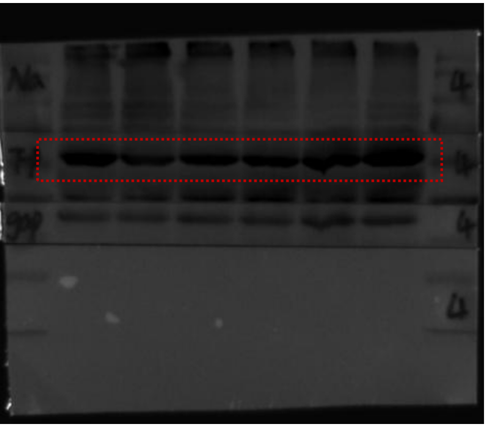

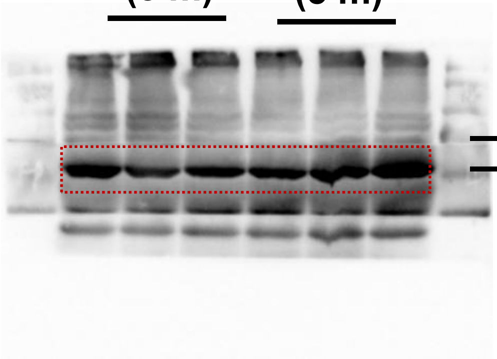

	tgfb	gap			
N1	19866. 35	14463. 13	1. 373586068	1. 217369685	1. 128322879
N2	15041. 59	12493. 25	1. 203977653		0. 988999207
N3	18938. 81	17624. 95	1. 074545334		0. 882677914
C1	21996. 3	20048. 83	1. 097136033		0. 911259465
C2	27772. 59	26263. 83	1. 057446187		0. 984087087
C3	31045. 52	20650. 13	1. 503405815		1. 094511549

TGF-β 1/gapdh	CON	CREM
	1. 128322879	0. 911259465
	0. 988999207	0. 984087087
	0. 882677914	1. 094511549
	1. 241164766	1. 079846456
	0. 876631264	0. 918120037
	0. 882203971	0. 941358739

Figure S5D：CREM mice （5month）(n = 6)

Nav 1.5：226k Da

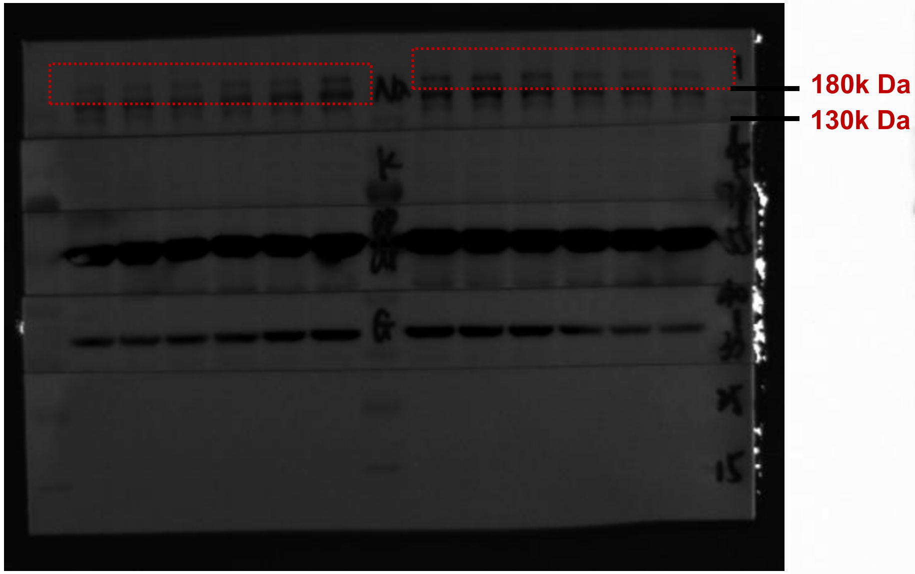

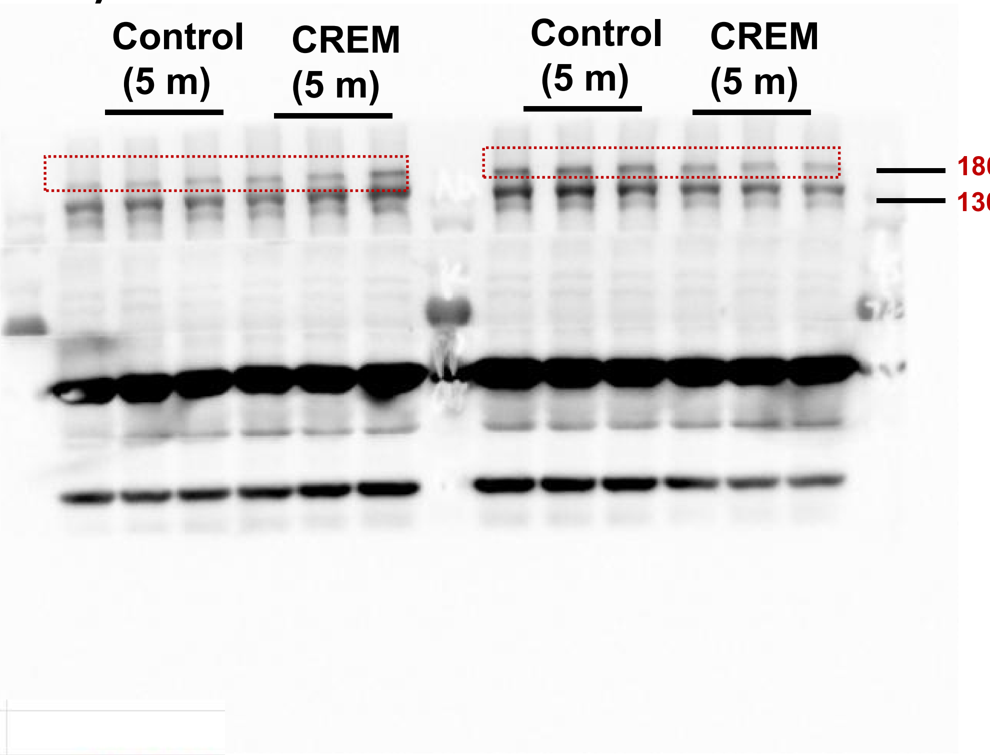

	Na	GAP			
C1	16012. 18	22614. 76	0. 708040868	0. 667962204	1. 060001394
C2	16833. 42	24811. 91	0. 678440984		1. 015687684
C3	17215. 66	27883. 91	0. 61740476		0. 924310921
CREM1	17996. 08	29206. 4	0. 616168951		0. 997998381
CREM2	19302. 66	28475. 74	0. 677863332		0. 957378822
CREM3	21317. 42	33949. 74	0. 627911083		0. 925520564

	NA	gap			
C4	21537. 468	31331. 49	0. 687406462	0. 728149428	0. 944045872
C5	21639. 368	29078. 66	0. 744166616		1. 021997117
C6	19972. 61	26528. 45	0. 752875206		1. 033957011
CREM4	17765. 782	24421. 03	0. 727478727		0. 966267346
CREM5	16074. 125	23327. 15	0. 689073558		0. 92596677
CREM6	15814. 054	23822. 64	0. 66382458		0. 965694414

Figure S5D：CREM mice （5month）(n = 6)

Kir 2.1：96k Da

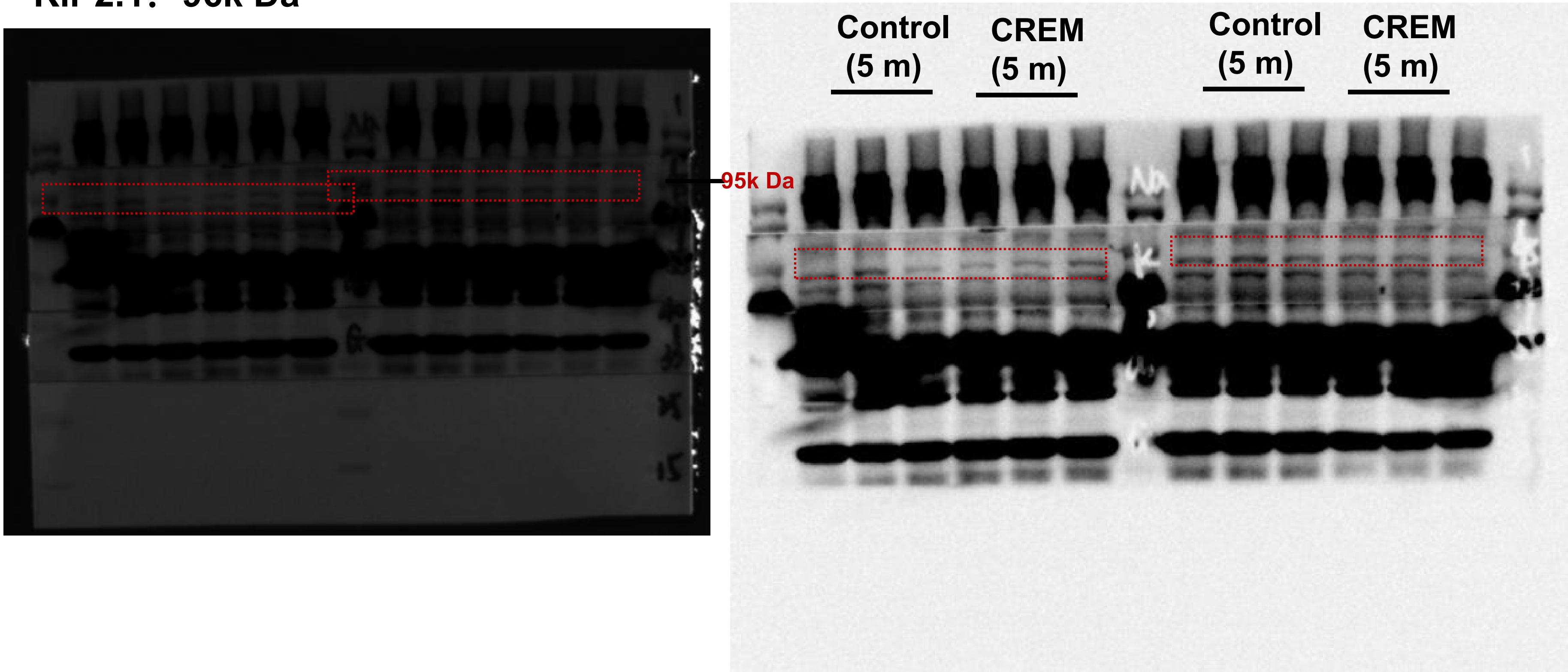

	K	GAP			
C1	13449. 05	22614. 76	0. 59470246	0. 653654118	0. 909812152
C2	19513. 59	24811. 91	0. 786460511		1. 203175333
C3	16167. 08	27883. 91	0. 579799384		0. 887012516
CREM1	19349. 54	29206. 4	0. 662510306		1. 14265438
CREM2	23659. 78	28475. 74	0. 830875054		1. 056473964
CREM3	26427	33949. 74	0. 778415505		1. 308915898

	K	gap			
C4	22924. 004	31331. 489	0. 731660216	0. 83579557	0. 875405711
C5	25416. 66	29078. 66	0. 874065724		1. 045788892
C6	23919. 66	26528. 447	0. 901660772		1. 078805397
CREM4	19300. 368	24421. 033	0. 790317428		0. 876513045
CREM5	17873. 539	23327. 154	0. 766211729		0. 876606539
CREM6	17213. 468	23822. 64	0. 722567608		0. 987572636

Figure S5D：CREM mice （5month）(n = 6)

Cav 1.2：239k Da

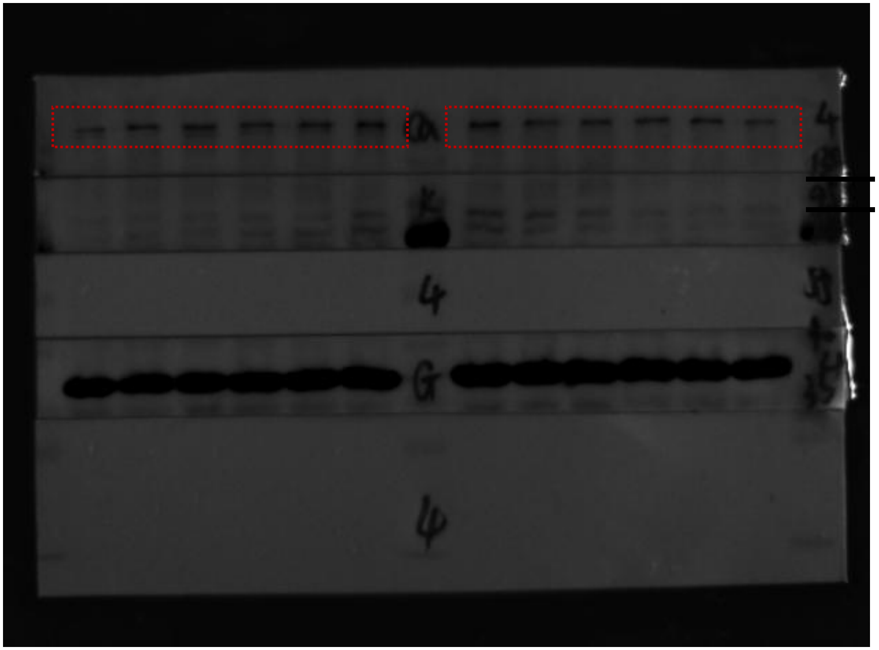

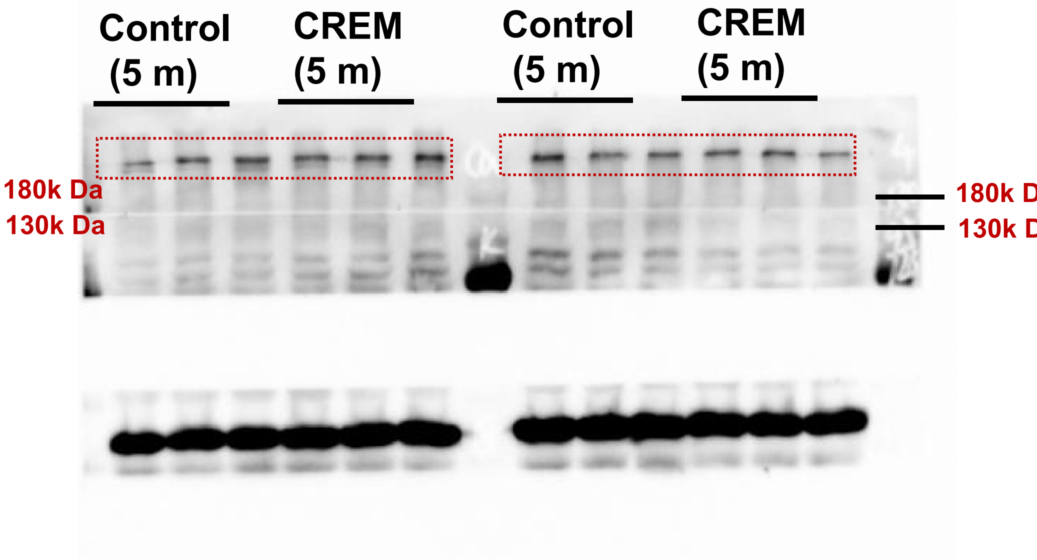

	Ca	Gap			
c1	10239.18	22962.54	0.445908	0.538804	0.827588
c2	13247.08	24236.47	0.546576		1.014424
c3	15908.71	25497.62	0.623929		1.157988
CREM1	15811.49	28383.52	0.557066		1.019192
CREM2	15922.13	28592.45	0.556865		1.248834
CREM3	18344.47	31726.3	0.57821		0.926724
	Ca	Gap			
C4	20759.78	30654.02	0.677229	0.588363	1.151039
C5	15497.25	26568.4	0.583296		0.991389
C6	14480.59	28699.23	0.504564		0.857572
CREM4	15658.61	28358.54	0.552166		0.94663
CREM5	15270.3	23852.23	0.640204		0.94533
CREM6	10759.76	25459.9	0.422616		0.837587

Figure S6B：(n = 6)

HK：102k Da

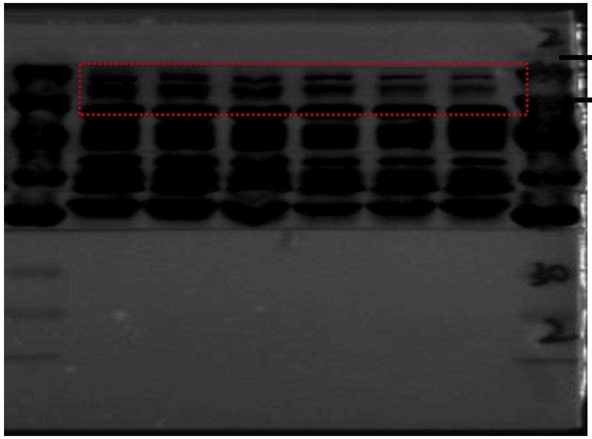

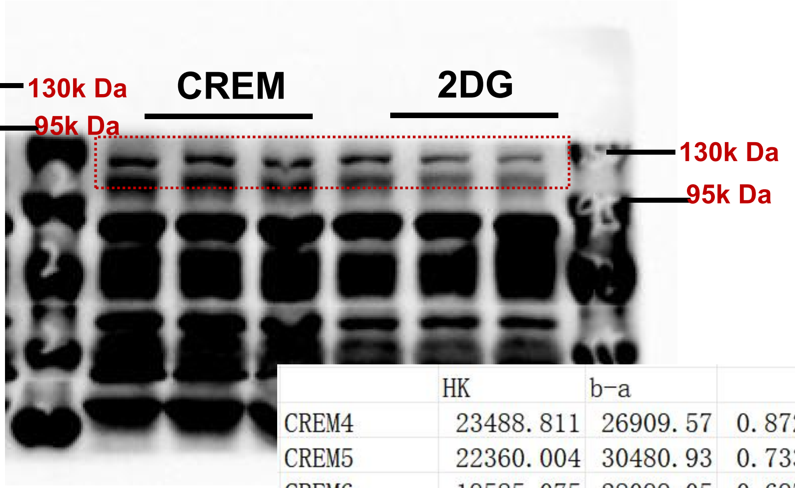

	HK	b-a			
CREM4	23488.811	26909.57	0.872879495	0.767818065	1.136831152
CREM5	22360.004	30480.93	0.733573477		0.955400127
CREM6	19585.075	28099.05	0.697001223		0.907768721
CREM_2DG6	19100.489	21340.64	0.895028874		1.025375071
CREM-2DG5	12953.054	23793.25	0.544400457		0.742121238
CREM-2DG4	10075.497	25454.1	0.395829961		0.567904256

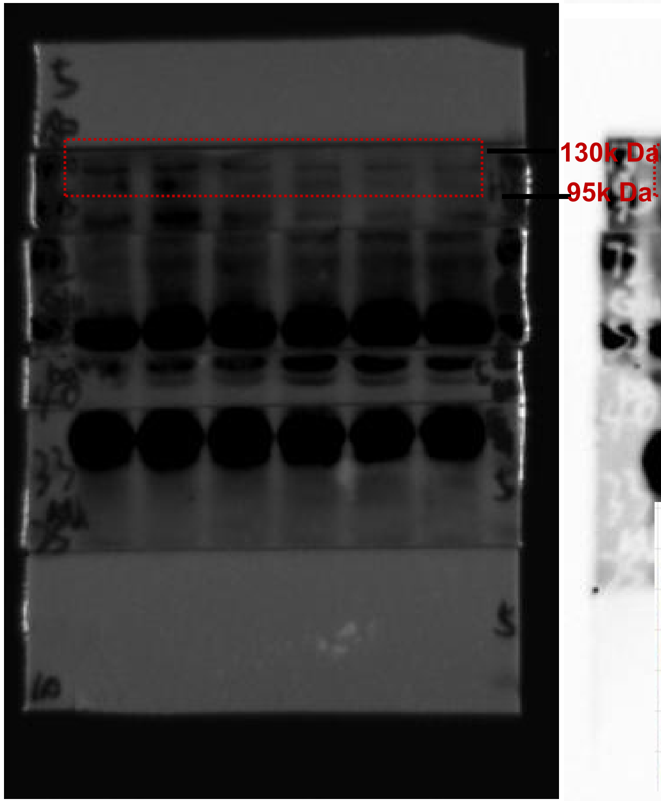

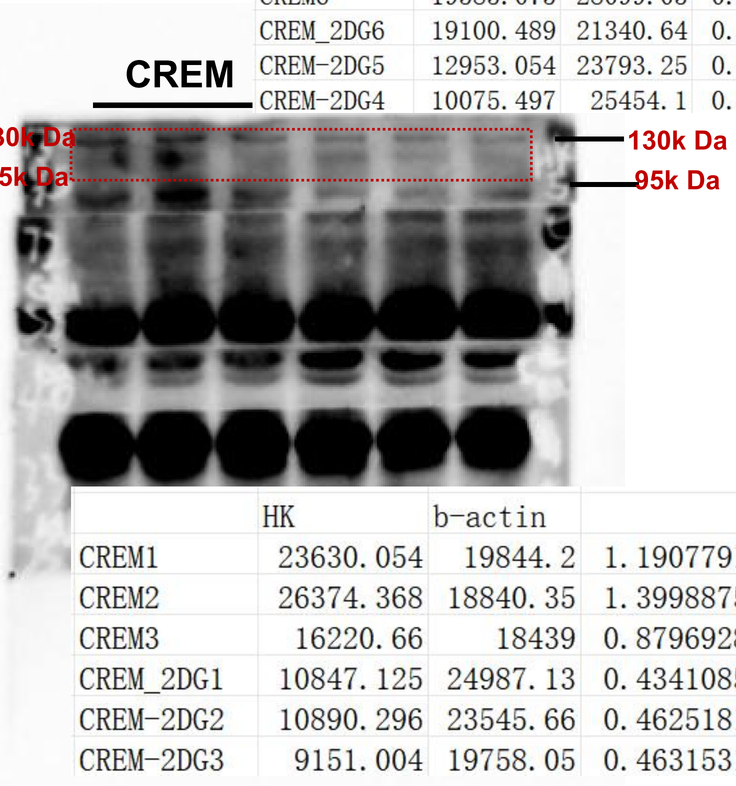

	HK	b-actin			
CREM1	23630.054	19844.2	1.190779107	1.156786517	1.029385362
CREM2	26374.368	18840.35	1.399887592		1.210152065
CREM3	16220.66	18439	0.879692851		0.760462573
CREM_2DG1	10847.125	24987.13	0.434108566		0.493477428
CREM-2DG2	10890.296	23545.66	0.462518188		0.388416445
CREM-2DG3	9151.004	19758.05	0.463153102		0.330850209

Figure S6B：(n = 6)

PFKM：85k Da

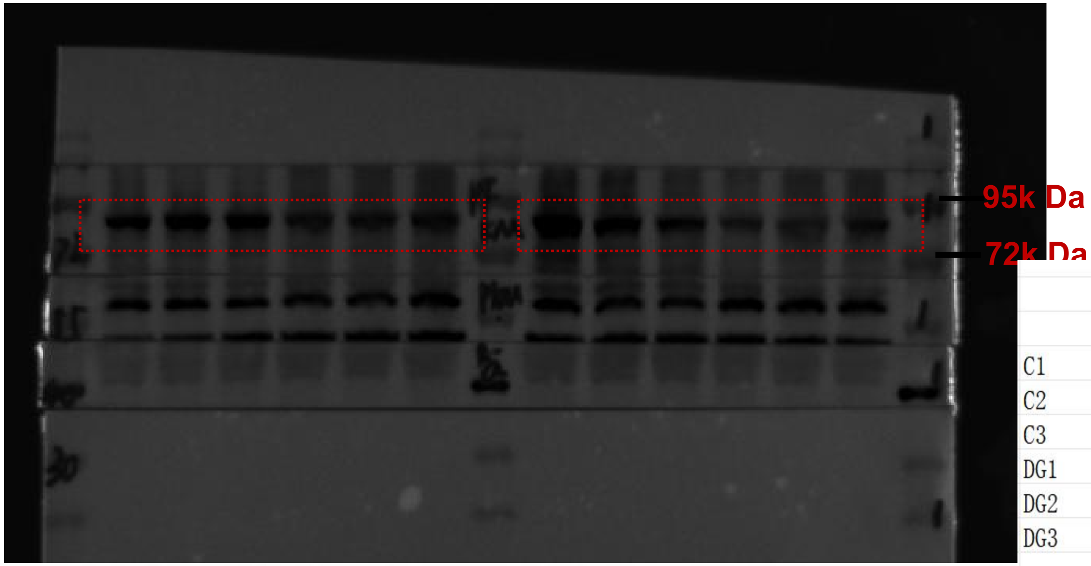

	PFKM	b-a			
C1	20744.004	21659.811	0.957718606	1.042076044	0.919048674
C2	24509.418	20780.246	1.179457548		1.131834433
C3	22867.125	23120.246	0.989051976		0.949116893
DG1	19510.418	23662.347	0.824534354		0.833661298
DG2	19889.347	22161.539	0.89747138		0.760918765
DG3	19475.447	24263.125	0.802676778		0.83811338

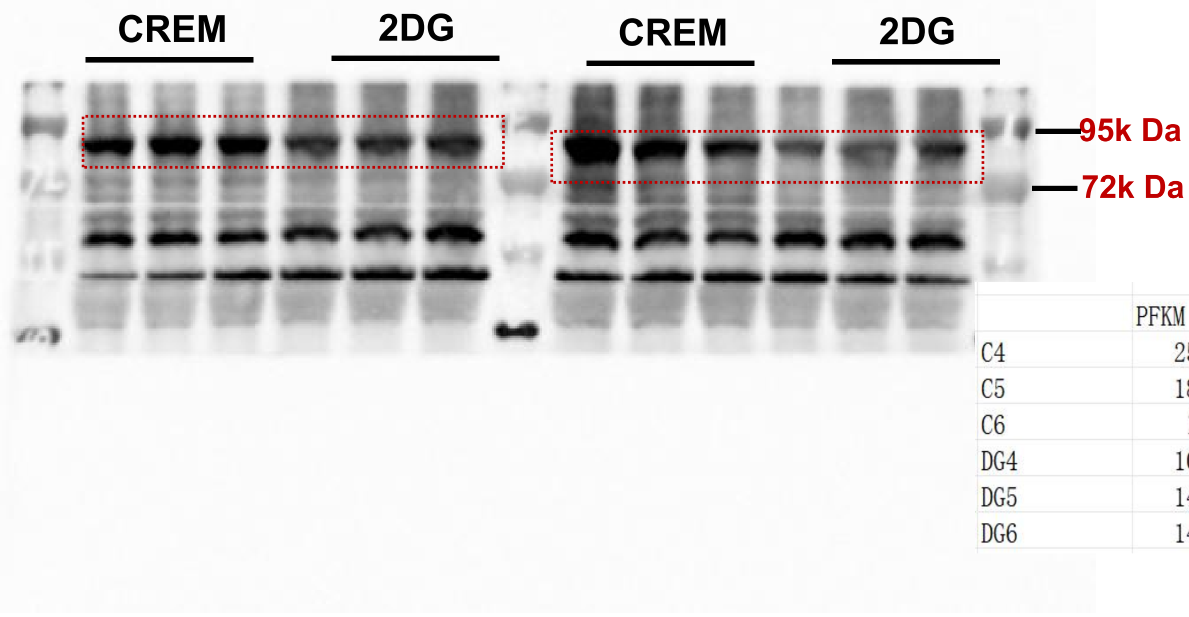

	PFKM	b-a			
C4	25676.054	25687.075	0.999570952	0.823167096	1.214298964
C5	18812.861	25317.953	0.743064062		0.902689218
C6	16430.69	22604.832	0.726866274		0.883011818
DG4	10631.326	19617.711	0.541924896		0.745563408
DG5	14600.296	22902.539	0.637496829		0.857929837
DG6	14679.619	20760.69	0.70708724		0.707390745

Figure S6B：(n = 6)

PKM：60k Da

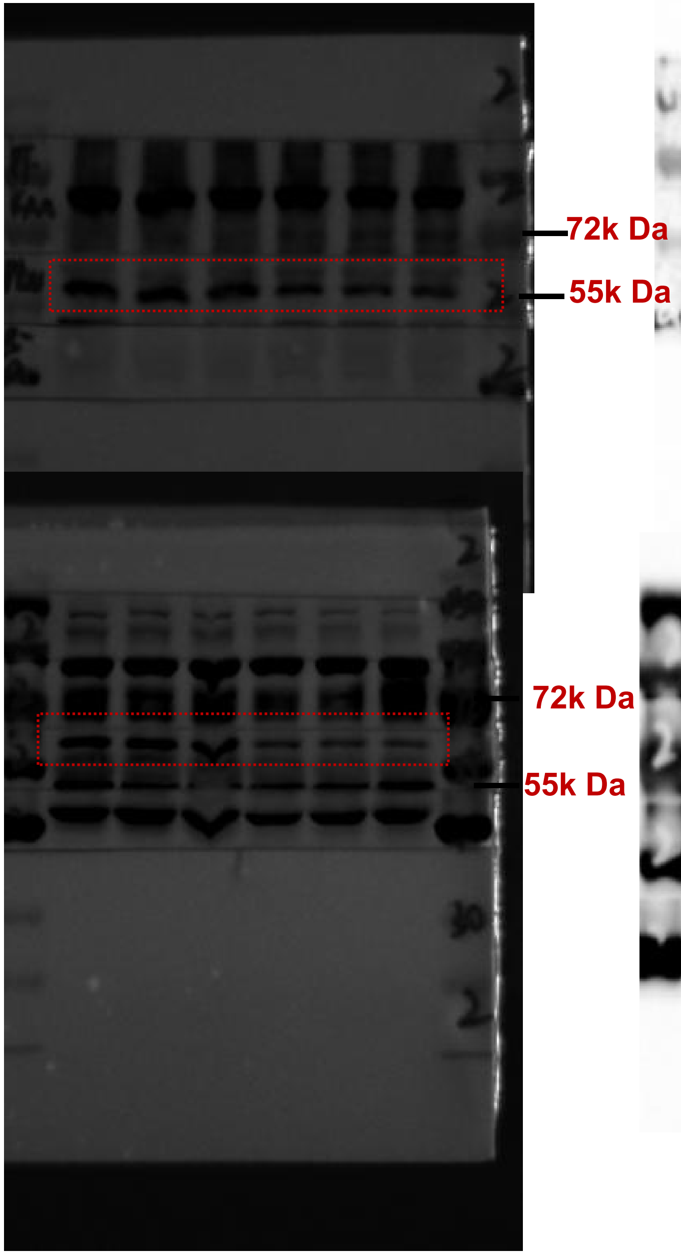

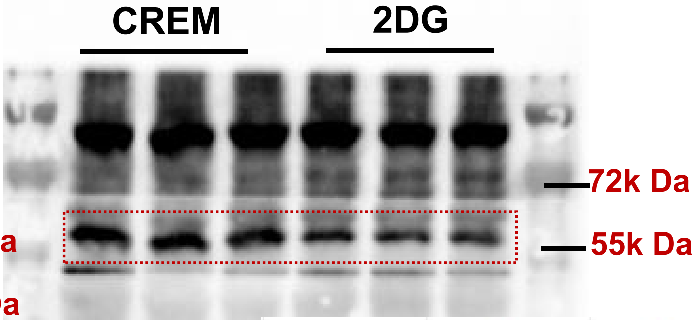

	PKM	b-a			
C1	26881.246	24005.347	1.119802434	1.200787559	0.932556659
C2	25505.296	20793.953	1.226572744		1.021473561
C3	24297.882	19345.64	1.255987499		1.04596978
DG1	19154.64	22300.004	0.858952312		0.683886036
DG2	18225.832	24918.368	0.731421576		0.653170197
DG3	17849.861	22380.225	0.797572902		0.650245088

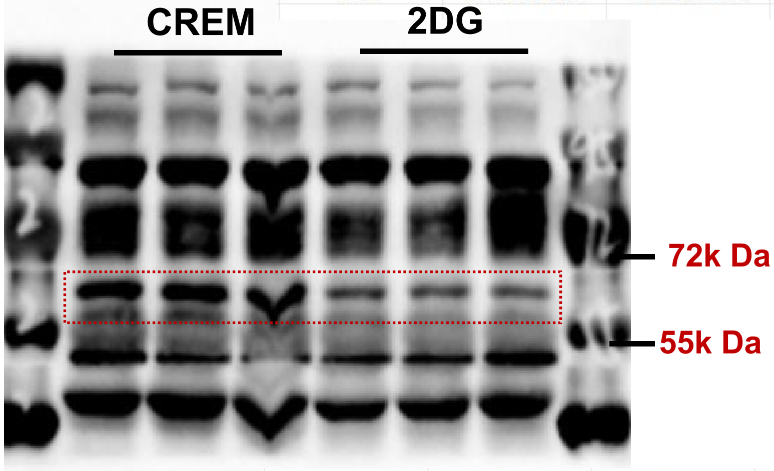

	PKM	b-a			
C4	26651.539	26909.569	0.990411218	0.890577836	1.112099558
C5	26229.832	30480.933	0.860532452		0.966263045
C6	23063.418	28099.054	0.820789839		0.921637397
DG4	17515.539	21340.64	0.82075978		0.828706062
DG5	15859.296	23793.246	0.666546128		0.774574076
DG6	14561.983	25454.104	0.572087825		0.69699672

Figure S6B：(n = 6)

LDHA：37k Da

	LDHA	b-actin			
C1	28303.104	19844.2	1.426266098	1.490685242	0.956785549
C2	28494.69	18840.35	1.51242915		1.014586518
[illegible]	[illegible]64	18439	1.533360479		1.028627932
[illegible]	[illegible]25	24987.13	1.122507091		0.787025011
[illegible]	[illegible]69	23545.66	1.147963956		0.759019988
[illegible]	[illegible]64	19758.05	1.438989892		0.938455055

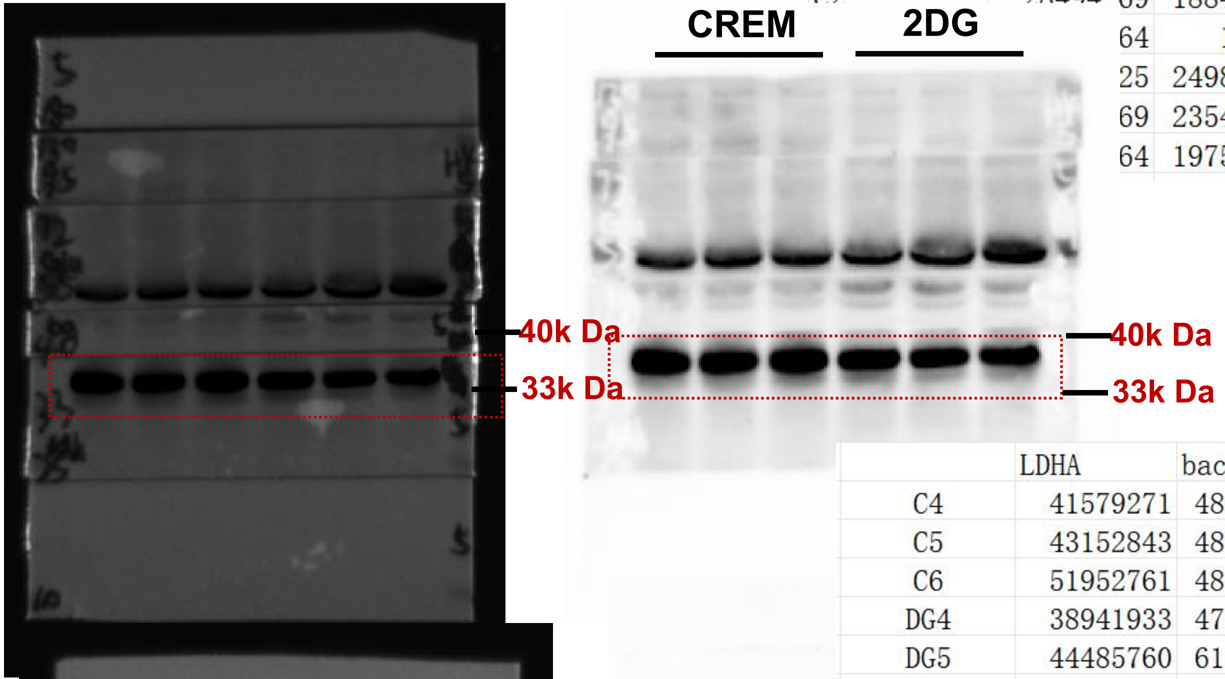

	LDHA	bactin			
C4	41579271	48495090	0.857391357	0.940192578	0.911931637
C5	43152843	48541817	0.888982854		0.945532728
C6	51952761	48363983	1.074203525		1.142535635
DG4	38941933	47771037	0.815178724		0.916979131
DG5	44485760	61530467	0.722987524		0.843240976
DG6	35892973	38497793	0.932338459		0.867934649

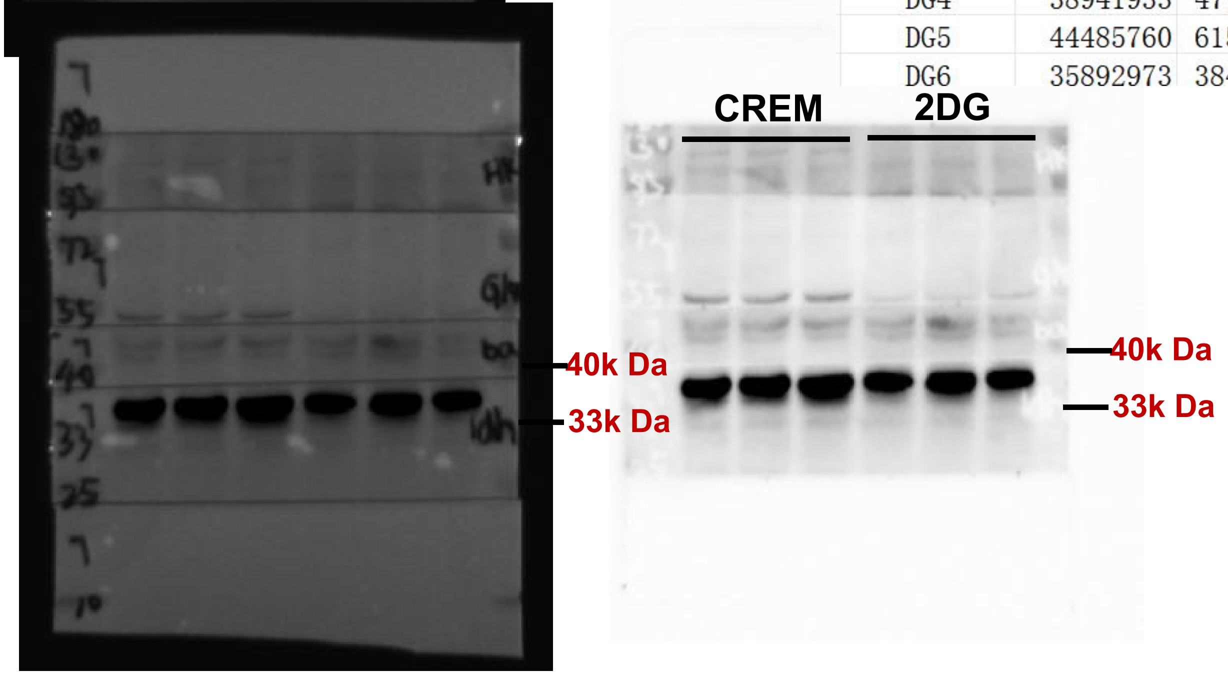

Figure S6J：(n = 6)

Col3：225k Da

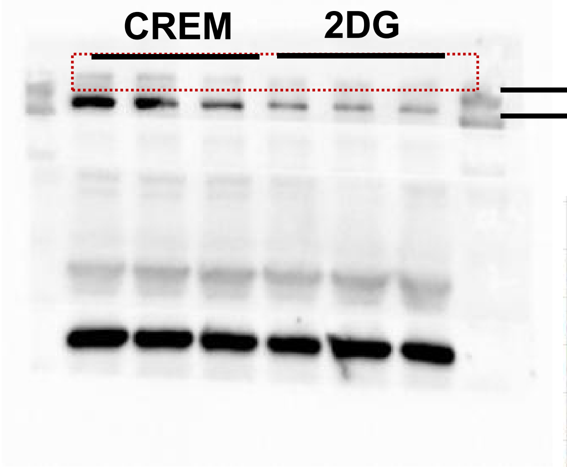

	COL3	gap			
C4	24225. 69	25406. 933	0. 95350706	0. 847075599	1. 125645764
C5	21801. 539	23605. 205	0. 923590327		1. 090328098
C6	15509. 711	23353. 447	0. 664129411		0. 784026138
2DG4	12613. 711	21579. 64	0. 584519065		0. 613020175
SDG5	13825. 075	26806. 418	0. 5157375		0. 776561753
2DG6	12489. 276	22249. 033	0. 561340172		0. 607780479

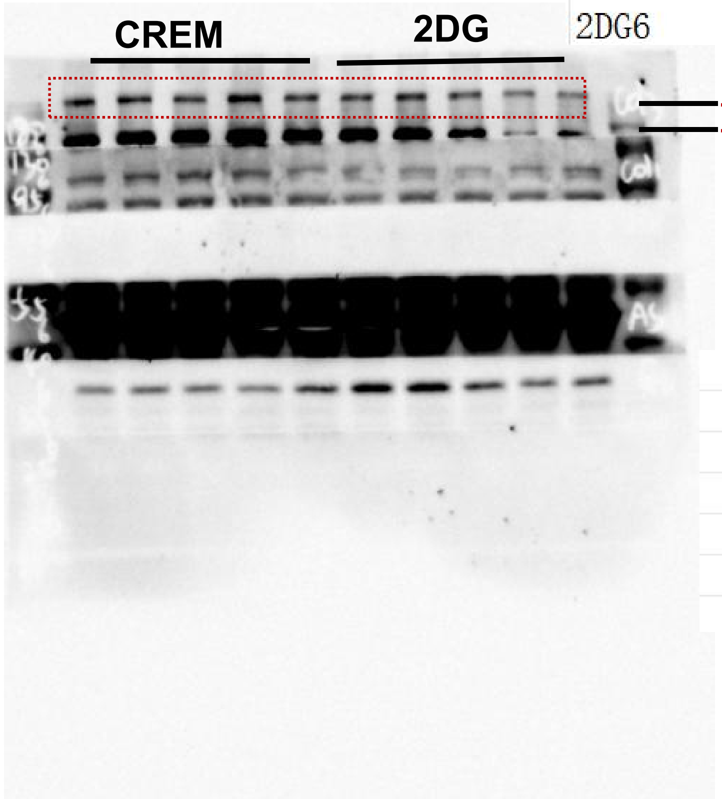

	Col3	gap			
C1	10072. 255	4614. 548	2. 182717571	1. 947249818	1. 120923238
C2	12131. 79	5741. 497	2. 113001191		1. 085120754
C3	9153. 083	5920. 376	1. 546030691		0. 793956008
2DG1	7051. 134	5979. 083	1. 179300237		0. 762792255
2DG2	5829. 669	3485. 841	1. 672385229		0. 79147387
2DG3	6241. 77	4566. 719	1. 366795286		0. 626189711

Figure S6J：(n = 6)

α-SMA：42k Da

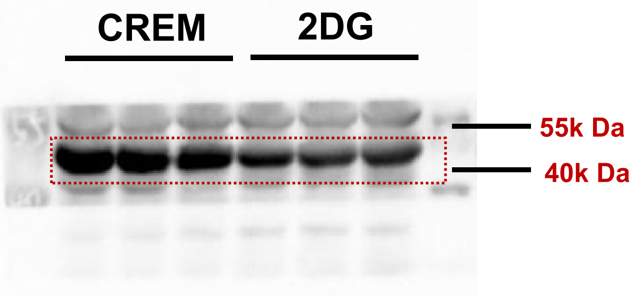

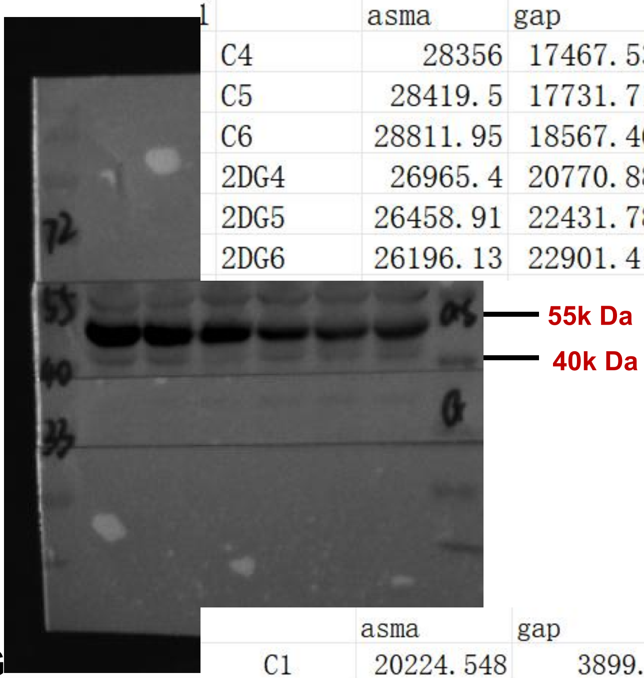

1	asma	gap			
C4	28356	17467.539	1.62335427	1.592615964	1.019300513
C5	28419.5	17731.711	1.60274984		1.006363039
C6	28811.95	18567.468	1.55174378		0.974336448
2DG4	26965.4	20770.882	1.29823072		0.799721133
2DG5	26458.91	22431.782	1.17952787		0.735940093
2DG6	26196.13	22901.418	1.14386476		0.737147958

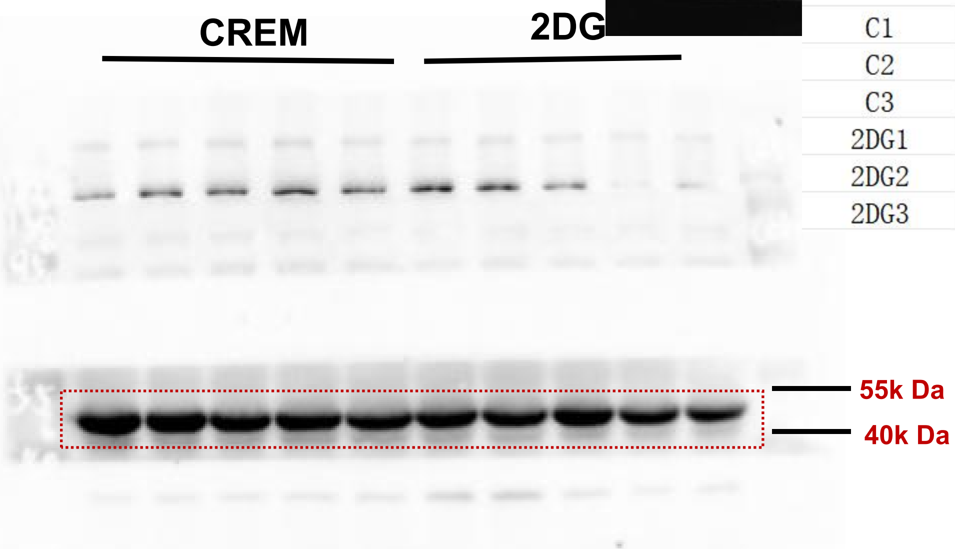

	asma	gap			
C1	20224.548	3899.134	5.186933304	4.147647008	1.250572504
C2	16631.184	4405.841	3.774803494		0.910107221
C3	16064.184	4614.548	3.481204226		0.839320275
2DG1	14892.477	5979.083	2.490762714		0.65983904
2DG2	11529.355	3485.841	3.307481609		0.637656476
2DG3	11204.376	4566.719	2.453484876		0.704780506

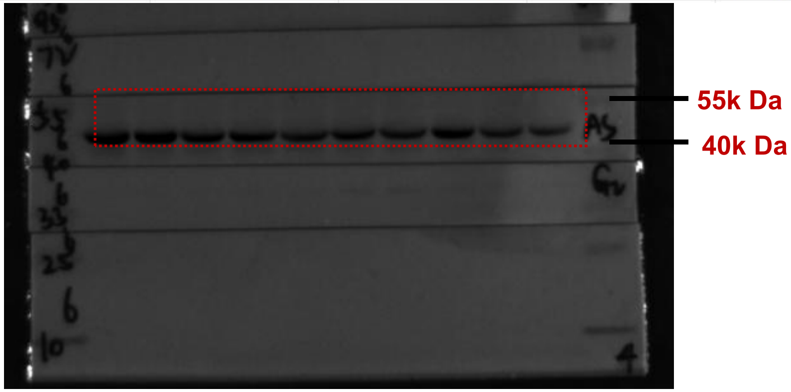

Figure S6J：(n = 6)

TGF-β1 ：55k Da

	T-B	gap			
C1	22358. 83	23870. 69	0. 93666467	0. 907588043	1. 032037254
C2	21801. 49	24752. 569	0. 88077682		0. 970458818
C3	23566. 83	26031. 418	0. 90532264		0. 997503928
2DG1	19187. 37	24073. 054	0. 79704752		0. 850942226
2DG2	17730. 95	23874. 175	0. 74268338		0. 84321404
2DG3	18643. 42	24542. 933	0. 7596247		0. 839065173

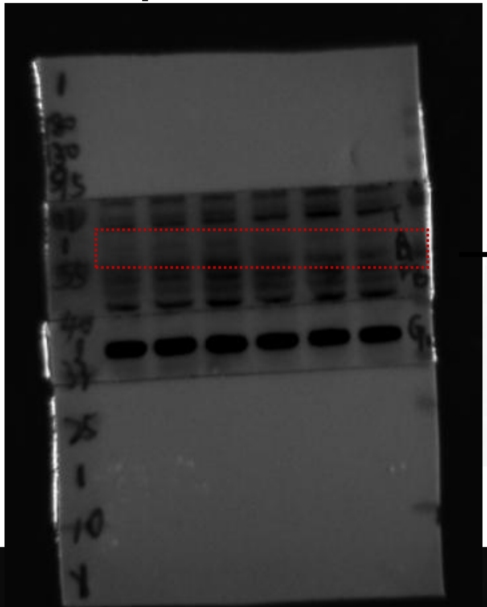

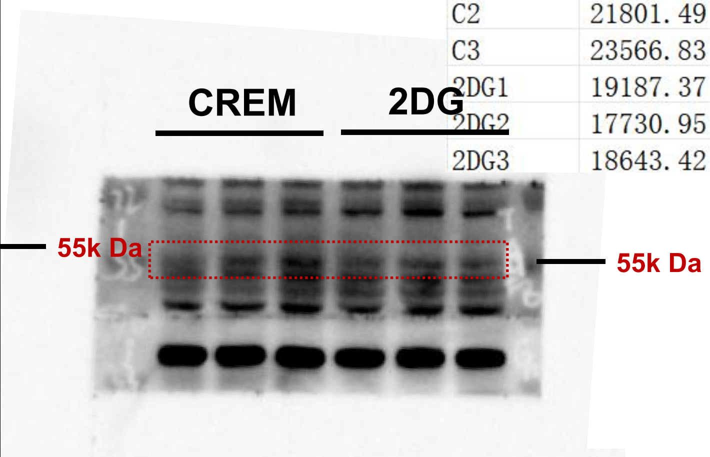

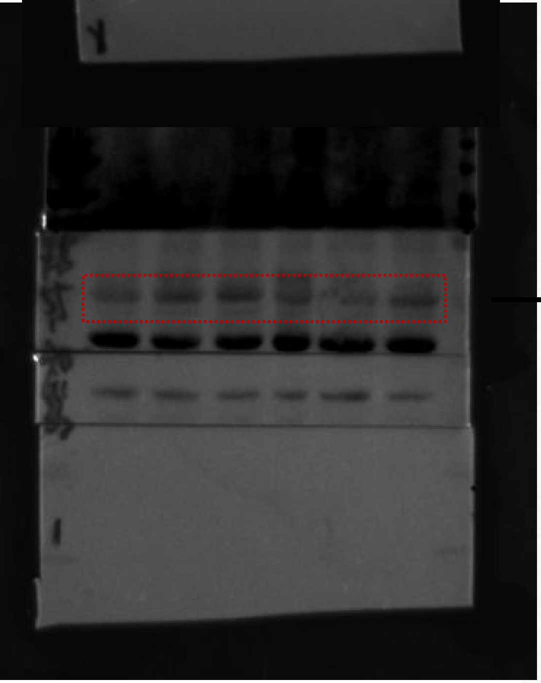

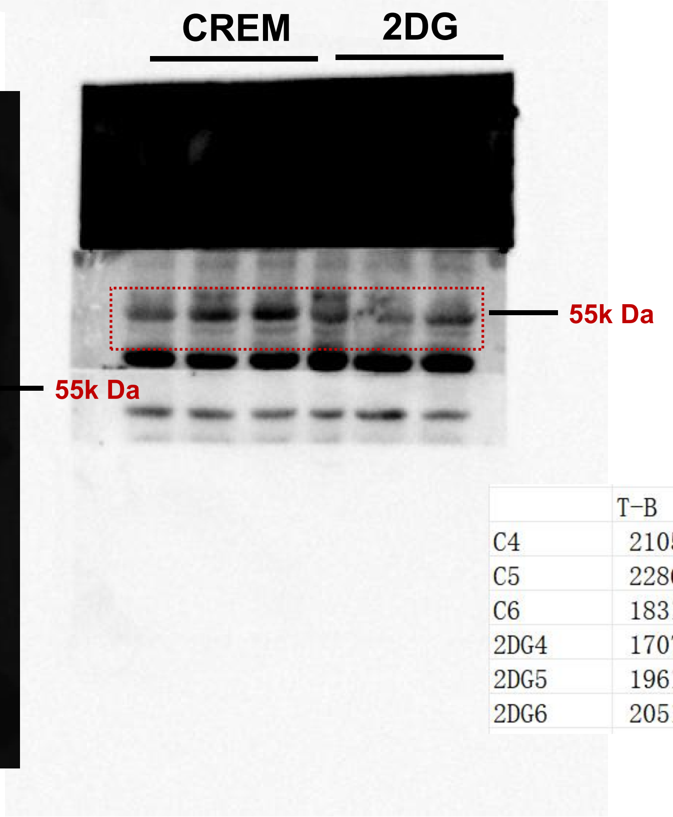

	T-B	gap			
C4	21058. 075	21171. 468	0. 99464407	0. 913067136	1. 089343846
C5	22860. 418	24158. 761	0. 94625788		1. 036350827
C6	18315. 347	22942. 953	0. 79829946		0. 874305327
2DG4	17073. 418	23419. 075	0. 72903896		0. 913239946
2DG5	19610. 368	20795. 832	0. 94299512		0. 948072932
2DG6	20511. 054	23888. 054	0. 85863227		0. 907397746

Figure S6J：(n = 6)

TGF-β1：55k Da

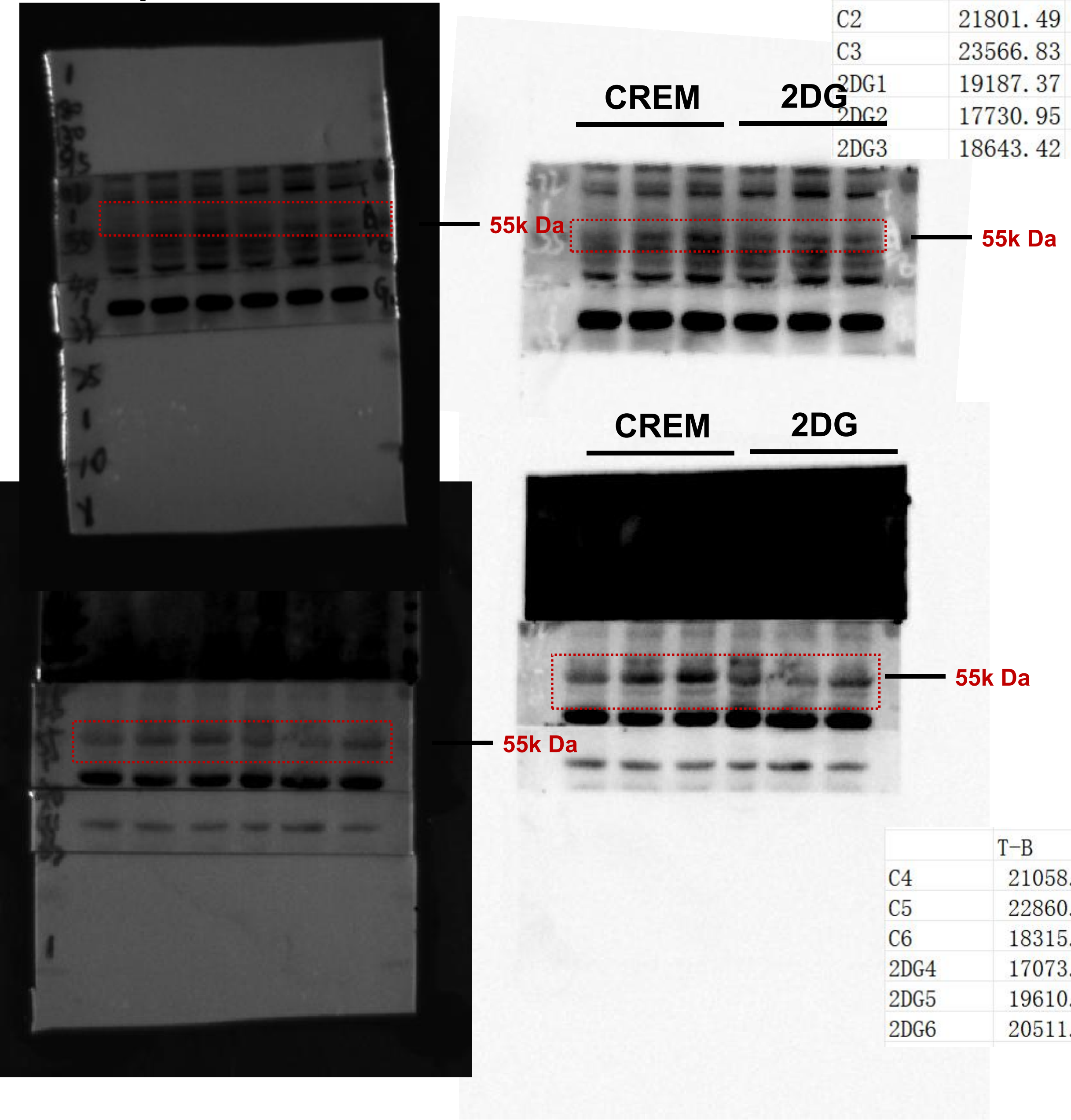

	T-B	gap			
C1	22358. 83	23870. 69	0. 93666467	0. 907588043	1. 032037254
C2	21801. 49	24752. 569	0. 88077682		0. 970458818
C3	23566. 83	26031. 418	0. 90532264		0. 997503928
2DG1	19187. 37	24073. 054	0. 79704752		0. 850942226
2DG2	17730. 95	23874. 175	0. 74268338		0. 84321404
2DG3	18643. 42	24542. 933	0. 7596247		0. 839065173

	T-B	gap			
C4	21058. 075	21171. 468	0. 99464407	0. 913067136	1. 089343846
C5	22860. 418	24158. 761	0. 94625788		1. 036350827
C6	18315. 347	22942. 953	0. 79829946		0. 874305327
2DG4	17073. 418	23419. 075	0. 72903896		0. 913239946
2DG5	19610. 368	20795. 832	0. 94299512		0. 948072932
2DG6	20511. 054	23888. 054	0. 85863227		0. 907397746

Figure S12A：atrial tissues from mice(n = 6)

Pan Kla：17k Da

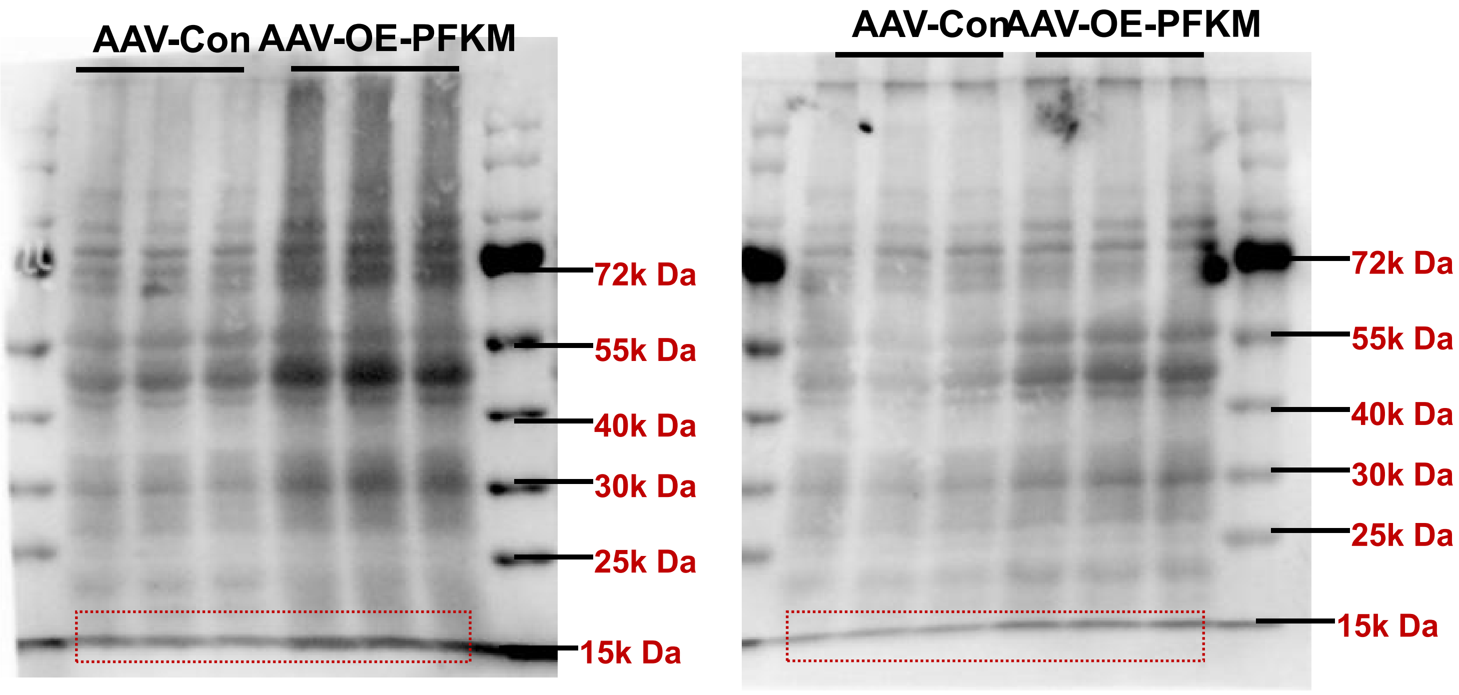

Pan Kla：17k Da

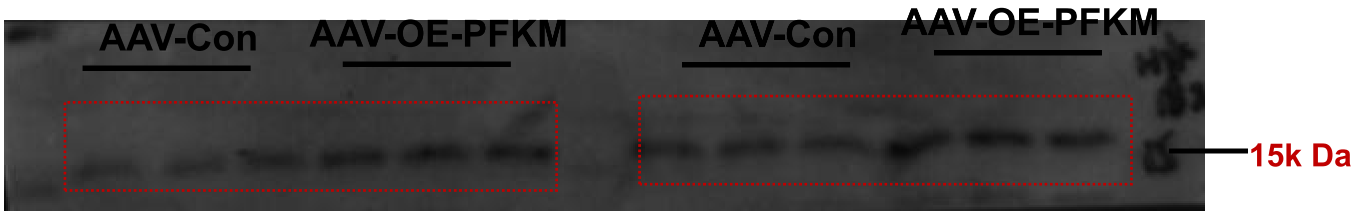

Histone H3：17k Da

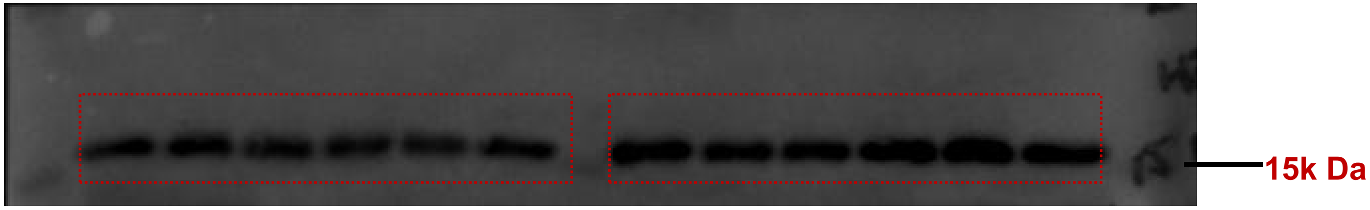

Figure S12B：atrial fibroblasts from mice(n = 3)

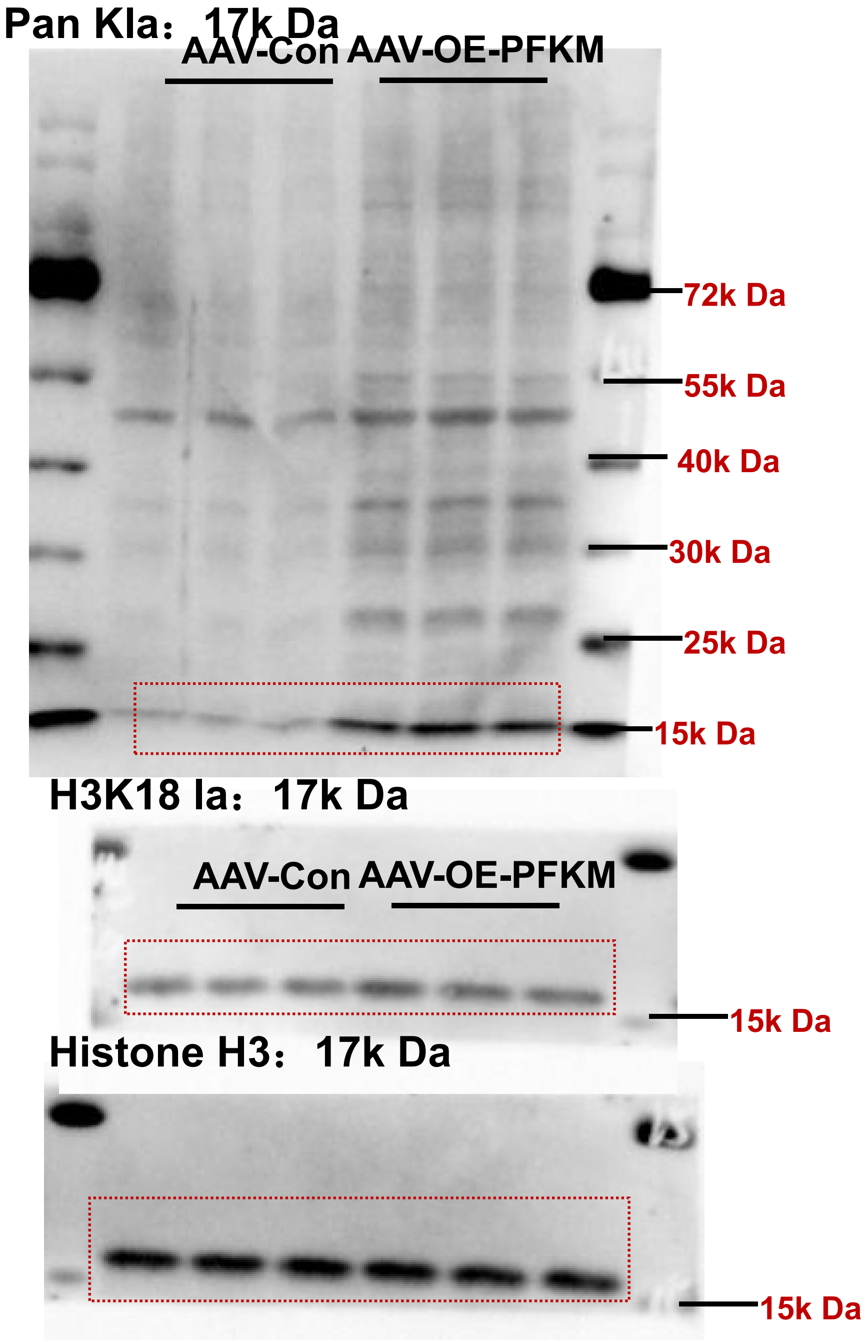

Figure S12C：atrial tissues from CREM mice treated with AAV-PFKM (n = 6)

Pan Kla：17k Da

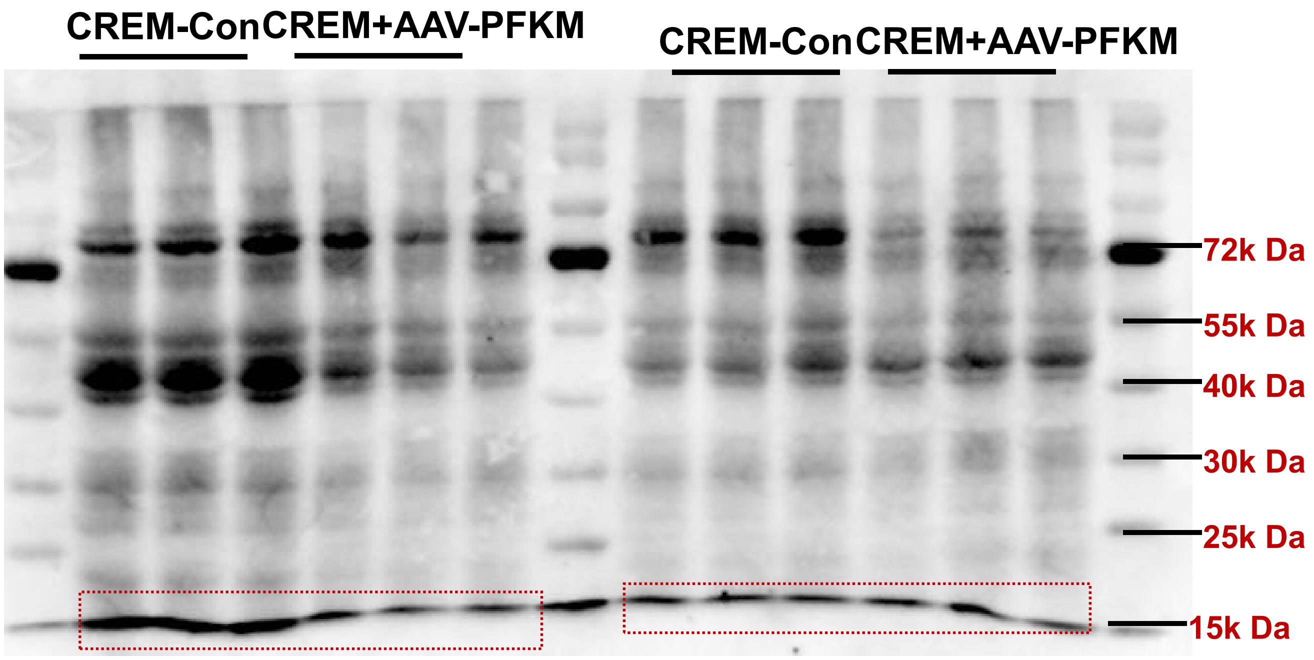

H3 K18 la：17k Da

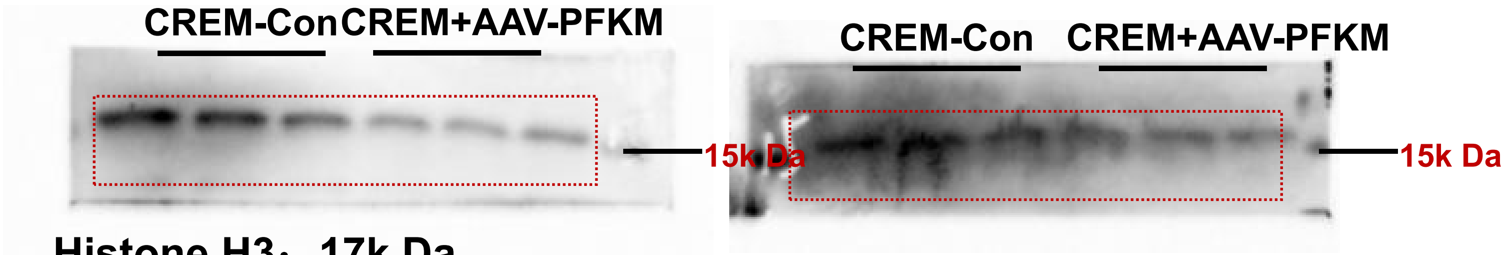

Histone H3：17k Da

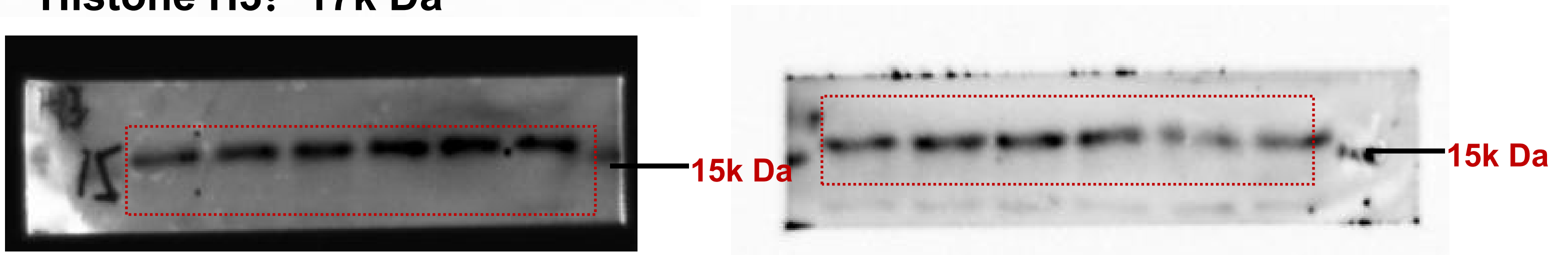

Figure S12C：atrial fibroblasts from CREM mice treated with AAV-PFKM (n = 6)

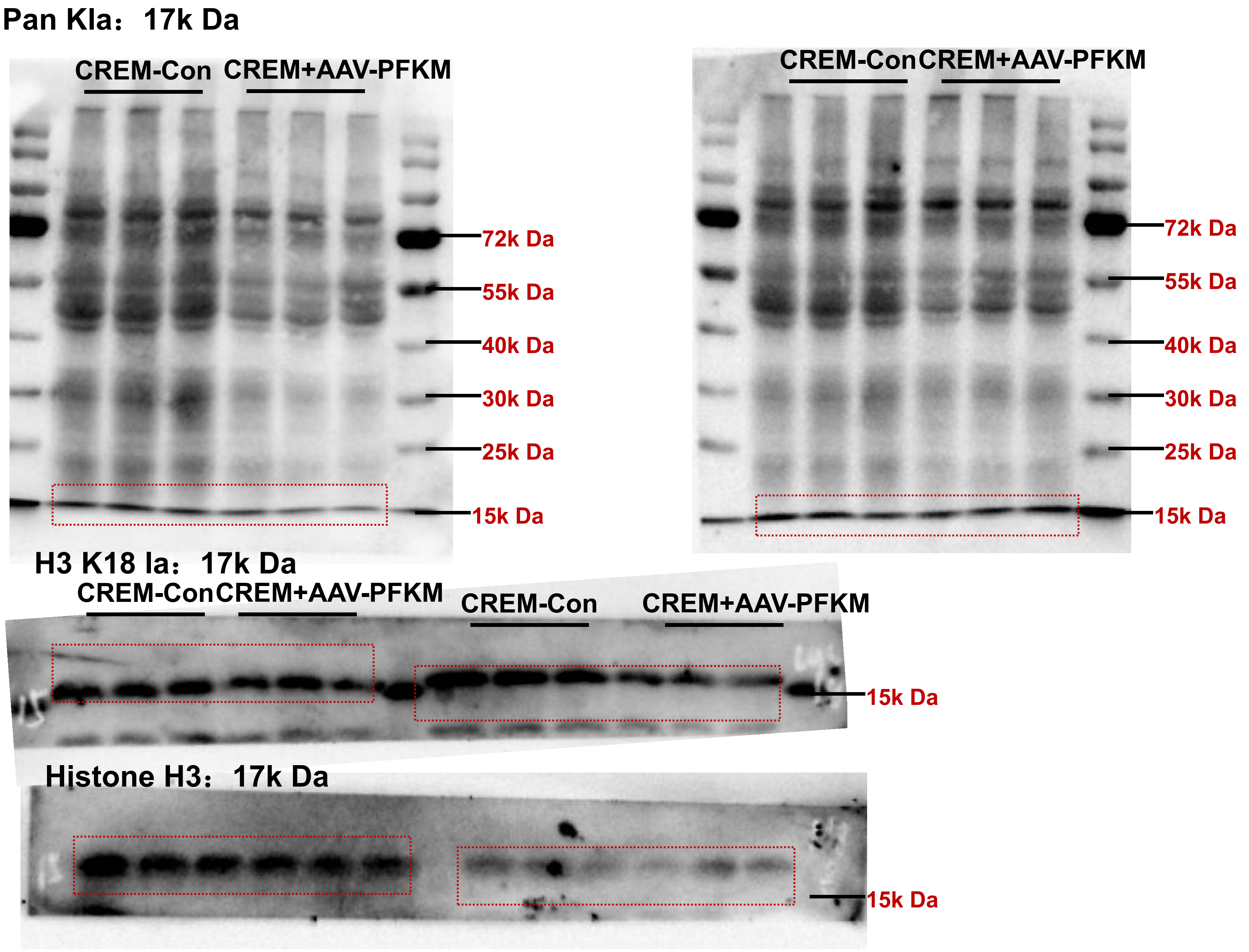

Figure 3E：PFKM immunohistochemical staining of atrial tissue in control group, 3, 5, and 7 months of age

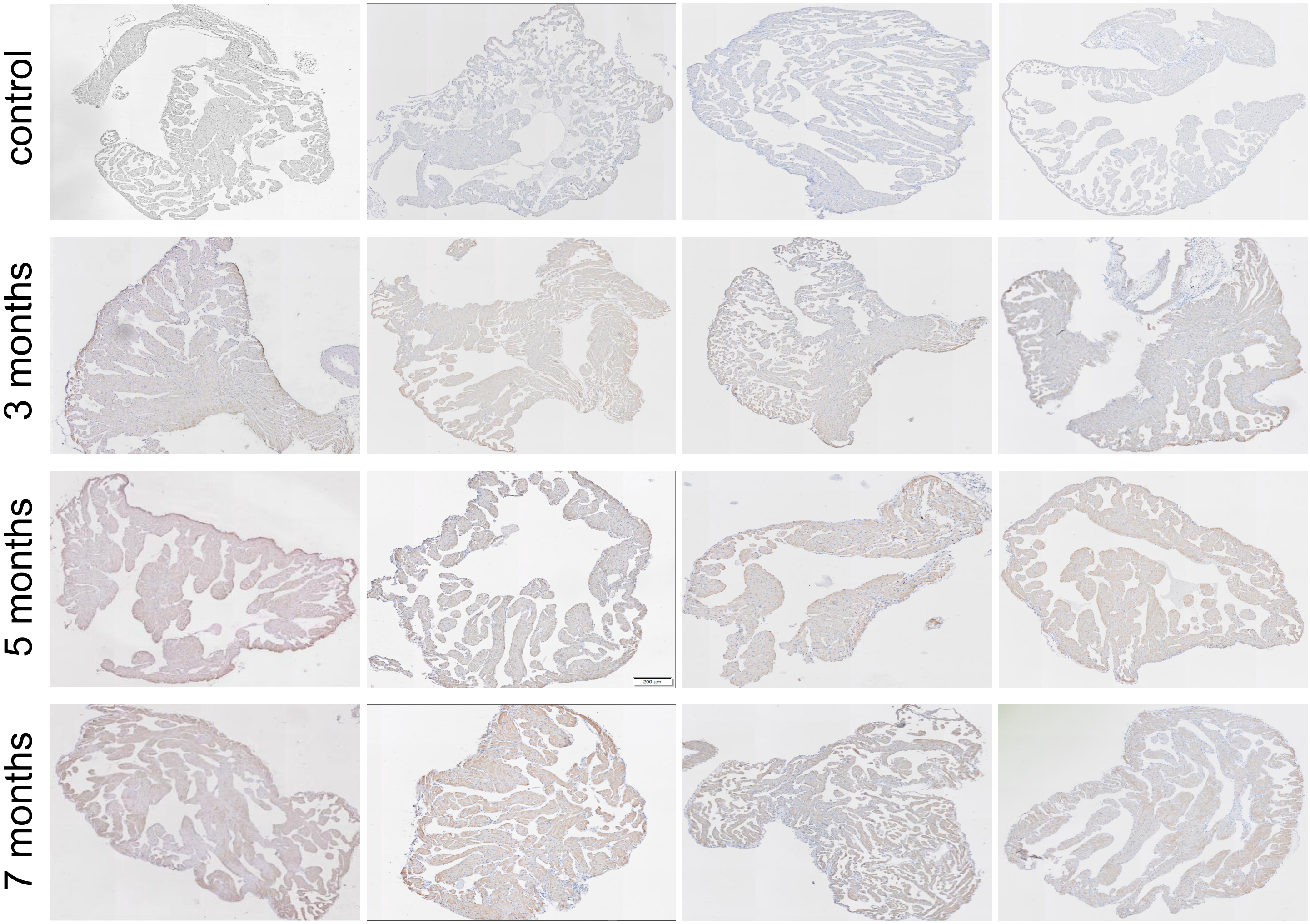

Figure 5D：

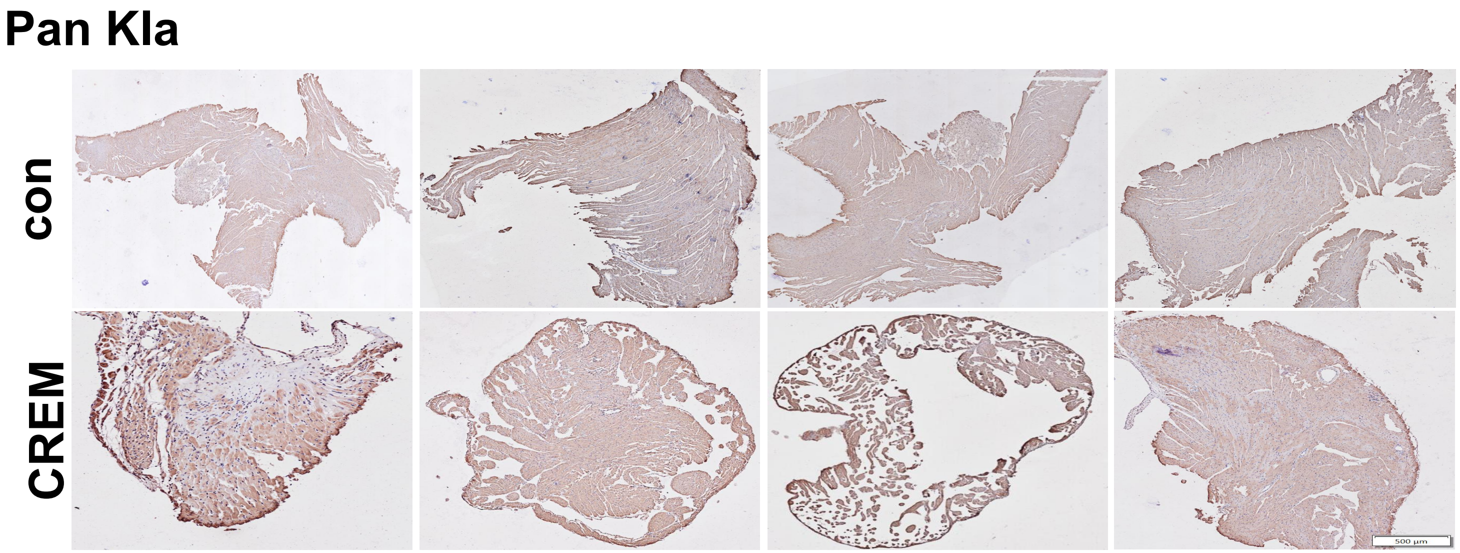

Figure 5E：

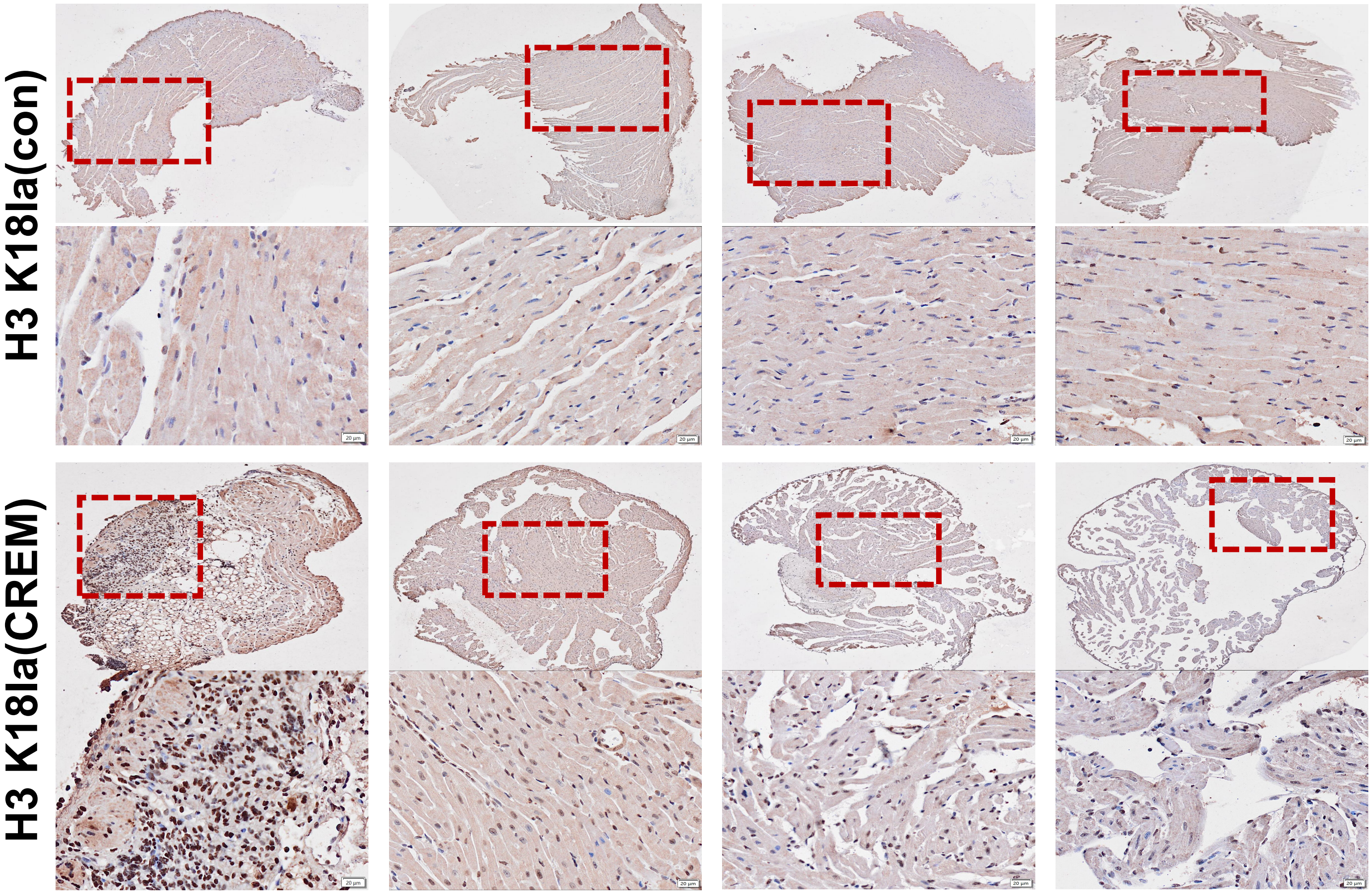

Figure 5F：

Pan Kla(Con)

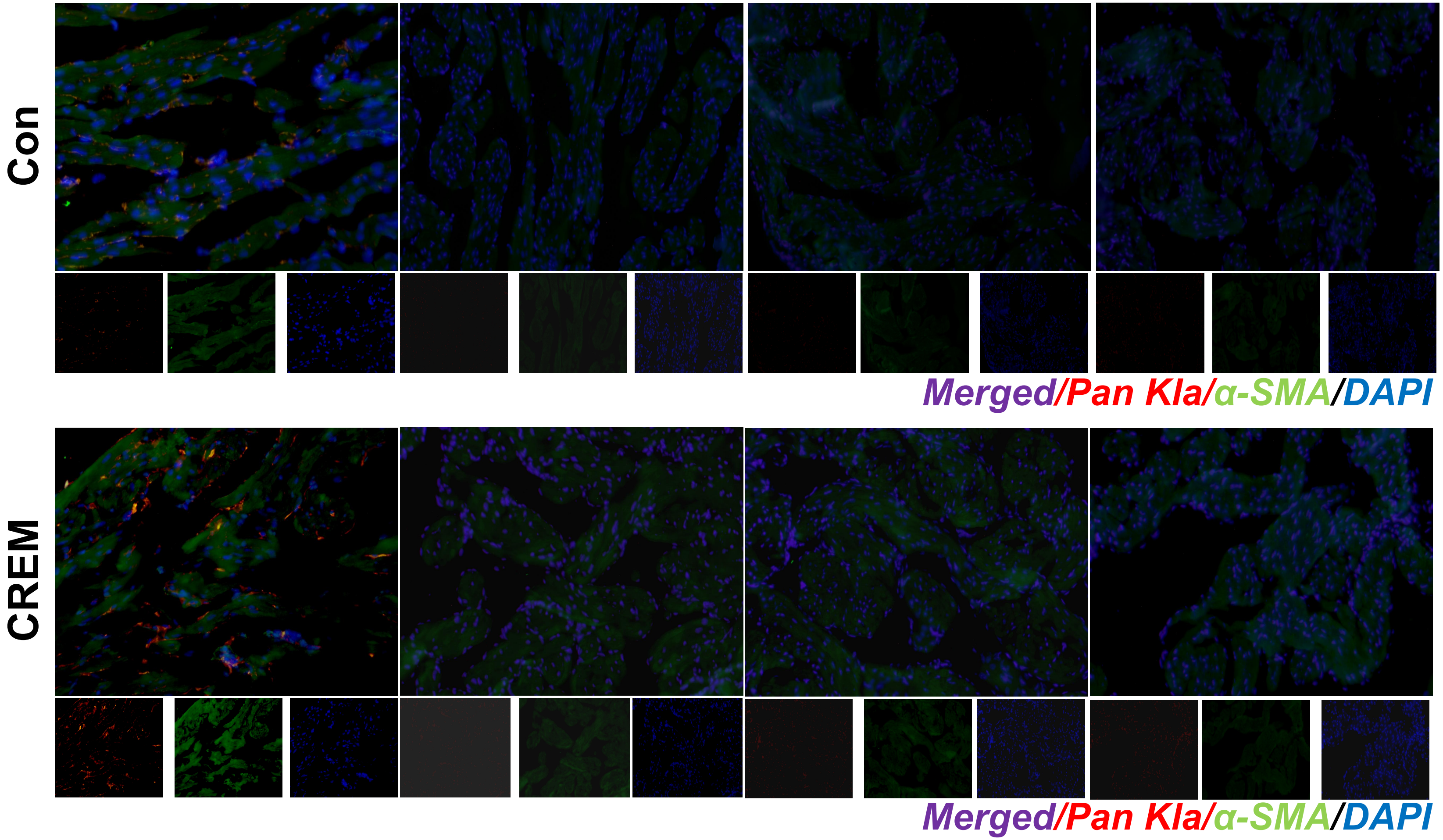

Figure 5G：

H3K18 la(Con Crem)

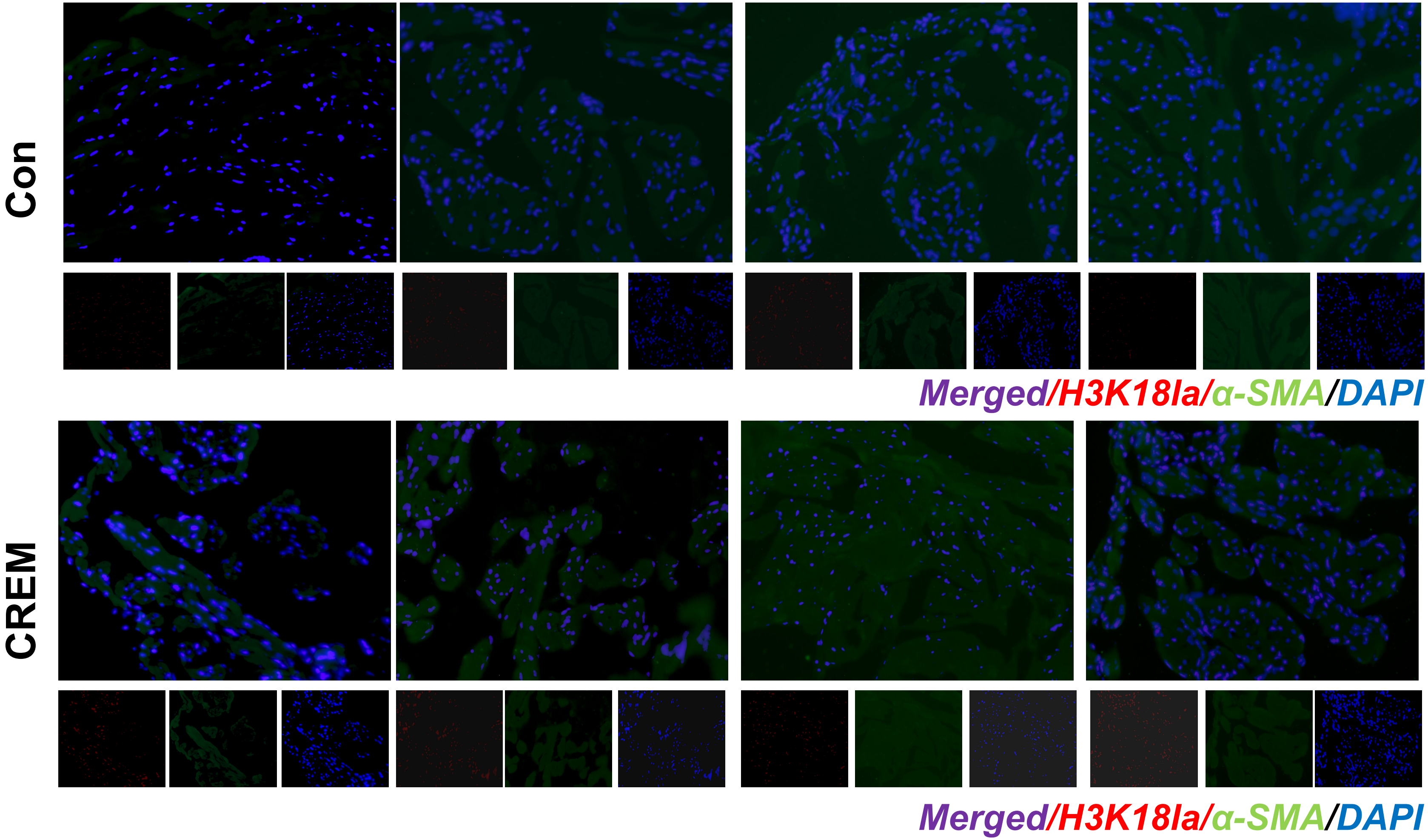

Figure 4G Vimentin Staining in Fibroblasts

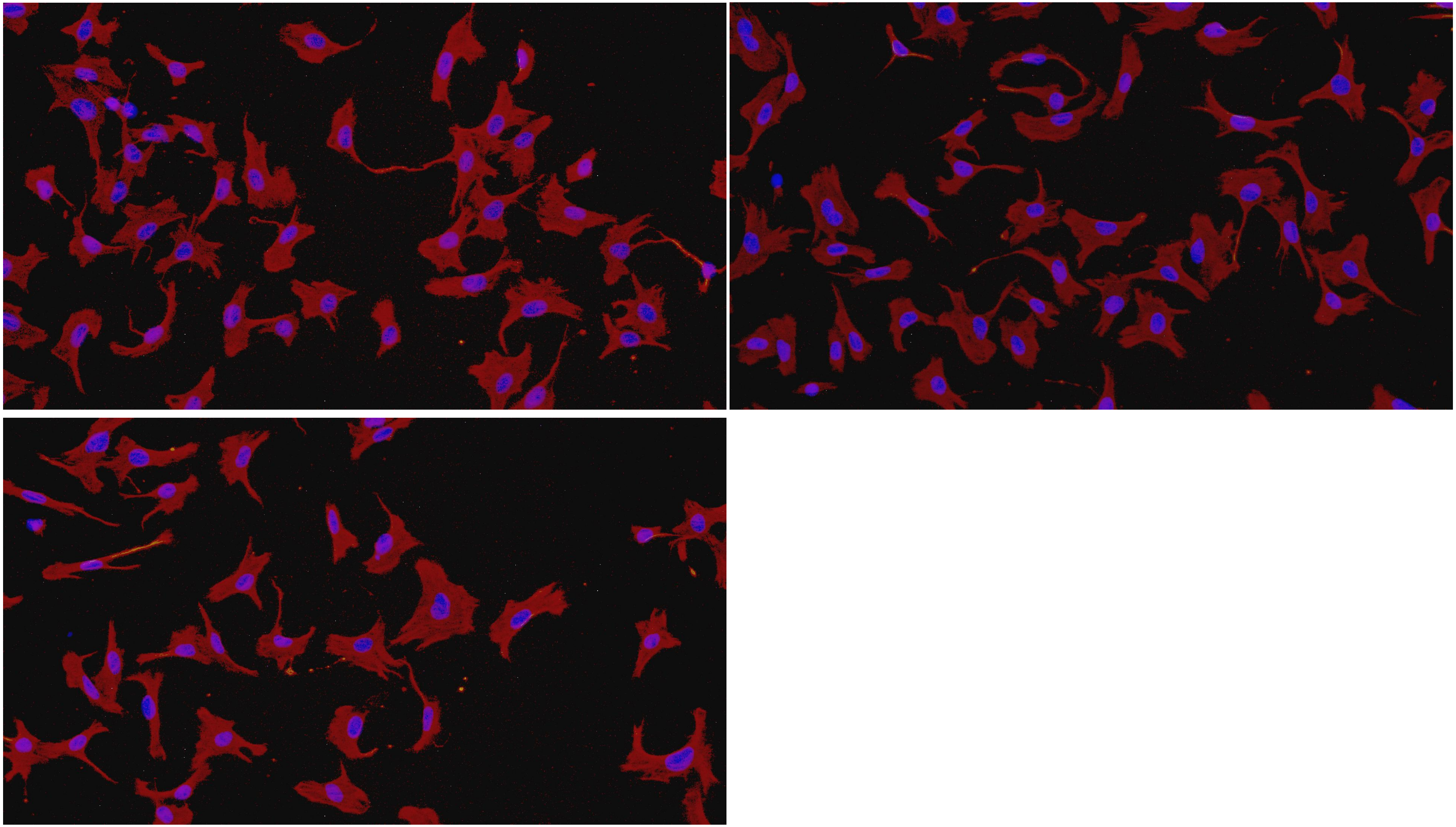

Figure 6J Vimentin Staining in Fibroblasts

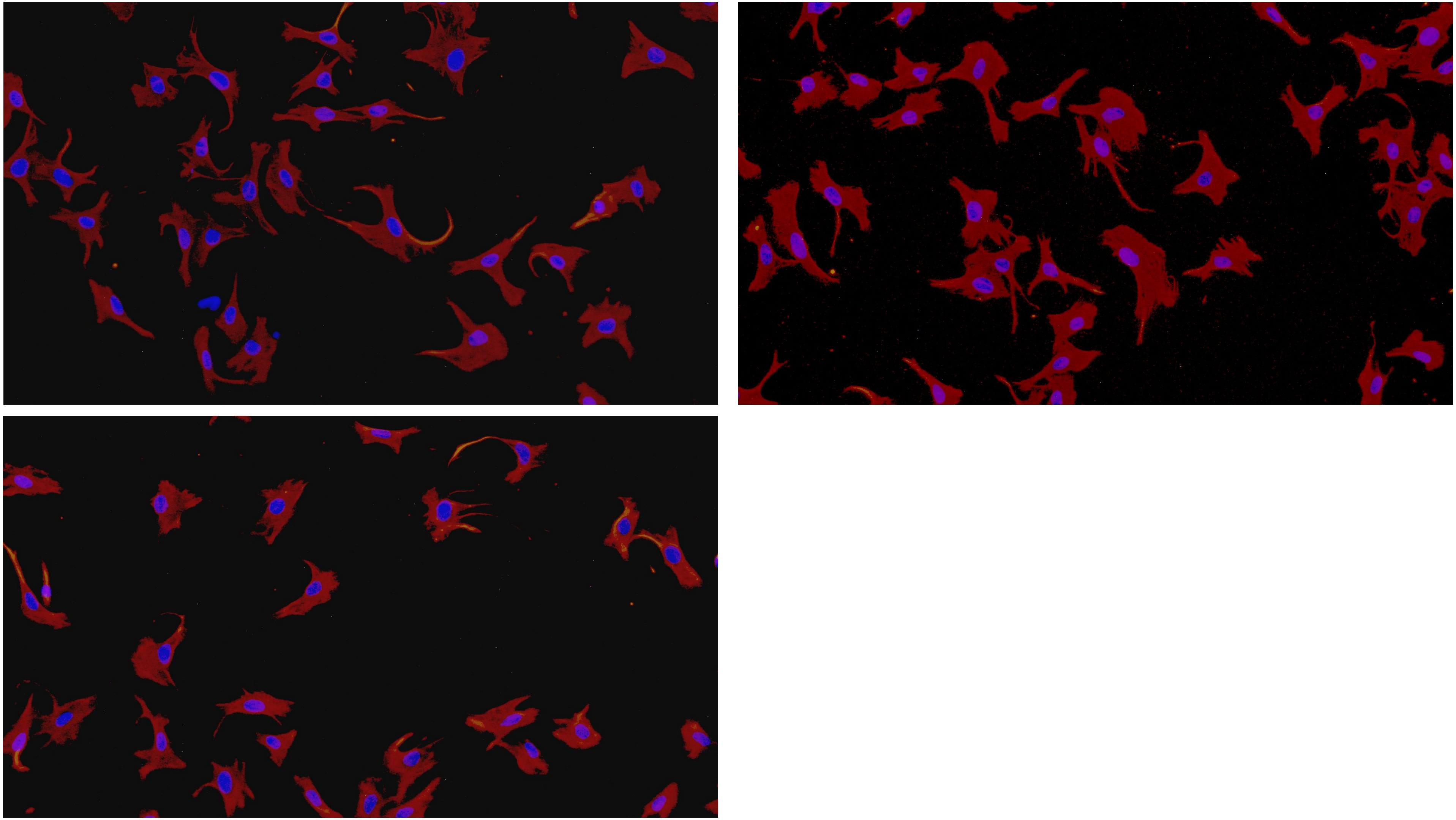

Figure 7G Vimentin Staining in Fibroblasts

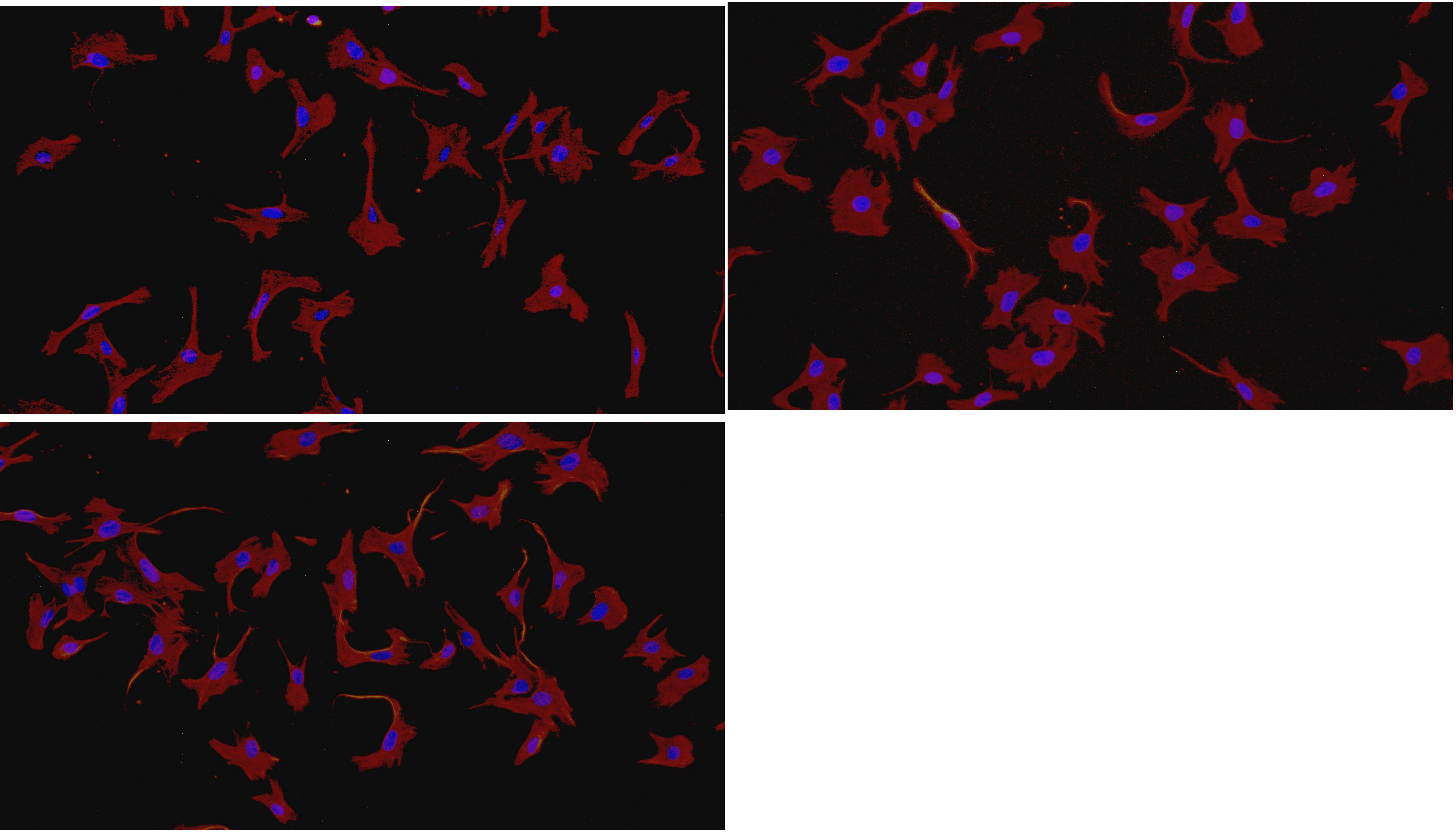

Figure 4G Fluorescence Staining for α-SMA in the control Group

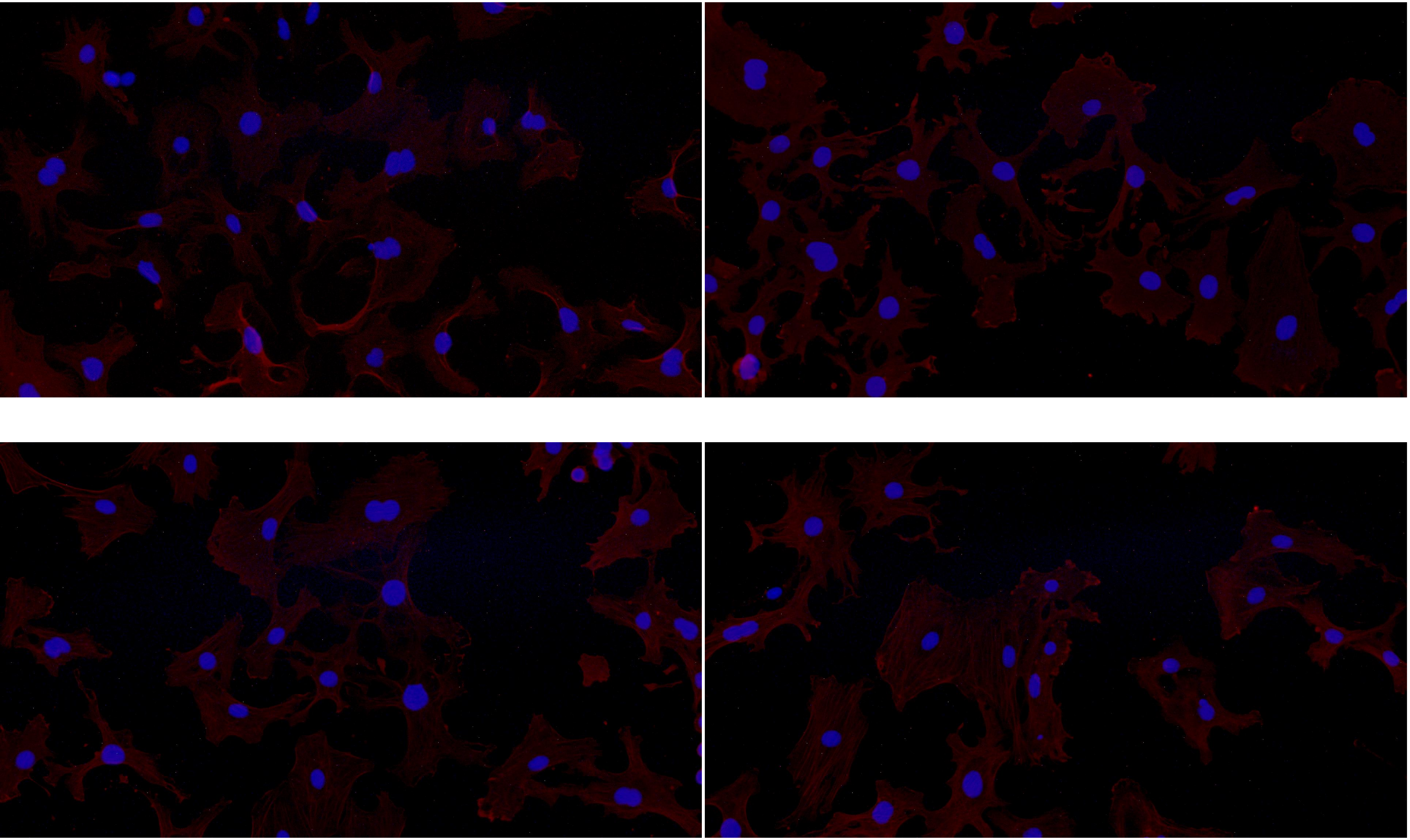

Figure 4G Fluorescence Staining for α-SMA in the Lac Group

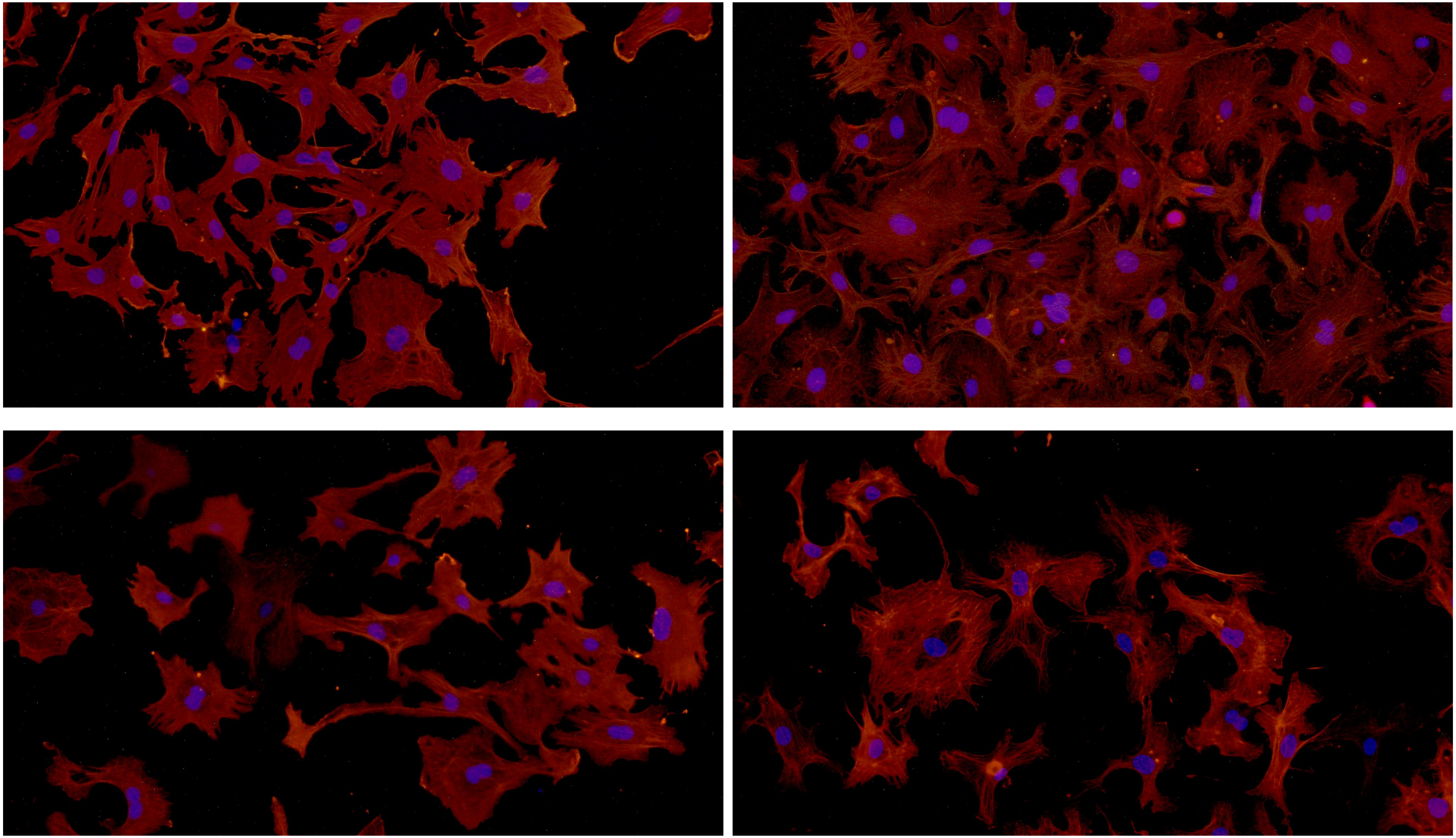

Figure 6J Fluorescence Staining for α-SMA in the Con Group

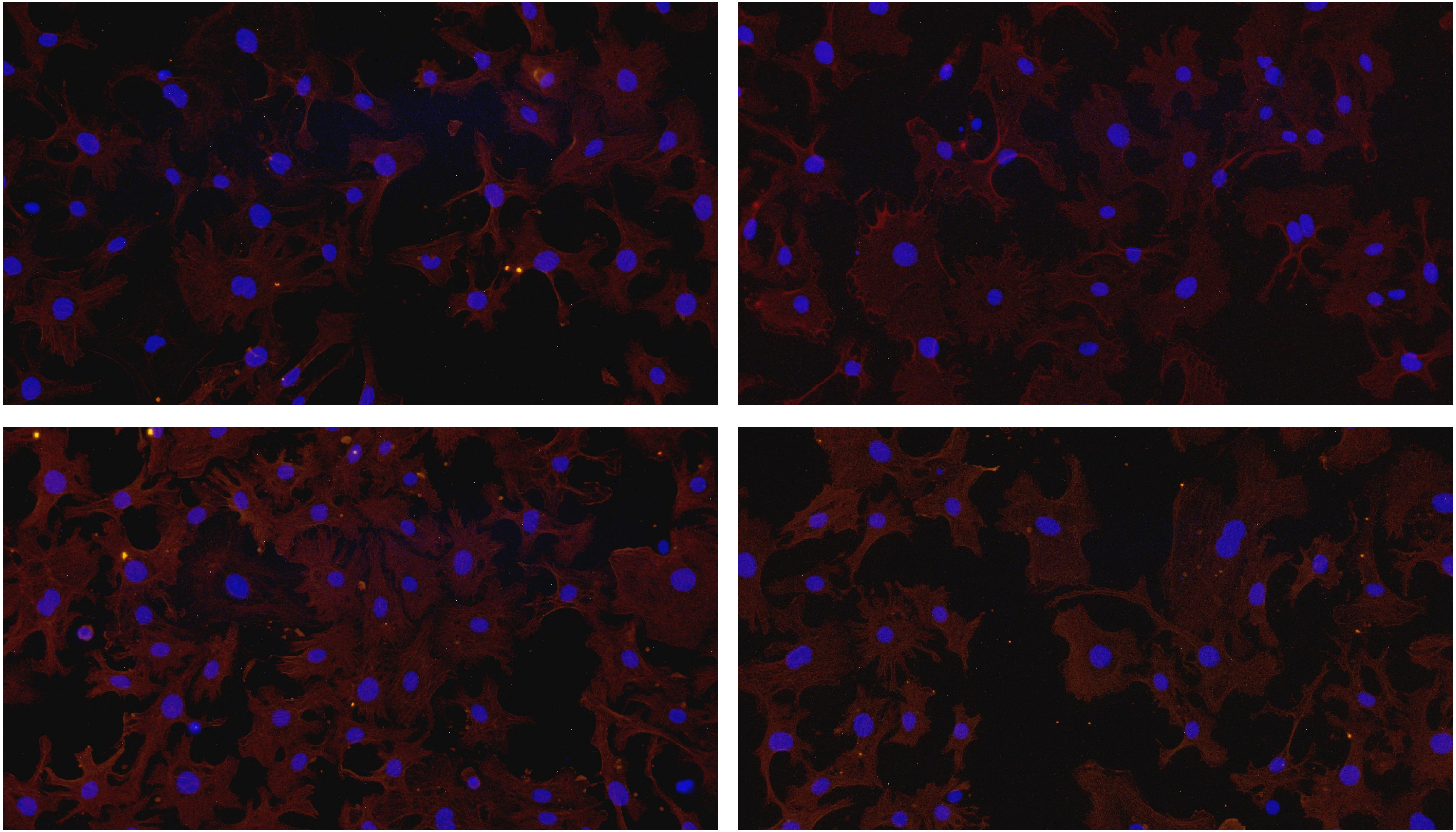

Figure 6J Fluorescence Staining for α-SMA in the Lac Group

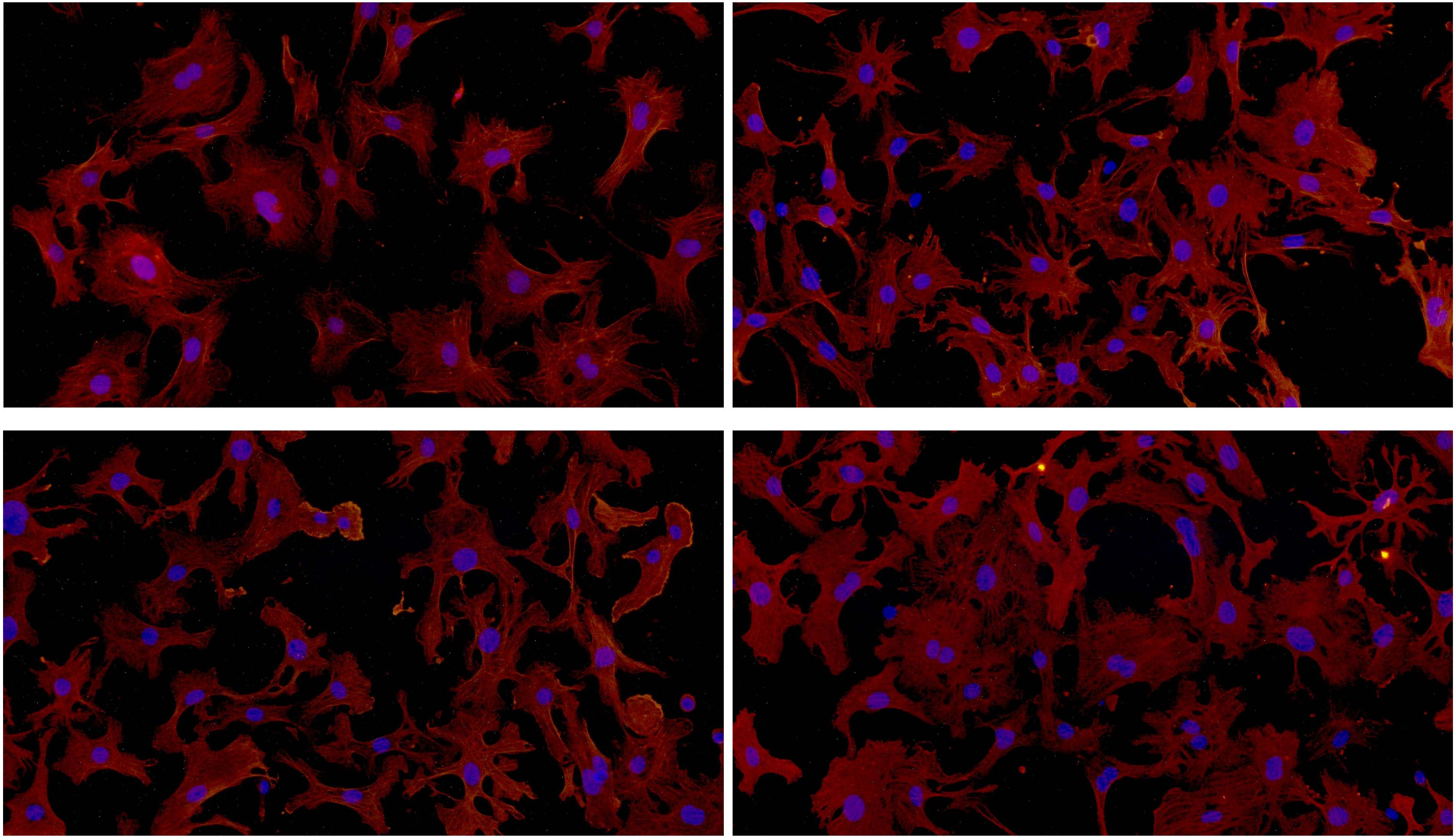

Figure 6J Fluorescence Staining for α-SMA in the Lac+si TGF-β1 Group

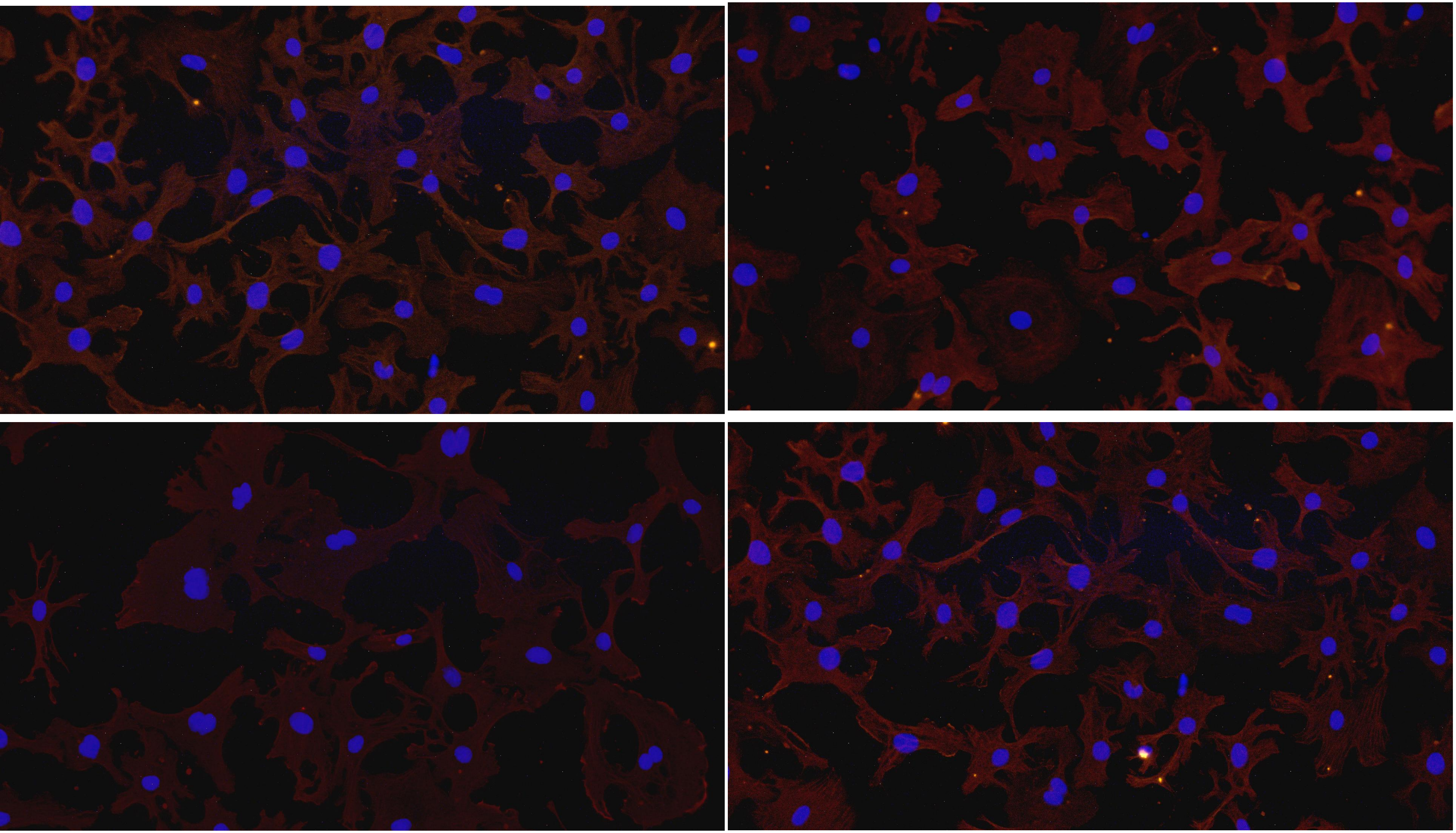

Figure 7G Fluorescence Staining for α-SMA in the Con Group

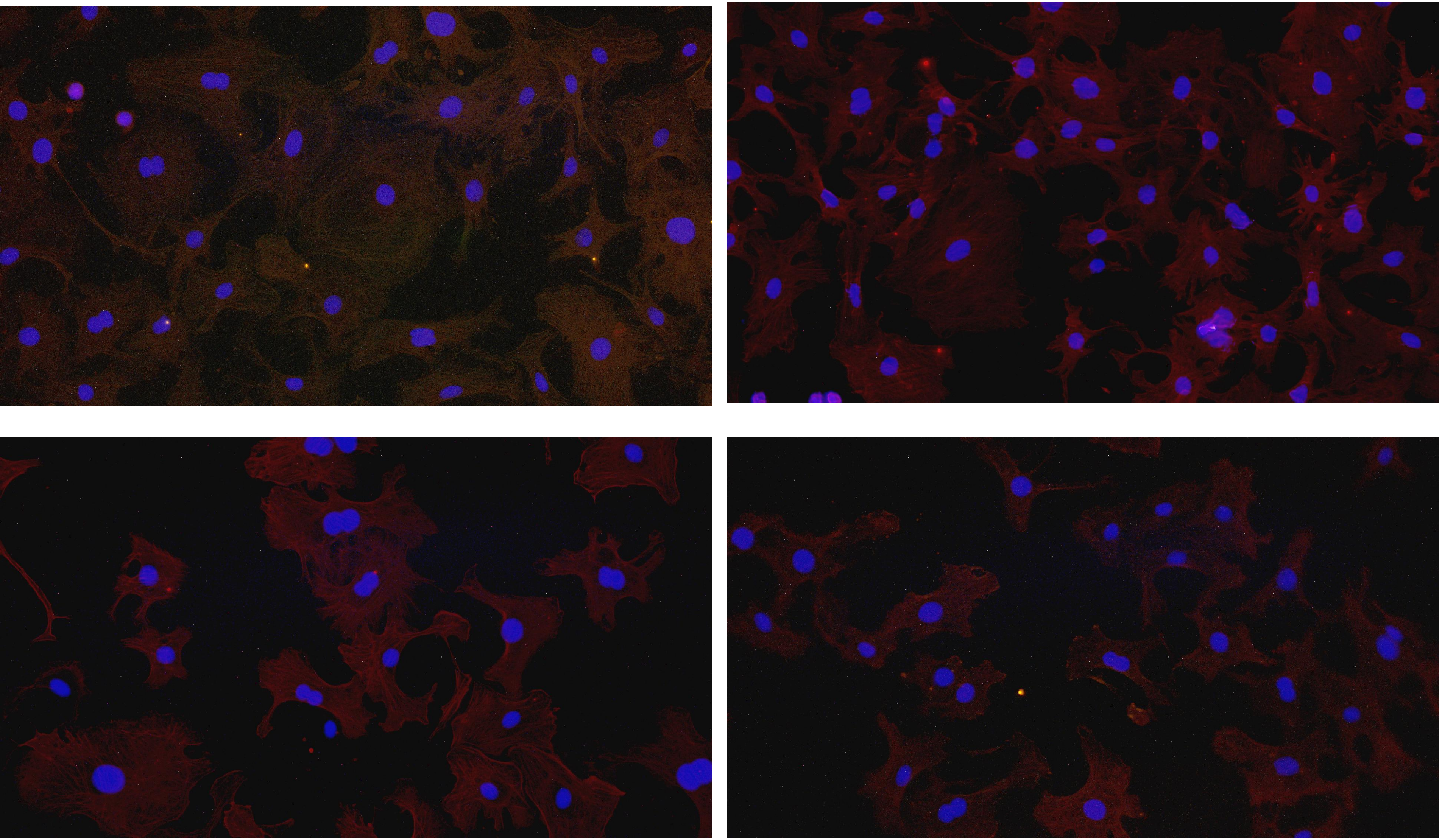

Figure 7G Fluorescence Staining for α-SMA in the Lac Group

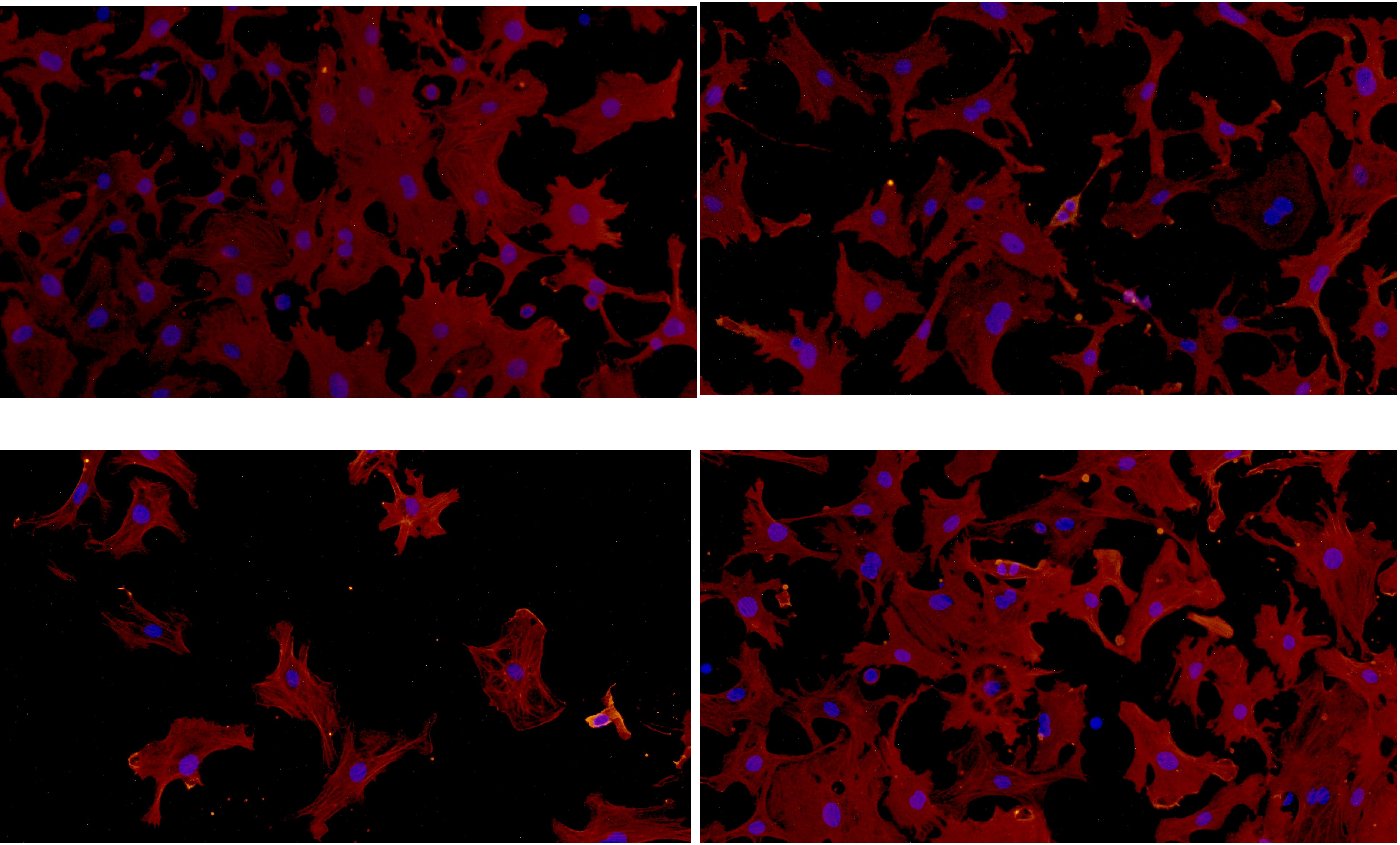

Figure 7G Fluorescence Staining for α-SMA in the siP300 Group

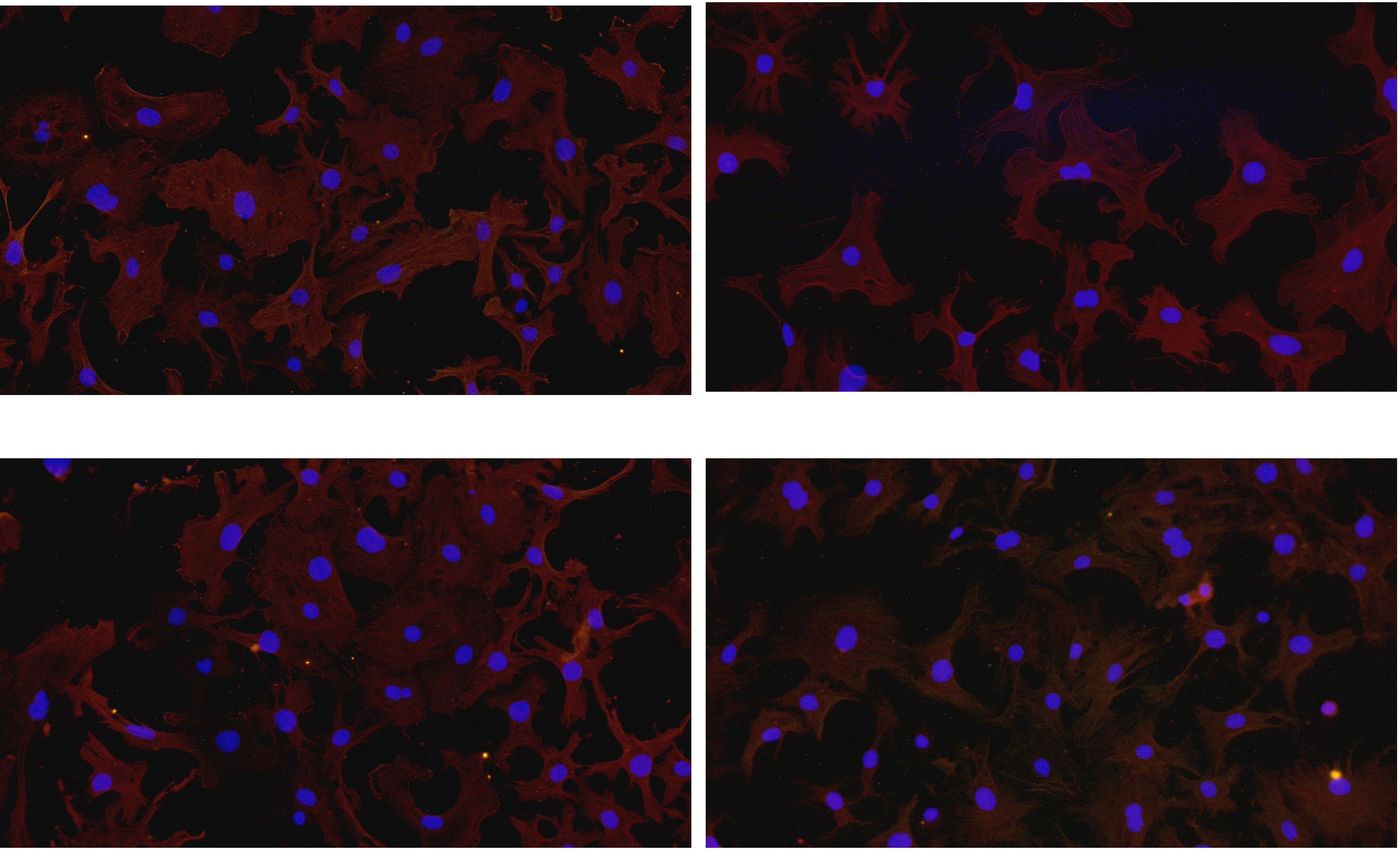

Figure 7G Fluorescence Staining for α-SMA in the si MOF Group

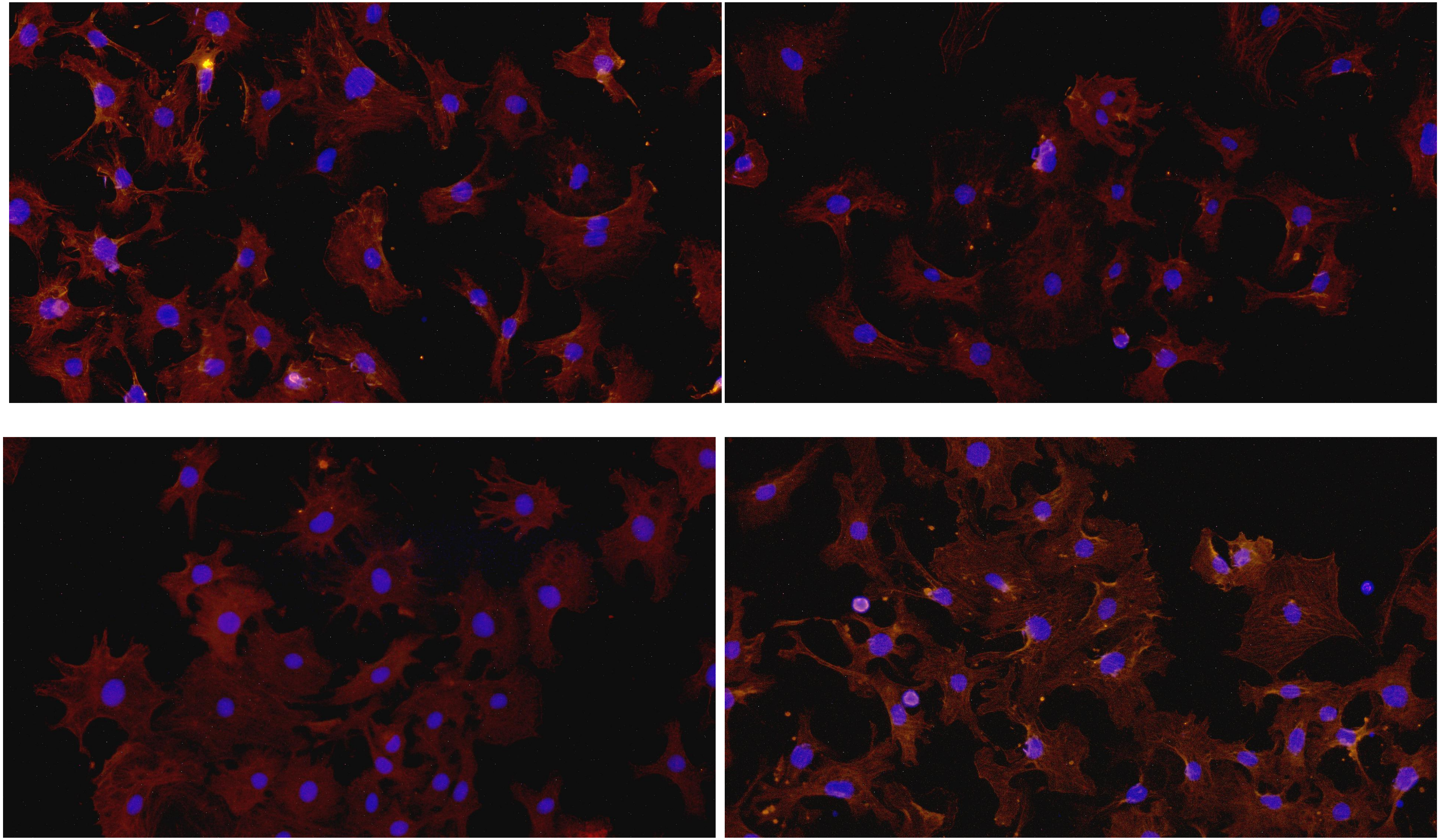

Figure 7G Fluorescence Staining for α-SMA in the si GCN5 Group

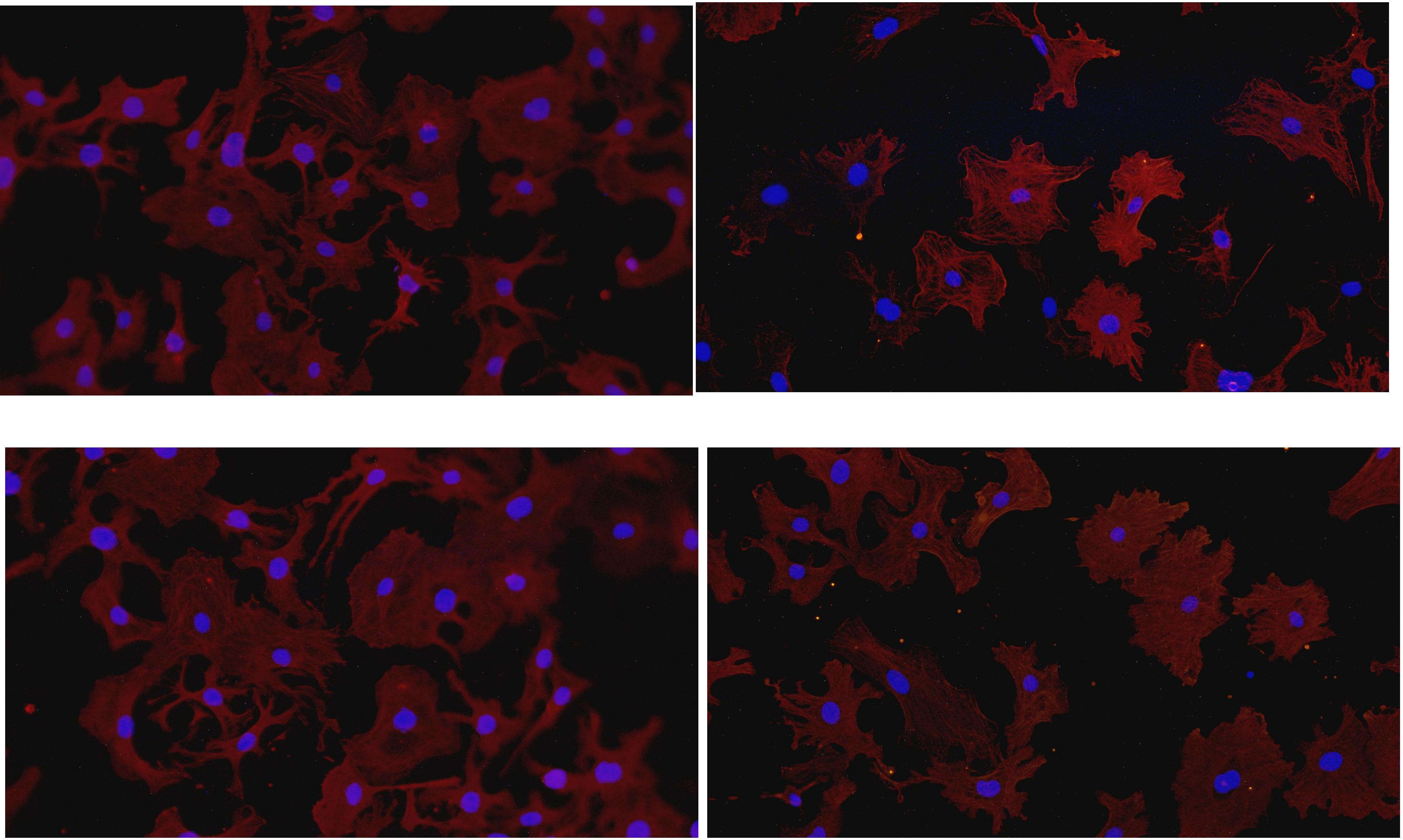

Supplement: Supplementary file 2 — Supporting pdf [file ADVS-12-e00963-s002.zip › supplemental gels and images.pdf]
