# Supplementary material for: PFKM‐Driven Lactate Overproduction Promotes Atrial Fibrillation via Triggering Cardiac Fibroblasts Histone Lactylation
Source: Adv Sci (Weinh). 2025 Jun 26;12(34):e00963. doi: 10.1002/advs.202500963 (PMC12442653; doi:10.1002/advs.202500963)
Supplement: Supplementary file 2 — Supporting pdf [file ADVS-12-e00963-s002.zip › supplemental- animal report and verification.pdf]

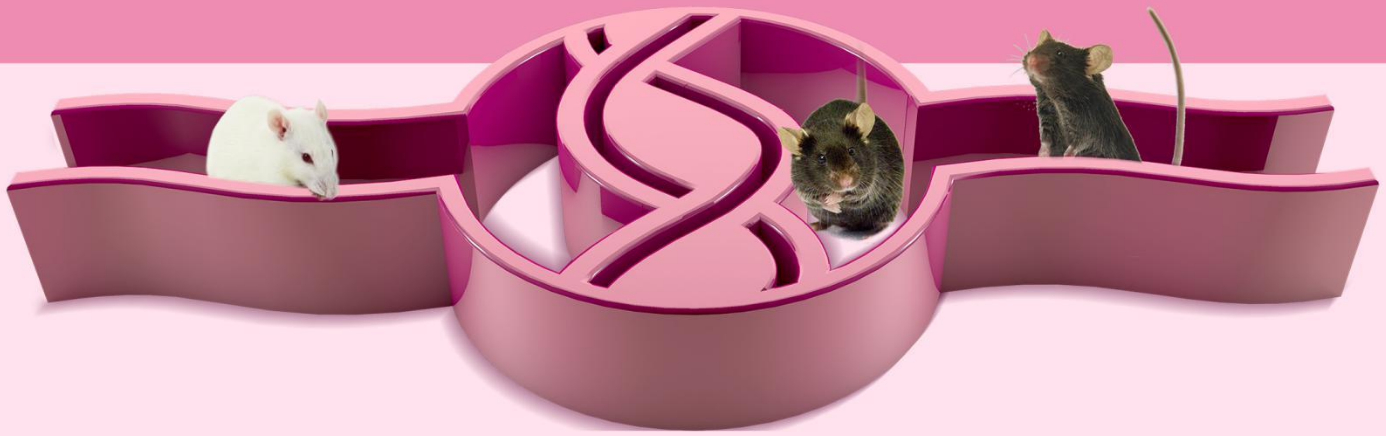

Animal Report

Quote: TGBS220428LB1

- Confidential -

赛业(苏州)生物科技有限公司
Cyagen Biosciences (Suzhou) Inc.
质量保障部
Quality Assurance Department

1. Product 1

**Name of Injected DNA**	CREM-Ib△C-X		
**Mouse Strain**	FVB×FVB		
**Date of Birth**	08-20-2022		
**Description**	Total: 60 pups		
	CREM-Ib△C-X: 1-60		
**PCR-Positive Pups**	♂	2	CREM-Ib△C-X: 17, 38
	♀	2	CREM-Ib△C-X: 12, 50

1.1 Genotyping Strategy

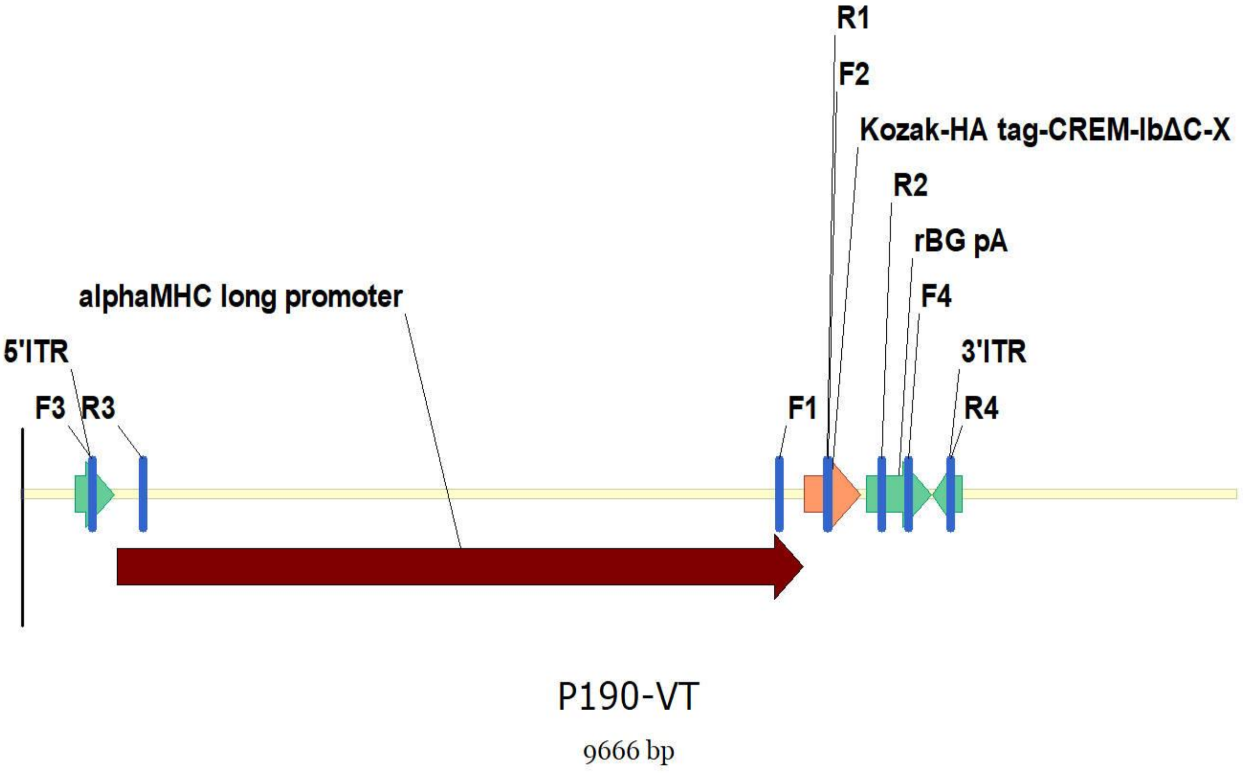

1.2 PCR Conditions

The pups were screened by the following PCR assay. Out of 60 pups screened, 4 were identified positive, which were then confirmed by the same PCR with recut samples.

- **Primers Used:**

Transgene PCR primer F1: TGACAGAGAAGCAGGCACTTTAC
Transgene PCR primer R1: TGGCAAAGCAGCAGTAGGAGCT
Annealing Temp: 60 ℃

Expected PCR Product:

Transgene PCR product size: 399 bp

- **Primers Used:**

Transgene PCR primer F2: CTTTGCCACAGGGAGTGGTGAT
Transgene PCR primer R2: CTTTATTAGCCAGAAGTCAGATGC
Annealing Temp: 60 ℃

Expected PCR Product:

Transgene PCR product size: 446 bp

- **Primers Used:**

Transgene PCR primer F3: GCTTGTCAATGCGGTAAGTGTCA
Transgene PCR primer R3: CACCTAAAGGGCTGTTGCAAA
Annealing Temp: 60 ℃

Expected PCR Product:

Transgene PCR product size: 430 bp

- **Primers Used:**

Transgene PCR primer F4: CTGCTGTCCATTCCTTATTCCATAG
Transgene PCR primer R4: TTATCGGTCTGTATATCGAGG
Annealing Temp: 60 ℃

Expected PCR Product:

Transgene PCR product size: 356 bp

1.3 PCR Result

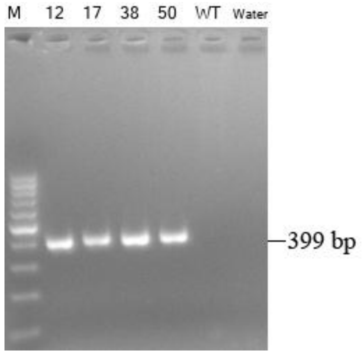

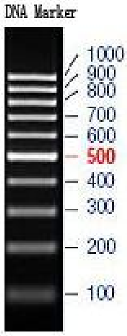

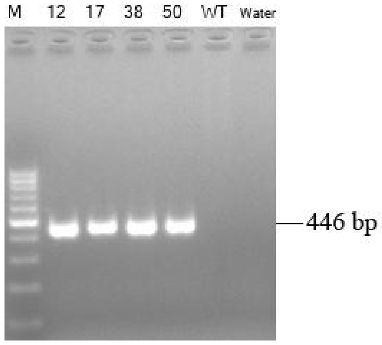

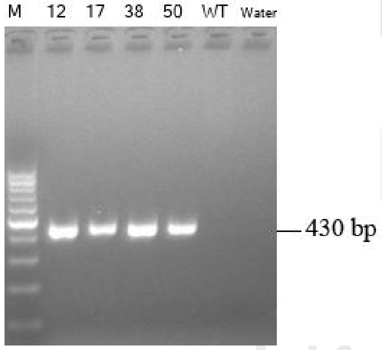

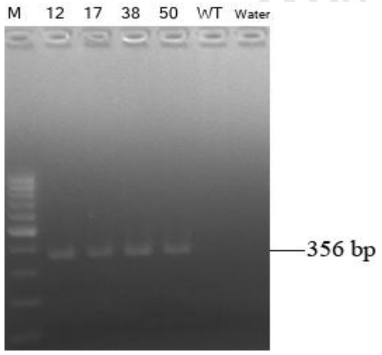

cyagen We help you discover life

Note:

1) PCR was carried out in 25 μL volume for 35 cycles under standard conditions, with all primers listed above added to each reaction.

2) Taq DNA polymerase used was P222.

3) Two controls used in PCR genotyping are:

- Water control: No DNA template added.
- Wildtype control: Mouse genomic DNA.

4) For PiggyBac transgenic mice, all positive pups were confirmed by PCR to not contain any integration of the helper plasmid.

   Primers used in the PCR to test for helper plasmid integration:

   PiggyBac Helper plasmid-F: CTGGACGAGCAGAACGTGATCG

   PiggyBac Helper plasmid-R: CGAAGAAGGCGTAGATCTCGTCCTC

   Annealing Temp: 60 ℃

   Expected PCR product size: 352 bp

Client should report any problems with the delivered mice and other materials to Cyagen within one week of receiving the shipment.

cyagen We help you discover life

2. PCR Conditions Attachment

2.1 DNA Extraction

- Method One:

We recommend that using TaKaRa MiniBEST Universal Genomic DNA Extraction kit (Ver.5.0_Code No. 9765) to gain high purity of genomic DNA.

a. Add 180 μL of Buffer GL, 20 μL of Proteinase K and 10 μL of RNase A per tail piece (2-5 mm) in a microcentrifuge tube. Be careful not to cut too much tail.

b. Incubate the tube at 56 ℃ overnight.

c. Spin in microcentrifuge at 12,000 rpm for 2 minutes to remove impurities.

d. Add 200 μL Buffer GB and 200 μL absolute ethyl alcohol with sufficient mixing.

e. Place the spin Column in a collection tube. Apply the sample to the spin and centrifuge at 12,000 rpm for 2 min. Discard flow-through.

f. Add 500 μL Buffer WA to the spin column and centrifuge at 12,000 rpm for 1 min. Discard flow-through.

g. Add 700 μL Buffer WB to the spin column and centrifuge at 12,000 rpm for 1 min. Discard flow-through. (Note: Make sure the Buffer WB has been premixed with 100% ethanol. When adding Buffer WB, add to the tube wall to wash off the residual salt.)

h. Repeat step g.

i. Place the spin Column in a collection tube and centrifuge at 12,000 rpm for 2 min.

j. Place the spin Column in a new 1.5ml tube. Add 50~200 μL sterilized water or elution buffer to the center of the column membrane and let the column stand 5min. (Note: Heating sterilized water or elution buffer up to 65℃ can increase the yield of elution.)

k. To elute DNA, centrifuge the column at 12,000 rpm for 2 min. To increase the yield of DNA, add the flow-through and/or 50~200 μL sterilized water or elution buffer to the center of the spin column membrane and let the column stand 5 min. Centrifuge at 12,000 rpm for 2 min.

l. Quantify to genomic DNA. Eluted genomic DNA can be quantified by electrophoresis or electrophoresis.

- Method Two:

A low-cost and sample method to gain rough genomic DNA.

a. Add 100 μL of tail digestion buffer per tail piece (2-5 mm) in a microcentrifuge tube. Be careful not to cut too much tail.

b. Incubate the tube at 56 ℃ overnight.

c. Incubate the tube at 98 ℃ for 13 minutes to denature the Proteinase K.

d. Spin in microcentrifuge at top speed for 15 minutes. Use an aliquot of supernatant straight from the tube (1.5 μL in a 25 μL reaction) for PCR.

Final concentration of tail digestion buffer:

- 50 mM KCl
- 10 mM Tris-HCl (pH 9.0)

- 0.1 % Triton X-100
- 0.4 mg/mL Proteinase K

2.2 PCR Mixture (primer concentration: 10μM):

Component	x1
ddH_2O	9.0 μl
Product primer F	1.0 μl
Product primer R	1.0 μl
Premix Taq	12.5 μl
DNA	1.5 μl
Total	25 μl

2.3 PCR Reaction Conditions:

Step	Temp.	Time	Cycles
Initial denaturation	94 °C	3 min	
Denaturation	94 °C	30 s	35 x
Annealing	60 °C	35 s	
Extension	72 °C	35 s	
Additional extension	72 °C	5 min	

2.4 Relevant Reagents:

**Trizma Hydrochloride Solution**	Sigma, Cat. No. T2663
**Proteinase K**	Merck, Cat. No. MK539480
**Triton X-100**	Sigma, T8787-50 mL
**2 × Taq Master Mix (Dye Plus)**	Vazyme, P222
**Agarose**	BIOWEST AGAROSE, REGULAR
**DNA Marker**	Thermo Scientific GeneRuler 100 bp DNA Ladder #SM0242
**0.5×TBE**	Tris Bio Basic Inc, TBO194-500g
	EDTA Shanghai Sangon, 0105-500g
	Boric Acid, Shanghai Sangon, 0588-500g

转基因动物项目协议(PiggyBac)

协议编号：TGBS220428LB1

甲　　方：哈尔滨医科大学附属第一医院	乙　方：赛业（苏州）生物科技有限公司
课 题 组：公永太	联系人：刘博
负 责 人：金学鑫	电　话：400-680-8038
电　　话：18845076706	传　真：0512-82782879
邮　　箱：1554959823@qq.com	邮　箱：info@cyagen.com
发票抬头：	户　名：赛业（苏州）生物科技有限公司
纳税人识别号：	开户行：交通银行股份有限公司太仓分行
地　　址：黑龙江省哈尔滨市南岗区哈尔滨医科大学附属第一医院	账　号：3926 8860 1018 1701 58636
邮　　编：150081	开户行联行号：301305100011
	地　址：江苏省苏州市太仓市东仓南路 19 号
	邮　编：215400

内容概述：

甲乙双方经协商一致，同意开展项目：转基因动物项目(PiggyBac)。

本协议内容共八项，细则详见第 2 页到第 5 页：

一、实验信息表

二、项目内容

三、项目费用

四、付款方式

五、项目启动与汇报

六、成果交付

七、保密责任

八、违约责任

甲　　方：	乙　　方：赛业（苏州）生物科技有限公司
负责人签字（签章）：	代表签字（签章）：
签约时间：　　　　年　　月　　日	签约时间：　　　　年　　月　　日

项目名称：转基因动物技术服务协议(PiggyBac)

协议编号：TGBS220428LB1

一. 实验信息

目的基因信息	名称	CREM-IbΔC-X	Gene ID	NM_183011.2
**动物品系**	**FVB**		**动物级别**	SPF 级
**备注**	方案见附件			

二. 项目内容

**1、载体构建和纯化**	■乙方按照甲方的要求构建用于制作转基因鼠所需的载体，并将其纯化。
**2、转基因鼠的制备**	■乙方将（1）纯化的载体和 PiggyBac Helper plasmid 注射到受精卵后移植到代孕母鼠输卵管中。最终提供给甲方经 PCR 鉴定的至少 3 只阳性首建鼠。
**3、RT-QPCR**	3 只阳性首建鼠取鼠尾进行 RT-QPCR 检测。

三. 项目费用

序号	项目	周期（周）	标准价格（元）	优惠价格（元）
GPPS	启动子调取	2	500	500
	基因合成			
TGVBS	载体构建	7	8500	8500
TBS	载体纯化	1	1000	1000
	DNA 显微注射（FVB）	8-12	40000	40000
	RT-QPCR（3 只）	1-2	1320	1320
	5 只 FVB 野生型小鼠（C001193）	——	免费	免费
合计		19-24	51320	51320
协议金额	**￥51320** 元（大写人民币：伍万壹仟叁佰贰拾圆整）			
备注 1	**本协议款项中 49500 元来源于合同 KOCRS210906LB2 和 KOCRS210906LB3-B，**			

	**甲方需在本协议支付 1820 元。**
备注 2	**本项目发货后如果甲方需要将小鼠（FVB）进行精子冻存，则乙方赠送甲方一个 line 小鼠精子冻存，接鼠费用 1800 元甲方另付，冻存超出一个 line 按照 6000/line 标准收费。**

四．　项目启动与汇报

1、本协议约定项目于本协议签署后的 5 个工作日内启动。

2、乙方指定专人负责项目汇报，根据项目实际进度提供汇报。

五．　成果交付

1、甲方付清本协议全款后，将获得以下项目成果。

项目阶段	项目成果	
载体构建	载体设计方案及实验报告（1 套）	载体（1 管）
显微注射	小鼠鉴定实验报告（1 套）	PCR 鉴定为阳性的首建鼠（3 只），RT-QPCR 报告
备注	甲方赠送乙方 5 只 FVB 野生型小鼠（C001193）和项目鼠一同发货	

2、双方对本项目相关产权约定如下：

①本协议签订前双方已有的知识产权归双方各自所有。

②甲方按照本协议约定付清全款后，享有本项目约定交付项目成果中载体及阳性鼠的所有权，甲方亦可援引载体设计方案及实验报告、鼠鉴定实验报告中的有关内容进行文章创作、发表。甲方基于本项目交付项目成果的后续改进而形成的相关知识产权归甲方所有。若乙方基于本项目所获得的载体、阳性鼠多于本协议约定的交付量，未经甲方书面同意，乙方不得将该等载体、阳性鼠以任何方式向第三方转让、提供或泄露。

③除上述约定外，在本项目开展过程中，产生的其他知识产权包括但不限于制备、鉴定鼠的工艺、流程、方法、技术、方案、报告等智力成果涉及的专利、著作权等，归乙方所有。

3、乙方在项目正式启动后的 22 个周内，为甲方提供以上项目成果，并确保安全送达甲方所在地(甲方指定的接收项目成果的地点为【黑龙江省哈尔滨市南岗区哈尔滨医科大学附属第一医院】；若部分项目成果为电子文件，则甲方指定接收电子文件的邮箱为【1554959823@qq.com】)。

4、甲方应在接到乙方通知后 1 个月内完成小鼠接收。超过 1 个月部分乙方将按照 500 元/月计费，不足 1 个月按一个月计费，收取小鼠饲养管理费用；乙方发出接收通知 2 个月后，如甲方仍未能接收小鼠，乙方有权对本项目小鼠进行冷冻保种，且不再保存活体鼠，甲方仍需支付本协议所需的费用；后续甲方若需要小鼠，甲方需承担复苏费用 5000 元（包含复苏所需 WT 雌鼠动物费、精子复苏操作、体外受精（IVF）、出生子代的鉴定和饲养费用，不包含子代小鼠运输费用），乙方可按照本协议承诺交付结果提供小鼠，届时双方另行签署补充协议收取费用。

七．保密责任

任何一方违反以下保密责任的，须赔偿对方由此产生的全部损失：

1、乙方对甲方提供的技术信息和技术资料负有保密的义务，未经甲方允许不得向任何第三方提供。对于已公开的技术信息和技术资料，乙方不承担保密责任。

2、甲方对乙方所提供的项目的技术信息、技术资料及商业秘密（包括但不限于成交价格、发票、财务收据等），负有保密义务，未经乙方允许不得向任何第三方提供。对于已公开的技术信息和技术资料，甲方不承担保密责任。

八．违约责任

1、 如若需要进行第二次注射，则协议周期相应延长 6-8 周，由此造成的项目延期，不属于乙方违约范畴。

2、 如果基因具有胚胎致死性导致实验失败，则甲方应能够理解和接受，并按照协议金额支付显微注射的全部费用。如果基因不具有胚胎致死性或不明原因导致实验失败，乙方在进行第二次注射后仍然无法获得甲乙双方约定的实验成果时，在甲方要求或双方协商一致后，每延期 1 周（不足 1 周按 1 周算）乙方按合同总金额 0.3%的标准补偿金赔付甲方，至乙方向甲方发出项目结题通知之日止。如果甲方同意乙方继续完成项目，则甲乙双方共同另行约定周期。

3、 若乙方未能获得约定的 PCR 鉴定为阳性的首建鼠，乙方可要求解除本协议，并承诺可全额退还甲方已支付的款项，此外，甲方不得要求乙方承担任何违约责任；若乙方已向甲方支付延期补偿金，可在退还甲方的款项中予以扣除。

4、 乙方保证实验操作的准确性，但由于生物学的未知性，乙方不能确认最终提供基因打靶小鼠具备客户预期的生理功能，且暂不提供表型分析服务。

5、 若由于甲方单方面要求终止项目，则甲方应支付乙方已开展部分的费用，乙方可将已开展部分的项目成果交付给甲方。

6、 乙方应承担的所有补偿或赔偿等金额总额不超过本协议下甲方已向乙方支付的费用总额。除以上补偿或赔偿外，在任何情况下乙方均不承担甲方或者第三方的其他任何直接和间接的损失，也不承担任何连带赔偿责任。

7、 若甲方付款不及时所占用的时间将在项目周期上顺延，甲方每延期付款 1 个月，须按合同总金额 1%的延期补偿金赔付乙方；甲方拖延支付 3 个月以上，乙方有权随时单方面终止本协议，要求甲方立即支付该项目已经发生的所有费用，并有权追究甲方相应的违约责任，且未付款项目所涉及的所有成果归乙方所有。

8、 本协议一式两份，甲乙双方各持一份，每一份均具有同等法律效力。本协议自双方签字或盖章后生效。双方均确认可通过传真或电子邮件的方式签订协议，并一致认可传真件和扫描件的有效性。甲乙双方亦同意签署人以电子签名的方式签订本协议及相关附件，且认可该等电子协议、附件的法律效力，双方认可签署人在电子签名过程中提交的实名认证资料、短信验证码及其他行为产生的电子证据，且同意若因本协议的履行产生纠纷，该证据可直接作为纠纷处理依据。

9、 本协议的订立、效力、解释、履行、变更、终止和争议的解决均应适用中国法律。如因本协议产生纠纷，双方愿提交原告所在地人民法院管辖。

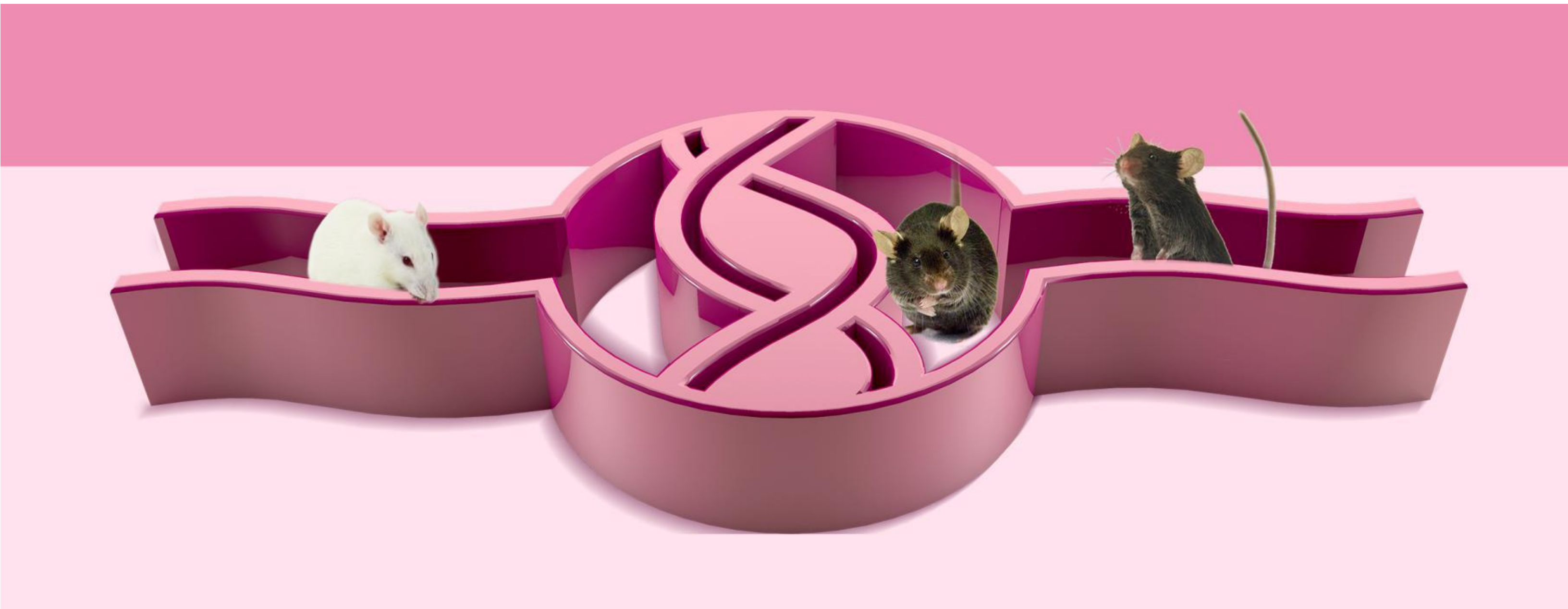

CREM-IbΔC-X PiggyBac Transgenic Project

Designer: Lindy Zhao

Reviewer: Beryl Bai

Design Date: 2022-04-01

Project Version: vA

Objective

To create a PiggyBac transgenic line expressing CREM-IbΔC-X under the control of alphaMHC_long promoter in FVB mouse.

Summary

1. The alphaMHC_long promoter (Mouse α-cardiac myosin heavy chain promoter (5.4 kb)) is a tissue_specific promoter.

2. The human CREM gene (NCBI Reference Sequence: NM_183011.2) is located on human chromosome 10.

3. In the PiggyBac vector, the “alphaMHC_long promoter-Kozak-HA tag-CREM-IbΔC-X-rBG pA” cassette will be flanked by two PiggyBac ITRs to facilitate transposes mediated transgene integration.

4. The PiggyBac vector will be co-injected with transposes into fertilized eggs from FVB mice.

5. The pups will be genotyped by PCR to identify the ones carrying the desired PiggyBac transgene.

6. The positive founder mice will be counter screened for transposes.

Template Needed	Cost of Purchase	Turnaround Time
HA tag-CREM-IbΔC-X (446 bp)	¥ 437	1-2 weeks

Vector Map

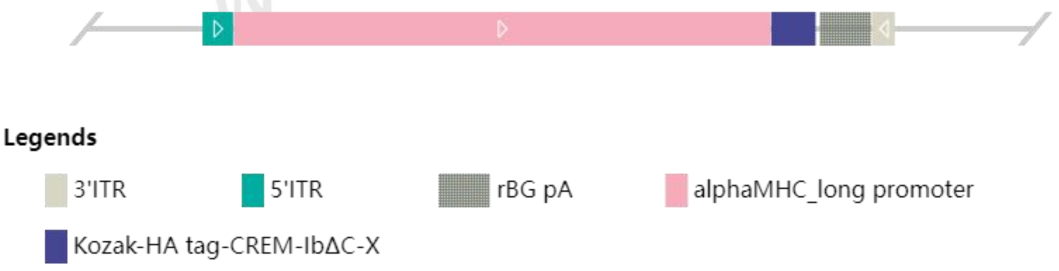

Gene and protein information

CREM cAMP responsive element modulator [*Homo sapiens* (human)]

Gene ID: 1390, updated on 27-Feb-2022

Gene summary

Official Symbol	CREMprovided by HGNC
Official Full Name	cAMP responsive element modulatorprovided by HGNC
Primary source	HGNC:HGNC:2352
See related	Ensembl:ENSG00000095794 MIM:123812; AllianceGenome:HGNC:2352
Gene type	protein coding
RefSeq status	REVIEWED
Organism	Homo sapiens
Lineage	Eukaryota; Metazoa; Chordata; Craniata; Vertebrata; Euteleostomi; Mammalia; Eutheria; Euarchontoglires; Primates; Haplorrhini; Catarrhini; Hominidae; Homo
Also known as	ICER; CREM-2; hCREM-2
Summary	This gene encodes a bZIP transcription factor that binds to the cAMP responsive element found in many viral and cellular promoters. It is an important component of cAMP-mediated signal transduction during the spermatogenetic cycle, as well as other complex processes. Alternative promoter and translation initiation site usage allows this gene to exert spatial and temporal specificity to cAMP responsiveness. Multiple alternatively spliced transcript variants encoding several different isoforms have been found for this gene, with some of them functioning as activators and some as repressors of transcription. [provided by RefSeq, Jul 2008]
Expression	Broad expression in testis (RPKM 19.2), adrenal (RPKM 15.7) and 21 other tissues See more
Orthologs	mouse all
NEW	**Try the new Gene table**
	**Try the new Transcript table**

Genomic context

Location: 10p11.21

See CREM in Genome Data Viewer

Exon count: 18

Annotation release	Status	Assembly	Chr	Location
109.20211119	current	GRCh38.p13 (GCF_000001405.39)	10	NC_000010.11 (35126841..35212958)
105.20201022	previous assembly	GRCh37.p13 (GCF_000001405.25)	10	NC_000010.10 (35415929..35501886)

Chromosome 10 - NC_000010.11

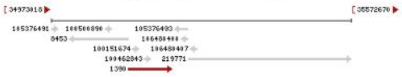

Transcripts: This gene has 48 transcripts

Gene: CREM ENSG00000095794

Description cAMP responsive element modulator [Source:HGNC Symbol;Acc:HGNC:2352]

Gene Synonyms hCREM-2

Location Chromosome 10: 35,126,791-35,212,958 forward strand.
GRCh38:CM000672.2

About this gene This gene has 48 transcripts (splice variants), 266 orthologues, 9 paralogues and is a member of 1 Ensembl protein family.

Transcripts

Name	Transcript ID	bp	Protein	Translation ID	Biotype	CCDS	UniProt	RefSeq Match	Flags
CREM-217	ENST00000439705.5	2306	248aa	ENSP00000409220.2	Protein coding	CCDS7182	Q03060	-	TSL:5GENCODE basic
CREM-204	ENST00000345491.7	2220	300aa	ENSP00000265372.5	Protein coding	CCDS7180	Q03060	-	TSL:1GENCODE basicAPPRIS P4
CREM-208	ENST00000361599.8	2170	270aa	ENSP00000354593.4	Protein coding	CCDS7185	Q03060	-	TSL:5GENCODE basicAPPRIS ALT1
CREM-212	ENST00000374728.7	1933	221aa	ENSP00000363860.3	Protein coding	CCDS7183	Q03060	-	TSL:5GENCODE basic
CREM-207	ENST00000356917.9	1895	108aa	ENSP00000349387.5	Protein coding	CCDS53518	Q03060	-	TSL:2GENCODE basic
CREM-205	ENST00000348787.6	1821	221aa	ENSP00000345384.2	Protein coding	CCDS7183	Q03060	-	TSL:1GENCODE basic
CREM-203	ENST00000344351.5	1625	95aa	ENSP00000344365.5	Protein coding	CCDS53522	E9PAR2	-	TSL:2GENCODE basic
CREM-206	ENST00000354759.7	1589	248aa	ENSP00000346804.3	Protein coding	CCDS7182	Q03060	-	TSL:1GENCODE basic
CREM-202	ENST00000342105.7	1541	245aa	ENSP00000341875.3	Protein coding	CCDS7186	Q03060	-	TSL:1GENCODE basic
CREM-218	ENST00000460270.5	1416	95aa	ENSP00000420437.1	Protein coding	CCDS53522	E9PAR2	-	TSL:2GENCODE basic
CREM-211	ENST00000374726.7	1207	137aa	ENSP00000363858.3	Protein coding	CCDS7184	Q03060	-	TSL:1GENCODE basic
CREM-228	ENST00000473940.5	1182	120aa	ENSP00000420681.1	Protein coding	CCDS7187	Q03060	-	TSL:1GENCODE basic
CREM-237	ENST00000488328.5	1049	109aa	ENSP00000417460.1	Protein coding	CCDS53519	Q03060	-	TSL:2GENCODE basic
CREM-229	ENST00000474362.5	1030	96aa	ENSP00000419018.1	Protein coding	CCDS53523	E9PB41	-	TSL:2GENCODE basic
CREM-220	ENST00000463314.5	964	138aa	ENSP00000418336.1	Protein coding	CCDS58075	Q03060	-	TSL:2GENCODE basic
CREM-201	ENST00000337656.8	900	299aa	ENSP00000337138.4	Protein coding	CCDS7181	Q03060	-	TSL:5GENCODE basicAPPRIS ALT1
CREM-214	ENST00000395887.7	849	282aa	ENSP00000379225.3	Protein coding	CCDS58074	Q03060	-	TSL:5GENCODE basicAPPRIS ALT1
CREM-239	ENST00000489321.5	731	137aa	ENSP00000419924.1	Protein coding	CCDS7184	Q03060	-	TSL:3GENCODE basic
CREM-213	ENST00000374734.7	711	236aa	ENSP00000363866.3	Protein coding	CCDS31181	Q03060	-	TSL:5GENCODE basicAPPRIS ALT1
CREM-238	ENST00000488741.5	651	102aa	ENSP00000419075.1	Protein coding	CCDS53521	Q03060	-	TSL:2GENCODE basic
CREM-230	ENST00000474931.5	626	112aa	ENSP00000417562.1	Protein coding	CCDS7188	Q03060	-	TSL:2GENCODE basic
CREM-224	ENST00000468236.5	582	125aa	ENSP00000419810.1	Protein coding	CCDS58076	Q03060	-	TSL:3GENCODE basic
CREM-242	ENST00000490511.1	469	113aa	ENSP00000417327.1	Protein coding	CCDS53520	Q03060	-	TSL:2GENCODE basic
CREM-236	ENST00000487763.5	429	121aa	ENSP00000417807.1	Protein coding	CCDS53517	Q03060	-	TSL:1GENCODE basic

CREM-210	ENST00000374721.7	2020	269aa	ENSP00000363853.3	Protein coding	-	G5E998	-	TSL:5GENCODE basic
CREM-216	ENST00000429130.7	1476	345aa	ENSP00000393538.2	Protein coding	-	J3KQC0 Q03060	-	TSL:5GENCODE basic
CREM-231	ENST00000479070.5	939	312aa	ENSP00000420511.1	Protein coding	-	Q03060	-	TSL:5GENCODE basic
CREM-226	ENST00000469949.6	721	59aa	ENSP00000473519.1	Protein coding	-	R4GN75	-	TSL:3GENCODE basic
CREM-234	ENST00000484283.5	660	219aa	ENSP00000417165.1	Protein coding	-	J3KR46	-	TSL:5GENCODE basic
CREM-235	ENST00000487132.5	655	186aa	ENSP00000418798.1	Protein coding	-	C9IYM9	-	CDS 3' incompleteTSL:5
CREM-215	ENST00000427847.6	635	156aa	ENSP00000403938.2	Protein coding	-	A0A0A0MSU8	-	CDS 3' incompleteTSL:3
CREM-221	ENST00000463960.5	582	193aa	ENSP00000419684.2	Protein coding	-	C9K092	-	CDS 5' incompleteTSL:5
CREM-243	ENST00000494479.5	563	115aa	ENSP00000417399.1	Protein coding	-	C9J2J0	-	CDS 3' incompleteTSL:3
CREM-244	ENST00000495301.1	555	78aa	ENSP00000417130.1	Protein coding	-	C9J5A7	-	CDS 3' incompleteTSL:4
CREM-241	ENST00000490460.5	1074	62aa	ENSP00000417058.1	Nonsense mediated decay	-	F8WB03	-	TSL:3
CREM-245	ENST00000495960.5	895	46aa	ENSP00000420453.1	Nonsense mediated decay	-	F8WDF2	-	TSL:5
CREM-222	ENST00000464475.1	453	53aa	ENSP00000418450.1	Nonsense mediated decay	-	H7C4X0	-	CDS 5' incompleteTSL:5
CREM-240	ENST00000489388.5	1022	No protein	-	Processed transcript	-	-	-	TSL:2
CREM-227	ENST00000472813.1	955	No protein	-	Processed transcript	-	-	-	TSL:5
CREM-225	ENST00000469517.1	910	No protein	-	Processed transcript	-	-	-	TSL:3
CREM-248	ENST00000497686.1	839	No protein	-	Processed transcript	-	-	-	TSL:3
CREM-219	ENST00000461968.5	747	No protein	-	Processed transcript	-	-	-	TSL:2
CREM-209	ENST00000374711.5	722	No protein	-	Processed transcript	-	-	-	TSL:5
CREM-233	ENST00000482646.5	715	No protein	-	Processed transcript	-	-	-	TSL:3
CREM-247	ENST00000496626.5	596	No protein	-	Processed transcript	-	-	-	TSL:4
CREM-246	ENST00000496019.5	563	No protein	-	Processed transcript	-	-	-	TSL:3
CREM-223	ENST00000466251.5	485	No protein	-	Processed transcript	-	-	-	TSL:5
CREM-232	ENST00000482633.5	415	No protein	-	Retained intron	-	-	-	TSL:2

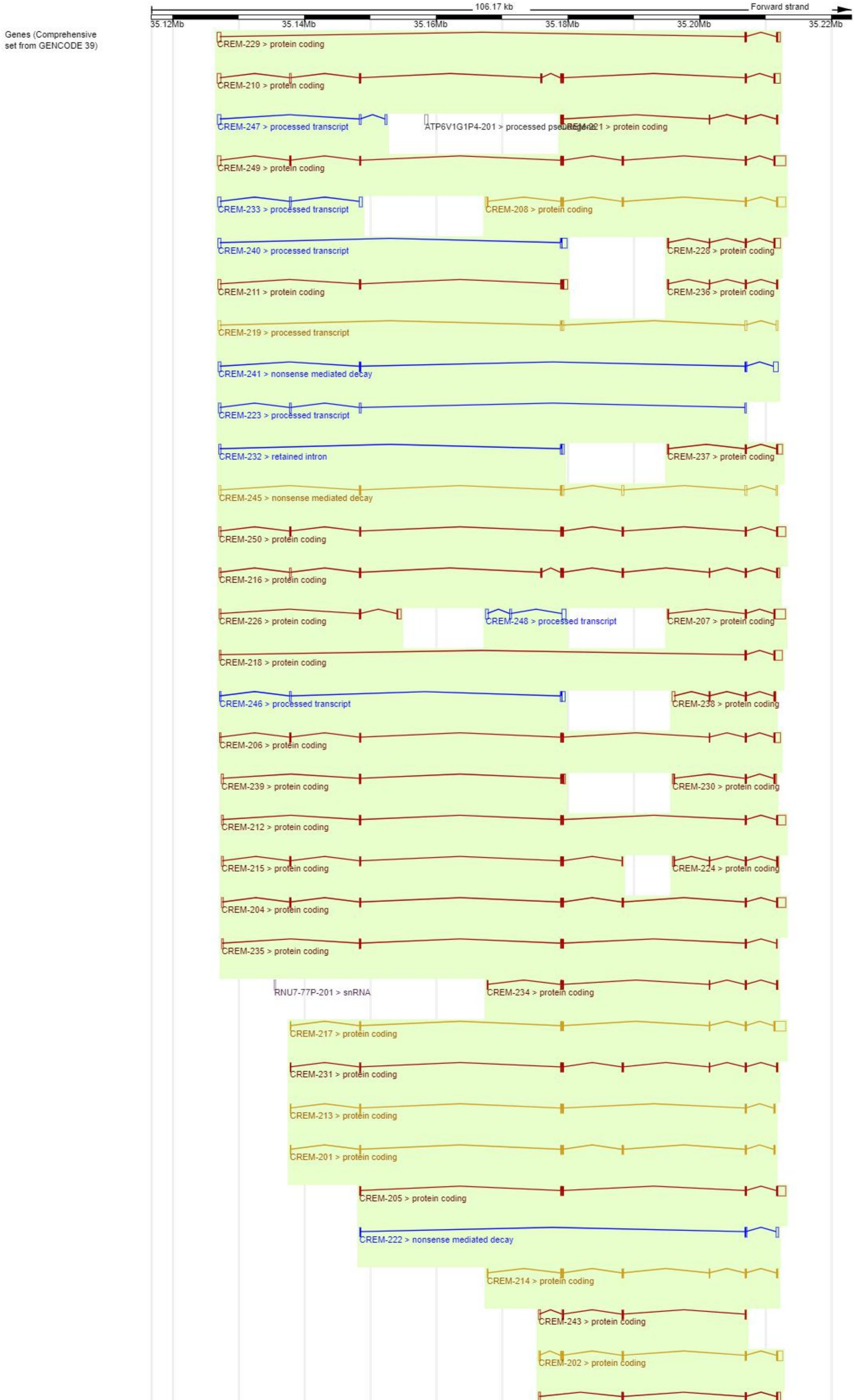
106.17 kb
Forward strand
35.12Mb
35.14Mb
35.16Mb
35.18Mb
35.20Mb
35.22Mb
Genes (Comprehensive set from GENCODE 39)
CREM-229 > protein coding
CREM-210 > protein coding
CREM-247 > processed transcript
ATP6V1G1P4-201 > processed pseudogene
CREM-221 > protein coding
CREM-249 > protein coding
CREM-233 > processed transcript
CREM-208 > protein coding
CREM-240 > processed transcript
CREM-228 > protein coding
CREM-211 > protein coding
CREM-236 > protein coding
CREM-219 > processed transcript
CREM-241 > nonsense mediated decay
CREM-223 > processed transcript
CREM-232 > retained intron
CREM-237 > protein coding
CREM-245 > nonsense mediated decay
CREM-250 > protein coding
CREM-216 > protein coding
CREM-226 > protein coding
CREM-248 > processed transcript
CREM-207 > protein coding
CREM-218 > protein coding
CREM-246 > processed transcript
CREM-238 > protein coding
CREM-206 > protein coding
CREM-239 > protein coding
CREM-230 > protein coding
CREM-212 > protein coding
CREM-215 > protein coding
CREM-224 > protein coding
CREM-204 > protein coding
CREM-235 > protein coding
RNU7-77P-201 > snRNA
CREM-234 > protein coding
CREM-217 > protein coding
CREM-231 > protein coding
CREM-213 > protein coding
CREM-201 > protein coding
CREM-205 > protein coding
CREM-222 > nonsense mediated decay
CREM-214 > protein coding
CREM-243 > protein coding
CREM-202 > protein coding

Gene expression/activity chart

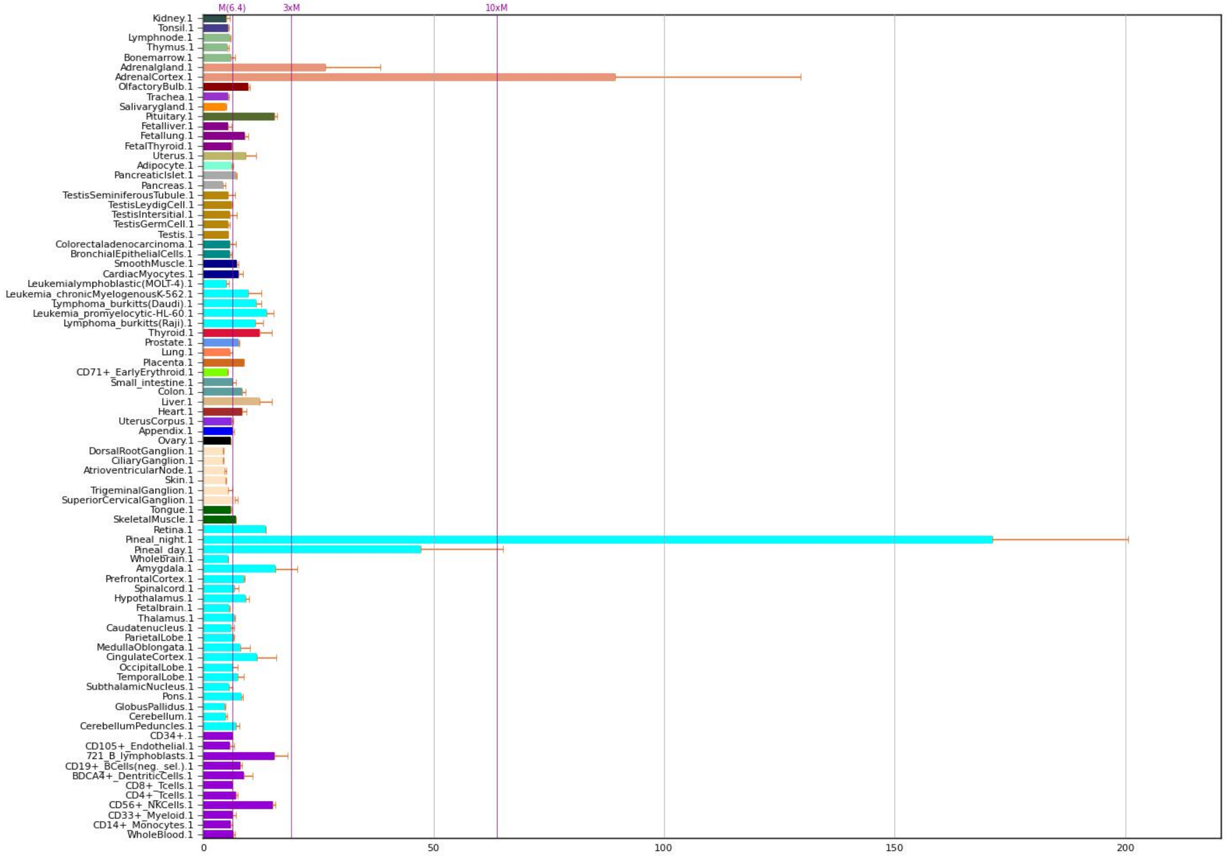

The sequence of alphaMHC_long promoter:

```
   1 GGTACC GGATCC TGCAAG GTCACA CAAGGG TCTCCA CCCACC AGGTGC CCTAGT CTCAAT
  61 TTCAGT TTCCAT GCCTTG TTCTCA CAATGC TGGCCT CCCCAG AGCTAA TTTGGA CTTTGT
 121 TTTTAT TTCAAA AGGGCC TGAATG AGGAGT AGATCT TGTGCT ACCCAG CTCTAA GGGTGC
 181 CCGTGA AGCCCT CAGACC TGGAGC CTTTGC AACAGC CCTTTA GGTGGA AGCAGA ATAAAG
 241 CAATTT TCCTTA AAGCCA AAATCC TGCCTC TAGACT CTTCTT CTCTGA CCTCGG TCCCTG
 301 GGCTCT AGGGTG GGGAGG TGGGGC TTGGAA GAAGAA GGTGGG GAAGTG GCAAAA GCCGAT
 361 CCCTAG GGCCCT GTGAAG TTCGGA GCCTTC CCTGTA CAGCAC TGGCTC ATAGAT CCTCCT
 421 CCAGCC AAACAT AGCAAG AAGTGA TACCTC CTTTGT GACTTC CCCAGG CCCAGT ACCTGT
 481 CAGGTT GAAACA GGATTT AGAGAA GCCTCT GAACTC ACCTGA ACTCTG AAGCTC ATCCAC
 541 CAAGCA AGCACC TAGGTG CCACTG CTAGTT AGTATC CTACGC TGATAA TATGCA GAGCTG
 601 GGCCAC AGAAGT CCTGGG GTGTAG GAACTG ACCAGT GACTTT TCAGTC GGCAAA GGTATG
 661 ACCCCC TCAGCA GATGTA GTAATG TCCCCT TAGATC CCATCC CAGGCA GGTCTC TAAGAG
 721 GACATG GGATGA GAGATG TAGTCA TGTGGC ATTCCA AACACA GCTATC CACAGT GTCCCT
 781 TGCCCC TTCCAC TTAGCC AGGAGG ACAGTA ACCTTA GCCTAT CTTTCT TCCTCC CCATCC
 841 TCCCAG GACACA CCCCCT GGTCTG CAGTAT TCATTT CTTCCT TCACGT CCCCTC TGTGAC
 901 TTCCAT TTGCAA GGCTTT TGACCT CTGCAG CTGCTG GAAGAT AGAGTT TGGCCC TAGGTG
 961 TGGCAA GCCATC TCAAGA GAAAGC AGACAA CAGGGG GACCAG ATTTTG GAAGGA TCAGGA
1021 ACTAAA TCACTG GCGGGC CTGGGG GTAGAA AAAAGA GTGAGT GAGTCC GCTCCA GCTAAG
1081 CCAAGC TAGTCC CCGAGA TACTCT GCCACA GCTGGG CTGCTC GGGGTA GCTTTA GGAATG
1141 TGGGTC TGAAAG ACAATG GGATTG GAAGAC ATCTCT TTGAGT CTCCCC TCAACC CCACCT
1201 ACAGAC ACACTC GTGTGT GGCCAG ACTCCT GTTCAA CAGCCC TCTGTG TTCTGA CCACTG
1261 AGCTAG GCAACC AGAGCA TGGGCC CTGTGC TGAGGA TGAAGA GTTGGT TACCAA TAGCAA
1321 AAACAG CAGGGG AGGGAG AACAGA GAACGA AATAAG GAAGGA AGAAGG AAAGGC CAGTCA
1381 ATCAGA TGCAGT CAGAAG AGATGG GAAGCC AACACA CAGCTT GAGCAG AGGAAA CAGAAA
1441 AGGGAG AGATTC TGGGCA TAAGGA GGCCAC AGAAAG AAGAGC CCAGGC CCCCCA AGTCTC
1501 CTCTTT ATACCC TCATCC CGTCTC CCAATT AAGCCC ACTCTT CTTCCT AGATCA GACCTG
1561 AGCTGC AGCGAA GAGACC CGTAGG GAGGAT CACACT GGATGA AGGAGA TGTGTG GAGAAG
1621 TCCAGG GAACCT AAGAGC CAGAGC CTAAAA GAGCAA GAGATA AAGGTG CTTCAA AGGTGG
1681 CCAGGC TGTGCA CACAGA GGGTCG AGGACT GGTGGT AGAGCC TCAAGA TAAGGA TGATGC
1741 TCAGAA TGGGCG GGGGGG GGGATT CTGGGG GGGGGA GAGAGA AGGTGA GAAGGA GCCTGG
1801 AACAGA GAATCT GGAAGC GCTGGA AACGAT ACCATA AAGGGA AGAACC CAGGCT ACCTTT
1861 AGATGT AAATCA TGAAAG ACAGGG AGAAGG GAAGCT GGAGAG AGTAGA AGGACC CCGGGG
1921 CAAGAC ATTGAA GCAAGG ACAAGC CAGGTT GAGCGC TCCGTG AAATCA GCCTGC TGAAGG
1981 CAGAGC CCTGGT ATGAGC ACCAGA ACAGCA GAGGCT AGGGTT AATGTC GAGACA GGGAAC
2041 AGAAGG TAGACA CAGGAA CAGACA GAGACG GGGGAG CCAGGT AACAAA GGAATG GTCCTT
2101 CTCACC TGTGGC CAGAGC GTCCAT CTGTGT CCACAT ACTCTA GAATGT TCATCA GACTGC
2161 AGGGCT GGCTTG GGAGGC AGCTGG AAAGAG TATGTG AGAGCC AGGGGA GACAAG GGGGCC
2221 TAGGAA AGGAAG AAGAGG GCAAAC CAGGCC ACACAA GAGGGC AGAGCC CAGAAC TGAGTT
2281 AACTCC TTCCTT GTTGCA TCTTCC ATAGGA GGCAGT GGGAAC TCTGTG ACCACC ATCCCC
2341 CATGAG CCCCCA CTACCC ATACCA AGTTTG GCCTGA GTGGCA TTCTAG GTTCCC TGAGGA
```

2401 CAGAGC CTGGCC TTTGTC TCTTGG ACCTGA CCCAAG CTGACC CAATGT TCTCAG TACCTT
2461 ATCATG CCCTCA AGAGCT TGAGAA CCAGGC AGTGAC ATATTA GGCCAT GGGCTA ACCCTG
2521 GAGCTT GCACAC AGGAGC CTCAAG TGACCT CCAGGG ACACAG CTGCAG ACAGGT GGCCTT
2581 TATCCC CAAAGA GCAACC ATTTGG CATAGG TGGCTG CAAATG GGAATG CAAGGT TGAATC
2641 AGGTCC CTTCAA GAATAC TGCATG CAAGAC CTAAGA CCCCTG GAGAGA GGGGTA TGCTCC
2701 TGCCCC CACCCA CCATAA GGGGAG TGAACT ATCCTA GGGGGC TGGCGA CCTTGG GGAGAC
2761 ACCACA TTACTG AGAGTG CTGAGC CCAGAA AAACTG ACCGCC CTGTGT CCTGCC CACCTC
2821 CACACT CTAGAG CTATAT TGAGAG GTGACA GTAGAT AGGGTG GGAGCT GGTAGC AGGGAG
2881 AGTGTT CCTGGG TGTGAG GGTGTA GGGGAA AGCCAG AGCAGG GGAGTC TGGCTT TGTCTC
2941 CTGAAC ACAATG TCTACT TAGTTA TAACAG GCATGA CCTGCT AAAGAC CCAACA TCTACG
3001 ACCTCT GAAAAG ACAGCA GCCCTG GAGGAC AGGGGT TGTCTC TGAGCC TTGGGT GCTTGA
3061 TGGTGC CACAAA GGAGGG CATGAG TGTGAG TATAAG GCCCCA GGAGCG TTAGAG AAGGGC
3121 ACTTGG GAAGGG GTCAGT CTGCAG AGCCCC TATCCA TGGAAT CTGGAG CCTGGG GCCAAC
3181 TGGTGT AAATCT CTGGGC CTGCCA GGCATT CAAAGC AGCACC TGCATC CTCTGG CAGCCT
3241 GGGGAG GCGGAA GGGAGC AACCCC CCACTT ATACCC TTTCTC CCTCAG CCCCAG GATTAA
3301 CACCTC TGGCCT TCCCCC TTCCCA CCTCCC ATCAGG AGTGGA GGGTTG CAGAGG GAGGGT
3361 AAAAAC CTACAT GTCCAA ACATCA TGGTGC ACGATA TATGGA TCAGTA TGTGTA GAGGCA
3421 AGAAAG GAAATC TGCAGG CTTAAC TGGGTT AATGTG TAAAGT CTGTGT GCATGT GTGTGT
3481 GTCTGA CTGAAA ACGGGC ATGGCT GTGCAG CTGTTC AGTTCT GTGCGT GAGGTT ACCAGA
3541 CTGCAG GTTTGT GTGTAA ATTGCC CAAGGC AAAGTG GGTGAA TCCCTT CCATGG TTTAAA
3601 GAGATT GGATGA TGGCCT GCATCT CAAGGA CCATGG AAAATA GAATGG ACACTC TATATG
3661 TGTCTC TAAGCT AAGGTA GCAAGG TCTTTG GAGGAC ACCTGT CTAGAG ATGTGG GCAACA
3721 GAGACT ACAGAC AGTATC TGTACA GAGTAA GGAGAG AGAGGA GGGGGT GTAGAA TTCTCT
3781 TACTAT CAAAGG GAAACT GAGTCG TGCACC TGCAAA GTGGAT GCTCTC CCTAGA CATCAT
3841 GACTTT GTCTCT GGGGAG CCAGCA CTGTGG AACTTC AGGTCT GAGAGA GTAGGA GGCTCC
3901 CCTCAG CCTGAA GCTATG CAGATA GCCAGG GTTGAA AGGGGG AAGGGA GAGCCT GGGATG
3961 GGAGCT TGTGTG TTGGAG GCAGGG GACAGA TATTAA GCCTGG AAGAGA AGGTGA CCCTTA
4021 CCCAGT TGTTCA ACTCAC CCTTCA GATTAA AAATAA CTGAGG TAAGGG CCTGGG TAGGGG
4081 AGGTGG TGTGAG ACGCTC CTGTCT CTCCTC TATCTG CCCATC GGCCCT TTGGGG AGGAGG
4141 AATGTG CCCAAG GACTAA AAAAAG GCCATG GAGCCA GAGGGG CGAGGG CAACAG ACCTTT
4201 CATGGG CAAACC TTGGGG CCCTGC TGTCCT CCTGTC ACCTCC AGAGCC AAGGGA TCAAAG
4261 GAGGAG GAGCCA GGACAG GAGGGA AGTGGG AGGGAG GGTCCC AGCAGA GGACTC CAAATT
4321 TAGGCA GCAGGC ATATGG GATGGG ATATAA AGGGGC TGGAGC ACTGAG AGCTGT CAGAGA
4381 TTTCTC CAACCC AGGTAA GAGGGA GTTTCG GGTGGG GGCTCT TCACCC ACACCA GACCTC
4441 TCCCCA CCTAGA AGGAAA CTGCCT TTCCTG GAAGTG GGGTTC AGGCCG GTCAGA GATCTG
4501 ACAGGG TGGCCT TCCACC AGCCTG GGAAGT TCTCAG TGGCAG GAGGTT TCCACA AGAAAC
4561 ACTGGA TGCCCC TTCCCT TACGCT GTCTTC TCCATC TTCCTC CTGGGG ATGCTC CTCCCC
4621 GTCTTG GTTTAT CTTGGC TCTTCG TCTTCA GCAAGA TTTGCC CTGTGC TGTCCA CTCCAT
4681 CTTTCT CTACTG TCTCCG TGCCTT GCCTTG CCTTCT TGCGTG TCCTTC CTTTCC ACCCAT
4741 TTCTCA CTTCAC CTTTTC TCCCCT TCTCAT TTGTAT TCATCC TTCCTT CCTTCC TTCCTT
4801 CCTTCC TTCCTT CCTTCC TTCCTT CCTTTC TCCCTT CCTTCC TTCCTT CCTTCC TTCCTT

4861 CCTTCC TTCCTT CCTGTG TCAGAG TGCTGA GAATCA CACCTG GGGTTC CCACCC TTATGT
4921 AAACAA TCTTCC AGTGAG CCACAG CTTCAG TGCTGC TGGGTG CTCTCT TACCTT CCTCAC
4981 CCCCTG GCTTGT CCTGTT CCATCC TGGTCA GGATCT CTAGAT TGGTCT CCCAGC CTCTGC
5041 TACTCC TCTTCC TGCCTG TTCCTC TCTCTG TCCAGC TGCGCC ACTGTG GTGCCT CGTTCC
5101 AGCTGT GGTCCA CATTCT TCAGGA TTCTCT GAAAAG TTAACC AGGTGA GAATGT TTCCCC
5161 TGTAGA CAGCAG ATCACG ATTCTC CCGGAA GTCAGG CTTCCA GCCCTC TCTTTC TCTGCC
5221 CAGCTG CCCGGC ACTCTT AGCAAA CCTCAG GCACCC TTACCC CACATA GACCTC TGACAG
5281 AGAAGC AGGCAC TTTACA TGGAGT CCTGGT GGGAGA GCCATA GGCTAC GGTGTA AAAGAG
5341 GCAGGG AAGTGG TGGTGT AGGAAA GTCAGG ACTTCA CATAGA AGCCTA GCCCAC ACCAGA
5401 AATGAC AGACAG ATCCCT CCTATC TCCCCC ATAAGA GTTTGA GTCGAC CCGCGG CCCCGA
5461 ATTG

The sequence of HA tag-CREM-IbΔC-X (https://faseb.onlinelibrary.wiley.com/doi/full/10.1096/fasebj.12.12.1191):

1 ATGTAC CCATAC GATGTT CCAGAT TACGCT GAAACA GTTGAA TCCCAG CATGAT GGAAGT
61 ATAACA GCTTCT TTGACA GAGAGC AAGTCT GCTCAT GTGCAG ACTCAG ACTGGC CAAAAT
121 TCAATC CCTGCT TTAGCT CAGCTG CCACTG GTGACA TGCCAA CTTACC AGATCC GAGCTC
181 CTACTG CTGCTT TGCCAC AGGGAG TGGTGA TGGCTG CATCGC CCGGAA GTTTGC ACAGTC
241 CCCAGC AGCTGG CAGAAG AAGCAA CACGCA AACGAG AGCTGA GGCTAA TGAAAA ACAGGG
301 AAGCTG CCAAAG AATGTC GACGTC GAAAGA AAGAAT ATGTAA AATGTC TGGAGA GCCGAG
361 TTGCAG TGCTGG AAGTCC AGAACA AGAAGC TTATAG AGGAAC TTGAAA CCTTGA AAGACA
421 TTTGTT CTCCCA AAACAG ATTACT AG

The sequence of rBG pA:

1 TCCTCA GGTGCA GGCTGC CTATCA GAAGGT GGTGGC TGGTGT GGCCAA TGCCCT GGCTCA
61 CAAATA CCACTG AGATCT TTTTCC CTCTGC CAAAAA TTATGG GGACAT CATGAA GCCCCT
121 TGAGCA TCTGAC TTCTGG CTAATA AAGGAA ATTTAT TTTCAT TGCAAT AGTGTG TTGGAA
181 TTTTTT GTGTCT CTCACT CGGAAG GACATA TGGGAG GGCAAA TCATTT AAAACA TCAGAA
241 TGAGTA TTTGGT TTAGAG TTTGGC AACATA TGCCCA TATGCT GGCTGC CATGAA CAAAGG
301 TTGGCT ATAAAG AGGTCA TCAGTA TATGAA ACAGCC CCCTGC TGTCCA TTCCTT ATTCCA
361 TAGAAA AGCCTT GACTTG AGGTTA GATTTT TTTTAT ATTTTG TTTTGT GTTATT TTTTTC
421 TTTAAC ATCCCT AAAATT TTCCTT ACATGT TTTACT AGCCAG ATTTTT CCTCCT CTCCTG
481 ACTACT CCCAGT CATAGC TGTCCC TCTTCT CTTATG GAGATC

cyagen We help you discover life

甲方课题组负责人确认，已经知晓并同意上述方案的全部内容，对此无任何异议。

甲方负责人签字确认：

日期：　　年　月　日
